# MePMe-seq: antibody-free simultaneous m^6^A and m^5^C mapping in mRNA by metabolic propargyl labeling and sequencing

**DOI:** 10.1038/s41467-023-42832-z

**Published:** 2023-11-07

**Authors:** Katja Hartstock, Nadine A. Kueck, Petr Spacek, Anna Ovcharenko, Sabine Hüwel, Nicolas V. Cornelissen, Amarnath Bollu, Christoph Dieterich, Andrea Rentmeister

**Affiliations:** 1 Institute of Biochemistry, Faculty of Chemistry and Pharmacy, University of Münster, Corrensstraße 36, 48149 Münster, Germany; 2 Section of Bioinformatics and Systems Cardiology, Klaus Tschira Institute for Integrative Computational Cardiology, Heidelberg, Germany; 3 Department of Internal Medicine III (Cardiology, Angiology, and Pneumology), University Hospital Heidelberg, Heidelberg, Germany; 4 DZHK (German Centre for Cardiovascular Research), Partner Site Heidelberg/Mannheim, Berlin, Germany

**Keywords:** Next-generation sequencing, RNA, RNA modification, RNA sequencing, Methylation

## Abstract

Internal modifications of mRNA have emerged as widespread and versatile regulatory mechanism to control gene expression at the post-transcriptional level. Most of these modifications are methyl groups, making *S*-adenosyl-L-methionine (SAM) a central metabolic hub. Here we show that metabolic labeling with a clickable metabolic precursor of SAM, propargyl-selenohomocysteine (PSH), enables detection and identification of various methylation sites. Propargylated A, C, and G nucleosides form at detectable amounts via intracellular generation of the corresponding SAM analogue. Integration into next generation sequencing enables mapping of *N*^6^-methyladenosine (m^6^A) and 5-methylcytidine (m^5^C) sites in mRNA with single nucleotide precision (MePMe-seq). Analysis of the termination profiles can be used to distinguish m^6^A from 2′-*O*-methyladenosine (A_m_) and *N*1-methyladenosine (m^1^A) sites. MePMe-seq overcomes the problems of antibodies for enrichment and sequence-motifs for evaluation, which was limiting previous methodologies. Metabolic labeling via clickable SAM facilitates the joint evaluation of methylation sites in RNA and potentially DNA and proteins.

## Abstract (web summary)

Methylation is the dominant modification in mRNA and occurs at a variety of sites. Here, Hartstock et al. show that a clickable analogue of the key cosubstrate *S*-adenosyl-L-methionine (SAM) can be produced in cells, allowing for identification and mapping of different methylated nucleosides in mRNA.

## Introduction

Eukaryotic mRNA is canonically modified by the addition of the 5ʹ cap and bears additional modifications at internal sites. The *N*^6^-methylation of adenosine (m^6^A) is the most abundant and best-studied internal modification of mRNA. It has been linked to cellular differentiation, cancer progression, development and ageing^1–7^. Most of the more than 12,000 sites are introduced by the METTL3-14 complex, whereas METTL16 is responsible for six additional validated sites in mRNA^8–12^. Several reader proteins have been identified and mediate the effects of m^6^A in mRNA translation and degradation^13–25^. m^5^C is another internal modification in mammalian mRNA. The reported number of sites ranges from a few hundred to 40,000 sites, and various writer proteins (NSUN2, NSUN6, and TRDMT1) have been identified for mammalian cells^26–29^. Several reader proteins have been found, linking m^5^C to repair (via RAD52), export (via ALYREF), and proliferation (via YBX1 and ELAV) causing bladder cancer^30–34^. In total, ten different internal modifications of eukaryotic mRNA have been described and mapped^35^. In addition to m^6^A and m^5^C, these comprise the altered nucleobases inosine and pseudouridine, the acetylation ac^4^C, the oxidation 8-oxo-G and further methylations or derivatives thereof at either the nucleobase (m^1^A, m^7^G, hm^5^C) or the ribose (N_m_). The prevalence of methylation as mRNA modification mark is striking, suggesting that the responsible cofactor SAM plays a key role for their abundance and potential interconnectivity^36^.

Owing to the importance and abundance of m^6^A, multiple approaches have been developed to assign its positions on a transcriptome-wide level. Most methods rely on antibodies in combination with next-generation sequencing (NGS). While early transcriptome-wide detection methods had limited resolution^37–39^, crosslinking and bioinformatic analyses including search for the DRACH motif, improved the accuracy of single nucleotides^40–42^. Concerns regarding bias of antibodies in recognizing the tiny nucleobase as epitope along with the inability to distinguish between m^6^A and m^6^A_m_ have prompted the development of antibody-free methods. DART-seq uses a YTH reader protein for m^6^A recognition and introduces adjacent C-to-U mutations by a fused deaminase^43,44^. MAZTER seq relies on the methylation-sensitive ribonuclease and bioinformatic alignment of cleaved versus uncleaved sequences at its target ACA sites^45,46^. m^6^A-SEAL uses m^6^A-specific methyl oxidation by FTO for further derivatization and enrichment^47^. m^6^A-REF-seq combines m^6^A demethylation by FTO and cleavage of ACA-sites with a m^6^A-sensitive RNA endonuclease^48^. eTAM-seq uses TadA for specific deamination of A to induce an A-to-I conversion during reverse transcription (RT)^49^. m^6^A-SAC-seq relies on *N*^6^-allylation of m^6^A using the methyltransferase (MTase) MjDim1 followed by *N*1-*N*^6^-cyclization, which leads to a mismatch in RT^50^. These methods rely on exogenous enzymes for modification, which bring about their own biases, such as preference for or even limitation to certain sequences. Furthermore, some of these approaches require transfection of cells. GLORI is a different recently released methodology. This approach is ideal for transcriptome-wide localization of m^6^A with single-nucleotide precision and quantification and also relies on deamination of A utilizing glyoxal and nitrite treatment instead of enzymes^51^. In summary, there is a plethora of methods for mapping m^6^A sites, however, most of them cannot be used to assign other methylation sites.

Metabolic labeling with methionine (Met) analogues presents an interesting alternative approach for m^6^A detection^52,53^. After feeding cells with PSH, modified adenosines in rRNA could be detected via click chemistry and enrichment^53^. Label-Seq determined m^6^A-sites in mRNA by feeding allyl-selenohomocysteine followed by a highly specific cyclization reaction of the resulting *N*^6^-allyladenosine, causing mutations in RT identified by NGS. However, an antibody was required to enrich the allyl-modified mRNA and other modified nucleosides were not detected^52^.

For m^5^C, another abundant internal modification of mRNA, chemical conversion of C to U in bisulfite sequencing is frequently used for mapping^54,55^. This treatment risks damaging RNA and causing artifacts, necessitating careful and repeated controls to obtain reliable data^56^. Antibody-based methods with and without photo-crosslinking have been developed in analogy to m^6^A mapping methods and underlie the same limitations^26,57,58^. The development of antibody-free methods includes progress towards nanopore sequencing and TAWO-seq, but the latter has not yet been implemented on a transcriptome-wide scale^59,60^.

Taken together, both m^6^A and m^5^C as well as other methylations rely on the cofactor SAM, suggesting that the methyl-based modifications could be interconnected via SAM levels, and it would be important to study them in context^36^. Yet, current methodology has mainly focused on specific binding and detection of the modified nucleoside instead of the underlying and unifying process. The enrichment via antibodies or binding proteins, or the specific modification by m^6^A-sensitive enzymes counteracts a more global look at possible links.

**Fig. 1:**
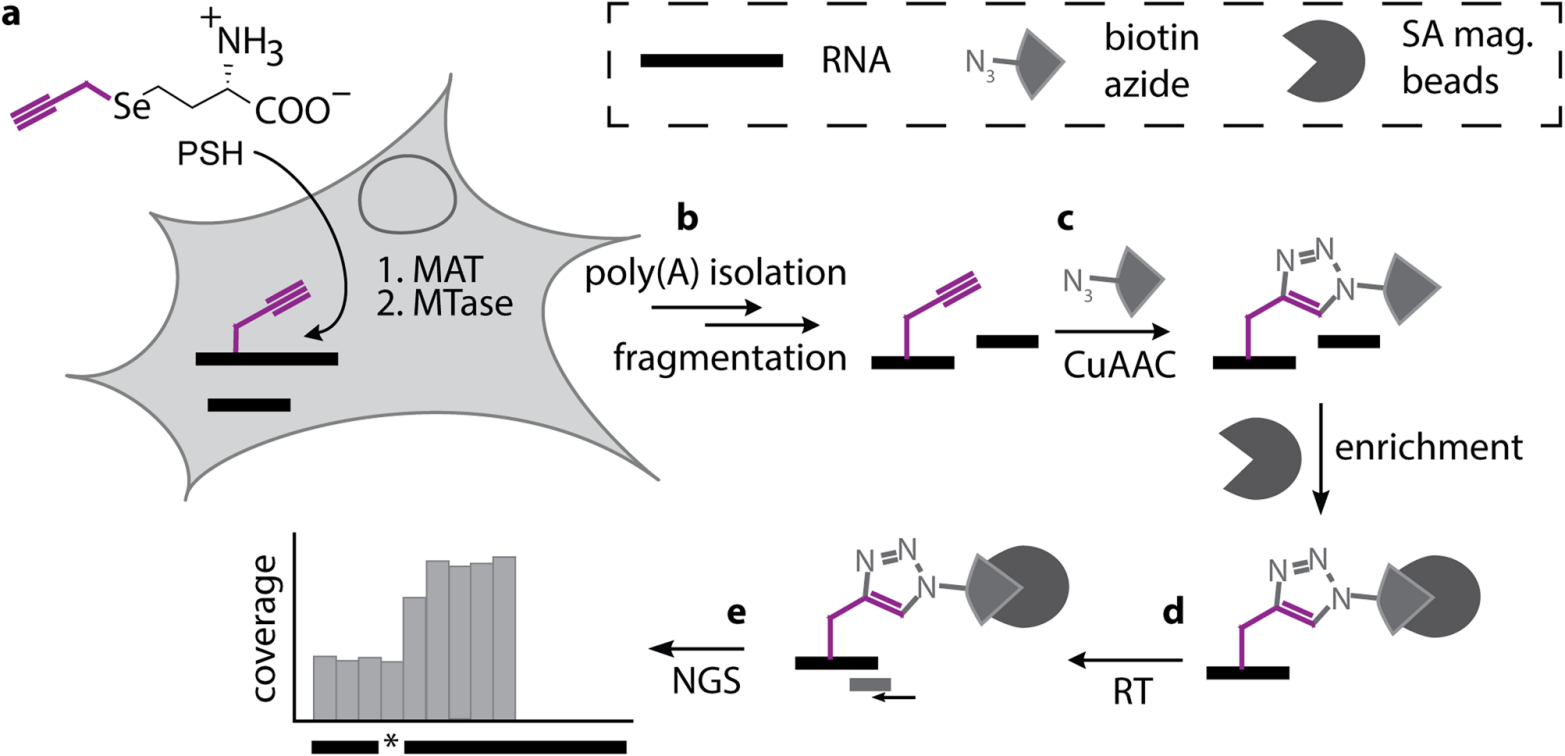
Scheme of MePMe-seq (metabolic propargylation for methylation sequencing). **a** Metabolic labeling of cells with PSH leads to methionine adenosyl transferase (MAT)-catalyzed formation of SAM-analogue and propargylation of methyltransferase (MTase) target sites. **b** After cell lysis, poly(A)^+^ RNA is isolated and fragmented. **c** Propargylated fragments react with biotin azide in a copper-catalyzed azide-alkyne cycloaddition (CuAAC) and are enriched on streptavidin-coated magnetic beads (SA mag. beads). **d** On-bead reverse transcription (RT) terminates at modified sites. **e** Libraries for next generation sequencing (NGS) are prepared. Modified sites are detected as coverage drops with distinct termination profiles.

In this work, we therefore set out to develop metabolic labeling via the SAM pathway as methodology to detect more than one type of modified nucleoside by NGS. Such methodology should hinge on a SAM analogue that (1) can be efficiently made in genetically unaltered mammalian cells, (2) is accepted by promiscuous activity of several MTases, and (3) provides a universal handle for efficient antibody-free enrichment of different nucleosides to (4) make modified nucleosides amenable to detection in NGS. A perfect metabolite is the propargylic SAM analogue SeAdoYn, which is readily produced in cells with unaltered genetic makeup and is recognized by most MTases^53,61^. Above all, the propargyl group is bioorthogonal and specifically reacts with azides in a click reaction, making it possible to chemically enrich target RNA without the need for antibodies (Fig. 1a–e).

## Results

### Metabolic labeling and quantification of propargylated nucleosides

**Fig. 2:**
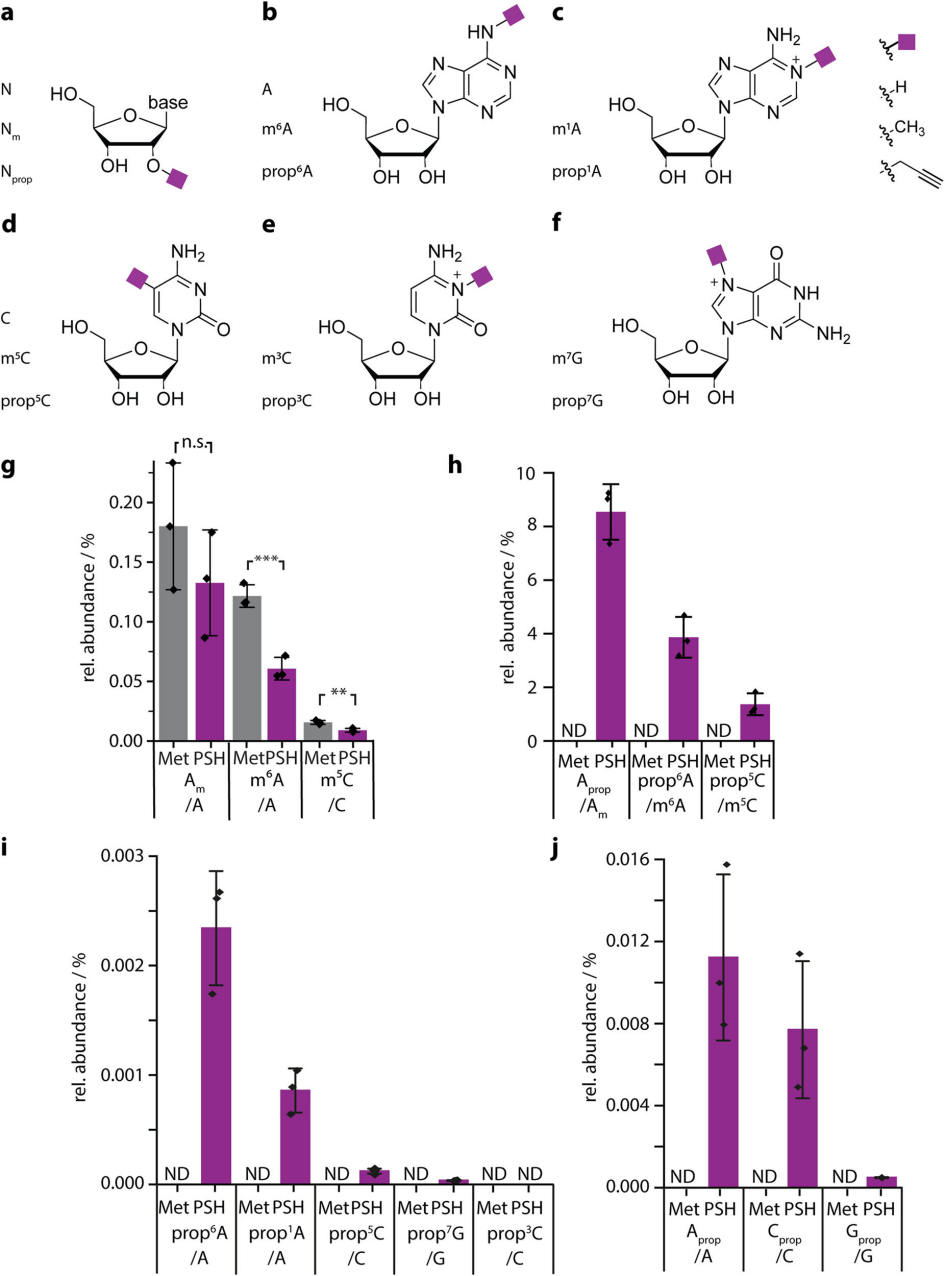
Metabolic labeling of mRNA via PSH in HeLa cells. PSH metabolism modifies RNA with propargyl (prop) at positions naturally found to be methylated. **a**–**f** Modified and unmodified nucleosides investigated by LC-QqQ-MS. Structures of (**a**) a nucleoside (N), a 2′-*O*-methylated nucleoside (N_m_), a 2′-*O*-propargylated nucleoside (N_prop_), (**b**) *N*^6^-methyl adenosine (m^6^A), *N*^6^-propargyl adenosine, (**c**) *N*1-methyl adenosine (m^1^A), *N*1-propargyl adenosine (prop^1^A), (**d**) cytidine (C), 5-methylcytidine (m^5^C), 5-propargylcytidine (prop^5^C), (**e**) *N*3-methyl cytidine (m^3^C), *N*3-propargyl cytidine (prop^3^C), (**f**) *N*7-methyl guanosine (m^7^G), *N*7-propargyl guanosine (prop^7^G). **g**–**j**, Quantification of modified nucleosides in poly(A)^+^ RNA from HeLa cells treated with 2.5 mM PSH (purple) or methionine (gray) as control. Relative abundance of (**g**) A_m_, m^6^A and m^5^C, (**h**) A_prop_, prop^6^A and prop^5^C with respect to the methylated nucleoside, (**i**) prop^6^A, prop^1^A, prop^5^C, prop^7^G and prop3C, (**j**) A_prop_, C_prop_ and G_prop_. Quantification from dynamic MRM run on LC-QqQ-MS using external synthetic standards. Not detected (ND): no signal with correct quantifier detected. Mean values and SD from *n* = 3 biological replicates are shown. Statistical significance determined via unpaired two-sample two-tailed *t* test (n.s. *P* > 0.05; *P ≤ 0.05; **P ≤ 0.01; ***P ≤ 0.001). The *P* value for A_m_/A Met versus A_m_/A PSH is 0.30, for m^6^A/A Met versus m^6^A/A PSH it is 7.0 × 10^−4^, for m^5^C/C Met versus m^5^C/C PSH it is 0.004. Source data are provided as a Source Data file.

Metabolic labeling with PSH embarks on the natural methylation pathways and the broad scope of nucleoside modifications. Previous work showed that many MTases are promiscuous regarding the cosubstrate and transfer also so-called bioorthogonal groups to the natural methylation sites, as validated for select RNA and histone modifications^53,62,63^. However, the potential to investigate more than one type of modification has not been exploited, as the level of most non-natural modifications remains low^64^. We therefore sought to maximize the level of metabolic RNA propargylation while maintaining cell viability and treated HeLa cells with different concentrations of PSH or Met (Supplementary Fig. 1a). As proxy for the general propargylation level of RNA, we quantified A_prop_ (Fig. 2a) in total RNA using LC-QqQ-MS and found that the level increased with higher concentrations of PSH up to 2.5 mM (Supplementary Fig. 1b, c). Under these conditions, 2.2% of A_m_ were substituted by A_prop_ in total RNA and the levels of the natural methylation A_m_ and m^6^A remained largely unaffected by metabolic PSH labeling (A_m_/A ~ 4%, m^6^A/A 0.3%; Supplementary Fig. 1d, e). These values are in line with literature reports for total RNA, suggesting that the general cellular methylation itself is not perturbed^56,65–67^. The cell viability was only slightly compromised by metabolic labeling with PSH, remaining at 81% at 2.5 mM PSH with respect to untreated cells (Supplementary Fig. 1f). Controls were treated identically but using Met instead of PSH.

Next, we focused on propargylation and methylation levels in mRNA after two times of poly(A)-enrichment, which is reported to reduce N_m_ originating from rRNA by 10–20-fold but maintain m^6^A, which is abundant in mRNA^56,65^. We found a ~20-fold reduction of A_m_ (3.8% vs. 0.18%) compared to total RNA, whereas the m^6^A level remained almost constant (0.3% vs. 0.12%) (Fig. 2g, Supplementary Fig. 1d, e), suggesting successful enrichment of mRNA, albeit with residual rRNA, as expected^67^. Metabolic labeling with PSH did not significantly affect the A_m_/A ratio in poly(A)^+^ RNA (0.13–0.18%, Fig. 2g), however, the m^6^A/A levels in these samples were reduced from 0.12% to 0.06% (Fig. 2g). This suggests a stronger effect of metabolic labeling on mRNAs, which have a shorter half-life than rRNAs.

Analyzing the propagylation by LC-QqQ-MS, we observed a ~10-fold reduction of the A_prop_ level after poly(A)-enrichment (0.011% vs. 0.10%), which is in line with the observed A_m_ depletion (Fig. 2j, Supplementary Fig. 1b). In poly(A)^+^ RNA from labeled cells, prop^6^A was unambiguously detected, whereas it was not detectable in control cells (Fig. 2i). It replaced 3.5% of m^6^A (Fig. 2h) and was present in 0.0023% of all As (Fig. 2i). Taken together, these data indicate that PSH can be used for metabolic labeling and enables detection of prop^6^A and A_prop_ in poly(A)^+^ RNA under conditions retaining cell viability.

As metabolic labeling with PSH via the respective SAM analogues would be expected to propargylate also other nucleosides at various positions, we investigated whether additional propargylated nucleosides could be detected in poly(A)^+^ RNA. Current work agrees that mRNA contains m^6^A and m^5^C in addition to various methylations at the 5′ cap and suggests that m^1^A, m^7^G and m^3^C as well as ribose methylation (N_m_) may exist, albeit with controversial reports^68^. To test for the respective propargylated nucleosides, we assembled a panel of nine propargyl-containing nucleosides that—in addition to prop^6^A and A_prop_—contained all sugar modified nucleosides (N_prop_) as well as prop^5^C, prop^1^A, prop^7^G, and prop^3^C for which natural methylation had been reported. Synthesis of the synthetic standards prop^5^C, prop^1^A, prop^3^C and prop^7^G (Fig. 2c–f) is described in Supplementary Methods. C_prop_, G_prop_, prop^5^U and U_prop_ are commercially available. We then developed LC-QqQ-MS methods for all synthetic standards and analyzed cellular poly(A)^+^ RNA after metabolic labeling with PSH.

To our delight, we could detect prop^5^C in poly(A)^+^ RNA from metabolically PSH-labeled HeLa cells (Fig. 2i) based on the MS/MS fragmentation (MRM transition 282.2 → 150.1) that was used for quantification (Fig. 2i) and two additional MS/MS peaks (MRM transition 282.1 → 121.9, 282.1 → 80) as qualifier (Supplementary Fig. 2, for details see Supplementary Methods). The abundance of prop^5^C was ~20-fold lower than for prop^6^A (prop^5^C/C 0.00012%) and ~100-fold lower than for A_prop_, which means that 1.4% of m^5^C was substituted by prop^5^C (Fig. 2h). This is also consistent with the ratio of abundances of the natural methylated nucleotides m^6^A, A_m_ and m^5^C^37,56,67,69,70^.

Further LC-MS analysis revealed that in addition to prop^6^A, A_prop_ and prop^5^C, we could confirm the formation of prop^1^A, prop^7^G, C_prop_, and G_prop_ in poly(A)^+^ RNA (Fig. 2i, j). However, prop^3^C and U_prop_ could not be detected in poly(A)^+^ RNA after PSH feeding. These data show that metabolic labeling via the methylation pathway leads to various nucleoside modifications that can be analyzed from the same sample. As poly(A)^+^ RNA is known to contain residual rRNA, the LC-MS measurements of poly(A)^+^ RNA do not permit conclusions regarding their presence in mRNA^56^.

To the best of our knowledge, this is the first dataset quantifying non-natural nucleosides from RNA comprehensively and provides insights into how well different MTases accept non-natural substrates. It also shows the scope of this methodology, by pointing out which modifications can be made available to NGS.

### Transcriptome-wide analysis of m^6^A from metabolic labeling with PSH

The bioorthogonal propargyl group can be reacted with azides in a copper-catalyzed azide-alkyne cycloaddition (CuAAC). In order to effectively enrich modified sites and increase steric bulk to prevent reverse transcription, we installed an affinity tag using biotin-azide (Fig. 1)^53^. Click chemistry relies on covalent bond formation and requires the terminal alkyne as a functional group. As a result, it is not affected by the interactions of nearby nucleotides, which is an advantage compared to methods relying on non-covalent interactions^71–73^. Propargylated nucleosides will therefore be universally enriched by metabolic PSH labeling and click chemistry.

For transcriptome-wide analysis, we isolated poly(A)^+^ RNA from cells after metabolic PSH labeling and performed click chemistry and enrichment (Fig. 1). The reaction with biotin-azide was almost complete (up to 96%, Supplementary Fig. 4). We first analyzed m^6^A as the most abundant modification in mRNA. To precisely assign m^6^A sites, we performed RT under conditions optimized to cause precise and strong termination (80%, Supplementary Fig. 5). We prepared libraries for NGS using an adapted iCLIP2 protocol^74^. The reads were preprocessed (FASTQ processing, barcode filtering and quality control), mapped to the human genome (hg38) and duplicate reads removed based on the introduced unique molecule identifier (UMIs). We obtained 14.8 (rep1) and 15.5 (rep2) million reads for the PSH-treated samples (see Supplementary Table 1).

Visual inspection of the coverage profiles for known and validated m^6^A sites showed remarkably sharp edges one nucleotide downstream of m^6^A sites. This is exemplified by the six m^6^A positions in the hairpins of MAT2A, the m^6^A2515 and m^6^A2577 in MALAT1 as well as m^6^A1216 in β-actin (Supplementary Fig. 4), which are known targets of METTL16 and METTL3-14, respectively^8,75,76^. These data indicate (1) that metabolic labeling results in METTL3-14 as well as METTL16-mediated propargylation at the *N*^6^-position of adenosine in poly(A)^+^ RNA and (2) that reverse transcription in isolated mRNA terminates one nucleotide downstream of clicked sites, allowing assignment of m^6^A sites in mRNA with single nucleotide precision in NGS data.

For systematic analysis of MePMe-seq data on the transcriptome-wide level, we used JACUSA2^77^. This improved version of JACUSA, a software for site-specific identification of RNA editing events from replicate sequencing data, is able to identify read termination events by calculating the arrest rate, i.e. the fraction of reads stopping at the position, from NGS data^77^. The difference between arrest rates from the sample and control (${\Delta }_{{RT\; arrest}}$) was used to filter the terminations identified by the algorithm and remove false positives. Setting a sample read coverage threshold ( > 35 for high stringency (HS) filter, >20 for low stringency (LS) filter) and arrest score threshold (${\Delta }_{{RT\; arrest}}$ > 20 for HS filter; ${\Delta }_{{RT\; arrest}}$ > 15 for LS filter), resulted in calling a total number of 8802 (rep1) and 7124 (rep2) termination sites for all four nucleotides, if high stringency settings were used (Supplementary data 1). Filtering with low stringency called more termination sites, i.e. 26,673 (rep1) and 27,869 sites (rep2), respectively (Supplementary Figs. 7, 8). For subsequent analysis, we exclusively used the results from high stringency filtering, which is most likely an underestimation of sites (Supplementary Fig. 12). JACUSA2 is available at https://github.com/dieterich-lab/JACUSA2.

**Fig. 3:**
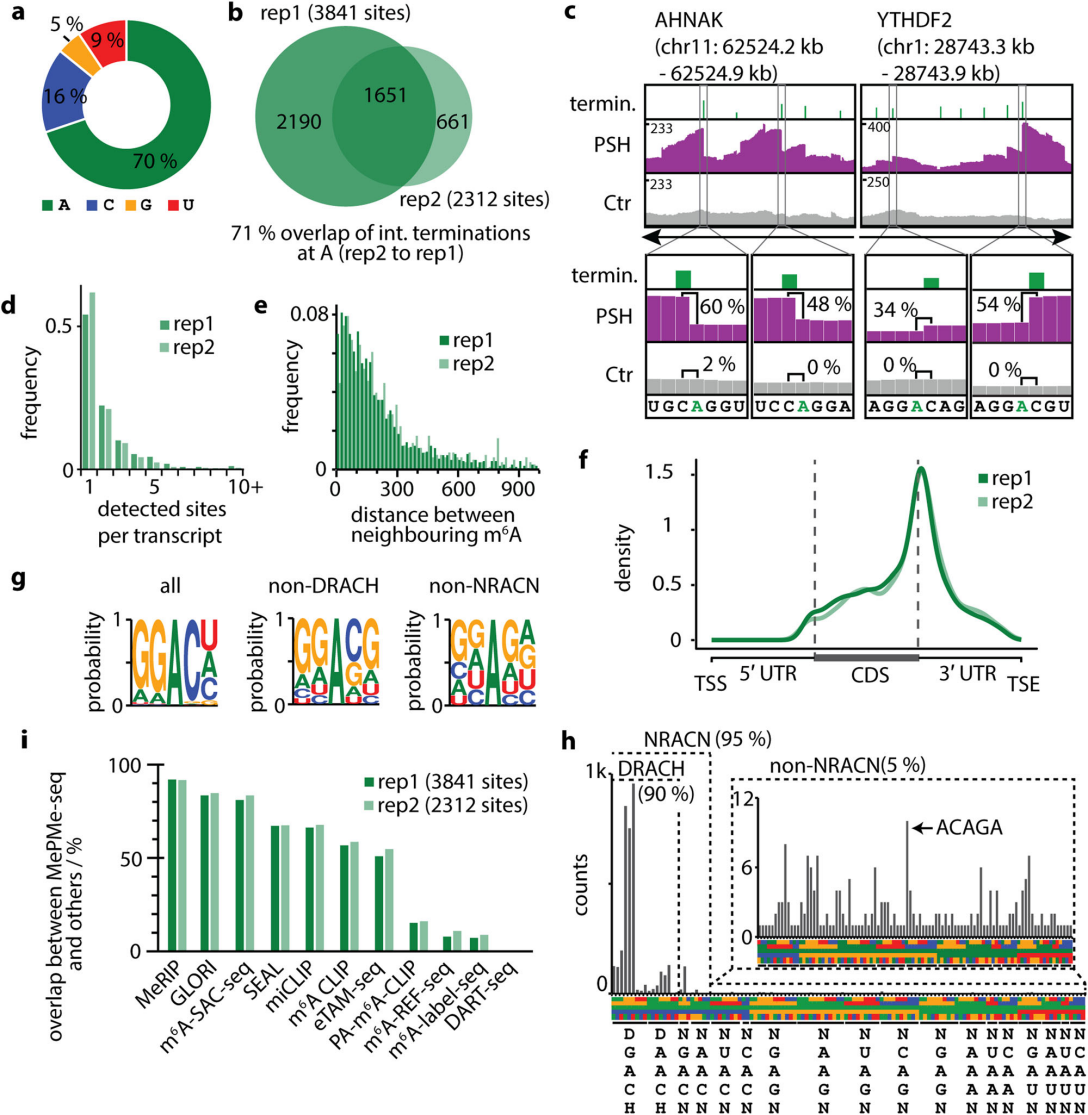
Detection of m^6^A sites using MePMe-seq. **a** Distribution of all modified nucleotides identified in MePMe-seq. **b** Overlap of m^6^A sites identified in *n* = 2 independent experiments (% calculated from rep2 to rep1). **c** Integrative genomics viewer (IGV) browser coverage tracks of MePMe-seq data for the indicated AHNAK and YTHDF2 mRNAs from cells labeled with PSH (purple) or methionine as control (gray). Green bars indicate terminations identified by JACUSA2 applying high stringency filtering. Numbers (%) for calculated arrest rate at indicated positions are shown. Arrow indicates orientation of coding strand. **d** Frequency of m^6^A sites per transcript. **e** Frequency of distance between neighboring m^6^A positions located on the same transcript. Cutoff at 1000 nt (for cutoff at 5000 nt see Supplementary Fig. 14a). **f** Metatranscript analysis showing a density plot of the distribution of prop^6^A sites detected by MePMe-seq. **g** Consensus motif for sequences surrounding identified m^6^A (HS filtering) for all 5-mers (all), if DRACH sequences are excluded (non-DRACH) or if NRACN sequences are excluded (non-NRACN). Representative example of *n* = 2 biologically independent samples is shown (2nd replicate: Supplementary Fig. 14b). **h** Sequence motifs surrounding identified m^6^A sites (HS filtering), sorted by consensus motif DRACH, NRACN or non-NRACN, respectively. Arrow indicates ACAGA-motif, which is part of METTL16 motif. **i** Overlap of all 4502 m^6^A sites identified in MePMe-seq with sites identified by MeRIP, GLORI, m^6^A-SAC-seq, SEAL, miCLIP, m^6^A CLIP, PA-m^6^A-CLIP, eTAM-seq, m^6^A-seq improved (imp.), m^6^A-REF-seq, DART-seq and m^6^A-label-seq^64,81,82,91^.

Initial inspection of these terminations revealed clustering at transcription start sites (TSS) (Supplementary Fig. 13). Accordingly, NGS coverage profiles showed strong enrichment of 5′ end fragments (i.e. ~150 nt regions) for many transcripts and steep drop-offs within this region, which were called as arrests by JACUSA2 (Supplementary Fig. 9). Based on early literature on metabolic labeling with radioactive Met^78–80^, which led to the identification of multiple methylation sites at the 5′ cap, it is reasonable to assume that metabolic PSH labeling will also target the canonical cap methylation sites, resulting in the observed clustering. To rule out effects from metabolic cap labeling, we excluded regions ≤5 nt upstream from the TSS from analysis of internal modification sites. Using this computational pipeline and high stringency filtering for data analysis, MePMe-seq identified 5506 (rep1) and 3714 (rep2) modified internal sites for all four nucleosides in mRNA from PSH labeled cells (Supplementary Fig. 7). The modified nucleotides were predominantly adenosine (A, 70%), followed by cytidine (C, 16%), uridine (U, 9%) and guanosine (G, 5%) (Fig. 3a).

In two biological replicates, MePMe-seq identified 3841 (rep1) and 2312 (rep2) internal As as MTase target sites in mRNA from HeLa cells using high stringency filtering (Fig. 3b, Supplementary Data 1). Of the modified As, 1651 (71%) were identified in both replicates, indicating very good reproducibility (Fig. 3b).

Inspection of the hits in the coverage profile showed drop-offs at the m^6^A sites identified by JACUSA2 in mRNA from labeled cells that are not observed in mRNA from control cells. This is illustrated for the AHNAK and the YTHDF2 mRNAs (Fig. 3c, Supplementary Fig. 10), which are known for their high m^6^A content^64,81,82^. The drop-offs are remarkably sharp and correspond to termination one nucleotide downstream of the modified A, in line with our thorough in vitro evaluation (Supplementary Fig. 5). Arrest rates ranged from 34–60%. These results demonstrate that MePMe-seq in combination with JACUSA2 analysis enables reliable calling of m^6^A sites with single-nucleotide precision.

Next, we looked at the abundance of m^6^A sites in individual transcripts. MePMe-seq identified methylated A in 1834 different transcripts (Supplementary Data 2) (1311 in rep2). In 54% of these transcripts, a single methylated A was found (for rep2: 61%) (Fig. 3d). In all other transcripts (i.e. 46% in rep1 and 39% in rep2, respectively), more than one methylated A was present, including some with >10 m^6^A sites (Fig. 3d, Supplementary Data 2). Some of the highest m^6^A densities were found on AHNAK, PLEC and YTHDF2 mRNAs (Fig. 3c, Supplementary Figs. 8, 10), in line with previous reports^64^. We were particularly interested in these clustered m^6^A sites that currently pose a challenge to most of the m^6^A mapping methods. MePMe-seq identified a total of 80, 25, and 12 m^6^A sites for AHNAK, PLEC, and YTHDF2, respectively (56, 18, 10, in rep2). We calculated the distance between neighboring m^6^As on the same transcript and found that they tend to cluster in short distances (Fig. 3, Supplementary Fig. 14a), emphasizing the importance of precise assignment. In summary, MePMe-seq showed remarkable precision in assigning the position of m^6^A sites and identified m^6^A sites in very close proximity (<10 nt) to each other.

We looked at the distribution of m^6^A sites by performing a metagene analysis of all modified As detected by MePMe-seq. The density plot shows enrichment at the 3′ end of the coding sequence (CDS) and around the stop codons (Fig. 3f). This result is in line with the m^6^A distribution reported by various methods, confirming that metabolic PSH labeling in combination with MePMe-seq identifies natural m^6^A sites^37,38,41–43,64,83^. The metabolic propargylation does not seem to introduce bias, except for the heavily and canonically methylated 5′ cap region which had to be excluded from analysis.

Comparing 5-mer sequences around the identified methylated internal adenosines revealed DRACH as the prevailing motif (Fig. 3g, Supplementary Fig. 14b) with an abundance of 90% (most abundant: GG**A**CU 25%, GG**A**CA 22%, GG**A**CC 20%, AG**A**CU 5%, others <5% abundance, Fig. 3h), which has been reported previously as the main consensus motif for *N*^6^-methylation of A via METTL3-14^37,38,42,43,64,83,84^. Interestingly, 10% of the m^6^A sites identified by MePMe-seq are located in non-DRACH motifs (Fig. 3h). These are composed of NR**A**CN sequences (5%), which are closely related to the DRACH motif and non-NR**A**CN motifs (5% in total) (Fig. 3h). The non-NRACN motifs do not share a consensus motif, but G is preferred over other nucleotides directly downstream of A (Fig. 3g). Within the non-NR**A**CN hits, the sequence AC**A**GA is most abundant (Fig. 3h). This sequence is part of the motif targeted by METTL16^85^. Of note, MePMe-seq identified all currently known methylation sites of METTL16, i.e. six sites in the 3ʹ-UTR of MAT2A-mRNA, as well as the U6 snRNA (Supplementary Figs. 11, 15). These non-DRACH sites escape antibody-based approaches and approaches relying on bioinformatics searches for the DRACH motif, like m^6^A-CLIP^41^. MePMe-seq is thus able to accurately detect m^6^A in non-DRACH contexts and provide data about the interconnectivity of different methylations in an unbiased manner^81,82^.

### Overlap with datasets from other m^6^A-mapping methods

m^6^A sites have been mapped previously using antibody-dependent and antibody-independent methods^37–39,41–43,64,83,86–91^. To compare MePMe-seq results with m^6^A sites found in previous studies, we assembled data from the databases REPIC^82^, ATLAS^81^ and publications comprising various methodologies^64,91^. However, in a pairwise comparison of published datasets from individual miCLIP experiments, the detected m^6^A sites differed significantly, even for the same cell line (Supplementary Table 2). We therefore combined the hits reported in different experiments to obtain an unbiased and more comprehensive reference dataset^88^.

We found that 92% of the m^6^A sites identified by MePMe-seq matched the reported MeRIP hits (Fig. 3i). 81–85% of the MePMe-seq sites overlapped with GLORI and m^6^A-SAC-seq, 51–67% with m^6^A sites identified using SEAL, miCLIP, m^6^A CLIP or m^6^A-SAC-seq. A lower fraction (8–15%) of the MePMe-seq sites were found in other antibody-free single nucleotide resolution techniques, i.e. PA-m^6^A-seq, m^6^A-REF-seq, m^6^A-label-seq (Fig. 3i). Only 18 sites (0.4%) of m^6^A sites identified by MePMe-seq were also reported in DART-seq. This fraction increases when the exact sites are extended: 5% of m^6^A sites identified in MePMe-seq are in close proximity (±50 nt) to sites identified in DART-seq and the overlap between the techniques increases up to ~11% if an uncertainty range of ±150 nt is allowed (Supplementary Fig. 14c). Of note, the overlap of m^6^A sites detected by MePMe-seq is higher than the range obtained by comparison of other single nucleotide resolution methods with CLIP and better than comparison between each other (Supplementary Table 3, Supplementary data 3), suggesting that m^6^A sites reported by MePMe-seq are highly reliable.

### Independent validation of m^6^A sites identified by MePMe-seq

**Fig. 4:**
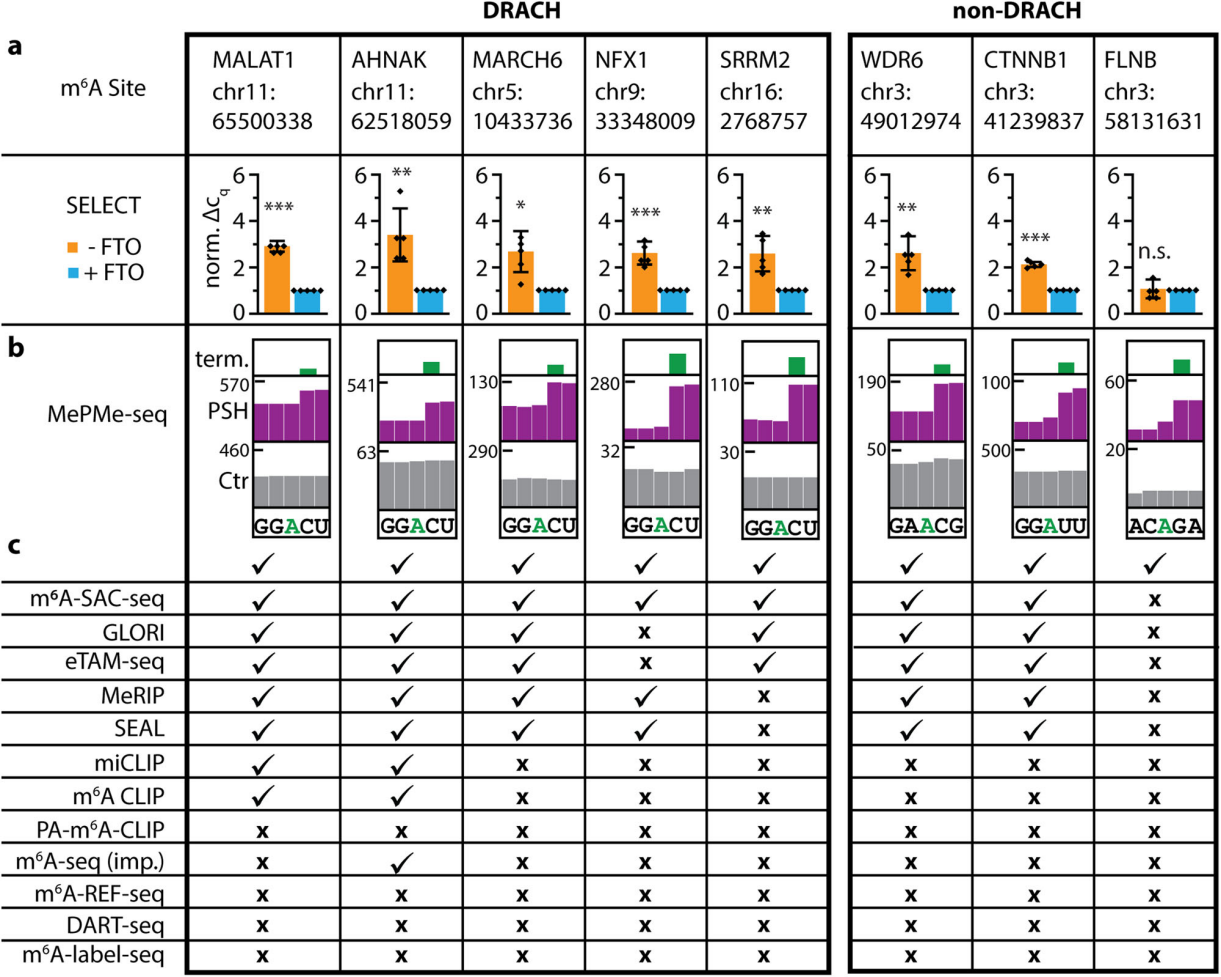
Validation of m^6^A sites identified in MePMe-seq via SELECT in HeLa poly(A)^+^ RNA. **a** The normalized ΔC_q_ values of SELECT qPCR measurements are shown for five sites located in a DRACH motif and three sites located in a non-DRACH motif. Mean values and SD from *n* = 5 biological replicates are shown. Statistical significance determined via one-sample one-tailed *t* test (n.s. *P* > 0.05; *P ≤ 0.05; **P ≤ 0.01; ***P ≤ 0.001). The *P* values for – FTO versus + FTO samples are for MALAT1 2.6 × 10^−5^, for AHNAK 4.8 × 10^−3^, for MARCH6 6.9 × 10^−3^, for NFX1 9.8 × 10^−4^, for SRRM2 5.0 × 10^−3^, for WDR6 4.1 × 10^−3^, for CTNNB1 1.1 × 10^−5^ and for FLNB 0.39. **b** IGV browser coverage tracks of MePMe-seq data for the same sites from cells grown with PSH (purple) or methionine (gray) as control. Green bars indicate terminations identified by JACUSA2. **c** Comparison with m^6^A-SAC-seq, GLORI, eTAM-seq, MeRIP, SEAL, miCLIP, m^6^A CLIP, PA-m^6^A-CLIP, improved (imp.) m^6^A-seq, m^6^A-REF-seq, DART-seq or m^6^A-label-seq sequencing datasets^64,81,82,91^. Checkmark for sites present, x for sites not present in dataset (data obtained from literature). Source data are provided as a Source Data file.

To independently validate m^6^A sites identified by MePMe-seq, we performed SELECT, an elongation- and ligation-based qPCR amplification method with single-nucleotide resolution^92^. We evaluated eight putative m^6^A sites in poly(A)^+^ RNA, five of them with a DRACH motif and three with a non-DRACH motif (Fig. 4a). Comparing the normalized Δ*C*_q_ values of qPCRs of samples with and without FTO treatment, a Δ*C*_q_ > 1 for —FTO indicated the presence of m^6^A. We found that all five chosen DRACH sites indeed contained m^6^A (Fig. 4a). This includes an m^6^A site in the mRNA coding for the serine/arginine repetitive matrix protein 2 (SRRM2) that escaped many methods and was only recently reported^49,51,93^ (Fig. 4c). Of the three tested non-DRACH sites, WDR6 and CTNNB1 mRNAs were confirmed to contain m^6^A. These sites have been reported before via MeRIP and SEAL and only recently with the single-base resolution techniques m^6^A-SAC-seq, GLORI and eTAM-seq (Fig. 4c). The putative non-DRACH m^6^A site in FLNB, however, could not be validated by SELECT (Fig. 4a, b). As FTO has sequential and structural preferences^71^ it is conceivable that this m^6^A site in the ACAGA sequence is not a good substrate for FTO and therefore not detectable via SELECT. To test this hypothesis, we tried to validate a known non-DRACH m^6^A site located in the same sequence motif in the 3′ UTR of MAT2A forming a hairpin structure. Indeed, SELECT failed to detect this well-known non-DRACH m^6^A site, most likely because of its hairpin structure and lack of FTO-mediated demethylation (Supplementary Fig. 16).

In summary, all of the five tested m^6^A sites in a DRACH motif were confirmed by SELECT. In addition, two of the three putative m^6^A sites detected by MePMe-seq (Fig. 4b) in non-DRACH sites were confirmed by SELECT, indicating that MePMe-seq is one of the first methods with single nucleotide resolution able to detect m^6^A-sites in non-NRACN motifs. Since SELECT relies on FTO, bias originating from the enzyme’s substrate preference has to be considered. Therefore, it is conceivable that non-DRACH sites reported by MePMe-seq are true sites, even if confirmation by SELECT is not possible.

### Identification of METTL16-specific target sites by combined in vitro and metabolic modification

MePMe-seq relies on intracellular formation of the SAM analogue SeAdoYn and therefore detects m^6^A sites originating from different MTases. The in vitro modification with a specific MTase, on the other hand, bears potential to modify exactly the target sites of this particular MTase. With this direct approach, modifications can be assigned to a specific MTase, provided that the target sites are not fully modified in cellular RNA. In mRNA, modifications are often sub-stoichiometric, allowing for subsequent in vitro modification. While most m^6^A sites are METTL3-14 dependent, METTL16 is an emerging player in the RNA modification landscape of the human cell^94^. METTL16 has been shown to bind a number of RNAs, including mRNAs and lncRNAs^9,94^, however methylation was only confirmed for six sites in MAT2A mRNA and U6 snRNA^8^.

**Fig. 5:**
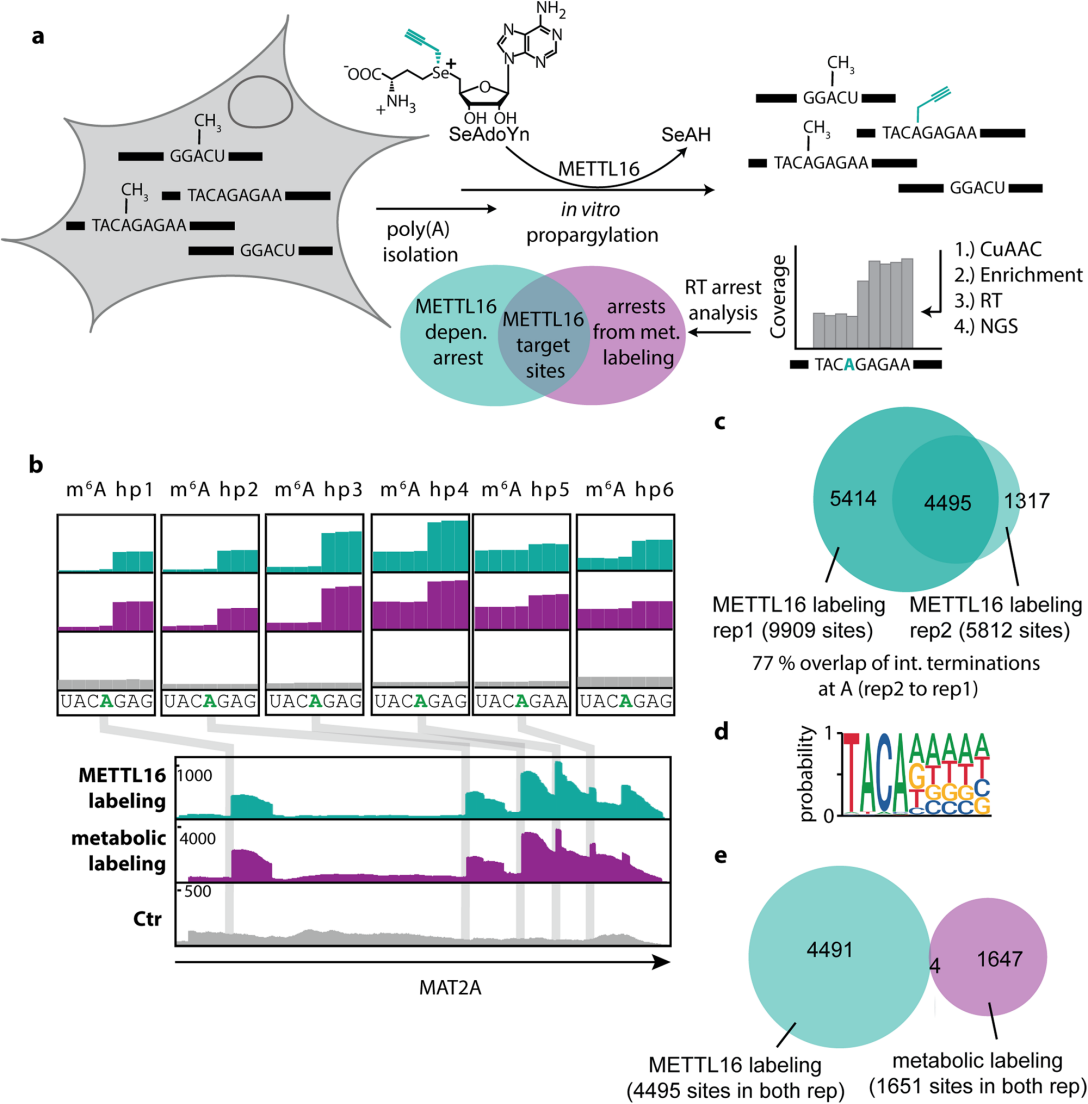
METTL16-dependent propargylation. **a** Scheme illustrating METTL16-dependent labeling in combination with MePMe-seq to identify METTL16 target sites. Isolated mRNA is propargylated in vitro using METTL16 and analyzed by NGS. To eliminate false positive hits from in vitro off-target effects of METTL16, only hits observed also in MePMe-seq are identified as METTL16 targets. **b** IGV browser coverage tracks for MAT2A-mRNA mapped by METT16-labeling in vitro (cyan) or MePMe-seq (purple), or control (gray). Arrow indicates orientation of coding strand. **c** Overlap of identified m^6^A sites in *n* = 2 independent METTL16-labeling experiments. **d** Consensus motif for sequences surrounding identified As after METTL16-dependent labeling in vitro. **e** Overlap of identified m^6^A sites in METTL16-dependent in vitro and metabolic labeling for sites present in *n* = 2 independent experiments with HS filtering.

To pinpoint METTL16-dependent m^6^A sites, we isolated poly(A)^+^ RNA from untreated HeLa cells and propargylated it in vitro using recombinantly produced METTL16 and SeAdoYn (Fig. 5a, Supplementary Fig. 17). The in vitro propargylated mRNA was then processed as described above to enrich biotinylated RNA and determine the modification sites via termination in NGS. Visual inspection of the few known METTL16 target sites revealed sharp edges in the coverage profile precisely one nucleotide upstream of the targeted adenosine in all cases, i.e. the hairpins in the 3′ UTR of the MAT2A-mRNA and the U6 snRNA (Fig. 5b, Supplementary Fig. 15, Supplementary data 4). These drops were exclusively found in the modified sample but not in a control sample and matched sites found by metabolic labeling, confirming that these are METTL16-dependent target sites that are also installed in intact cells.

Sequencing and evaluation yielded 9909 and 5812 putative METTL16 target sites in two independent replicates (Fig. 5c, Supplementary data 5). Of these sites, 4495 were found in both replicates (i.e. 77% overlap), indicating good reproducibility. Within these hits, we inspected previously reported interaction sites of METTL16, such as STUB1, RBM3, MYC, NT5DC2, GNPTG, GMIP and MALAT1^9,95^, for which it is unclear, whether they are also methylated by METTL16. Interestingly, we detected several of these sites in both replicates for MYC, RBM3, NT5DC2 and MALAT1 (Supplementary Fig. 19, Supplementary Table 4), providing evidence that METTL16 is indeed able to modify them in vitro. We observed multiple METTL16-dependent sites in the cancer-associated MALAT1 lncRNA, however, A8290 was not methylated in vitro (Supplementary Fig. 20b). This is of particular interest, as A8290 was shown to interact with METTL16 but could not be validated as methylation target^94,96^. Analysis of the sequence motif adjacent to the m^6^A sites resulting from in vitro METTL16 labeling, identified a TACAD (Fig. 5d) motif, containing the reported METTL16 consensus motif TACA motif^85^.

The large number of METTL16 sites identified by in vitro labeling is in stark contrast to the small number of confirmed sites. In vitro modification of RNA has also been used in other methods^97^, however, we wondered whether the non-natural conditions could lead to off-target modification by METTL16 in vitro. To unambiguously identify METTL16 sites, we therefore matched the data from in vitro METTL16 labeling with the data from metabolic labeling. Hits identified in both approaches should be relevant METTL16 sites in cells. For this comparison, we used sites appearing in both replicates of in vitro METTL16 labeling (4495 hits) and MePMe-seq (1651 hits) and identified only four overlapping sites as hits (Fig. 5e). This indeed suggests that a large fraction of hits from in vitro METTL16 labeling result from off-target effects. Analysis of the four hits showed that these are the previously reported METTL16 target sites in the 3′-UTR of MAT2A^85^. Two of the reported METTL16 hits in mRNA escaped this assignment using the HS filtering conditions but were detected in either rep1 or rep2 of MePMe-seq (Supplementary data 3).

When we inspected A8290 from MALAT1, which has been previously suspected to be a METTL16 target site, we found that this site was neither called by METTL16 in vitro labeling nor by metabolic labeling (Supplementary Fig. 20b, Supplementary Table 4)^98^. However, several m^6^A sites in close proximity to the putative METTL16 target site in MALAT1 could be clearly assigned owing to the high precision by MePMe-seq. Based on the combined analysis of in vitro and metabolic labeling we can now exclude A8290 in MALAT1 as a target of METTL16.

In summary, the in vitro modification data of METTL16 show that real and off-targets are detected within the consensus motif TACAD when applied in vitro at high concentrations. As several of the METTL16-dependent in vitro sites coincide with the interaction sites identified by CRAC, it could mean that METTL16 binds and—with SeAdoYn—can modify them. It cannot be excluded that additional proteins/RNAs as cofactors facilitate METTL16-dependent methylation in cells. We could show that the combination of in vitro and metabolic labeling provides a reliable protocol to assign the m^6^A sites to a certain MTase and determine its target sites with single nucleotide precision.

### m^6^A, A_m_ and m^1^A can be distinguished by termination signatures

**Fig. 6:**
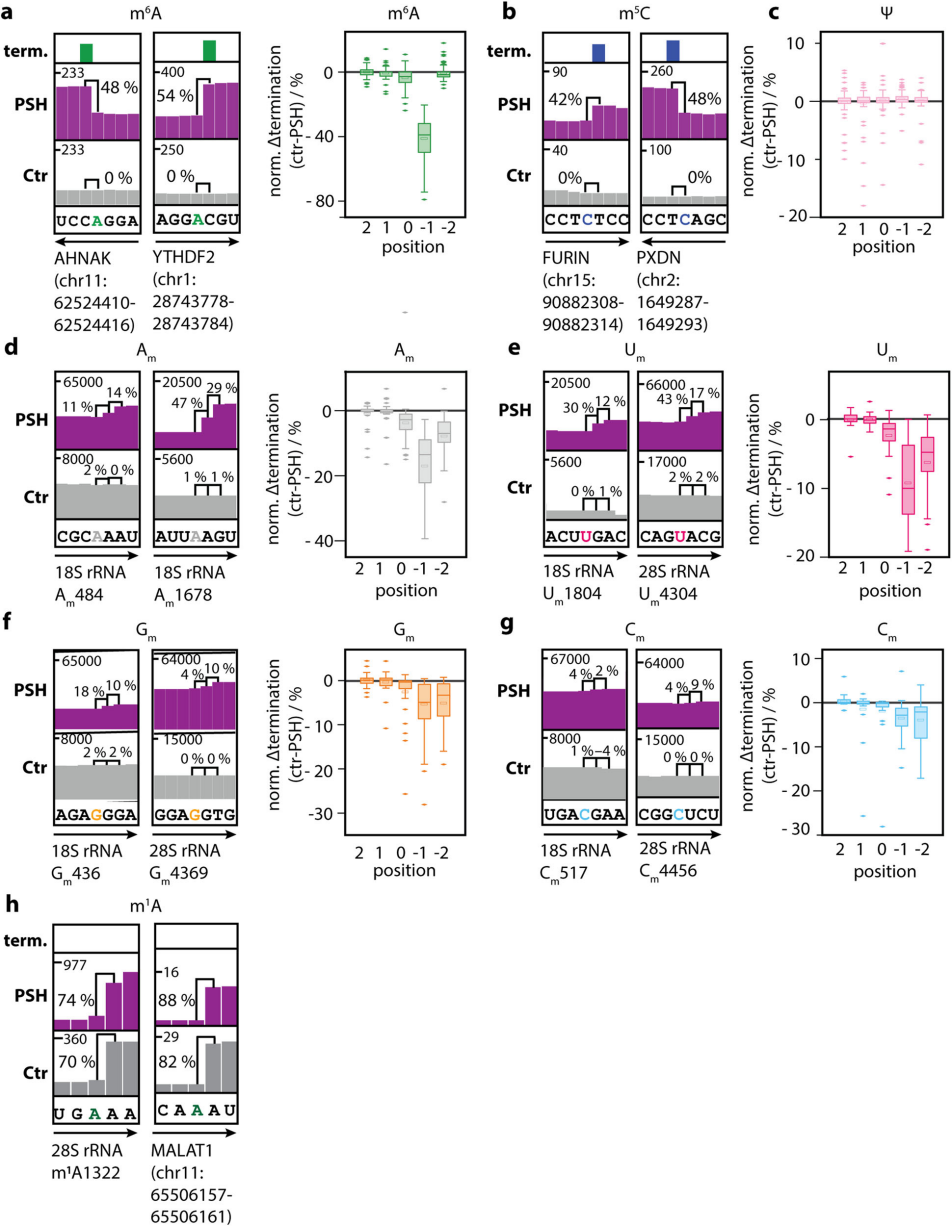
Termination signatures at different modified nucleosides after metabolic labeling. Two representative IGV coverage tracks of MePMe-seq data are shown for RNA modifications (**a**) m^6^A, (**b**) m^5^C, (**d**) A_m_, (**e**) U_m_, (**f**) G_m_, (**g**) C_m_ and (**h**) m^1^A. poly(A)^+^ RNA from HeLa cells labeled with PSH (purple) or methionine as control (gray) was used. Terminations identified by JACUSA2 are indicated by a additional brackets. Numbers indicate reads and the calculated arrest rate (%) in that position. Arrow indicates orientation of coding strand. Box plots show bioinformatic analysis of termination signatures based on the coverage (Diff ctr-PSH) at the positions –2, –1, 0, 1, 2 of frequently identified modification sites of (**a**) m^6^A in mRNA, (**c**) Ψ, (**d**) A_m_, (**e**) U_m_, (**f**) G_m_ and (**g**) C_m_ (N_m_ in rRNA). In the boxplots the center lines, medians, upper and lower quartiles, whiskers (1.5×) and outliers are shown. Source data are provided as a Source Data file.

In order to efficiently enrich MTase target sites and cause termination, we used propargylation and click chemistry. This enables transcriptome-wide identification of m^6^A sites. However, termination can also be brought on by A_m_ and m^1^A. We therefore checked whether MePMe-seq hits for m^6^A (Fig. 6) still contained various modifications. We examined the termination signatures of prop^6^A and A_prop_ in vitro to see if our technique can distinguish between m^6^A and A_m_ sites (Supplementary Figs. 5, 6). Indeed, the termination at prop^6^A and A_prop_ results in distinct patterns, suggesting that these modified nucleosides can be distinguished.

The next step was to analyze our MePMe-seq data to separate m^6^A sites from A_m_ sites on a transcriptome-wide scale. We thoroughly examined termination signals near known A_m_ sites in rRNA making use of residual rRNA in our poly(A)^+^ RNA samples (Fig. 6). We discovered a stepwise termination pattern at positions –1 and –2 obvious in the IGV coverage tracks, as indicated for two known A_m_ sites in the 18S rRNA (Fig. 6). Similar to this, we employed a test set of well-known and frequently identified m^6^A sites (identified in ≥7 independent studies and MePMe-seq) in mRNA to identify the m^6^A termination signature. We verified the strong and nearly exclusive termination at position –1 for m^6^A sites (Fig. 6). The stepwise versus precise termination signatures of A_m_ compared to m^6^A sites obtained from transcriptome-wide analysis are consistent with the in vitro data (Supplementary Figs. 5, 6).

JACUSA2 would classify both, the A_m_ and m^6^A sites, as hits (because of the –1 termination), but with the more accurate analysis, we can attribute the stepwise termination to A_m_. We conducted a cluster analysis of the termination signals to identify probable A_m_ sites in mRNA in our MePMe-seq data. Two groups of termination signatures were found. Cluster 1 displayed the precise termination at –1 typical of m^6^A sites, while cluster 2 displayed a distinct pattern that did not correspond to the termination signature seen for A_m_ sites (Supplementary Fig. 22). Most of the modified As discovered in mRNA by MePMe-seq do in fact originate from m^6^A sites, as evidenced by cluster 1’s dominance and presence of >98% of sites in both replicates (Supplementary Figs. 22, 23). Also the putative m^6^A site in FLNB RNA was identified as member of cluster 1. Nearly all of the remaining <2% of cluster 2 sites are situated in close proximity to a TSS (≤5 nt) and were removed during filtering in our MePMe-seq-analysis. The remaining 5 sites (less than 0.2%) could be false positives brought on by alternative TSS. According to the additional pattern analysis MePMe-seq identifies m^6^A sites in mRNA and we do not have evidence for A_m_ sites in mRNA.

Next, we asked whether MePMe-seq would also identify m^1^A sites. However, position *N*1 is in the Watson-Crick side and its methylation impedes polymerases^99,100^. Inspecting known m^1^A sites in rRNA, such as the conserved m^1^A1322 in human 28S rRNA^101,102^, confirmed that the IGV coverage tracks for methionine-fed controls exhibit a strong termination at m^1^A sites (Fig. 6h). RNA from PSH-labeled cells likewise exhibits this termination. As a result, JACUSA2—which examines the difference in arrest rate between PSH-cells and controls—does not classify these sites as hits. Therefore, m^1^A sites are absent from MePMe-seq hits for modified adenosines.

The reports on m^1^A mapping remain controversial and reveal the limitations of antibody-based approaches^68^. Although JACUSA2 will not identify m^1^A sites as hits on a transcriptome-wide level, we can individually validate putative m^1^A sites by inspecting the IGV coverage tracks. We examined a putative m^1^A site in MALAT1^103^, and 9 internal m^1^A sites in cytosolic mRNA as well as 12 mitochondrial RNAs from previous reports^103,104^. The m^1^A sites in MALAT1 and one in the mitochondrial 16S rRNA were verified by visual inspection (Fig. 6h). However, none of the reported m^1^A sites in mRNA and the additional mitochondrial RNAs had the PSH- and control-specific termination signature that would be anticipated for m^1^A sites (Supplementary Fig. 24). These findings indicate that the majority of the m^1^A sites previously identified are not detectable by termination, which may be because they do not exist or are modified at a very low stoichiometry. As the sole technique not reliant on antibodies, MePMe-seq can help validate probable m^1^A locations. Importantly, MePMe-seq will not identify false positive m^6^A hits that originate from m^1^A.

### Analysis of additional methylation sites

The examination of termination signatures was then expanded to include all 2′-*O*-methylated nucleosides from rRNA (Fig. 6). A stepwise termination pattern was visible at all N_m_ sites. U_m_ sites were extremely noticeable, much like A_m_ sites, whereas G_m_ and C_m_ sites resulted in weaker termination. As control, we analyzed pseudouridine sites in the same rRNAs (Fig. 6c). These caused no discernable profile for the median of termination difference between PSH treated sample and control, confirming that the termination signatures for N_m_ sites are associated with ribose propargylation. The termination signatures were independently confirmed via primer extension assays, using short RNAs with the corresponding modification at one specific position (Supplementary Fig. 6).

As additional control, we compared our results to a previous report on N_m_ in human mRNA from NGS data at single nucleotide resolution^65^. We discovered five sites that matched (Supplementary Table 7). On closer investigation, it was discovered that they were situated in two rDNA gene family members, indicating that they are misaligned rRNA fragments rather than mRNA (Supplementary Table 7). All things considered, we do not have evidence that MePMe-seq found internal N_m_ sites in mRNA.

### MePMe-seq identifies m^5^C sites in mRNA

**Fig. 7:**
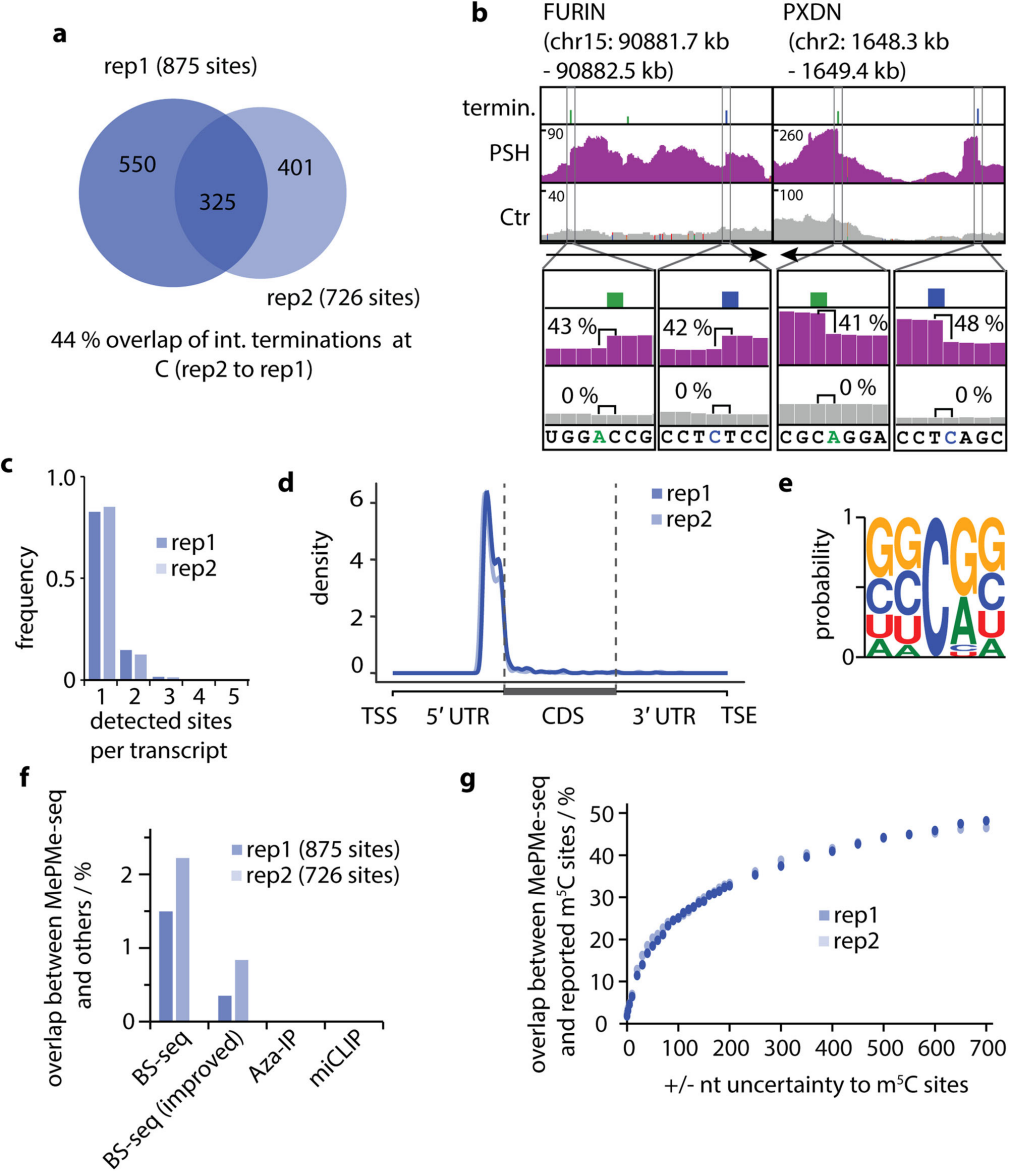
Detection of m^5^C sites using MePMe-seq. **a** Overlap of identified m^5^C sites between *n* = 2 MePMe-seq experiments (44% calculated from rep2 to rep1). **b** IGV browser coverage tracks of MePMe-seq data for the indicated FURIN and PXDN mRNAs from cells labeled with PSH (purple) or methionine as control (gray). Blue and green bars indicate terminations at C and A, respectively, identified by JACUSA2. Numbers (%) denote the calculated arrest rate in that position. Arrow indicates orientation of coding strand. **c** Frequency of m^5^C sites per transcript and replicate. **d** Metatranscript analysis showing a density plot of the distribution of m^5^C sites detected by MePMe-seq. **e** Consensus motif for sequences surrounding identified m^5^C sites. Representative example of *n* = 2 biologically independent samples. **f** Overlap of m^5^C identified in MePMe-seq per replicate (combined sites from *n* = 2 independent experiments with HS filtering) with sites identified by bisulfite sequencing (BS-seq), improved BS-seq, Aza-IP and miCLIP^81^. **g** Overlap of m^5^C sites identified in MePMe-seq (combined sites from *n* = 2 independent experiments) with all m^5^C sites from ATLAS database^81^, when increasing uncertainty region around the site is applied.

Next, we examined the second-most prevalent termination in our sample—cytidines. 16% of terminations one nucleotide upstream of cytidines were found by MePMe-seq (Fig. 3a). A total number of 1276 sites were discovered. In more detail, 875 sites were discovered in rep1, 726 sites in rep2, and 325 sites (44%) in both replicates (Fig. 7a). These findings imply that metabolic labeling causes cytidines in mRNA to become propargylated, which can then be found using MePMe-seq.

In our LC-QqQ-MS analysis of poly(A)^+^ RNA, we detected C_prop_ and prop^5^C, but no prop^3^C (Fig. 2). The termination analysis of prop^5^C and C_prop_ in vitro showed that propargylation at either position results in termination of reverse transcription after click chemistry and binding to streptavidin-beads (Supplementary Fig. 6).

Visual inspection of the sequencing data for the frequently reported m^5^C-containing FURIN- and PXDN-mRNAs shows sharp drop-offs not only for m^6^A sites but also for putative m^5^C sites (Fig. 7b). Furthermore, these mRNAs appear enriched compared to the control samples that had not been treated with PSH, supporting installation of propargyl groups as handles for click chemistry and biotin-based isolation of modified sites (Fig. 7b). Terminations downstream of cytosine occurred in most cases only once per transcript (Fig. 7c) and were located mainly at the end of the 5′ UTR (Fig. 7d). The frequency and distribution for putative m^5^C sites are thus markedly different from m^6^A (Fig. 3f), in line with previous reports about m^5^C^32,69,105,106^. Analysis of the sequence context did not reveal a clear consensus motif (Fig. 7e, Supplementary Fig. 25a). This result is in accordance with literature for type I m^5^C sites^107–109^. The known motif for type II m^5^C sites (**C**UCCA) can be found in the results but is not prevalent, in line with their low abundance (Supplementary Fig. 25b)^108^.

We wanted to find out whether the modified Cs identified by JACUSA2 are indeed m^5^C sites or contain C_m_ sites. However, confirmed m^5^C sites in rRNA and mRNA are rare, preventing reliable analysis of termination signatures. As a control, we therefore compared our transcriptome-wide termination data to previously published m^5^C and C_m_ sites^32,34,69,106,107^. We have 22 overlapping sites with the recent report by Yang et al., including the above mentioned FURIN- and PXDN-mRNAs, and additional overlaps with other publications (Supplementary Table 5, Fig. 7f)^32^. For m^5^C we found terminations at reported sites in mRNA, but not in rRNA. For N_m_ sites we only found terminations at reported N_m_ sites in rRNA, but not in mRNA. These data indicate that MePMe-seq detects 5-methylated rather than 2′-*O*-methylated Cs.

Similar to sequencing data for m^6^A, reported m^5^C sites vary strongly between different reports as can be seen in a pairwise comparison of results from different base resolution techniques (Supplementary Tables 5, 6), retrieved from ATLAS database^81^. The overlap of m^5^C sites identified by MePMe-seq with reported sites is also very low. However, if an uncertainty window of ±50 nt is allowed, 20% of the identified sites overlap with sites from previous studies. The imperfections of different m^5^C mapping methods are a well-known problem in the field, demanding for m^5^C mapping methods independent of bisulfite treatment^29,106,110^. Of note, the m^5^C sites overlapping between MePMe-seq and individual bisulfite experiments were reported in multiple experiments. For example, m^5^C in NECTIN2, FURIN, TRAF7, PXDN and THOC7 was present in 6–17 experiments (according to the ATLAS database^81^), thus independently confirming their existence.

Taken together, our data indicate that termination is induced at prop^5^C and C_prop_ after click chemistry. Comparison with previous datasets indicates that we detect prop^5^C sites in mRNA representing natural m^5^C sites. Similar to A_m_, we have no evidence for C_m_ sites in MePMe-seq data from poly(A)^+^ RNA.

## Discussion

We demonstrated that metabolic labeling with PSH produces a broad scope of propargylated nucleotides that are amenable to enrichment from cellular RNA. Different methylation sites can then be detected in mRNA with single nucleotide precision^106,111,112^. The bioorthogonal propargyl group replaces 1.5–3.5% of methyl groups in m^6^A and m^5^C, respectively and provides two key features, (i) it enables enrichment of the modified sites via click reaction with biotin and (ii) it induces termination when reverse transcription is performed on beads. MePMe-seq proved powerful for the precise assignment of m^6^A sites in mRNA even if multiple sites were in closer proximity. Importantly, it is completely independent of antibodies and sequence-searches, such as the DRACH- or NRACN-motif that is used in many cases. MePMe-seq is therefore able to find non-NRACN motifs, such as METTL16 sites and sites that escape many methods. The combined analysis of metabolic labeling and in vitro labeling proves suitable to assign m^6^A sites as target sites to a specific MTase in cells, as demonstrated for METTL16. The refined analysis of termination signatures allowed to distinguish m^6^A from A_m_ and m^1^A sites. The m^5^C sites vary depending on the method used, however, our data confirm repeatedly reported sites and identify additional sites.

MePMe-seq is, to our knowledge, the first method enabling simultaneous mapping of m^6^A and m^5^C in poly(A)^+^ RNA. According to LC-QqQ-MS analysis, N_m_ is also present in these samples. We found termination at m^6^A, m^5^C and N_m_ sites, but while we observed one remarkably sharp edge in the coverage profile for m^6^A and m^5^C sites one nucleotide downstream of the modification site, for 2′-*O*-modified sites it resulted in a stepwise termination. Additionally, the promiscuity of different MTases regarding their acceptance of SeAdoYn as co-substrate seems to vary. N_m_ sites were only detected on rRNA and m^6^A and m^5^C sites in rRNA that are usually used to benchmark a method did not show signals in MePMe-seq. As a result, MePMe-seq provides no evidence for a detection of A_m_ sites in mRNA, suggesting that detected N_m_ originates from residual rRNA. m^1^A sites are not called by JACUSA2 because the methylation itself already increases termination.

Cellular labeling via SAM analogues as central metabolic hub will be important for future studies about the interconnectivity of the different modifications. We showed this for m^6^A and m^5^C but it is conceivable that further improvements might allow to get insights into methylated uridines and guanines.

## Methods

### Experimental procedures

#### Chemical synthesis and characterization

PSH and SeAdoYn were synthesized as previously described^53^. Synthesis and characterization of prop^5^C, prop^1^A, prop^3^C, prop^7^G and 5-propargylcytidine-5′-triphosphate are described in the Supplementary Methods.

#### Details about LC-QqQ-MS machine and software setup

The LC-QqQ-MS system we used herein is an Agilent 1260 Infinity II LC-system coupled with and an Agilent Ultivo Triple Quadrupole mass spectrometer equipped with an advanced electrospray ionization (ESI) source (JetStream). Chromatographic separation was performed on an Agilent Poroshell 120 EC-C18 3 × 150 mm 2.7 μm column with complementary pre-column Agilent EC-C18 3 × 5 mm 1.9 µm.

The system was controlled by the MassHunter Data Acquisition Software (edition for Ultivo, version C.01.00). For basic analysis the MassHunter Qualitative Analysis Navigator (version B.08.00) was used and for quantification the MassHunter Quantitative Analysis Software (edition for QqQ, version B.09.00) was used. For optimization of MRM parameters, the manual methods developing and the MassHunter Optimizer (version C.01.00) were used complementary.

MS data were conducted via ESI in positive ion mode and multiple reaction monitoring (MRM) was employed for the detection and quantification. The ion source parameters used herein include: Main gas temperature = 250 °C, Sheath gas temperature = 375 °C and Capillary Voltage = 2400 V. The mass spectrometr Ultivo has the Cell Acceleration Voltage (CAV) hard set at 9 V.

In the method presented herein, the dynamic MRM (dMRM) mode was used. In dMRM mode, the number of concurrently measured transitions is minimized by using a defined time window for each analyte. Moreover, dMRM mode is able to prevent potential problems with partially overlapping analytes^113^.

#### Details to prop^5^C quantification via LC-QqQ-MS

The quantification process of prop^5^C was more difficult than for other analytes. For this reason, details are provided here. The solution of the prop^5^C standard for preparing a calibration curve had concentration 4.9 nM (1.39 pg/µL). The calibration curve (Supplementary Fig. 29) was made up from 7 points. Dilution pattern was the same for all analytes: 1:2:2.5:2:2:2.5:2, meaning 0.1, 0.2, 0.5, 1, 2, 5, 10 µL were injected.

For all points of the calibration curve of prop^5^C, the quantifier peaks had very good shape and quality (Supplementary Fig. 27). But because both qualifiers had low abundance (the signal of them is much lower in comparison with quantifier) (Supplementary Fig. 26), the qualifier peaks collapse between points 3 (cc3) and 2 (cc2) of the calibration curve (Supplementary Fig. 28). Already point 3 is not very strong. Even though the selection of qualifiers and mass spectrometric parameters optimization of qualifiers were managed carefully, the signal of them crossed the detection limit of the system. On the other hand, the quantifier signal is very good. Thus, for the quantification process itself (which is based on quantifier) the setup is sufficient. Considering the above, we decided to use the analytical data of prop^5^C standard and perform the measuring and quantification of the real samples.

#### In vitro transcription

*N*^6^-Propargyl adenosine-containing RNAs were produced by in vitro transcription using T7 RNA polymerase (RNAP). T7 primer was prehybridized with T7 template (10 μM each) before transcription was performed in 1× CARO buffer (120 mM Tris, 6 mM Spermidine, 0.03% (v/v) Triton-X, 4.5% (w/v) PEG6000, 15 mM DTT, pH 8.1), 15 mM Mg(OAc)_2_, 3.3 mM per NTP (UTP, GTP and CTP), 0.4 mM ATP (or modified ATP), 1 mg/mL T7 RNAP and 0.1 U PPase for 4 h at 37 °C. The template DNA was digested using 5 U/μL DNase I. The resulting test RNA was purified via PAGE (15% denat. PAA gel, 1× TBE), the desired band was cut out, RNA was eluted and precipitated. For biotinylated RNA, in vitro transcription with *N*^6^-propargyl-ATP and PAGE purification were followed by CuAAC (150 mM biotin-N_3_, 60 mM sodium phosphate buffer, 25 mM THPTA, 5 mM CuSO_4_ (THPTA and CuSO_4_) were pre-mixed) 100 mM sodium ascorbate) and an additional PAGE purification.

#### Primer extension assays for different modifications

RT primer 1/3 (20 μM) was radioactively labeled with [γ-^32^P]-ATP using 0.4 U/μL T4 PNK (30 min, 37 °C). Excess [γ-^32^P]-ATP was removed by MicroSpin G-25 columns. The propargylated test RNA was modified with biotin-N_3_ and purified as described before. The purified biotinylated test RNA was enriched via streptavidin coated magnetic beads. For this, 5 µL RNase free M-280 Streptavidine Dynabeads were saturated by adding oligo(dT) (0.2 µL, 100 µM, 10 min). Afterwards, the biotinylated test RNA (2.5 µL, 10 µM) was added and incubated for 1 h at rt and constant rotation. The beads were washed with the binding & washing buffer recommended by the manufacturer 5 × 1 mL and 1 × 1 mL ddH_2_O. The bead-bound RNA was resuspended in ddH_2_O. The test RNA (0.25 µM) with or without propargylation was hybridized with 2 µM [^32^P]-labeled RT-primer (5 min, 65 °C followed by 5 min, 0 °C) and incubated with 0.1 U/µL reverse transcriptase SSIV and 0.1 mM dNTP for 30 min at 50 °C. The reaction was stopped by addition of 0.1 M HClO_4_. The RNA was digested via alkaline hydrolysis by addition of 185 mM NaOH (80 °C, 10 min), neutralized with HCl and analyzed via PAGE. Termination bands were quantified using ImageJ and normalized to the total amount of primer extension products.

#### Determination of clicking efficiency

20 ng/µL test RNA were mixed with 150 mM biotin-N_3_ and 60 mM phosphate buffer. Then, 25 mM THPTA and 5 mM CuSO_4_ (pre-mixed) were added, the reaction was started by adding 100 mM NaAsc and incubated for 30 min at 37 °C. The reaction was stopped by adding 10 mM EDTA. The RNA was precipitated in EtOH, suspended in ddH_2_O and digested to nucleosides, using P1 nuclease and FastAP. For this, the RNA (140 ng/µL) was digested to nucleotides using 5 mU/µL P1 nuclease and 1× P1 nuclease buffer (1 h, 50 °C) and dephosphorylated to nucleosides by adding 0.05 U/µL FastAP and incubating for 1 h at 37 °C. 100 mM HClO_4_ was added to denature the enzymes and after centrifugation, the clear supernatant was used for HPLC or LC-MS analysis.

#### Metabolic labeling of HeLa cells

HeLa cells (Merck, 93021013) were cultured in MEM Earl´s with 10% (v/v) fetal bovine serum (FCS, PAN), 1% (v/v) non-essential amino acids (NEAS, PAN) and 1% (v/v) glutamine solution (200 mM in PBS, PAN) in a humidified incubator at 37 °C and 5% CO_2_. The cells (10 mL of a 2 × 10^5^ cells mL^−1^ suspension) were seeded into cell culture dishes (Ø = 10 cm) and cultivated for 1 d. The medium was removed and the cells were starved in Met-deficient medium (Gibco) supplemented with 1% (v/v) glutamine solution and 1 mM l-cysteine for 30 min to deplete intracellular Met. PSH was added at different concentrations and the cells were cultivated for another 16 h. For negative controls, L-Met was added instead of PSH. The cells were washed with PBS, trypsinized and suspended in 20 mL of supplemented MEM Earl´s. The suspension was centrifuged (550 × *g*, 10 min), the medium was removed, and the pellet was stored at –80 °C.

#### Isolation of total RNA from HeLa cells

Cell pellets from HeLa cells (~2.5–3.0 × 10^5^ cells) were lysed mechanically by pipetting in 5 mL lysis buffer (10 mM TrisHCl (pH 8.0), 150 mM NaCl, 0.5 mM NP40, adjusted to pH 7.5 with HCl). Total RNA was purified by two consecutive phenol-chloroform extractions (4:1 and 2:1) followed by extraction with 1 mL CHCl_3_, back-extraction with NaCl (0.9%) and EtOH precipitation. The resulting total RNA was treated with 1 U DNase I (Thermo Fisher) in 1× DNase buffer (Thermo Fisher) for 30 min at 37 °C, followed by phenol-CHCl_3_ (5:1) extraction and EtOH precipitation.

#### Isolation of Poly(A)^+^ mRNA from HeLa cells

Extraction of poly(A)^+^ RNA directly from HeLa cell pellets (1–5 × 10^6^ cells) was performed with Sera-Mag Oligo(dT)-Coated Magnetic Particles according to manufacturer’s instructions. Extraction was performed once for SELECT assays and twice for LC-QqQ-MS and NGS.

#### Quantification of nucleic acids via spectrophotometric assay using TECAN/Nanodrop

To quantify purified nucleic acids and assess their purity, absorption at 260 nm and 280 nm were measured with a TECAN Infinite M1000 Pro in combination with NanoQuant plate or with NanoDrop Spectrophotometer. Purity was determined by the 260/280 nm absorption ratios.

#### Quantification of nucleic acids via NanoQuant plate adapted PicoGreen assay

Concentration of NGS libraries was determined using Quant-iT™ PicoGreen™ dsDNA Assay Kit (Invitrogen) following the procedure for fluorescence-based DNA quantification in small volumes by TECAN on NanoQuant Plate^TM^ (TECAN). Measurement was repeated trice for technical replicates from the same mix. Raw values were blank corrected and technical replicates were averaged. Concentrations were determined by comparison to lambda DNA standards.

#### Digestion of total RNA/poly(A)^+^ RNA for LC-MS-QqQ analysis

Digestions of isolated RNA was performed as above for determination of clicking efficiency, using 0.1 U nuclease P1 per 1 µg RNA.

#### Quantification of modified and unmodified nucleosides via LC-QqQ-MS

For quantification of required analytes, the calibration curves of external standards were measured. We used commercially available nucleosides for all standards (see Supplementary Table 13) except for 5-propargylcytidine (prop^5^C). Prop^5^C was synthesized and characterized (see Supplementary Methods).

Quantification of modified and unmodified nucleosides was performed on a LC-QqQ-MS system. Digested, dephosphorylated nucleoside mix was separated with a linear gradient from buffer A (20 mM NH_4_OAc in ddH_2_O (pH 6.0) to buffer B (acetonitrile) (see Supplementary Table 10) at 40 °C with 0.8 mL/min. The principle of measuring, optimizing mass spectrometric parameters, and quantifying modified nucleosides was described before in great detail by Muthmann et al.^114^. For best performance, mass spectrometric parameters such as fragmentor voltage (FV) and collision energy (CE) were optimized individually for each product ion. The optimized MS conditions are listed in Supplementary Table 9.

Mass measurements for several nucleosides were performed in parallel in dynamic MRM mode. The retention time (RET.) of all analytes and respective RET windows are listed in Supplementary Table 9. For quantification, pairs of precursor ion and product ion were detected, using the most abundant MRM transition—fragment of ribose loss or ribose derivative loss^114^—as quantifier. The second most abundant or specific MRM transition was used as a qualifier and the ratio between quantifier and qualifier signals was used as extra evidence for analyte identity. In the cases of prop^6^A and prop^5^C, two qualifiers were used.

#### NGS library preparation

NGS libraries from mRNA were prepared using a modified version of the iCLIP2 protocol^74^. 5 µg of twice poly(A)-enriched mRNA was fragmented at 88 °C (25 ng/µL mRNA) in 1× first strand buffer for 4 min, immediately cooled on ice (5 min) and precipitated. Successful fragmentation was confirmed via Bioanalyzer RNA analysis. Samples were biotinylated in a CuAAC reaction by combining the pre-ligated copper catalyst (1:5 CuSO_4_:THPTA, 5 min at rt) with RNA and biotin azide in sodium-phosphate buffer. The reaction was started by addition of sodium ascorbate (final concentrations: 5 mM CuSO_4_, 25 mM THPTA, 50 ng/µL RNA, 30 µM biotin azide, 60 mM sodium phosphate buffer, 100 mM sodium ascorbate), incubated for 30 min at 37 °C, stopped by addition of EDTA (10 mM) and directly purified using Microspin G-25 by following manufacturer instructions. Biotinylated RNA was bound to 500 µg streptavidin coated magnetic beads (Dynabeads^TM^ M-280 Streptavidin, Invitrogen^TM^) mostly following the manufacturer instructions for coupling nucleic acids except for two changes: (1) After initial washing, beads were preincubated with 0.2 µM poly(dT)-Oligo (10 min, rt, constant rotation) before adding the RNA to reduce unspecific background during binding and (2) incubation time of RNA during binding step was increased (1 h, rt, constant rotation).

Next, immobilized RNA was dephosphorylated at the 3′ end with T4 PNK (1× T4 PNK buffer A, 0.8 U/µL Ribolock, 0.4 U/µL T4 PNK in 50 µL reaction volume, 30 min, 37 °C, shaking at 1100 rpm) followed by a washing step for which the bead-bound RNA was precipitated with a magnet to remove supernatant, and then washed with 500 µL 1× B&W buffer, followed by 500 µL ddH_2_O. Subsequently, the L3-Adapter was ligated at the 3′end (1× Ligation buffer, 0.4 U/µL Ribolock, 1.5 µM L3-Adapter, 20% PEG and 0.5 U/µL T4 RNA ligase 1 in 20 µL final reaction volume, overnight, 16 °C, shaking at 1100 rpm). Beads were precipitated after adding 500 µL 1× B&W buffer to reduce viscosity of the mixture followed by a washing step as described before. Bead bound RNA was hybridized with the RT primer (0.05 µM) in 10 µL reaction volume for 5 min at 70 °C followed by cooling on ice for 5 min. RT was performed with SSIV (hybridized RNA+primer mix, 1× RT buffer, 5 mM DTT, 0.4 U/µL Ribolock, 1 mM of each dNTP, 1 U/µL SSIV, in 20 µL reaction volume, 30 min, 50 °C). RNA was hydrolyzed (80 mM NaOH, 10 min, 98 °C), reaction was neutralized (1 eq. HCl) and beads were removed from mix by magnetic precipitation. Resulting cDNA was purified using silane coated magnetic beads following the protocol described in^74^. Briefly, 10 µL of (MyOne^TM^ Silane Dynabeads^TM^, Invitrogen^TM^) were washed once with RLT buffer. cDNA was mixed with the washed beads in 125 µL of RLT buffer and precipitated with 1 vl of ethanol. After incubation at room temperature (2 × 5 min, resuspending inbetween) beads were washed with ethanol (80%) trice, precipitatet and dried (5 min, rt).

For PCR amplification, ligation of a second linker is needed. The resuspended, bead bound cDNA (in ddH_2_O) was heated with the respective barcode-linker (L##clip2.0) in DMSO to denature any secondary structures (2 min, 70 °C followed by 1 min, 0 °C) and 1 mM ATP, 0.75 U/µL RNA Ligase (high conc.) and 22.5% PEG8000 in 1× RNA ligase buffer with DTT (20 µL reaction volume total) were added. After homogenizing the viscous mixture, another 0.3 U RNA Ligase (high conc.) were added and reacted over night at 16 °C (shaking at 1100 rpm). Following the ligation, a second clean-up using silane coated magnetic beads following the protocol from the iCLIP2 protocol^74^ was performed adding fresh beads (5 µL). After the washing steps and precipitation, RNA was eluted from the beads in 23 µL ddH_2_O (incubation for 5 min at rt) and beads were removed by magnetic precipitation.

Purified cDNA was used in a first PCR amplification (0.2 mM of each dNTP, 0.5 µM of each Solexa_s primer (rev and fwd) and 0.02 U/µL Phusion DNA polymerase in 1× HF buffer, 98 °C for 30 s, 6 × [98 °C for 10 s, 5 °C for 30 s, 72 °C for 30 s], 72 °C for 3 min) to reduce loss in the first size amplification step. To remove adapter and primer contaminations from previous steps, reaction was size-selected using the ProNex size-selective purification system (Promega) following manufacturer instructions using a 3:1 v/v ratio of ProNex Chemistry to sample. Deviating from the protocol, the pre-library was eluted from the beads in 23 µL ddH2O instead of the provided elution buffer.

Cycle number for the final amplification of the libraries was determined by performing test-PCR reactions using 1 µL of the pre-library for 10 µL of reaction split to 3 aliquotes (0.2 mM of each dNTP, 0.5 µM of each Solexa primer (rev and fwd) and 0.02 U/µL Phusion DNA polymerase in 1× HF buffer amplified with different cycle numbers (98 °C for 30 s, 15–19 × [98 °C for 10 s, 5 °C for 30 s, 72 °C for 30 s], 72 °C for 3 min). Checking the size profile of these libraries either on a nat. PAGE or via bioanalyzer, cycle numbers were chosen to ensure enough amplification without giving rise to overamplification (increasing background amplification) or adapter-dimers (defined bands at ~170 bp typically separate from the broader library peak at 250–600 bp). Final PCR was performed with the determined cycle number (reduced by one cycle because the input is increased) in two separate reactions to ensure backup in case of unforeseen problems (10 µL of pre-library, 0.2 mM of each dNTP, 0.5 µM of each Solexa primer (rev and fwd) and 0.02 U/µL Phusion DNA polymerase in 1× HF buffer). Final library was purified by size-selection using the ProNex size-selective purification system (Promega) following manufacturer instructions using a 2.4:1 v/v ratio of ProNex Chemistry to sample and eluted from the beads in 20 µL ddH_2_O instead of the provided elution buffer. Quality of the finished libraries was checked via bioanalyzer-High sensitivity DNA assay (Agilent) and concentration was determined by Quant it PicoGreen Assay (Thermo Fisher).

#### Illumina sequencing

The libraries were sequenced on Illumina platform as paired-end with the read length 150 (PE150). The received amount of data per sample was 5G which corresponds to ~33 million reads.

#### SELECT

SELECT was used to independently validate selected putative m^6^A sites identified via MePMe-seq. Experiments were performed with poly(A)^+^ RNA (one time enriched via oligo(dT) magnetic beads) from HeLa cells. RNA was split and methylations were removed by treatment with FTO ( + FTO) from one half but not the other (– FTO) in 50 mM MES-buffer (pH 5.5), 283 µM (NH_4_)_2_Fe(SO_4_)_2_, 300 µM α-ketoglutarate, 2 mM ascorbic acid, 50 ng/µL poly(A)^+^ RNA, 0.2 U/µL RiboLock, 0.1 µg/µL FTO ( + FTO) or ddH_2_O (-FTO) for 30 min at 37 °C. Reaction was stopped by adding 20 mM EDTA (95 °C, 5 min)^92^. The poly(A)^+^ RNAs were purified immediately via RNA clean & concentrator kit by Zymo Research. For the SELECT assay poly(A)^+^ RNA ( + FTO and – FTO, respectively) was mixed with 85 pmol dTTP, 1× CutSmart buffer (50 mM potassium acetate, 20 mM Tris-acetate, 10 mM magnesium acetate,100 µg/mL BSA, pH 7.9, NEB), 20 U RiboLock, 80 fmol up primer and 80 fmol down primer in 15 µL ddH_2_O. Depending on the transcript expression level either 50 ng or 100 ng of + FTO and – FTO poly(A)^+^ RNA were used. The primers were annealed to the RNA (90 °C for 1 min; –10 °C/min for 4 min; 40 °C for 6 min; keep at 4 °C) and the enzyme mixture (0.01 U Bst 2.0 DNA polymerase, 0.5 U SplintR ligase, 10 nmol ATP and 1× CutSmart buffer in 5 µL ddH_2_O) was added. The reaction was carried out at 40 °C for 20 min, denatured at 80 °C for 20 min and kept at 4 °C. For the qPCR 1 µL of the reaction mixture was added to 9 µL of the *PowerUp™ SYBR*® *Green Master Mix*. The data was analyzed with Bio-Rad CFX Maestro 1.0 software. The obtained results were normalized by N-site.

#### Recombinant production of MTAN

Recombinant MTAN was expressed as described previously^115^. *E. coli* BL21 (DE3) cells were transformed with pProEx plasmid coding for MTAN and directly used to inoculate an overnight pre-culture in 2YT/Amp. After inoculating the main cultures in 2YT/Amp with 1% of the pre-culture, the cultures were grown at 37 °C for 3 h to an OD600 of 0.6. The cultures were cooled by placing at room temperature for 30 minutes then induced using 0.2 mM isopropyl-β-d-thiogalactopyranoside (IPTG and the expression was carried out overnight at 17 °C for ~18 h. Afterwards, the cells were centrifuged (5000 *g*, 20 min). After sonification (3 × 3 min, 30% amplitude) in lysis buffer (50 mM sodium phosphate pH 7.5) on ice, the cell lysate was centrifuged and the supernatant purified via ÄKTApurifier with a HisTrap FF column (1 mL; elution buffer: 50 mM sodium phosphate pH 7.5, 250 mM imidazole; storage buffer: 50 mM sodium phosphate pH 7.5, 50 mM Hepes, 10% glycerol).

#### Recombinant production of FTO

For the recombinant production of FTO, *E. coli* BL21 (DE3) cells were transformed with a pET28a vector encoding FTO. The cells were grown in LB medium at 37 °C to an OD_600_ of 1, kept at room temperature for 30 min, induced by adding 1 mM IPTG followed by expression overnight (16 °C). The cells were lysed in binding buffer (50 mM sodium phosphate buffer (pH 8), 300 mM NaCl, 50 mM imidazole) and the enzyme was purified in two steps using the ÄKTApurifier system. In a first purification step, the lysate was loaded on a 1 mL HisTrap FF column. The column was washed with 4% elution buffer (50 mM sodium phosphate buffer (pH 8), 300 mM NaCl, 500 mM imidazole) in binding buffer at 1 mL/min in 6 column volumes (CV), followed by a linear gradient from 4–100% elution buffer in 10 CV. The gradient was held at 21% elution buffer when FTO started to elute and then continued the linear gradient to 100%. In a second step, the enzyme was purified using a Superdex 200 increase column with running buffer (50 mM sodium phosphate buffer (pH 8), 300 mM NaCl, 10% glycerol) at 0.45 mL/min.

#### Recombinant expression and purification of GST-METTL16

Recombinant GST-METTL16 was expressed as described previously^116^. For construct refer to Supplementary Fig. 21. For expression of recombinant GST-METTL16 a Bac-to-Bac expression system with Sf21 insect cells (Thermo Fisher, 11497013) was used following the supplier’s instructions of the Bac-to-Bac baculovirus expression system (Invitrogen). The cells were sonicated in ice-cold lysis buffer (1×PBS (pH 7.4), 1.5 M NaCl, 2 mM DTT, 0.08 mM phenylmethanesulfonyl-fluoride (PMSF), a spatula tip of DNase I and 2.4 mM MgCl_2_) (3 × 3 min, 30% intensity). After centrifugation (7000 ×g, 30 min, 4 °C), the supernatant was sterile filtered (0.45 µm syringe filter, Thermo Scientific) and the protein was purified via affinity chromatography (GSTrap, 4B, GE Healthcare) on ÄKTA Purifier System (GE Healthcare) (elution buffer: 50 mM Tris (pH 8), 200 mM NaCl, 10 mM reduced L-glutathione). For RNA-free preparation, an anion exchange purification step was added (HiTrap Q, GE Healthcare) (buffer: 25 mM HEPES (pH 7.50), 50 mM NaCl, 1 mM TCEP). GST-METTL16 was concentrated to approximately 100 µM (Amicon Ultra-15, 10 kDa cutoff, Millipore), and the buffer was supplemented with glycerol (to 40%) for storage. The aliquots were flash-frozen in liquid N_2_ and stored at −80 °C.

#### In vitro propargylation of mRNA with METTL16

To propargylate isolated poly(A)^+^ RNA, 4 µg of RNA were incubated in 30 µL volume with 1 mM SeAdoYn, 10 µM METTL16 (RNA free), 1 U/µL Ribolock and 0.4 µM MTAN in 10× METTL16 activity buffer (100 mM HEPES-KOH, pH 7.4, 1 M NaCl) in 30 µL for 1 h at 37 °C. RNA was purified with RNA Clean & Concentrator^TM^-5 kit (Zymo Research Europe GmbH). 100 ng of reaction product were used for analysis on a denaturing agarose gel.

### Computational procedures

#### Preprocessing of raw sequencing data

The quality filtering, trimming and UMI preprocessing were performed in one step with fastp software^117^. Since the first 15 bases of read 1 had the structure (UMI1)_5 nt_(Experimental barcode)_6 nt_(UMI2)_4 nt_, the entire 15-mer was treated as UMI. The barcode-based demultiplexing was performed manually with standard command-line tools, in order to retain only the reads containing the correct experimental barcode. The alignment was performed with hisat2^118^, with the disabled soft-clipping, as recommended by Busch et al.^119^. The deduplication step was performed with UMI-tools^120^ with the default directional method to define duplicates, as recommended for highly over-amplified libraries^119^.

#### Termination calling via JACUSA2 and filtering

Differential reverse transcription termination signatures between treated and control samples were analyzed with our software package JACUSA2^77^ https://github.com/dieterich-lab/JACUSA2 using run mode “rt-arrest”. As one replicate of MePMe-seq CTR failed, MePMe-seq PSH rep1 was compared in further analysis with the libraries from untreated HeLa mRNA (METTL16 CTR). We used the following set of parameters: rt-arrest -m 0 -c 4 -p 10 -P1 FR-SECONDSTRAND -P2 FR-SECONDSTRAND, which disable quality clipping of read alignments (-m), require a minimal coverage of 4 reads across all samples (-c), run with 10 CPU threads (-p 10) and set library orientation to second strand in cDNA synthesis (-P1, -P2). JACUSA2 outputs genomic site coordinates, read coverage and termination rates per sample. The difference between arrest rates from sample and control (Δ_(RT arrest)_) was used to filter the terminations identified by the algorithm and to remove false positives. We retained high and low confident predictions by setting a sample read coverage threshold ( > 35 for high stringency (HS) filter, >20 for low stringency (LS) filter) and arrest score threshold (${\varDelta }_{{RT\; arrest}}$ > 20 for HS filter; ${\varDelta }_{{RT\; arrest}}$ > 15 for LS filter). The termination was localized to occur −1 nt of the modification site. We annotated the reported sites with genomic features such as gene locus, exon class or intron, distance to next TSS in 5′direction and the sequence context using bedtools 2.29.2. Mapped data for both replicates of MePMe-seq and in vitro METTL16 labeling in HeLa cells are available from the NCBI Sequence Read Archive (SRA) under https://www.ncbi.nlm.nih.gov/sra/PRJNA811414 upon publication.

#### Metatranscript analysis

Genomic coordinates were converted into cDNA coordinates using R language (version 4.0.2, https://www.R-project.org/)^121^ and the Bioconductor package ensembldb (version 2.14.1, PMID: 30689724). We always selected the longest transcript per locus to which genomic coordinates mapped to. For every protein-coding transcript, we computed the respective cDNA coordinate of a given termination site and reported the length of this matching transcript (in nt), the length of the coding sequence, five and three prime untranslated regions (UTR, in nt). Subsequently, and using this information, we used the same strategy as in MetaPlotR (PMID: 28158328) to visualize the read termination site distribution along a metatranscript profile (5′UTR + CDS + 3′UTR). Graphical density plots were generated using the ggplot2 library (version 3.3.2)^122^.

#### Cluster analysis

We analyzed the context of a given arrest site by clustering the signal (Diff Ctr-PSH) of positions –3,…, 0,…,2 by hierarchical clustering using Euclidean distance. We cut the corresponding dendrogram into two clusters and visualize the respective sites in a box plot.

#### Data acquisition

For comparison with MePMe-seq sites m^6^A, N_m_ and m^5^C datasets were downloaded from ATLAS^81^ and REPIC^82^ database. Additional datasets were acquired from the respective publications^64,91^. Coordinates that were not in hg38 were converted using browser based LiftOver tool (available at https://genome.ucsc.edu/cgi-bin/hgLiftOver^123^).

#### Statistics and reproducibility

For statistical analysis of metabolic labeling and SELECT, a one-sample one-tailed t-test (n.s. *P* > 0.05; *P ≤ 0.05; **P ≤ 0.01; ***P ≤ 0.001) was used. No data was excluded except from outliers of technical replicates of SELECT assay that were determined via Grubbs’ test.

#### NMR assignments

Specific NMR assignments were made only on the basis of a complete investigation using multidimensional NMR experiments (COSY, HMBC, HSQC). All spectra are included as an attachment in Supplement II.

### Reporting summary

Further information on research design is available in the Nature Portfolio Reporting Summary linked to this article.

### Supplementary information

Supplementary Information

Description of Additional Supplementary Files

Supplementary data 1

Supplementary data 2

Supplementary data 3

Supplementary data 4

Supplementary data 5

Supplementary data 6

Supplementary data 7

Reporting Summary

### Source data

Source Data

## Data Availability

The mapped data generated in this study for both replicates of MePMe-seq and in vitro METTL16 labeling in HeLa cells have been deposited in the NCBI Sequence Read Archive (SRA) under the bioproject accession code PRJNA811414. The processed data shown in this study, including JACUSA2 sites from both replicates of MePMe-seq (Supplementary data 1), identified m^6^A sites from MePMe-seq (Supplementary data 2), identified m^5^C sites from MePMe-seq (Supplementary data 6), JACUSA2 sites from both replicates of in vitro METTL16 labeling (Supplementary data 4) and identified m^6^A sites from in vitro METTL16 labeling (Supplementary data 5) are provided as Supplementary data. The public datasets of PA-m6A-CLIP, DART-seq, miCLIP, m6A-REF-seq were downloaded from ATLAS database [http://180.208.58.19/m6A-Atlas/browser.html?Type=H.sapiens], public datasets of eTAM-seq, m6A-SAC-seq, GLORI were downloaded from GEO database under accession codes GSE211303, GSE210563, GSE198246, respectively. The genome hg38 was downloaded from the following link: http://apr2019.archive.ensembl.org/index.html. Source data are provided with this paper.

## Code availability

JACUSA2 is available at https://github.com/dieterich-lab/JACUSA2.
