# Supplementary material for: MePMe-seq: antibody-free simultaneous m6A and m5C mapping in mRNA by metabolic propargyl labeling and sequencing
Source: Nat Commun. 2023 Nov 7;14:7154. doi: 10.1038/s41467-023-42832-z (PMC10630376; doi:10.1038/s41467-023-42832-z)
Supplement: Supplementary file 1 — Supplementary Information [file 41467_2023_42832_MOESM1_ESM.pdf]

Supplementary Tables: 1

Supplementary Table 1: **Number of reads after NGS sequencing and details from NGS data processing.** 1

Supplementary Table 2: **Pairwise comparison of miCLIP experiments from human samples.** 2

Supplementary Table 3: **Pairwise comparison of base resolution m^6A sequencing techniques.** 3

Supplementary Table 4: **Clipping of m6A sites found via MePMe-seq.** 4

Supplementary Table 5: **Pairwise comparison of base resolution m^5C sequencing experiment.** 5

Supplementary Table 6: **Pairwise comparison of bisulfite sequencing experiments from human samples.** 6

Supplementary Table 7: **Pairwise comparison of base resolution N_m sequencing experiment.** 8

Supplementary Table 8: **Pairwise comparison of base resolution m^7G sequencing experiment.** 9

Supplementary Table 9: **The MRM transitions ions and optimized parameters for the analysis of nucleosides.** 10

Supplementary Table 10: **Elution gradient and chromatographic parameters.** 11

Supplementary Table 11: **Oligonucleotide sequences used.** 12

Supplementary Table 12: **Reagents, chemicals and special solvents.** 14

Supplementary Table 13: **Commercially available compounds, used as standards for LC-QqQ-MS quantification.** 15

Supplementary Figures: 16

Supplementary Figure 1: **Optimizing feeding conditions for HeLa cells.** 16

Supplementary Figure 2: **Detection and quantification of modified nucleosides in mRNA.** 17

Supplementary Figure 3: **Detection and Quantification of modified nucleosides in mRNA.** 18

Supplementary Figure 4: **CuAAC efficiency.** 19

Supplementary Figure 5: **Optimizing termination during RT with SuperScript SSIV.** 20

Supplementary Figure 6: **Terminations during RT of RNA templates with different modifications.** 21

Supplementary Figure 7: **Scheme of bioinformatic steps performed for MePMe-seq and filter settings applied to the data sets.** 22

Supplementary Figure 8: **IGV browser coverage tracks of MePMe-seq data for YTHDF2 mRNA.** . 23

Supplementary Figure 9: **Enrichment and terminations at 5'end of transcripts.** 24

Supplementary Figure 10: **Clustering m^6A sites identified via MePMe-seq.** 25

Supplementary Figure 11: **Integrative genomics viewer (IGV) browser coverage tracks of MePMe-seq data for the MAT2A, MALAT1 and ACTB RNAs.** 26

Supplementary Figure 12: **Overlap between identified m^6A sites.** 27

Supplementary Figure 13: **Clustering of JACUSA2 hits around the TSS.** 28

Supplementary Figure 14: **Analysis of identified m^6A sites in mRNA.** 29

Supplementary Figure 15: **IGV browser coverage tracks of U6 snRNA.** 30

Supplementary Figure 16: **Additional SELECT results.** 31

Supplementary Figure 17: **Scheme of METTL16 dependent labeling combined with metabolic labeling to identify METTL16 targets.** 32

Supplementary Figure 18: **Overlap between two METTL16 labeling experiments after LS filtering.** 33

Supplementary Figure 19: **IGV browser coverage tracks of combined data for exemplary RNAs.** 34

Supplementary Figure 20: **IGV browser coverage tracks of combined METTL16 and metabolic labeling data for exemplary RNAs.** 35

Supplementary Figure 21: **Depiction of the METTL16-construct used in this work.** 36

Supplementary Figure 22: **Cluster analysis on the termination pattern around terminations next to A in mRNA.** 37

Supplementary Figure 23: **Termination pattern around all identified modified A sites in mRNA.** 38

Supplementary Figure 24: **IGV browser coverage tracks of potential m^1A sites in cytosolic ZNF664 mRNA and mitochondrial 16S rRNA.** 39

Supplementary Figure 25: **Consensus motifs for modified C.** 40

Supplementary Figure 26: **The signal intensity comparison of the MRM transition ions of $prop^5C$.** 41

Supplementary Figure 27: **The representation of all points of calibration (cc1 to cc7) curve of $prop^5C$.** 42

Supplementary Figure 28: **The representation of all points of calibration (cc1 to cc7) curve of $prop^5C$.** 43

Supplementary Figure 29: **The calibration curve of $prop^5C$ standard** (concentration = 4.9 nM = 1.39 pg/µL). The concentration level of $prop^5C$ in the real sample is indicated by the pink arrow. 44

Supplementary Figure 30: **The signal intensity comparison of the MRM transition ions of $prop^5C$**. 45

Supplementary Figure 31: **Synthesis of prop^{5}C standard.** 46

Supplementary Figure 32: **Synthesis of prop^{1}A (a), prop^{3}C (b), prop^{7}G (c) standards and prop^{5}C triphosphate (d)**. 47

Supplementary Figure 33: **^{1}H-NMR spectrum of compound 1**. 48

Supplementary Figure 34: **^{13}C-NMR spectrum of compound 1.** 49

Supplementary Figure 35: **COSY spectrum of compound 1.** 50

Supplementary Figure 36: **HSQC spectrum of compound 1**. 51

Supplementary Figure 37: **HMBC spectrum of compound 1**. 52

Supplementary Figure 38: **^{1}H-NMR spectrum of compound 2**. 53

Supplementary Figure 39: **^{13}C-NMR spectrum of compound 2**. 54

Supplementary Figure 40: **COSY spectrum of compound 2**. 55

Supplementary Figure 41: **HSQC spectrum of compound 2**. 56

Supplementary Figure 42: **HMBC spectrum of compound 2**. 57

Supplementary Figure 43: **^{1}H-NMR spectrum of compound 3**. 58

Supplementary Figure 44: **^{13}C-NMR spectrum of compound 3**. 59

Supplementary Figure 45: **COSY spectrum of compound 3**. 60

Supplementary Figure 46: **HSQC spectrum of compound 3.** 61

Supplementary Figure 47: **HMBC spectrum of compound 3**. 62

Supplementary Figure 48: **^{1}H-NMR spectrum of compound 4.** 63

Supplementary Figure 49: **^{13}C-NMR spectrum of compound 4**. 64

Supplementary Figure 50: **COSY spectrum of compound 4.** 65

Supplementary Figure 51: **HSQC spectrum of compound 4**. 66

Supplementary Figure 52: **HMBC spectrum of compound 4**. 67

Supplementary Figure 53: **^{1}H-NMR spectrum of compound 5**. 68

Supplementary Figure 54: **^{13}C-NMR spectrum of compound 5**. 69

Supplementary Figure 55: **COSY spectrum of compound 5**. 70

Supplementary Figure 56: **HSQC spectrum of compound 5.** 71

Supplementary Figure 57: **HMBC spectrum of compound 5.** 72

Supplementary Figure 58: ^{1}H-**NMR spectrum of compound 6** (measured on Bruker NEO 400). 73

Supplementary Figure 59: **^{13}C-NMR spectrum of compound 6** (measured on Bruker NEO 400). 74

Supplementary Figure 60: **COSY spectrum of compound 6** (measured on Agilent DD2 500). 75

Supplementary Figure 61: **HSQC spectrum of compound 6** (measured on Agilent DD2 500). 76

Supplementary Figure 62: **HMBC spectrum of compound 6** (measured on Agilent DD2 500). 77

Supplementary Figure 63: **^{1}H-NMR spectrum of compound 7**. 78

Supplementary Figure 64: **^{13}C-NMR spectrum of compound 7**. 79

Supplementary Figure 65: **COSY spectrum of compound 7**. 80

Supplementary Figure 66: **HSQC spectrum of compound 7.** 81

Supplementary Figure 67: **HMBC spectrum of compound 7**. 82

Supplementary Figure 68: **^{1}H-NMR spectrum of compound 8.** 83

Supplementary Figure 69: **^{13}C-NMR spectrum of compound 8**. 84

Supplementary Figure 70: **COSY spectrum of compound 8**. 85

Supplementary Figure 71: **HSQC spectrum of compound 8**. 86

Supplementary Figure 72: **HMBC spectrum of compound 8.** 87

Supplementary Figure 73: **^{1}H-NMR spectrum of compound 9**. 88

Supplementary Figure 74: **^{13}C-NMR spectrum of compound 9.** 89

Supplementary Figure 75: **COSY spectrum of compound 9.** 90

Supplementary Figure 76: **HSQC spectrum of compound 9.** 91

Supplementary Figure 77: **HMBC spectrum of compound 9.** 92

Supplementary Figure 78: **^{31}P-NMR spectrum of compound 9** (1: ^{31}P{^{1}H} decoupling; 2: no decoupling). 93

Supplementary Methods: 94

Synthesis of prop^{5}C, prop^{1}A, prop^{3}C, prop^{7}G and 5-propargylcytidine 5'-triphosphate 94

Supplementary References 100

Supplementary Tables:

Supplementary Table 1: **Number of reads after NGS sequencing and details from NGS data processing.**

Libraries were prepared from mRNA isolated from HeLa fed with 2.5 mM PSH (MePMe-seq PSH) and 2.5 mM MET (MePMe-seq CTR) or mRNA isolated from untreated HeLa cells (METTL16 CTR) and labeled in vitro using METTL16 (METTL16 SAMPLE). As one replicate of MePMe-seq CTR failed, MePMe-seq PSH rep1 was compared in further analysis with the libraries from untreated HeLa mRNA (METTL16 CTR).

Sample	MePMe-seq PSH rep1	MePMe-seq PSH rep2	MePMe-seq CTR rep2	METTL16 SAMPLE rep 1	METTL16 SAMPLE rep 2	METTL16 CTR rep1	METTL16 CTR rep2
Barcode	ATTGGC	GCTCAT	AAGCTA	TACAAG	ACATCG	AAGCTA	CGTGAT
Total read pairs	27,247,772	26,521,975	19,459,453	28,723,676	26,608,786	28,877,411	20,738,712
Good quality reads (FASTQ)	96.37 %	93.30 %	80.52 %	88.30 %	94.13 %	92.40 %	92.96 %
Alignment rate	94.41 %	89.38 %	88.00 %	92.99 %	88.84 %	94.66 %	89.51 %
Mapped reads removed in deduplication	61.55 %	46.62 %	68.03 %	71.34 %	84.81 %	58.97 %	75.32 %
Reads after UMI deduplication	14,782,330	15,463,622	4,658,002	7,167,391	3,430,028	10,590,131	3,592,481

Supplementary Table 2: **Pairwise comparison of miCLIP experiments from human samples.** Number of overlapping identified m^6A sites between the indicated experiments and the corresponding percentage (referred to the total number of sites in the respective experiment).

		26121403; HEK293; Control	26121403; HEK293; sysy antibody CITS	26593424; HepG2; Control	28920958; MOLM13; Control	30867593; HepG2; Control	31279658; HEK293T; Control	31328227; HCT116; Control
26121403; HEK293; Control	sites	7370	957	273	1528	137	3454	706
	% of all	100.0	13.0	3.7	20.7	1.9	46.9	9.6
26121403; HEK293; sysy antinbody CITS	sites	957	6215	160	1599	105	3450	588
	% of all	15.4	100.0	2.6	25.7	1.7	55.5	9.5
26593424; HepG2; Control	sites	273	160	12207	488	79	710	296
	% of all	2.2	1.3	100.0	4.0	0.6	5.8	2.4
28920958; MOLM13; Control	sites	1528	1599	488	20515	227	6633	1217
	% of all	7.4	7.8	2.4	100.0	1.1	32.3	5.9
30867593; HepG2; Control	sites	137	105	79	227	3423	518	109
	% of all	4.0	3.1	2.3	6.6	100.0	15.1	3.2
31279658; HEK293T; Control	sites	3454	3450	710	6633	518	43651	2514
	% of all	7.9	7.9	1.6	15.2	1.2	100.0	5.8
31328227; HCT116; Control	sites	706	588	296	1217	109	2514	6368
	% of all	11.1	9.2	4.6	19.1	1.7	39.5	100.0

Supplementary Table 3: **Pairwise comparison of base resolution m^{6}A sequencing techniques.** Shown are the number of overlapping identified m^{6}A sites between the indicated technique and the corresponding percentage (calculated referred to the total number of sites in the respective experiment). Where feasible, results from several experiments featuring the same technique and performed on human samples (could be from different human tissues/cell types) were pooled.

		MePMe-seq rep1 HS	MePMe-seq rep2 HS	DART seq	eTAM total	GLORI	m6A-label-seq total	m6A SAC	m6A-CLIP total	m6A-REF seq total	miCLIP total	PA-m6A-seq
MePMe-seq rep1 HS	sites	3841	1651	15	1968	3221	316	3124	2192	312	2556	595
	% of all	100.0	43.0	0.4	51.2	83.9	8.2	81.3	57.1	8.1	66.5	15.5
MePMe-seq rep2 HS	sites	1651	2312	6	1272	1966	227	1938	1364	259	1572	379
	% of all	71.4	100.0	0.3	55.0	85.0	9.8	83.8	59.0	11.2	68.0	16.4
DART seq	sites	15	6	8543	395	1100	8	1226	881	167	1318	309
	% of all	0.2	0.1	100.0	4.6	12.9	0.1	14.4	10.3	2.0	15.4	3.6
eTAM total	sites	1968	1272	395	23432	18013	300	15468	9625	918	12043	1675
	% of all	8.4	5.4	1.7	100.0	76.9	1.3	66.0	41.1	3.9	51.4	7.1
GLORI	sites	3221	1966	1100	18013	170240	1236	48957	25765	2663	40636	6264
	% of all	1.9	1.2	0.6	10.6	100.0	0.7	28.8	15.1	1.6	23.9	3.7
m6A-label-seq total	sites	316	227	8	300	1236	2510	925	717	89	805	158
	% of all	12.6	9.0	0.3	12.0	49.2	100.0	36.9	28.6	3.5	32.1	6.3
m6A SAC	sites	3124	1938	1226	15468	48957	925	129263	18546	1774	25182	4021
	% of all	2.4	1.5	0.9	12.0	37.9	0.7	100.0	14.3	1.4	19.5	3.1
m6A-CLIP total	sites	2192	1364	881	9625	25765	717	18546	50092	1657	22701	3467
	% of all	4.4	2.7	1.8	19.2	51.4	1.4	37.0	100.0	3.3	45.3	6.9
m6A-REF seq total	sites	312	259	167	918	2663	89	1774	1657	11474	2310	448
	% of all	2.7	2.3	1.5	8.0	23.2	0.8	15.5	14.4	100.0	20.1	3.9
miCLIP total	sites	2556	1572	1318	12043	40636	805	25182	22701	2310	80100	5309
	% of all	3.2	2.0	1.6	15.0	50.7	1.0	31.4	28.3	2.9	100.0	6.6
PA-m6A-seq	sites	595	379	309	1675	6264	158	4021	3467	448	5309	19680
	% of all	3.0	1.9	1.6	8.5	31.8	0.8	20.4	17.6	2.3	27.0	100.0

Supplementary Table 4: **Clipping of m6A sites found via MePMe-seq.**
Shown are sites found in MALAT1, MYC, NT5DC2 and RBM transcripts that were found in both replicates of either METTL16 *in vitro* labeling or metabolic labeling.

coordinates (hg38)						Sequence context					MePMe-seq			
arrest Chr	arrest Start	arrest End	Strand	modification	gene	-2	-1	0	1	2	METTL16 rep1 HS	METTL16 rep2 HS	metabolic rep1 HS	metabolic rep2 HS
11	65503012	65503013	+	65503012	MALAT1	A	C	A	C	T	true	true		
11	65502223	65502224	+	65502223	MALAT1	A	C	A	A	T	true	true		
11	65502935	65502936	+	65502935	MALAT1	A	C	A	A	T	true	true		
11	65505045	65505046	+	65505045	MALAT1	A	C	A	T	A	true	true		
11	65503390	65503391	+	65503390	MALAT1	A	C	A	C	T	true	true		
11	65501369	65501370	+	65501369	MALAT1	A	C	A	G	T	true	true		
11	65504575	65504576	+	65504575	MALAT1	A	C	A	T	T	true	true		
11	65499371	65499372	+	65499371	MALAT1	A	C	A	G	A	true	true		
11	65504068	65504069	+	65504068	MALAT1	A	C	A	C	T	true	true		
11	65504324	65504325	+	65504324	MALAT1	A	C	A	G	A	true	true		
11	65504814	65504815	+	65504814	MALAT1	A	C	A	T	G	true	true		
8	127741111	127741112	+	127741111	MYC	G	G	A	C	T			true	true
8	127740891	127740892	+	127740891	MYC	G	G	A	C	T			true	true
8	127740621	127740622	+	127740621	MYC	G	G	A	C	A			true	true
8	127738657	127738658	+	127738657	MYC	G	G	A	C	T			true	true
8	127736472	127736473	+	127736472	MYC	A	C	A	A	C	true	true		
8	127740845	127740846	+	127740845	MYC	A	C	A	T	C	true	true		
8	127741209	127741210	+	127741209	MYC	A	C	A	C	A	true	true		
3	52529150	52529151	-	52529152	NT5DC2	A	C	A	A	G	true	true		
3	52528192	52528193	-	52528194	NT5DC2	A	C	A	A	G	true	true		
3	52528903	52528904	-	52528905	NT5DC2	A	C	A	A	C	true	true		
X	48577066	48577067	+	48577066	RBM3	A	C	A	G	A	true	true		

Supplementary Table 5: **Pairwise comparison of base resolution m^{5}C sequencing experiment.** Number of overlapping identified m^{5}C sites between the indicated technique and the corresponding percentage (calculated referred to the total number of sites in the respective experiment). Experiments performed in the same study (different tissues) were pooled.

		23604283 Aza-IP	27356879 miCLIP	22344696 BS-seq	27356879 BS-seq	28418038 (all) BS-seq	30526041 BS-seq	30872485 RBS seq	31061524 (all) BS-seq	31358969 BS-seq	MePMe-seq (HS)
23604283 Aza-IP	sites	597	4	2	12	5	119	4	1	0	0
	% of all	100.0	0.7	0.3	2.0	0.8	19.9	0.7	0.2	0.0	0.0
27356879 miCLIP	sites	4	361	1	86	361	37	0	0	94	0
	% of all	1.1	100.0	0.3	23.8	100.0	10.2	0.0	0.0	26.0	0.0
22344696 BS-seq	sites	2	1	5246	5	342	16	118	81	289	2
	% of all	0.0	0.0	100.0	0.1	6.5	0.3	2.2	1.5	5.5	0.0
27356879 BS-seq	sites	12	86	5	2357	94	73	5	2	212	0
	% of all	0.5	3.6	0.2	100.0	4.0	3.1	0.2	0.1	9.0	0.0
28418038 (all) BS-seq	sites	5	361	342	94	40554	65	495	1040	4397	22
	% of all	0.0	0.9	0.8	0.2	100.0	0.2	1.2	2.6	10.8	0.1
30526041 BS-seq	sites	119	37	16	73	65	12442	14	2	82	0
	% of all	1.0	0.3	0.1	0.6	0.5	100.0	0.1	0.0	0.7	0.0
30872485 RBS seq	sites	4	0	118	5	495	14	2159	161	327	2
	% of all	0.2	0.0	5.5	0.2	22.9	0.6	100.0	7.5	15.1	0.1
31061524 (all) BS-seq	sites	1	0	81	2	1040	2	161	3096	503	7
	% of all	0.0	0.0	2.6	0.1	33.6	0.1	5.2	100.0	16.2	0.2
31358969 BS-seq	sites	0	94	289	212	4397	82	327	503	20488	8
	% of all	0.0	0.5	1.4	1.0	21.5	0.4	1.6	2.5	100.0	0.0
MepMe-seq (HS)	sites	0	0	2	0	22	0	2	7	8	1305
	% of all	0.0	0.0	0.2	0.0	1.7	0.0	0.2	0.5	0.6	100.0

Supplementary Table 6: **Pairwise comparison of bisulfite sequencing experiments from human samples.**
Number of overlapping identified m^5C sites between the indicated experiments and the corresponding percentage (referred to the total number of sites in the respective experiment). Blue regions mark experiments performed in the same study from different tissues, orange regions mark experiments performed in HeLa cells in different studies.

		22344696	27356879 GSE66011	28418038 GSE74771	28418038 GSE74772	28418038 GSE74773	28418038 GSE74774	28418038 GSE74775	28418038 GSE93752	30526041 GSE122413	30872485 GSE90963	31061524 GSE122260	31061524 GSE122261	31061524 GSE122263	31061524 GSE122264	31061524 GSE122265	31061524 GSE122266	31061524 GSE122267	31061524 GSE122268	31061524 GSE122269	31358969 GSE133672
22344696	sites	5246	5	140	159	130	137	150	259	16	118	24	68	18	11	20	17	20	7	44	289
	% of all	100.0	0.1	2.7	3.0	2.5	2.6	2.9	4.9	0.3	2.2	0.5	1.3	0.3	0.2	0.4	0.3	0.4	0.1	0.8	5.5
27356879 GSE66011	sites	5	2357	88	9	3	4	5	3	73	5	2	1	2	2	2	2	2	2	2	212
	% of all	0.2	100.0	3.7	0.4	0.1	0.2	0.2	0.1	3.1	0.2	0.1	0.0	0.1	0.1	0.1	0.1	0.1	0.1	0.1	9.0
28418038 GSE74771	sites	140	88	11235	3851	3078	2856	3196	1817	48	210	149	298	111	51	84	84	57	53	177	1990
	% of all	1.2	0.8	100.0	34.3	27.4	25.4	28.4	16.2	0.4	1.9	1.3	2.7	1.0	0.5	0.7	0.7	0.5	0.5	1.6	17.7
28418038 GSE74772	sites	159	9	3851	13803	3202	3012	3942	2144	9	238	133	276	116	46	78	78	55	41	176	2353
	% of all	1.2	0.1	27.9	100.0	23.2	21.8	28.6	15.5	0.1	1.7	1.0	2.0	0.8	0.3	0.6	0.6	0.4	0.3	1.3	17.0
28418038 GSE74773	sites	130	3	3078	3202	9367	2646	2940	1703	15	227	117	274	109	50	84	83	56	48	159	1797
	% of all	1.4	0.0	32.9	34.2	100.0	28.2	31.4	18.2	0.2	2.4	1.2	2.9	1.2	0.5	0.9	0.9	0.6	0.5	1.7	19.2
28418038 GSE74774	sites	137	4	2856	3012	2646	8770	2945	1688	9	228	84	217	85	46	82	54	53	39	125	1848
	% of all	1.6	0.0	32.6	34.3	30.2	100.0	33.6	19.2	0.1	2.6	1.0	2.5	1.0	0.5	0.9	0.6	0.6	0.4	1.4	21.1
28418038 GSE74775	sites	150	5	3196	3942	2940	2945	11220	2059	7	291	119	383	123	69	111	90	91	57	227	2261
	% of all	1.3	0.0	28.5	35.1	26.2	26.2	100.0	18.4	0.1	2.6	1.1	3.4	1.1	0.6	1.0	0.8	0.8	0.5	2.0	20.2
28418038 GSE93752	sites	259	3	1817	2144	1703	1688	2059	6734	7	404	160	681	134	74	133	116	100	66	278	2488
	% of all	3.8	0.0	27.0	31.8	25.3	25.1	30.6	100.0	0.1	6.0	2.4	10.1	2.0	1.1	2.0	1.7	1.5	1.0	4.1	36.9
30526041 GSE122413	sites	16	73	48	9	15	9	7	7	12442	14	2	2	2	2	2	2	2	2	2	82
	% of all	0.1	0.6	0.4	0.1	0.1	0.1	0.1	0.1	100.0	0.1	0.0	0.0	0.0	0.0	0.0	0.0	0.0	0.0	0.0	0.7
	sites	118	5	210	238	227	228	291	404	14	2159	57	144	48	38	57	40	34	34	86	327

		22344696	27356879 GSE66011	28418038 GSE74771	28418038 GSE74772	28418038 GSE74773	28418038 GSE74774	28418038 GSE74775	28418038 GSE93752	30526041 GSE122413	30872485 GSE90963	31061524 GSE122260	31061524 GSE122261	31061524 GSE122263	31061524 GSE122264	31061524 GSE122265	31061524 GSE122266	31061524 GSE122267	31061524 GSE122268	31061524 GSE122269	31358969 GSE133672
30872485 GSE90963	% of all	5.5	0.2	9.7	11.0	10.5	10.6	13.5	18.7	0.6	100.0	2.6	6.7	2.2	1.8	2.6	1.9	1.6	1.6	4.0	15.1
31061524 GSE122260	sites	24	2	149	133	117	84	119	160	2	57	257	187	108	46	86	74	49	46	136	128
	% of all	9.3	0.8	58.0	51.8	45.5	32.7	46.3	62.3	0.8	22.2	100.0	72.8	42.0	17.9	33.5	28.8	19.1	17.9	52.9	49.8
31061524 GSE122261	sites	68	1	298	276	274	217	383	681	2	144	187	1232	180	89	164	131	125	81	324	410
	% of all	5.5	0.1	24.2	22.4	22.2	17.6	31.1	55.3	0.2	11.7	15.2	100.0	14.6	7.2	13.3	10.6	10.1	6.6	26.3	33.3
31061524 GSE122263	sites	18	2	111	116	109	85	123	134	2	48	108	180	514	68	115	77	67	63	166	136
	% of all	3.5	0.4	21.6	22.6	21.2	16.5	23.9	26.1	0.4	9.3	21.0	35.0	100.0	13.2	22.4	15.0	13.0	12.3	32.3	26.5
31061524 GSE122264	sites	11	2	51	46	50	46	69	74	2	38	46	89	68	218	84	80	104	55	89	69
	% of all	5.0	0.9	23.4	21.1	22.9	21.1	31.7	33.9	0.9	17.4	21.1	40.8	31.2	100.0	38.5	36.7	47.7	25.2	40.8	31.7
31061524 GSE122265	sites	20	2	84	78	84	82	111	133	2	57	86	164	115	84	351	94	87	94	167	115
	% of all	5.7	0.6	23.9	22.2	23.9	23.4	31.6	37.9	0.6	16.2	24.5	46.7	32.8	23.9	100.0	26.8	24.8	26.8	47.6	32.8
31061524 GSE122266	sites	17	2	84	78	83	54	90	116	2	40	74	131	77	80	94	381	84	65	137	80
	% of all	4.5	0.5	22.0	20.5	21.8	14.2	23.6	30.4	0.5	10.5	19.4	34.4	20.2	21.0	24.7	100.0	22.0	17.1	36.0	21.0
31061524 GSE122267	sites	20	2	57	55	56	53	91	100	2	34	49	125	67	104	87	84	369	56	124	76
	% of all	5.4	0.5	15.4	14.9	15.2	14.4	24.7	27.1	0.5	9.2	13.3	33.9	18.2	28.2	23.6	22.8	100.0	15.2	33.6	20.6
31061524 GSE122268	sites	7	2	53	41	48	39	57	66	2	34	46	81	63	55	94	65	56	276	96	62
	% of all	2.5	0.7	19.2	14.9	17.4	14.1	20.7	23.9	0.7	12.3	16.7	29.3	22.8	19.9	34.1	23.6	20.3	100.0	34.8	22.5
31061524 GSE122269	sites	44	2	177	176	159	125	227	278	2	86	136	324	166	89	167	137	124	96	1162	226
	% of all	3.8	0.2	15.2	15.1	13.7	10.8	19.5	23.9	0.2	7.4	11.7	27.9	14.3	7.7	14.4	11.8	10.7	8.3	100.0	19.4
31358969 GSE133672	sites	289	212	1990	2353	1797	1848	2261	2488	82	327	128	410	136	69	115	80	76	62	226	20488
	% of all	1.4	1.0	9.7	11.5	8.8	9.0	11.0	12.1	0.4	1.6	0.6	2.0	0.7	0.3	0.6	0.4	0.4	0.3	1.1	100.0

Supplementary Table 7: **Pairwise comparison of base resolution N_m sequencing experiment.** Shown are the overlapping identified N_m sites between MePMe-seq (HS filtering) and N_m-seq splitted into internal and cap adjacent sites.

Set	Chr	modified site	Strand	gene	-2	-1	***0***	1	2	internal or cap adjacent (≤5 nt to cap)
rep1	21	8214287	+	FP671120.2	A	G	**A**	G	A	internal
rep1	21	8400537	+	FP236383.1	G	G	**G**	G	A	internal
rep2	21	8400537	+	FP236383.1	G	G	**G**	G	A	internal
rep1	21	8400628	+	FP236383.1	C	A	**A**	A	G	internal
rep1	21	8400634	+	FP236383.1	G	A	**A**	G	A	internal
rep2	21	8400634	+	FP236383.1	G	A	**A**	G	A	internal
rep1	21	8401216	+	FP236383.1	A	G	**T**	A	C	internal

Supplementary Table 8: **Pairwise comparison of base resolution m^7G sequencing experiment.** Overlapping identified m^7G sites between MePMe-seq (HS filtering) and m^7G-MeRIP-seq are splitted into internal and cap adjacent sites.

Set	Chr	modified site	Strand	gene	-2	-1	***0***	1	2	internal or cap adjacent (≤5 nt to cap)
rep1	1	40040728	+	CAP1	G	C	**G**	G	A	cap adjacent
rep2	1	40040728	+	CAP1	G	C	**G**	G	A	cap adjacent
rep1	1	151347253	-	RFX5	T	G	**G**	G	C	cap adjacent
rep1	2	218270520	+	PNKD	C	G	**G**	G	G	cap adjacent
rep2	4	128287816	-	PGRMC2	A	G	**G**	A	G	cap adjacent
rep2	11	74949262	+	SPCS2	G	G	**G**	A	G	cap adjacent
rep2	12	76084688	-	NAP1L1	T	G	**G**	G	T	cap adjacent
rep1	17	7012623	+	RNASEK	T	G	**G**	G	C	cap adjacent
rep2	19	10577349	-	AP1M2	G	G	**G**	G	C	internal

Supplementary Table 9: **The MRM transitions ions and optimized parameters for the analysis of nucleosides.**

All MRMs were conducted in positive mode.

Analytes	MRM transition ions/(m/z)	type	Method	RT/min	RT windows/min	FV/V	CE/V	CAV/V
A	268.1 → 136.0	Quantifier	1	3.9	0.6	100	9	9
A	268.1 → 119.0	Qualifier	1	3.9	0.6	100	45	9
C	244.0 → 111.9	Quantifier	1	2.2	0.7	85	5	9
C	244.0 → 95.1	Qualifier	1	2.2	0.7	85	41	9
A_m	282.1 → 136.1	Quantifier	1	4.17	0.6	109	9	9
A_m	282.1 → 69.1	Qualifier	1	4.17	0.6	109	25	9
m^6A	282.1 → 150.1	Quantifier	1	4.29	0.7	120	13	9
m^6A	282.1 → 108.0	Qualifier	1	4.29	0.7	120	65	9
A_{prop}	306.1 → 136.1	Quantifier	2	4.4	0.5	109	9	9
A_{prop}	306.1 → 118.8	Qualifier	2	4.4	0.5	109	49	9
$prop^6A$	306.1 → 174.0	Quantifier	2	4.7	0.6	121	9	9
$prop^6A$	306.1 → 148.0	Qualifier	2	4.7	0.6	121	33	9
$prop^6A$	306.1 → 108.0	Qualifier	2	4.7	0.6	121	57	9
m^5C	258.1 → 126.0	Quantifier	2	3.5	0.4	96	4	9
m^5C	258.1 → 108.9	Qualifier	2	3.5	0.4	104	15	9
$prop^5C$	282.1 → 150.1	Quantifier	2	3.9	0.4	76	0	9
$prop^5C$	282.1 → 121.9	Qualifier	2	3.9	0.4	100	25	9
$prop^5C$	282.1 → 80.0	Qualifier	2	3.9	0.4	100	41	9
C_{prop}	282 → 111.9	Quantifier	3	3.7	1	80	0	9
G_{prop}	322.1 → 151.9	Quantifier	3	4.1	1	92	0	9
$prop^1A$	306.1 → 134.9	Quantifier	3	3.7	1	84	40	9
$prop^3C$	282.1 → 107	Quantifier	3	3.6	1	88	20	9
$prop^7G$	322.1 → 151.1	Quantifier	3	3.8	1	80	36	9

Supplementary Table 10: **Elution gradient and chromatographic parameters.**

Time/min	Buffer B/%
0.0	0
1.0	0
7.0	60
7.2	100
8.8	100
9.0	0
12.0	0

Supplementary Table 11: **Oligonucleotide sequences used.**
(*//////* = index sequence | N = randomized nucleotides | /5phos/ = phosphate | /rApp/ = 5'-adenylation | /3ddC/ = 3'-dideoxy-C)

Name	Oligonucleotidesequence	Source
**T7 template A rev**	GCACAGAGCAGCAAGAGGCAGTCCCGGGAGAGCGCCTATAGTGAG TCGTATTA	Biolegio
**T7 primer**	TAATACGACTCACTATAGG	Biolegio
**RT primer 1**	GCACAGAGCAGCAAGAG	Biolegio
**poly(dT)-Oligo**	TTTTTTTTTT	Biolegio
**L3-Adapter**	/rApp/AGATCGGAAGAGCGGTTCAG/ddC/	IDT
**RT primer 2**	GGATCCTGAACCGCT	Biolegio
**L##clip2.0**	/5Phos/NNNN*//////*NNNNNAGATCGGAAGAGCGTCGTG/3ddC/	IDT
**P5Solexa_s**	ACACGACGCTCTTCCGATCT	Biolegio
**P3Solexa_s**	CTGAACCGCTCTTCCGATCT	Biolegio
**P5Solexa**	AATGATACGGCGACCACCGAGATCTACACTCTTTCCCTACACGACGC TCTTCCGATCT	Biolegio
**P3Solexa**	CAAGCAGAAGACGGCATACGAGATCGGTCTCGGCATTCCTGCTGAA CCGCTCTTCCGATCT	Biolegio
**MALAT1_11:65.500.272_down**	/5phos/CACATTTTTCAAACTAAGCTACTcagaggctgagtcgctgcat	Biolegio
**MALAT1_11:65.500.272_up**	tagccagtaccgtagtgcgtgAATTACTTCCGTTACGAAAGTCCT	Biolegio
**MALAT1_1165.500.338_down**	/5phos/CCAATGCAAAAACATTAAGTcagaggctgagtcgctgcat	Biolegio
**MALAT1_11:65.500.338_up**	tagccagtaccgtagtgcgtgGGATTTAAAAAATAATCTTAACTCAAAG	Biolegio
**FLNB_3:58131631_down**	/5phos/GTGAATCCAAGAACATATCATTGGCGAGcagaggctgagtcgct gcat	Biolegio
**FLNB_3:58131631_up**	tagccagtaccgtagtgcgtgGAGGGAAAACCCCATTTGCCACTTC	Biolegio
**FLNB_3:58131742_down**	/5phos/AATTTCCCCCAAACCATTTCTGCCCcagaggctgagtcgctgcat	Biolegio
**FLNB_3:58131742_up**	tagccagtaccgtagtgcgtgGAATCTGATCCCAGACCCATGACC	Biolegio
**CTNNB1_3:41239837_down**	/5phos/CCATTTGTATTGTTACTCCTCGcagaggctgagtcgctgcat	Biolegio
**CTNNB1_3:41239837_up**	tagccagtaccgtagtgcgtgTCTTCACTTCTTGAGTCACTCCCAAAA	Biolegio
**CTNNB1_3:41239763_down**	/5phos/CAGCTTGGTTAGTGTGTCAGGCACTcagaggctgagtcgctgcat	Biolegio
**CTNNB1_3:41239763_up**	tagccagtaccgtagtgcgtgAGTTTACTTCAATTGTTCCCATAGGAAAC	Biolegio
**AHNAK_11:62518109_up**	tagccagtaccgtagtgcgtgCCTTTCAGGTTCACATCCACACC	Biolegio
**AHNAK_11:62518109_down**	/5phos/GGCCCCTTCAGTTCGCCAGAcagaggctgagtcgctgcat	Biolegio
**AHNAK_11:62518059_down**	/5phos/CCACATTCGGTGCTGAAAcagaggctgagtcgctgcat	Biolegio
**AHNAK_11:62518059_up**	tagccagtaccgtagtgcgtgTTGGTCCTTCCAAGTTAAAG	Biolegio
**WDR6_3:49012974_up**	tagccagtaccgtagtgcgtgGGCAGCAGGTACCGACAACG	Biolegio
**WDR6_3:49012974_down**	/5phos/TCCTTGACAAAGATGGCCTTGCCcagaggctgagtcgctgcat	Biolegio
**WDR6_3:49012969_up**	tagccagtaccgtagtgcgtgCAGCAGGTACCGACAACGTTCCT	Biolegio
**WDR6_3:49012969_down**	/5phos/GACAAAGATGGCCTTGCCAGAGGcagaggctgagtcgctgcat	Biolegio
**MARCH6_5:10433736_up**	tagccagtaccgtagtgcgtgTGGGAAAAATCTCCAAAGAGAGAAG	Biolegio
**MARCH6_5:10433736_down**	/5phos/CCACAAAAAAGGACATGTAAAGGGcagaggctgagtcgctgcat	Biolegio
**MARCH6_5:10433720_up**	tagccagtaccgtagtgcgtgGAGAGAAGTCCACAAAAAAGGACA	Biolegio
**MARCH6_5:10433720_down**	/5phos/GTAAAGGGGAAGGTCAAGTTGTTGcagaggctgagtcgctgcat	Biolegio
**NFX1_9:33348009_up**	tagccagtaccgtagtgcgtgCTTTCCCCAGAGTCCCCAAAG	Biolegio
**NFX1_9:33348009_down**	/5phos/CCATTGTATCATTCTCATGCCTTTGCcagaggctgagtcgctgcat	Biolegio
**NFX1_9:33348002_up**	tagccagtaccgtagtgcgtgCCAGAGTCCCCAAAGTCCATTG	Biolegio

**NFX1_9:33348002_down**	/5phos/ATCATTCTCATGCCTTTGCGTCCcagaggctgagtcgctgcat	Biolegio
**SRRM2_16:2768757_up**	tagccagtaccgtagtgcgtgGGATCAAGGTTAAACCTCAAGAATG	Biolegio
**SRRM2_16:2768757_down**	/5phos/CCCTGATGGAGGAATAAGGCcagaggctgagtcgctgcat	Biolegio
**SRRM2_16:2768735_up**	tagccagtaccgtagtgcgtgCCCTGATGGAGGAATAAGGCC	Biolegio
**SRRM2_16:2768735_down**	5phos/CTGGGCTCAGGTGTGGAAGcagaggctgagtcgctgcat	Biolegio
**MAT2A_2:85544902_down**	5phos/GTACTTACGCCATACCCCcagaggctgagtcgctgcat	Biolegio
**MAT2A_2:85544902_up**	tagccagtaccgtagtgcgtgCTGAGGTGATGGCTTCTC	Biolegio
**MAT2A_2:85544538_up**	tagccagtaccgtagtgcgtgACTAGGAGTTGGTGAGGTTTGGC	Biolegio
**MAT2A_2:85544538_down**	5phos/AGTGCTGAAACCATGCATAGGATTGcagaggctgagtcgctgcat	Biolegio
**SELECT PCR fwd**	ATGCAGCGACTCAGCCTCTG	Biolegio
**SELECT PCR rev**	TAGCCAGTACCGTAGTGCGTG	Biolegio
**T7 template C fwd**	TAATACGACTCACTATAGGATGGTTAGGTGCAGGTGTTGAGTGAGG TTGAGGG	Biolegio
**T7 template C rev**	CCCTCAACCTCACTCAACACCTGCACCTAACCATCCTATAGTGAGTCG TATTA	Biolegio
**A-36 mer**	GGCGCUCUCCCGGGACUGCCUCUUGCUGCUCUGUGC	Biomers
**A_{prop}-36 mer**	GGCGCUCUCCCGGGA_{prop}CUGCCUCUUGCUGCUCUGUGC	Biomers
**G_{prop}-36 mer**	GGCGCUCUCCCGGG_{prop}ACUGCCUCUUGCUGCUCUGUGC	Biomers
**C_{prop}-36 mer**	GGCGCUCUCCC_{prop}GGGACUGCCUCUUGCUGCUCUGUGC	Biomers
**U_{prop}-36 mer**	GGCGCUCU_{prop}CCCGGGACUGCCUCUUGCUGCUCUGUGC	Biomers
**RT primer 3**	CCCTCAACCTCACTCAA	Biolegio

Supplementary Table 12: **Reagents, chemicals and special solvents.**

**Chemicals**	**CAS**	**Vendor**	**Cat.No.**
1*H*-Imidazole	288-32-4	AppliChem	A1073,1000
4-Methylbenzene-1-sulfonyl chloride	98-59-9	Acros Organics	139031000
5-Methyluridine [m5U]	1463-10-1	Sigma-Aldrich	535893-25G
Acetic acid (LC-MS grade)	64-19-7	Merck	5.33001.0050
Ammonia solution (7N in methanol)	7664-41-7	Acros Organics	428381000
Ammonium acetate (LC-MS grade)	631-61-8	VWR	84885.180
Ammonium chloride	12125-02-9	Acros Organics	123340010
Ammonium fluoride	12125-01-8	Sigma-Aldrich	216011-100G
Azobisisobutyronitrile	78-67-1	Sigma-Aldrich	441090-25G
Bis-(tri-*n*-butylammonium) pyrophosphate	5975-18-8	Sigma-Aldrich	P8533-1G
Copper(I) cyanide	544-92-3	Merck	8.41811.0100
Ethynylmagnesium bromide (0.5M in THF)	4301-14-8	Sigma-Aldrich	346152-
Guanosine [G]	118-00-3	Alfa Aesar	A11328
N-Bromosuccinimide	128-08-5	Alfa Aesar	A15922
N-Methylpiperidine	626-67-5	Acros Organics	127480100
Phosphoroxychloride	10025-87-3	Fisher Scientific	10032470
Propargyl bromide	106-96-7	Alfa Aesar	L10595
tert-Butyldimethylsilyl chloride	18162-48-6	Sigma-Aldrich	190500-100G
Triethylamine	121-44-8	Carl Roth	X875.4
Dowex® 50WX8	11119-67-8	Thermo	L13922.30
POROS™ HQ 50	-	Thermo	1365906

**Special solvents**	**CAS**	**Vendor**	**Cat.No.**
Acetonitrile (HPLC grade)	75-05-8	Honeywell	34851-2.5L
Acetonitrile (LC-MS grade)	75-05-8	Fisher Scientific	A/0638/17
Dichloromethane (dry over molecular sieve)	75-09-2	Acros Organics	348465000
Dimethylformamide (dry over mol. sieve)	68-12-2	Acros Organics	348431000
Dimethyl sulfoxide	67-68-5	Fisher Scientific	10206581
Tetrahydrofuran (dry over mol. sieve) [THF]	109-99-9	Acros Organics	348451000
Trimethyl phosphate	512-56-1	Acros Organics	10593701

Supplementary Table 13: **Commercially available compounds, used as standards for LC-QqQ-MS quantification.**

Standards for LC-QqQ-MS quantification	CAS number	Vendor	Cat.No.
2’-(O-Propargyl)-adenosine [A_{prop}]	151390-97-5	Jena Bioscience	CLK-RP-3401-10
2'-O-Methyladenosine [A_m]	2140-79-6	Cayman Chemical	16936
5-Methyl-cytidine [m^5C]	2140-61-6	Jena Bioscience	N-RP-1832-1G
Adenosine [A]	58-61-7	Sigma-Aldrich	A9251-25G
Cytidine [C]	65-46-3	Sigma-Aldrich	C122106-1G
N^6-Methyladenosine [m^6A]	1867-73-8	Carbosynth	NM32281
N6-Propargyl-adenosine [$prop^6A$]	-	Jena Bioscience	CLK-N004-5
2‘-O-Propargylcytidine	206552-85-4	Biozol	CBS-NP06202
2‘-O-Propargylguanosine	206552-86-5	Biozol	CBS-NP06253

Supplementary Figures:

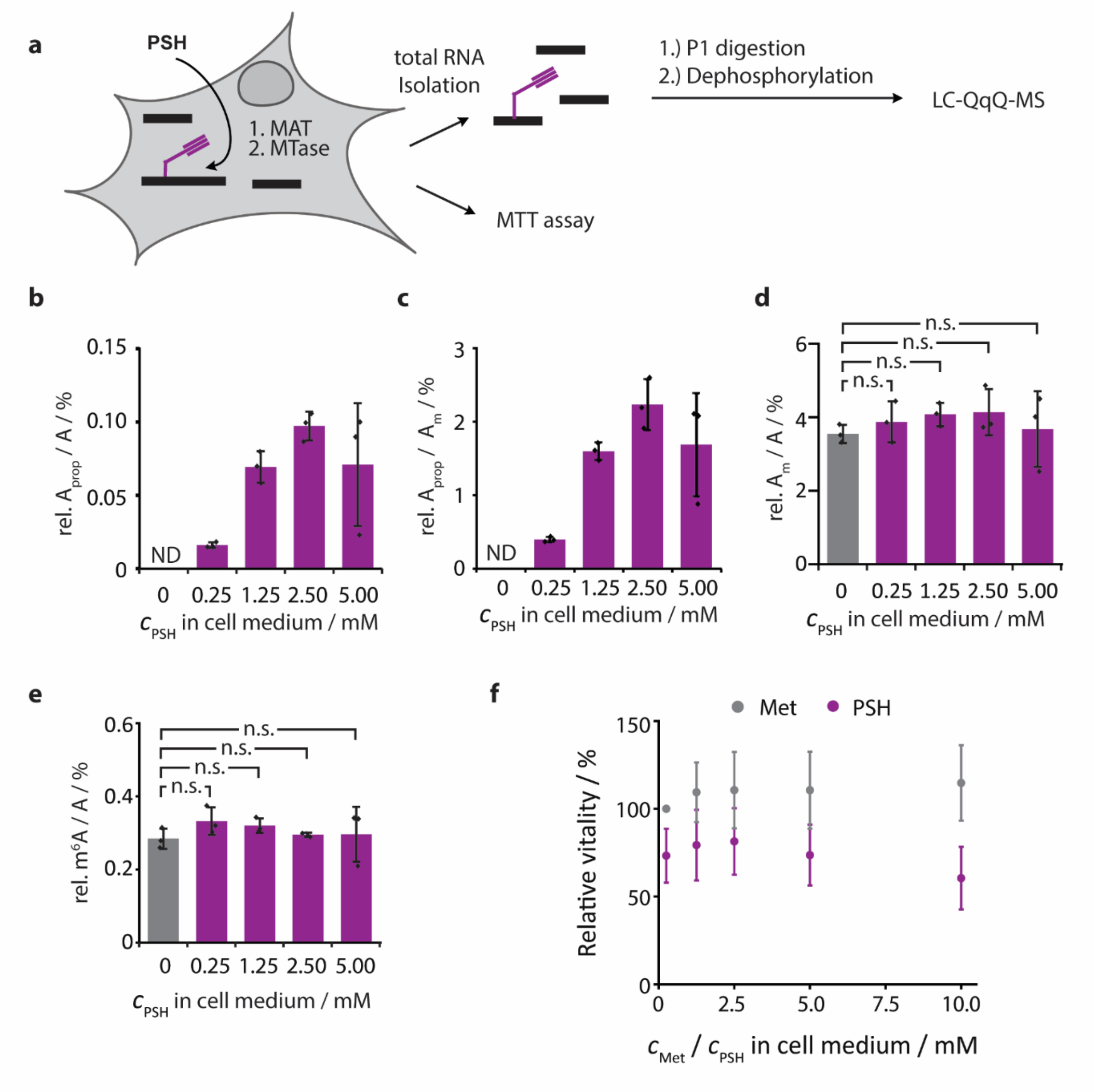

Supplementary Figure 1: **Optimizing feeding conditions for HeLa cells.**

a, Scheme b-e, Quantification of modified nucleotides in total RNA from HeLa cells treated with indicated concentrations of PSH. (b) A_{prop} relative to A, (c) A_{prop} relative to A_m, (d) A_m relative to A and (e) m^6A relative to A. Quantification from dynamic MRM run on LC-QqQ-MS using external synthetic standards. Not detected (ND) means no signal with correct quantifier detected. Mean values and SD from n=3 biological replicates are shown. Statistical significance determined via independent two-tailed t-test (n.s. P>0.05; * P≤0.05; ** P≤0.01; *** P≤0.001). Source data are provided as a Source Data file. f, Cell viability from MTT assay for HeLa cells treated with different amounts of PSH or methionine in cell media for 16 h. Vitality relative to cells in standard conditions (0.25 mM Met). SD from 3 biological replicates. Source data are provided as a Source Data file.

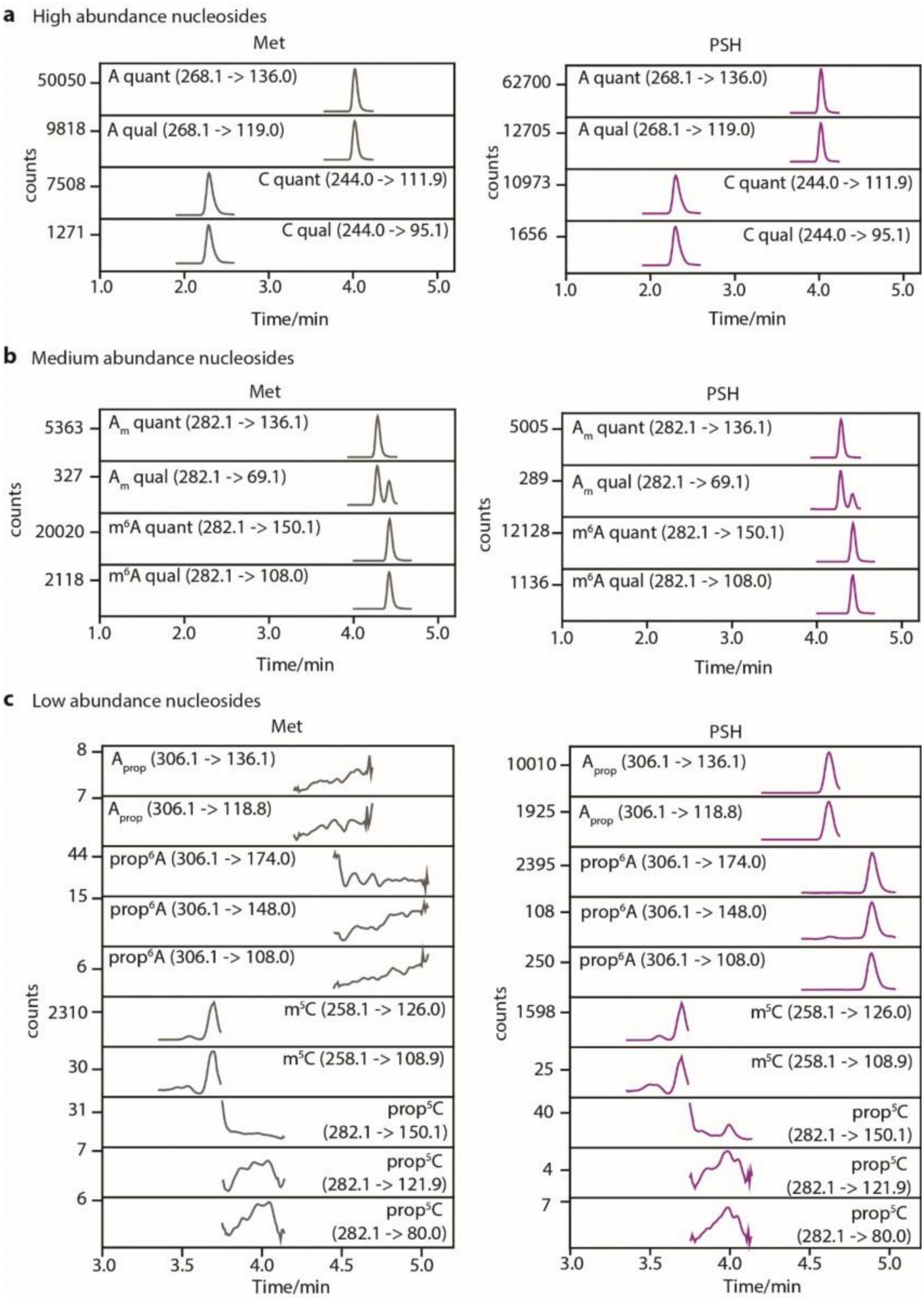

Supplementary Figure 2: **Detection and quantification of modified nucleosides in mRNA.**
a-c, Exemplary runs of +ESI dynamic MRM runs on LC-QqQ-MS to detect and quantify high abundant (a), medium abundant (b) and low abundant (c) modified nucleosides in digested, dephosphorylated, poly(A) enriched mRNA from HeLa cells metabolically labeled with 2.5 mM PSH (purple) or Met (gray) as control. Shown are the ion counts vs time for the retention time windows of the different analytes and indicated fragmentation.

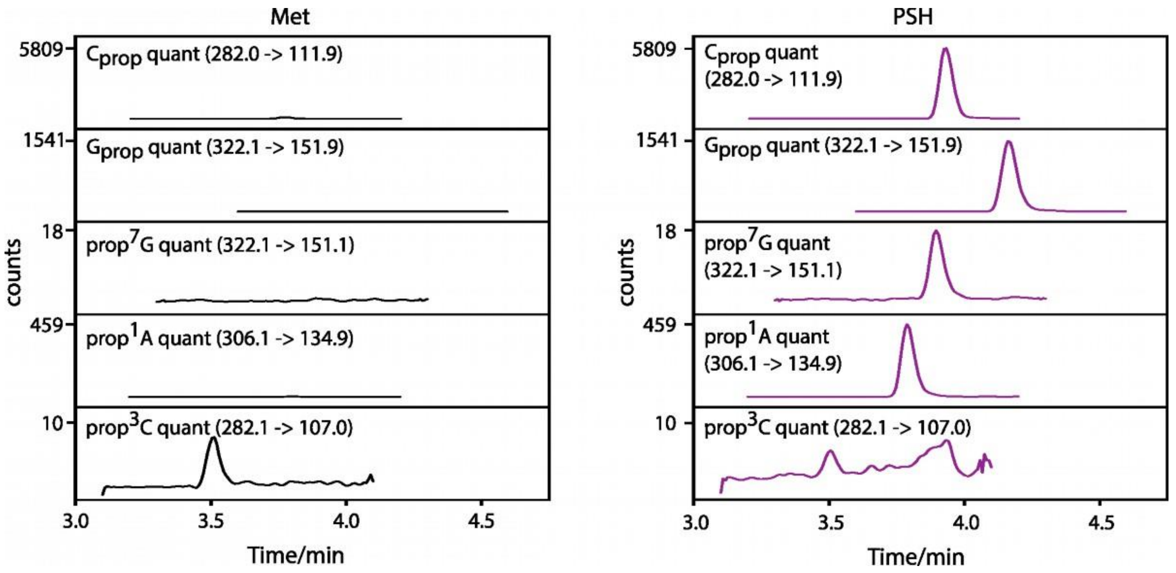

Supplementary Figure 3: **Detection and Quantification of modified nucleosides in mRNA.**
Exemplary runs of +ESI dynamic MRM runs on LC-QqQ-MS to detect and quantify low abundant modified nucleosides in digested, dephosphorylated, poly(A) enriched mRNA from HeLa cells metabolically labeled with 2.5 mM PSH (purple) or Met (gray) as control. Shown are the ion counts vs time for the retention time windows of the different analytes and indicated fragmentation.

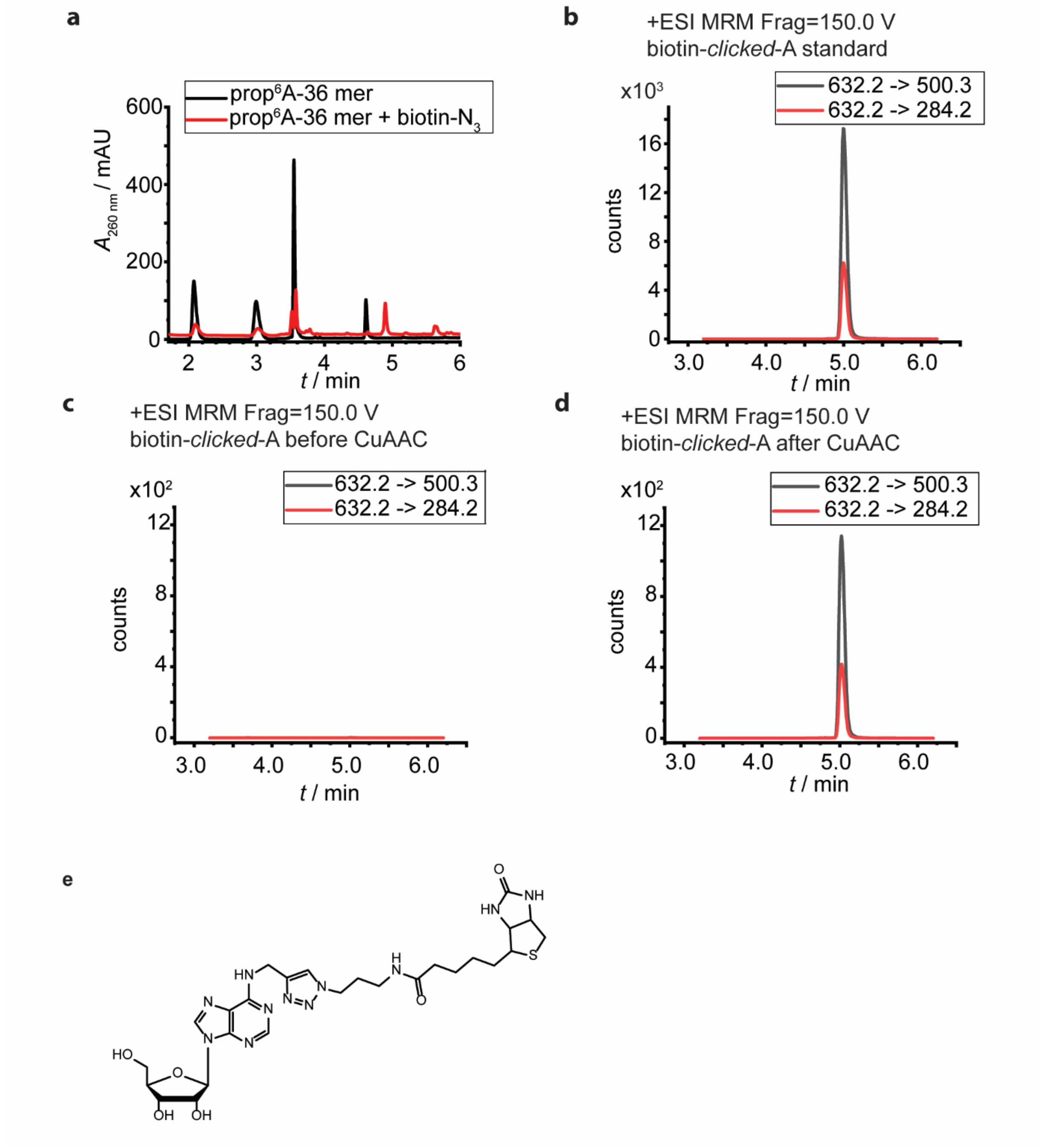

Supplementary Figure 4: **CuAAC efficiency.**

a, HPLC run of digested prop^{6}A-modified RNA (36mer) before and after CuAAC with biotin azide. b-d, LC-QqQ-MS verification of click-product formation. Fragmentation of N^6-biotinylated adenosine (b) was used to verify product formation after CuAAC (d) but not in control when omitting the biotin azide (c). e, Structure of biotin-*clicked*-A as product of CuAAC with prop^{6}A and biotin azide.

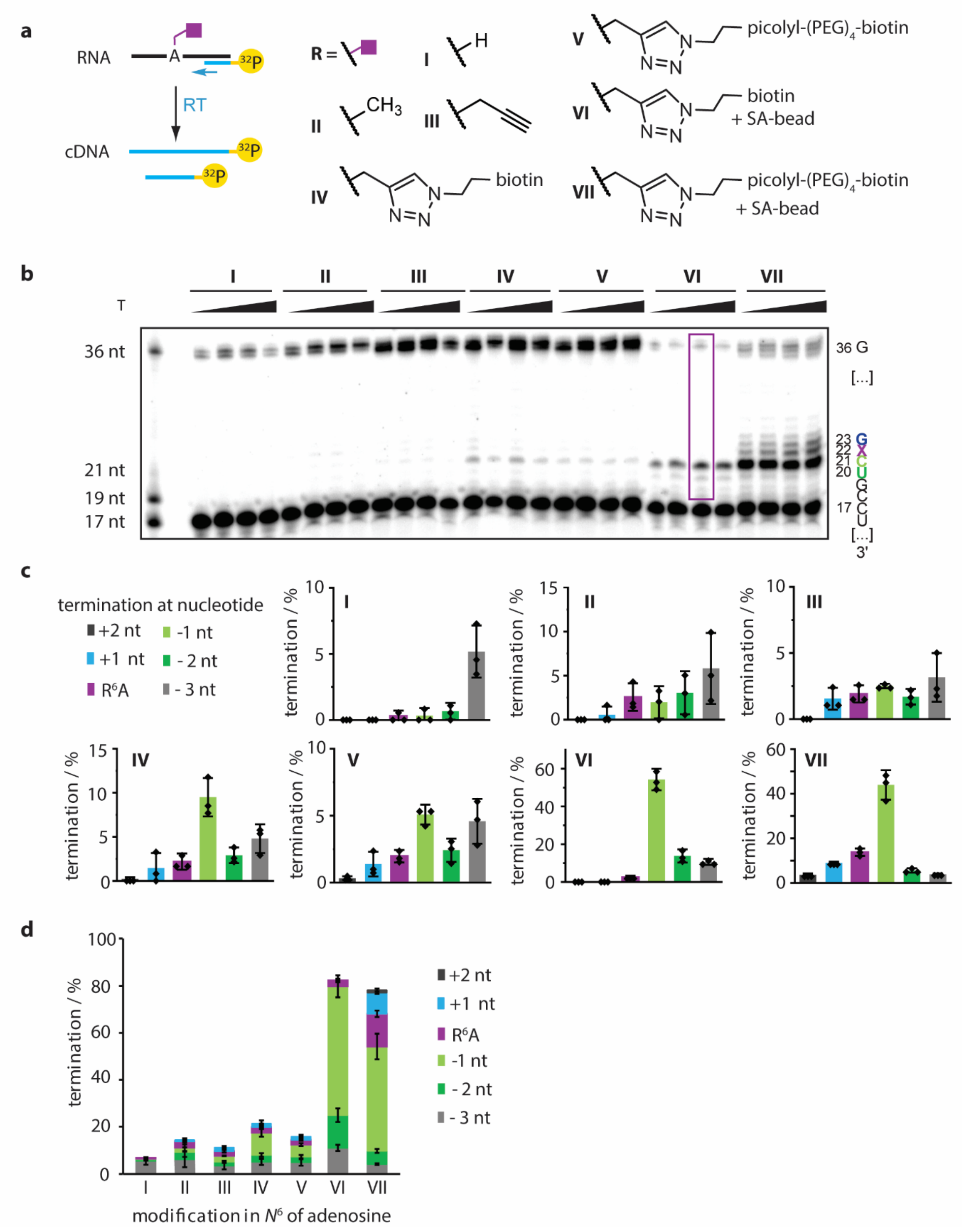

Supplementary Figure 5: **Optimizing termination during RT with SuperScript SSIV.**
a, Scheme of primer extension assay and N^6-modifications of A incorporated into the test RNA (marked with purple square (R)). b, Exemplary denat. PAGE (15 % PAA, 1× TBE) from primer extension assay with SS IV at different temperatures (37 °C, 45 °C, 50 °C and 57 °C, in increasing order indicated by triangles). Source data are provided as a Source Data file. c, Quantification of primer extension experiments. Occurring termination around the N^6-modified A (R^6A, purple square) was quantified for each respective position and each modified RNA (I-VII) from PAGE analyses and normalized to the total cDNA yield (≙ all bands within purple frame) (c). Source data are provided as a Source Data file. d, Stacked data from separate quantifications (c) to compare total termination occurring for indicated modifications. Purple frame indicates experimental conditions chosen for library preparation. Data and error bars represent mean value and SD of n=3 independent experiments. Source data are provided as a Source Data file.

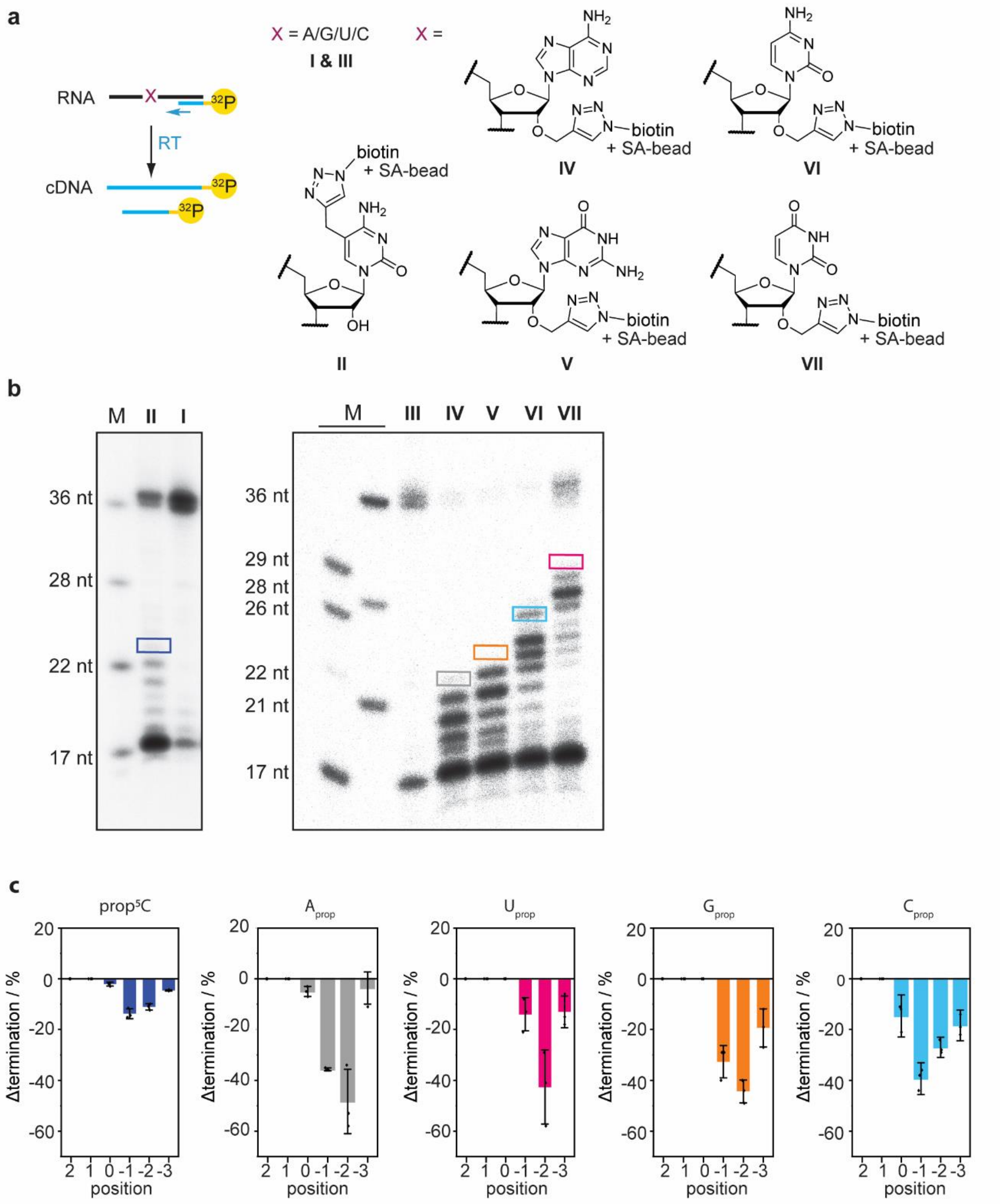

Supplementary Figure 6: **Terminations during RT of RNA templates with different modifications.**
a, Scheme of primer extension assay and (modified) nucleotides (X) that were incorporated into different 36 nt long test RNAs (see Supplementary Table 11). b, Exemplary denat. PAGEs (15 % denat. PAA, 1× TBE) from primer extension assays of RNAs with indicated modifications. Using unmodified RNAs (I & III) only signals for RT-primer (17 nt) and full-length product (36 nt) can be observed. For modified RNAs (II, IV-VII) additional signals can be observed, indicating termination during RT. Position of modified nucleotide is indicated by colored rectangle. Marker consists of synthetical DNA with the sequence of the respective cDNA. Source data are provided as a Source Data file. c, Quantification of primer extension experiments showing the modification specific termination signatures for prop^{5}C, A_{prop}, U_{prop}, G_{prop} and C_{prop}. Occurring terminations around the modifications were quantified for each respective position and each modified RNA from PAGE analysis and normalized to the total cDNA yield. Data and error bars represent mean value and SD of n=3 independent experiments. Source data are provided as a Source Data file.

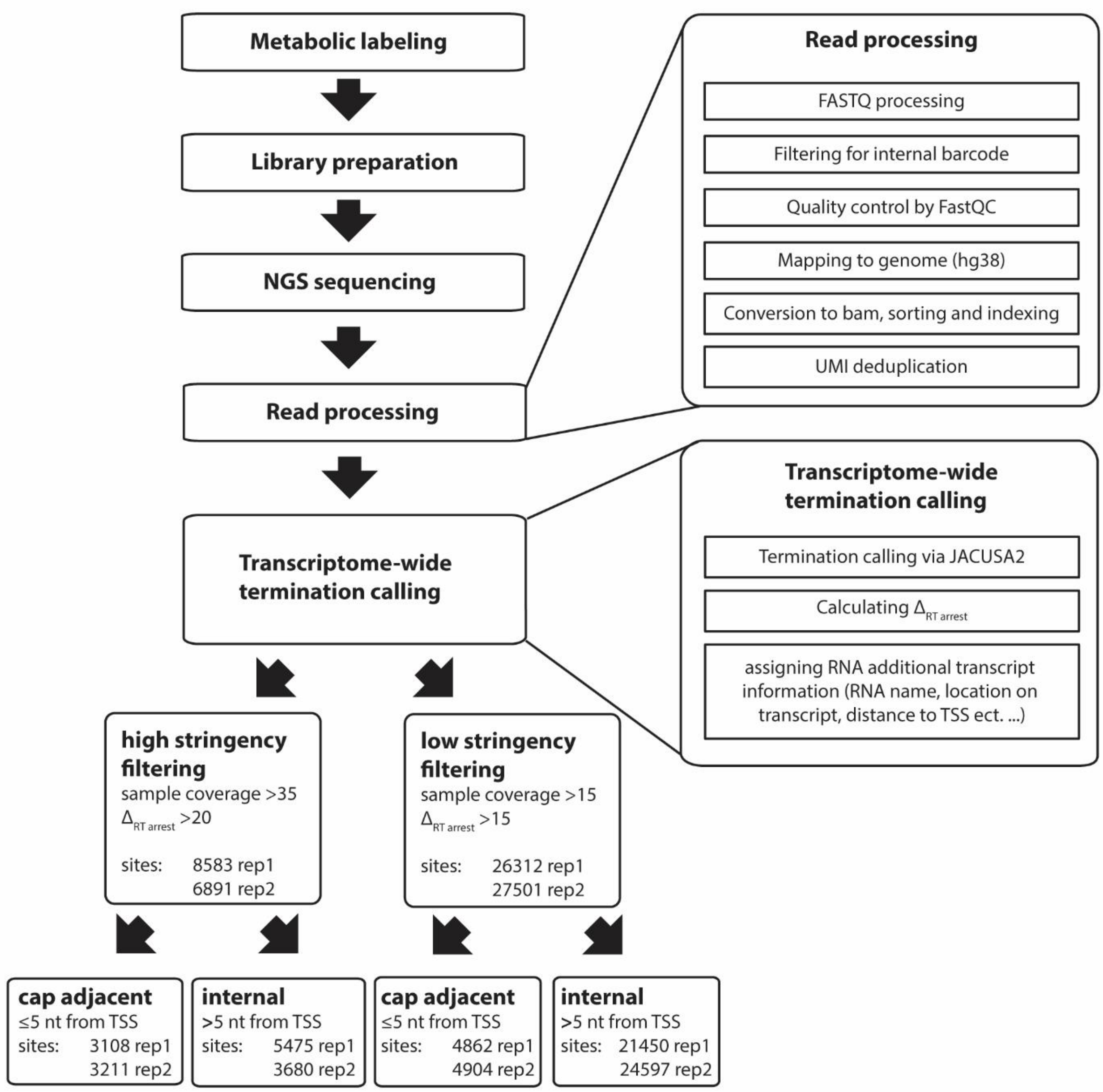

Supplementary Figure 7: **Scheme of bioinformatic steps performed for MePMe-seq and filter settings applied to the data sets.**

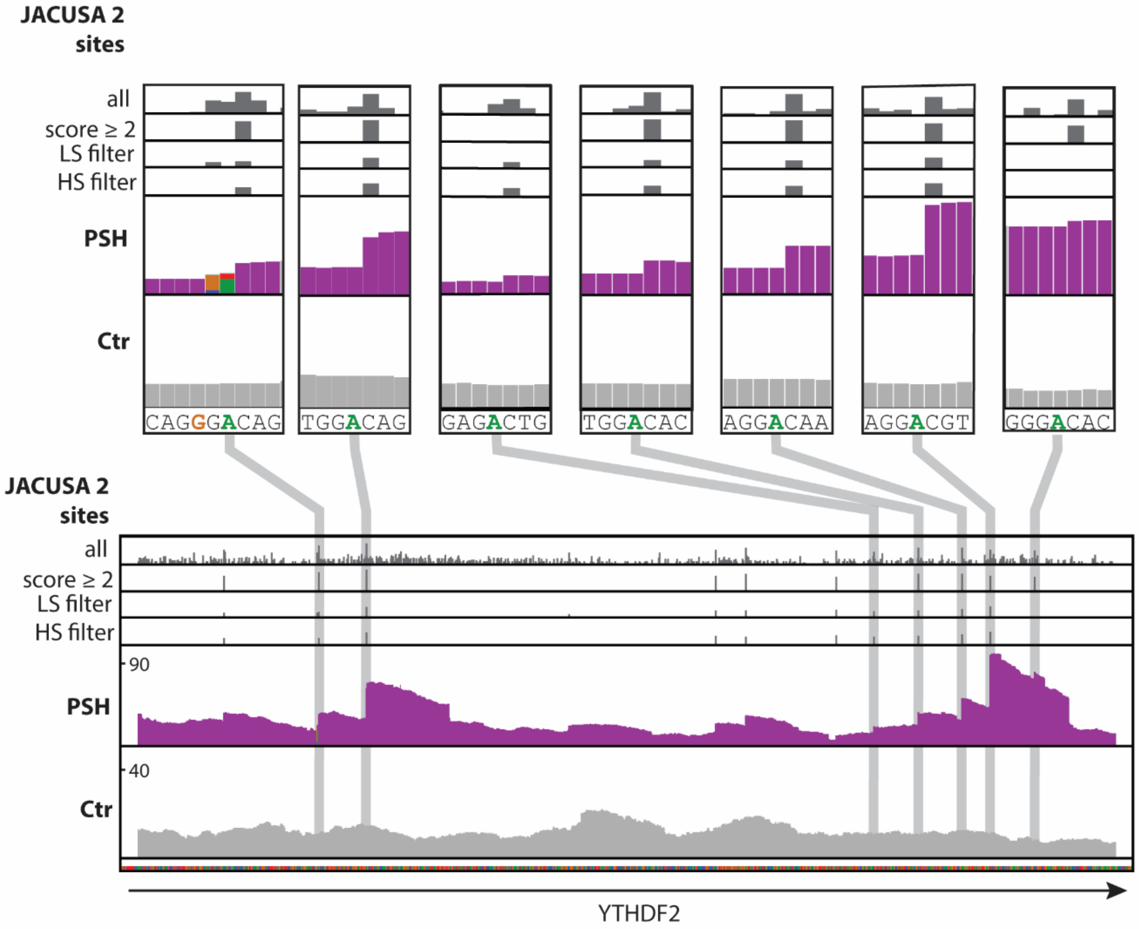

Supplementary Figure 8: **IGV browser coverage tracks of MePMe-seq data for YTHDF2 mRNA.**
Cells labeled with PSH (purple) or methionine as control (gray). Gray bars represent positions called by JACUSA2 as terminations under the indicated filtering conditions. Arrow indicates orientation of coding strand. One representative example of 2 independent experiments is shown.

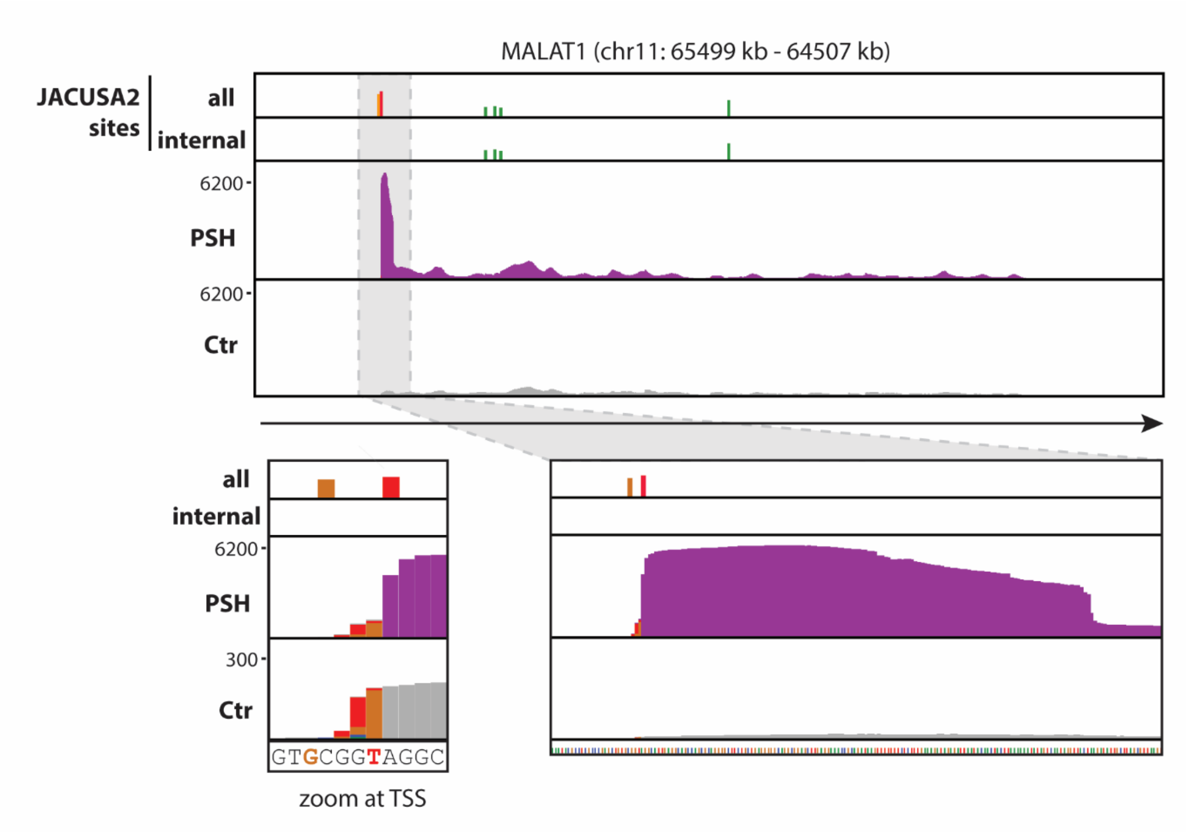

Supplementary Figure 9: **Enrichment and terminations at 5'end of transcripts.**
IGV browser coverage tracks of MePMe-seq data for MALAT1 RNA from cells labeled with PSH (purple) or methionine as control (gray). Colored bars represent terminations (red=T, orange=G, green=A,) identified by JACUSA2 hits (HS filtered). Arrow indicates orientation of coding strand.

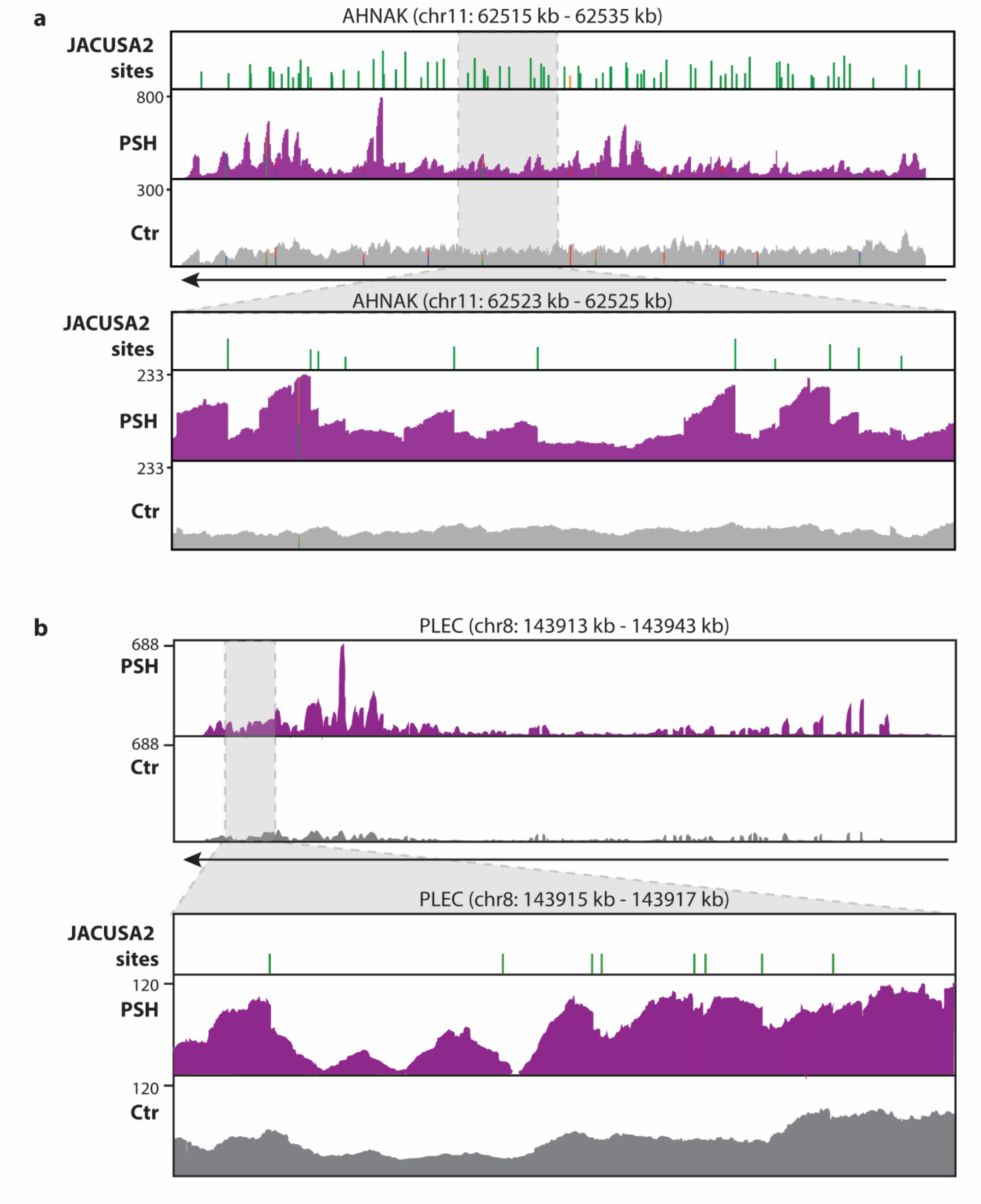

Supplementary Figure 10: **Clustering m^{6}A sites identified via MePMe-seq.**
IGV browser coverage tracks of MePMe-seq data for (a) AHNAK and (b) PLEC mRNA from cells labeled with PSH (purple) or methionine as control (gray). Colored bars represent terminations (orange=G, green=A,) identified by JACUSA2 hits (HS filtered). Arrow indicates orientation of coding strand. One representative example of 2 independent experiments is shown.

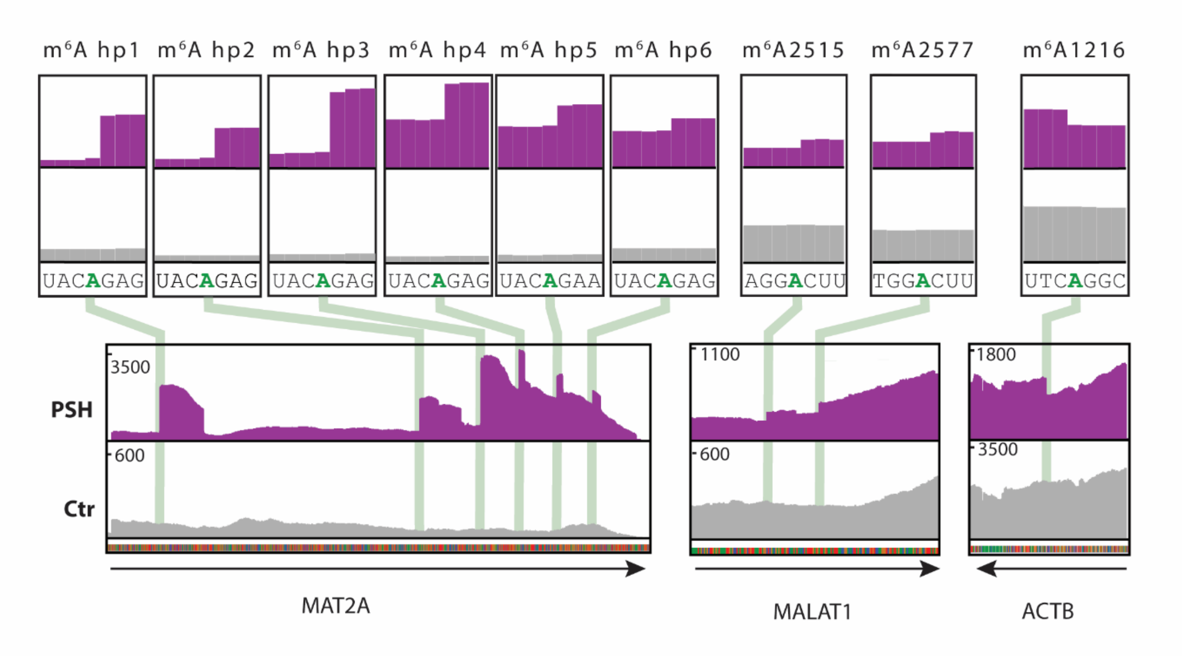

Supplementary Figure 11: **Integrative genomics viewer (IGV) browser coverage tracks of MePMe-seq data for the MAT2A, MALAT1 and ACTB RNAs.**
Cells labeled with PSH (purple) or methionine as control (gray). Arrow indicates orientation of coding strand. One representative example of 2 independent experiments is shown.

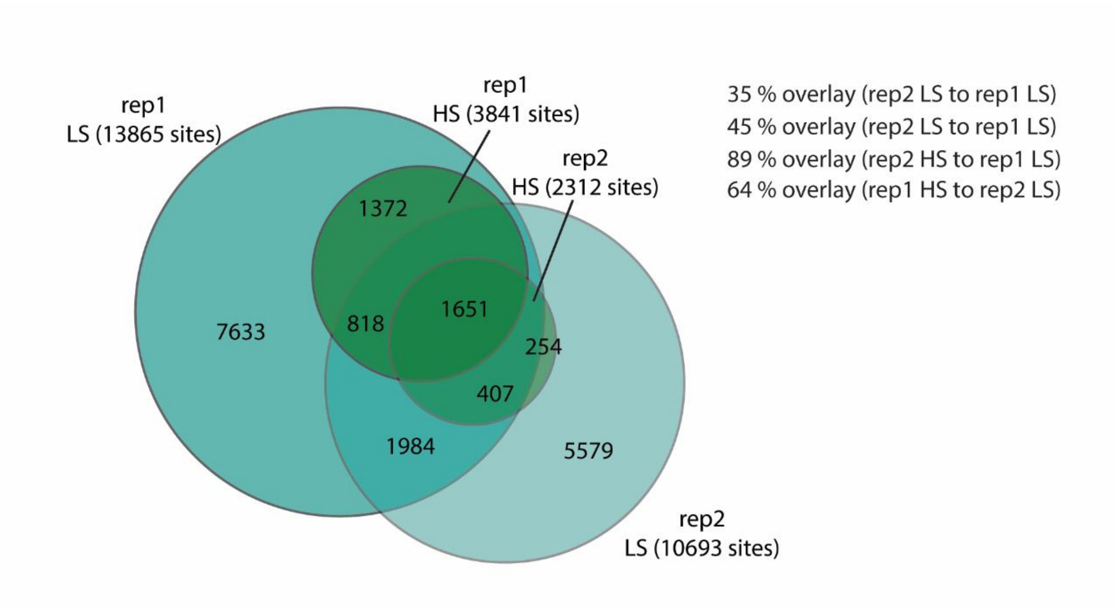

Supplementary Figure 12: **Overlap between identified m^{6}A sites.**
Two MePMe-seq experiments for HS and LS filter settings (% calculated as indicated).

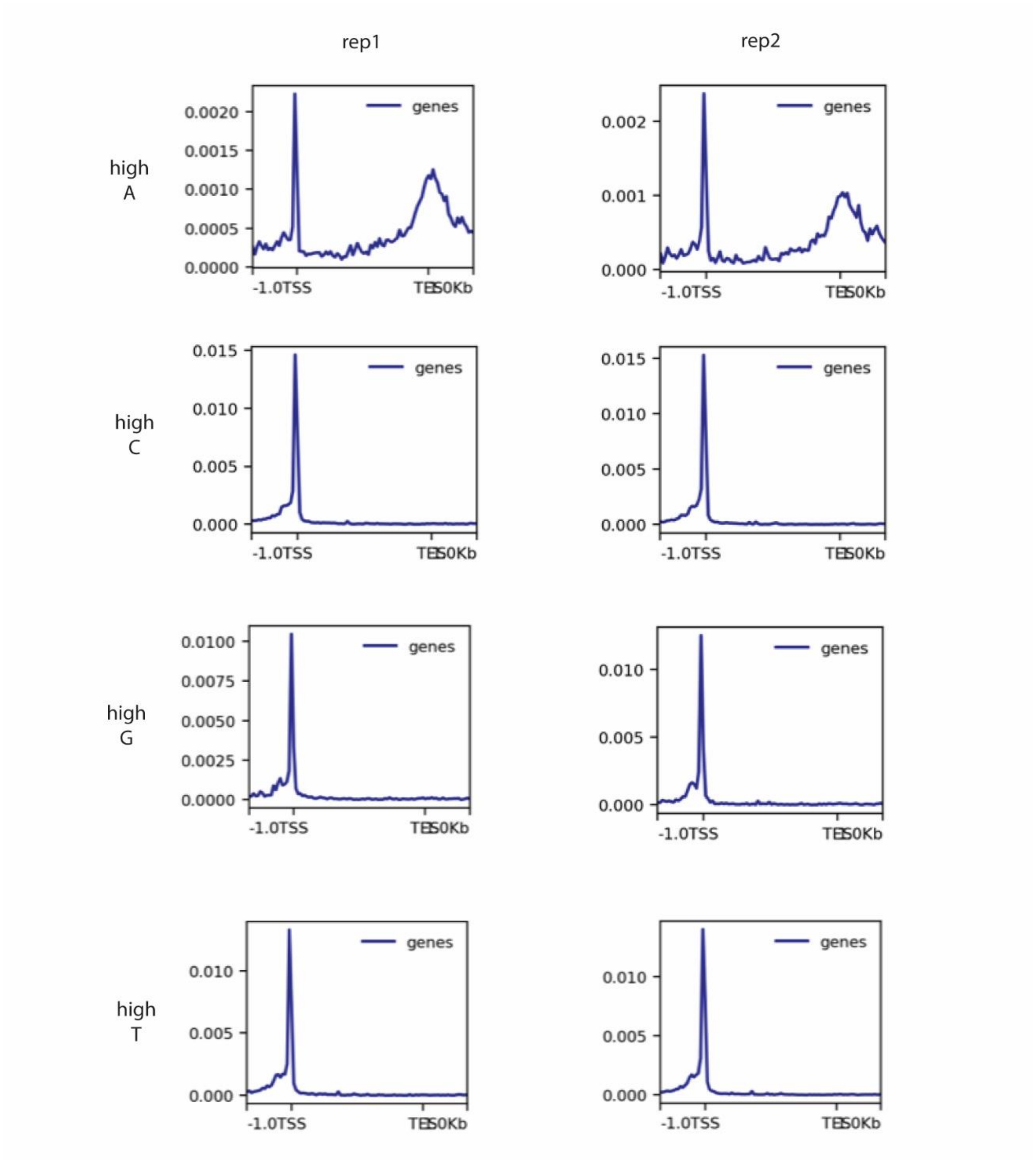

Supplementary Figure 13: **Clustering of JACUSA2 hits around the TSS.**
Frequency of JACUSA2 hits plotted against their position on the transcript showing clustering ~5 nt around the TSS for terminations at all nucleoside identities in both replicates.

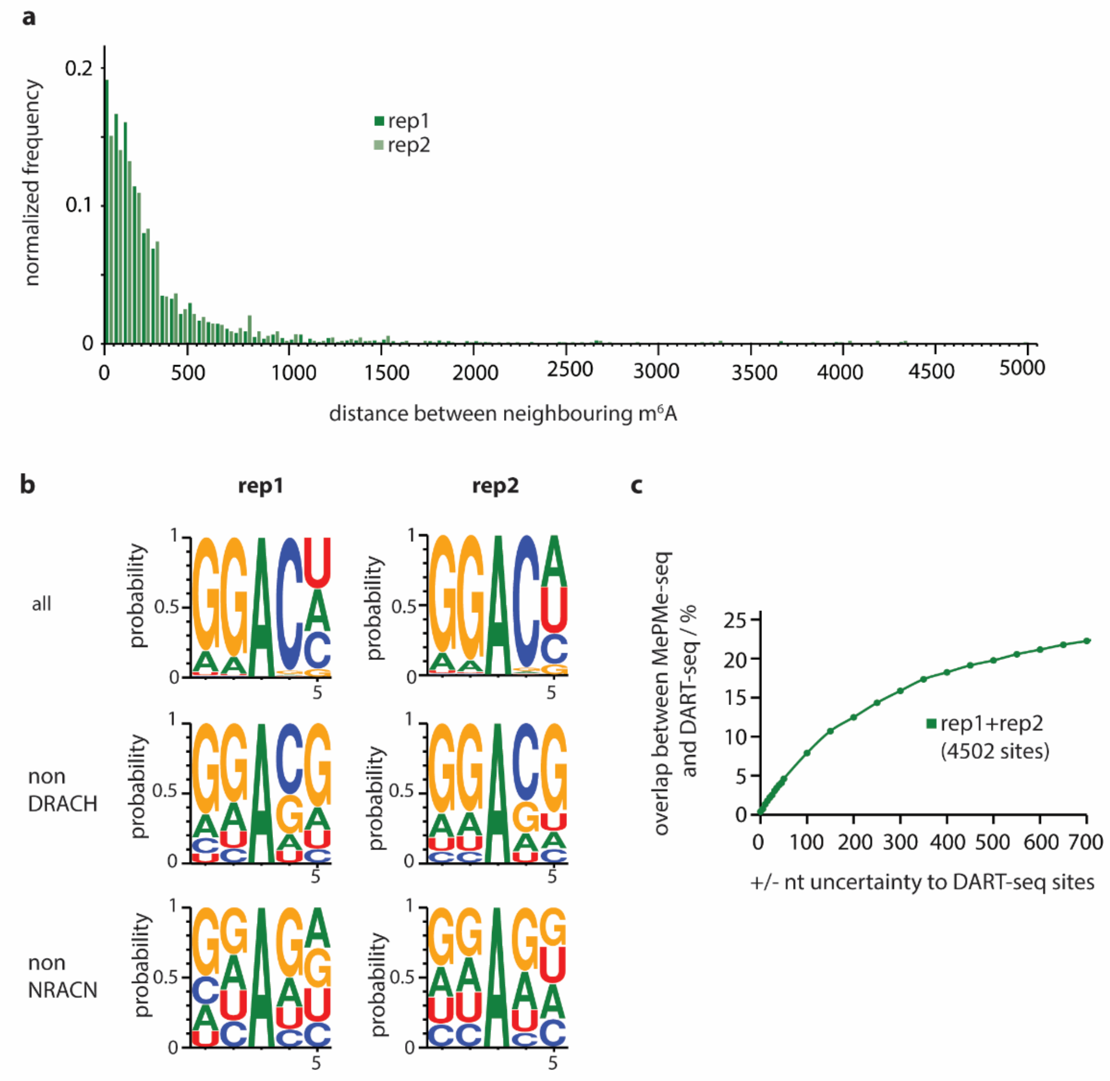

Supplementary Figure 14: **Analysis of identified m^{6}A sites in mRNA.**
a, Frequency of distance between neighboring m^{6}As. Located on the same transcript, shown cutoff at distances >5000 nt. b, Consensus motif for sequences surrounding identified m^{6}A (HS filtering) if all 5 mers are considered, if DRACH sequences are excluded and if NRACN sequences are excluded. c, Overlap between m^{6}A sites identified by MePMe-seq and m^{6}A sites identified by DART-seq when uncertainty region is allowed. n=2 biologically independent experiments are shown.

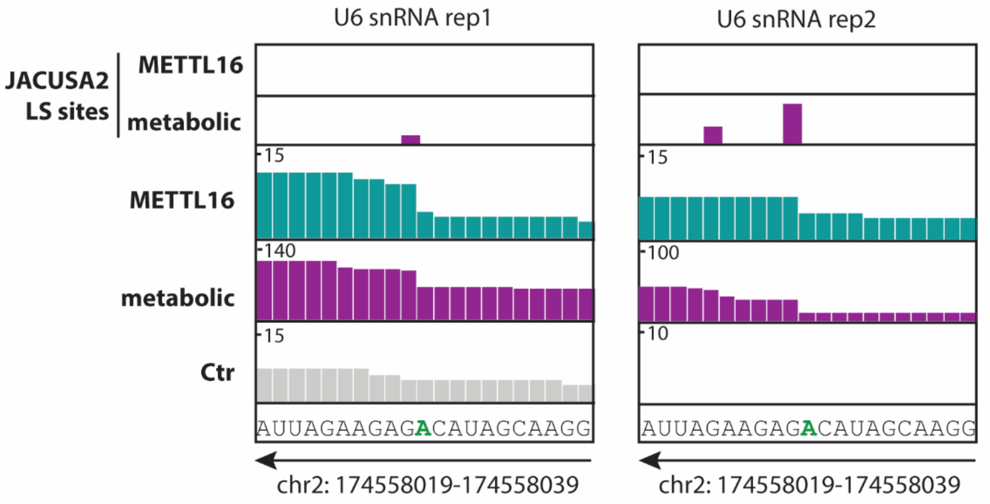

Supplementary Figure 15: **IGV browser coverage tracks of U6 snRNA.**
Combined data from RNA labeled with METTL16 (turquoise), metabolic feeding (purple) or control (gray). Arrow indicates orientation of coding strand. 2 biologically independent experiments are shown.

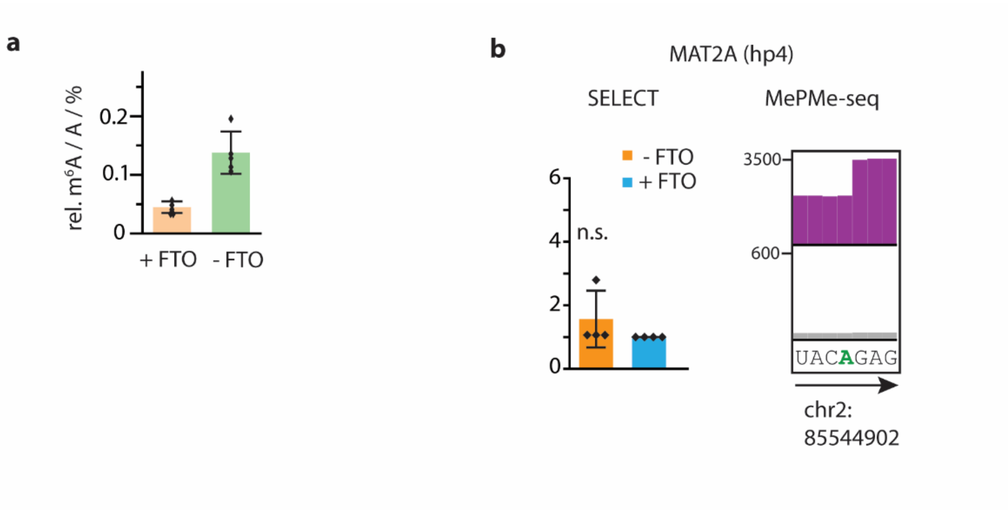

Supplementary Figure 16: **Additional SELECT results.**
a, Demethylation of poly(A)-isolated RNA for SELECT assay using FTO. Mean values and SD from n=5 biological replicates are shown. Source data are provided as a Source Data file. b, The normalized ΔC_q values of SELECT qPCR measurements for reported m^6A in hp4 of MAT2A. Mean values and SD from n=4 biological replicates are shown. Statistical significance determined via one-sample one-tailed t-test (n.s. P>0.05; * P≤0.05; ** P≤0.01; *** P≤0.001). Source data are provided as a Source Data file.

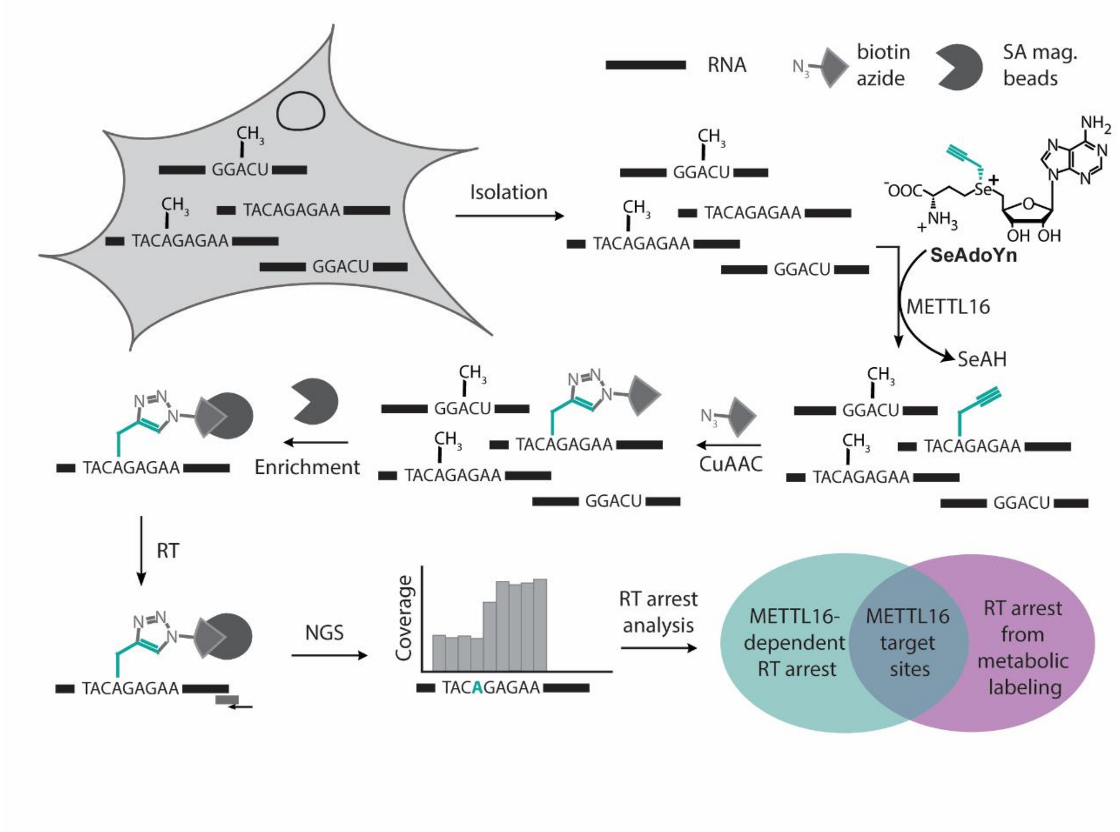

Supplementary Figure 17: **Scheme of METTL16 dependent labeling combined with metabolic labeling to identify METTL16 targets.**

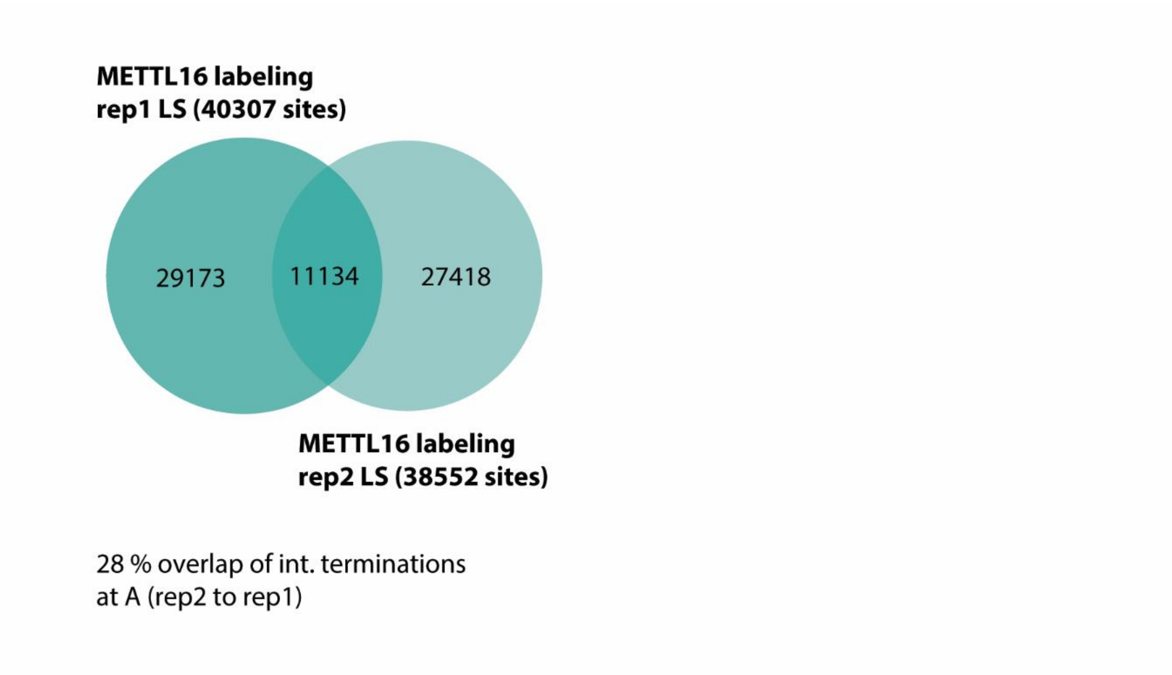

Supplementary Figure 18: **Overlap between two METTL16 labeling experiments after LS filtering.**

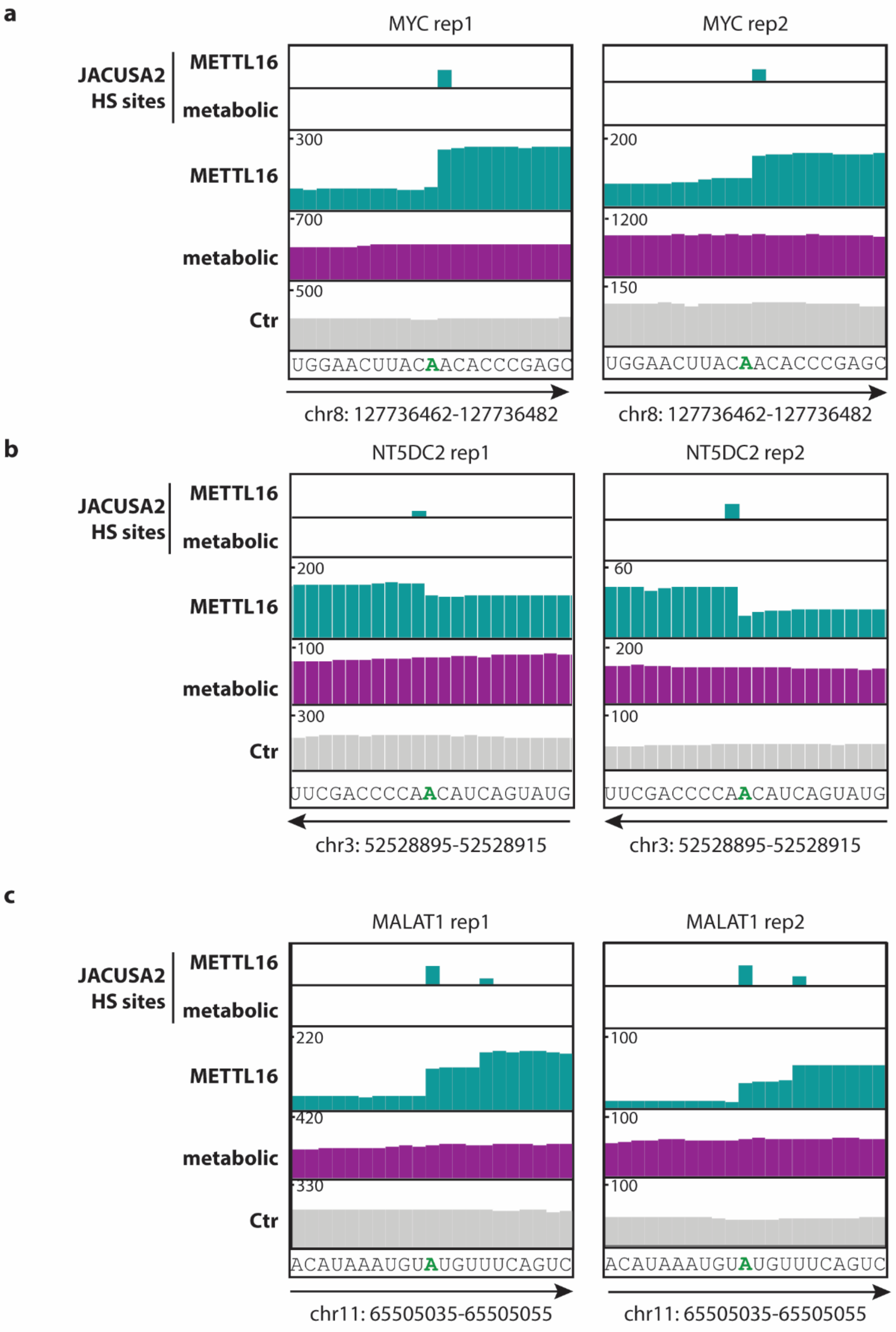

Supplementary Figure 19: **IGV browser coverage tracks of combined data for exemplary RNAs.** (a) MYCU6, (b) NT5DC2 and (c) MALAT1 from RNA labeled with METTL16 (turquoise), metabolic feeding (purple) or control (gray). Arrow indicates orientation of coding strand. 2 biologically independent experiments are shown.

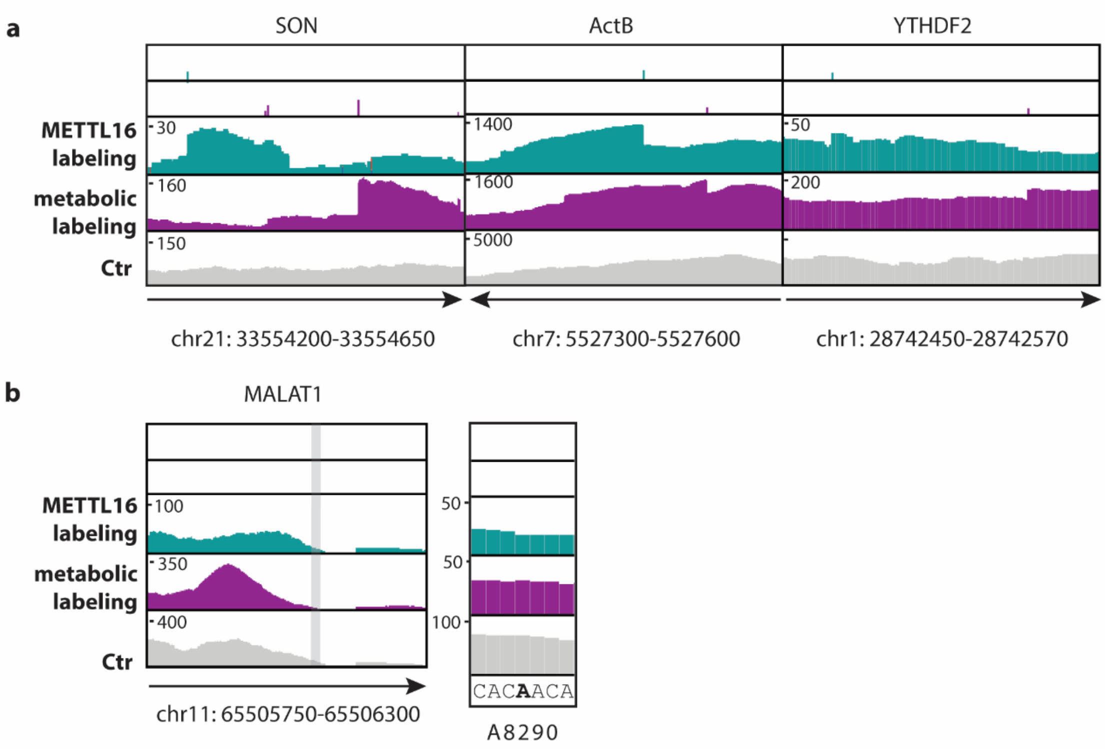

Supplementary Figure 20: **IGV browser coverage tracks of combined METTL16 and metabolic labeling data for exemplary RNAs.**

Indicated mRNA from RNA labeled with METTL16 (turquoise), metabolic feeding (purple) or control (gray). a, Exemplary sites having METTL16 and metabolic hits in the same transcript but different positions. b, Suspected METTL16 target site A8290 from MALAT1 showing no hit with METTL16 or metabolic feeding. Arrow indicates orientation of coding strand. One representative example of 2 independent experiments is shown.

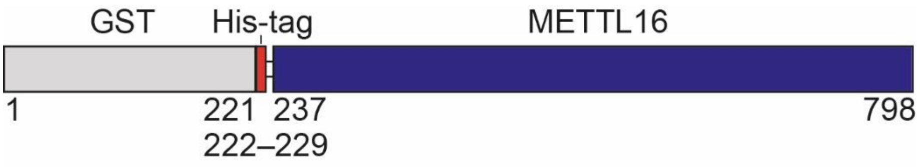

10 20 30 40 50 60
MSPILGYWKI KGLVQPTRLL LEYLEEKYEE HLYERDEGDK WRNKKFELGL EFPNLPYYID

70 80 90 100 110 120
GDVKLTQSMA IIRYIADKHN MLGGCPKERA EISMLEGAVL DIRYGVSRIA YSKDFETLKV

130 140 150 160 170 180
DFLSKLPEML KMFEDRLCHK TYLNGDHVTH PDFMLYDALD VVLYMDPMCL DAFPKLVCFK

190 200 210 220 230 240
KRIEAIPQID KYLKSSKYIA WPLQGWQATF GGGDHPPKSG SHHHHHHHHE NLYFQGMALS

250 260 270 280 290 300
KSMHARNRYK DKPPDFAYLA SKYPDFKQHV QINLNGRVSL NFKDPEAVRA LTCTLLREDF

310 320 330 340 350 360
GLSIDIPLER LIPTVPLRLN YIHWVEDLIG HQDSDKSTLR RGIDIGTGAS CIYPLLGATL

370 380 390 400 410 420
NGWYFLATEV DDMCFNYAKK NVEQNNLSDL IKVVKVPQKT LLMDALKEES EIIYDFCMCN

430 440 450 460 470 480
PPFFANQLEA KGVNSRNPRR PPPSSVNTGG ITEIMAEGGE LEFVKRIIHD SLQLKKRLRW

490 500 510 520 530 540
YSCMLGKKCS LAPLKEELRI QGVPKVTYTE FCQGRTMRWA LAWSFYDDVT VPSPPSKRRK

550 560 570 580 590 600
LEKPRKPITF VVLASVMKEL SLKASPLRSE TAEGIVVVTT WIEKILTDLK VQHKRVPCGK

610 620 630 640 650 660
EEVSLFLTAI ENSWIHLRRK KRERVRQLRE VPRAPEDVIQ ALEEKKPTPK ESGNSQELAR

670 680 690 700 710 720
GPQERTPCGP ALREGEAAAV EGPCPSQESL SQEENPEPTE DERSEEKGGV EVLESCQGSS

730 740 750 760 770 780
NGAQDQEASE QFGSPVAERG KRLPGVAGQY LFKCLINVKK EVDDALVEMH WVEGQNRDLM

790
NQLCTYIRNQ IFRLVAVN

Supplementary Figure 21: **Depiction of the METTL16-construct used in this work.**

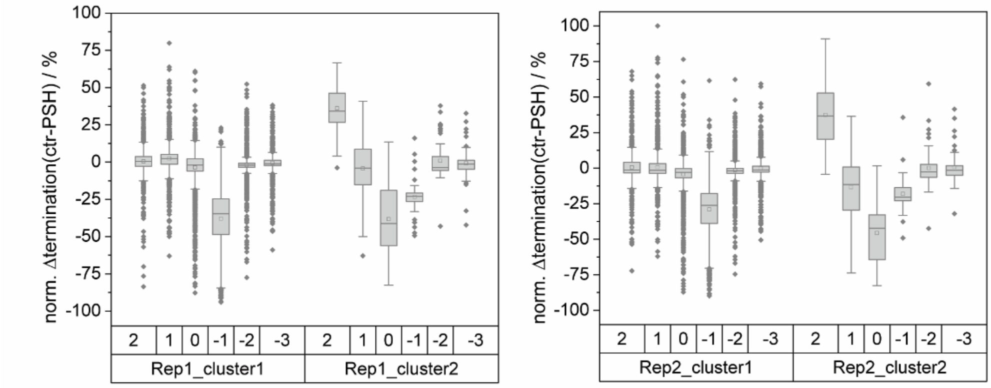

Supplementary Figure 22: **Cluster analysis on the termination pattern around terminations next to A in mRNA.**

A clustering of the bioinformatically determined termination signatures which are based on the coverage (Diff ctr-PSH) at the positions –2, –1, 0, 1, 2 around as modified identified As is shown in the box plot diagrams. Box plots after cutting the dendrogram from hierarchical clustering of all terminations next to A in mRNA (before filtering in internal and cap adjacent) using Euclidean distance into two clusters. In replicate 1 3,984 sites (98.8 %) and 2,443 sites in replicate 2 (98.7 %) were assigned to cluster 1 and only 48 sites in replicate 1 (1.2 %) and 32 sites in replicate 2 (1.3 %) were assigned to cluster2. In the boxplots the center lines, medians, upper and lower quartiles, whiskers (1.5×) and outliers are shown. Source data are provided as a Source Data file.

.

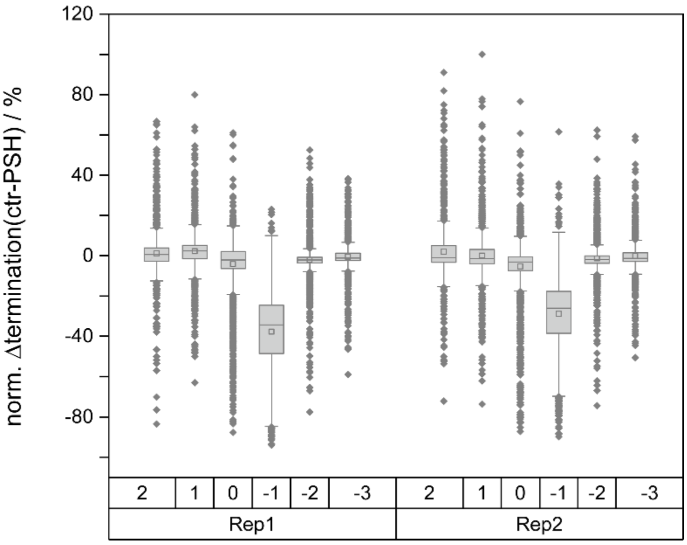

Supplementary Figure 23: **Termination pattern around all identified modified A sites in mRNA.**
Box plot of termination signatures which are based on the coverage (Diff ctr-PSH) at the positions –2, –1, 0, 1, 2 of all terminations that were assigned as happening -1 nt to an A in mRNA. In the boxplots of both replicates the center lines, medians, upper and lower quartiles, whiskers (1.5×) and outliers are shown. Source data are provided as a Source Data file.

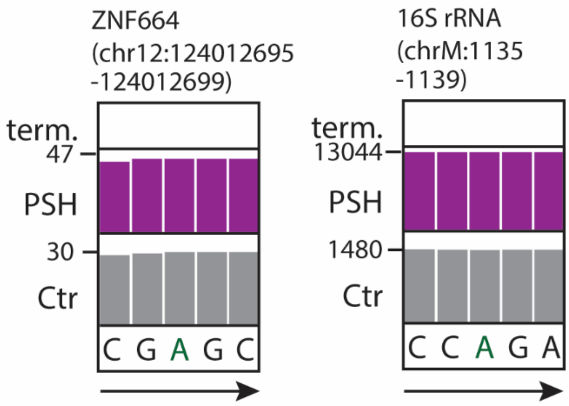

Supplementary Figure 24: **IGV browser coverage tracks of potential m^1A sites in cytosolic ZNF664 mRNA and mitochondrial 16S rRNA.**

Metabolic labeling (purple) and control (grey) showing both no termination at the potential m^1A sites (green, exemplary for rep1).

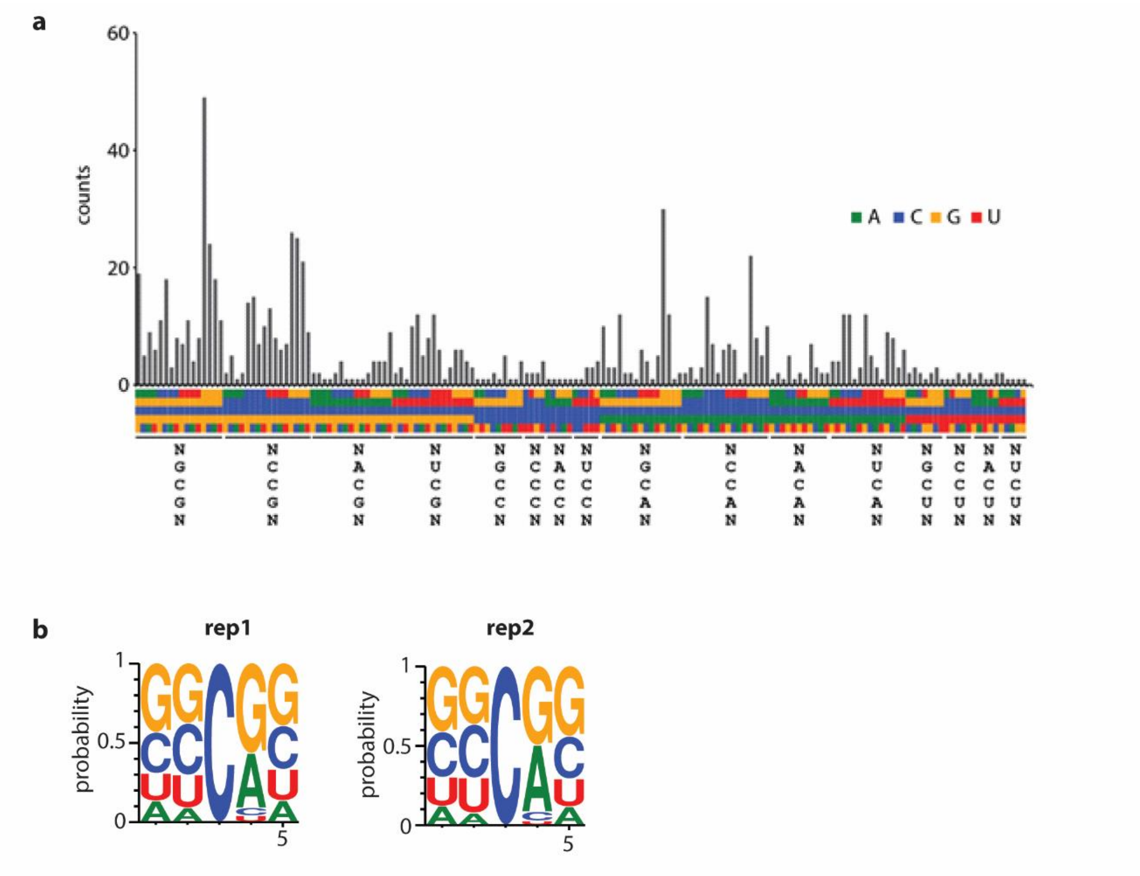

Supplementary Figure 25: **Consensus motifs for modified C.**
a, Counts per sequence motif surrounding identified m^5C sites (HS filtering), sorted by consensus motif. b, Consensus motif for 5mer sequences surrounding identified terminations next to C when filtering with HS filtering. Two independent experiments are shown.

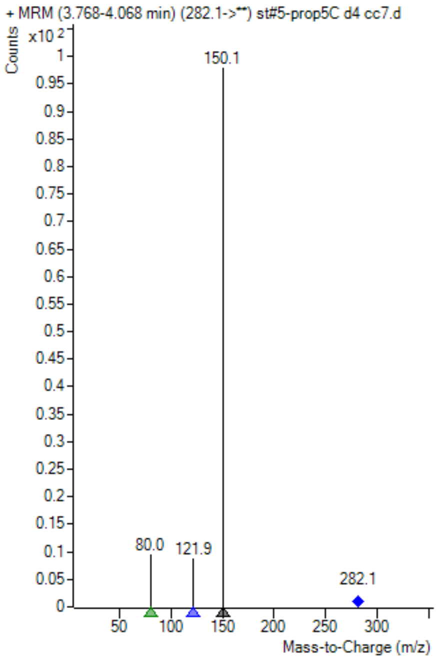

Supplementary Figure 26: **The signal intensity comparison of the MRM transition ions of prop^{5}C.** The MRM 282.1 → 150.1 (quantifier) is much more abundant than the MRM 282.1 → 121.9 and 282.1 → 80.0 (qualifiers). The signal is from highest point (cc7) of calibration curve (10 µL injection = 13.9 pg of prop^{5}C).

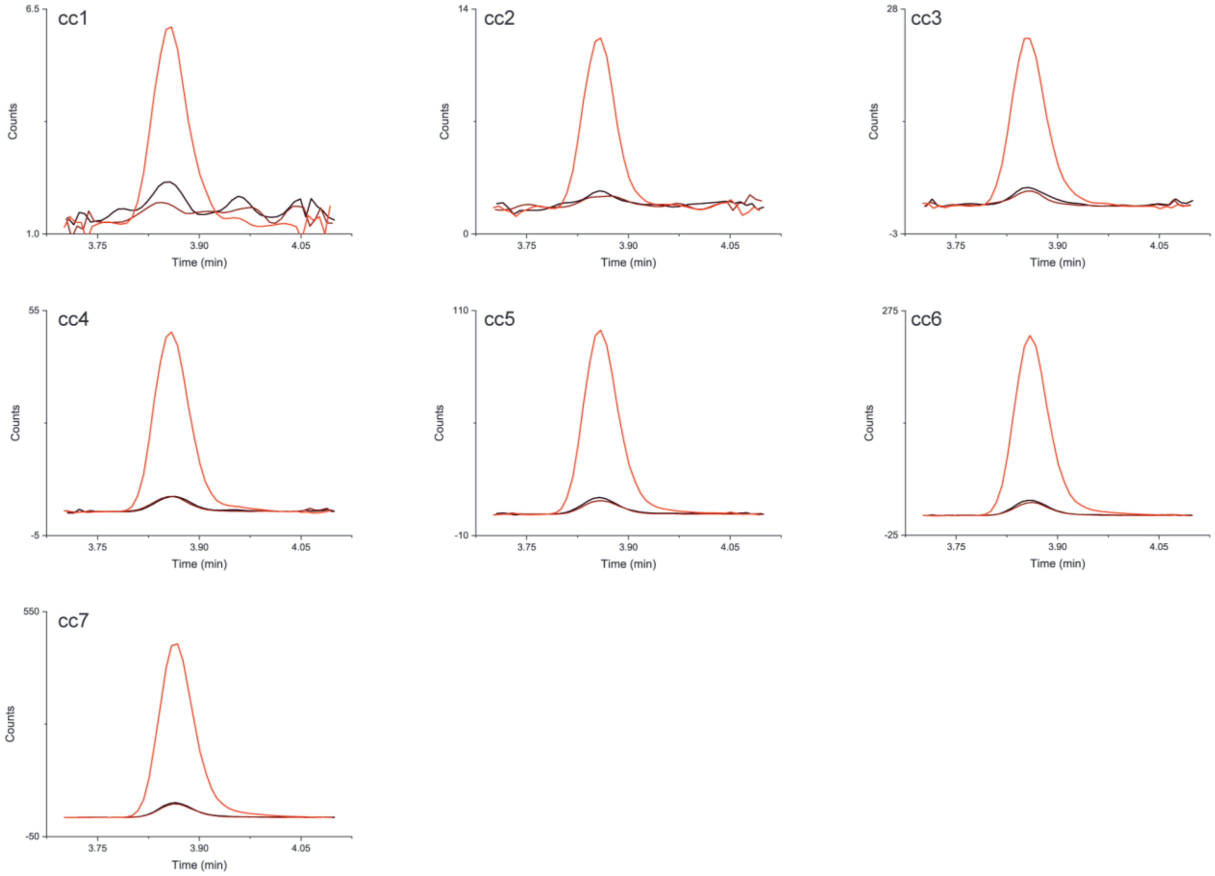

Supplementary Figure 27: **The representation of all points of calibration (cc1 to cc7) curve of prop^{5}C.** Quantifier (red line) and 2 qualifiers (brown and black lines).

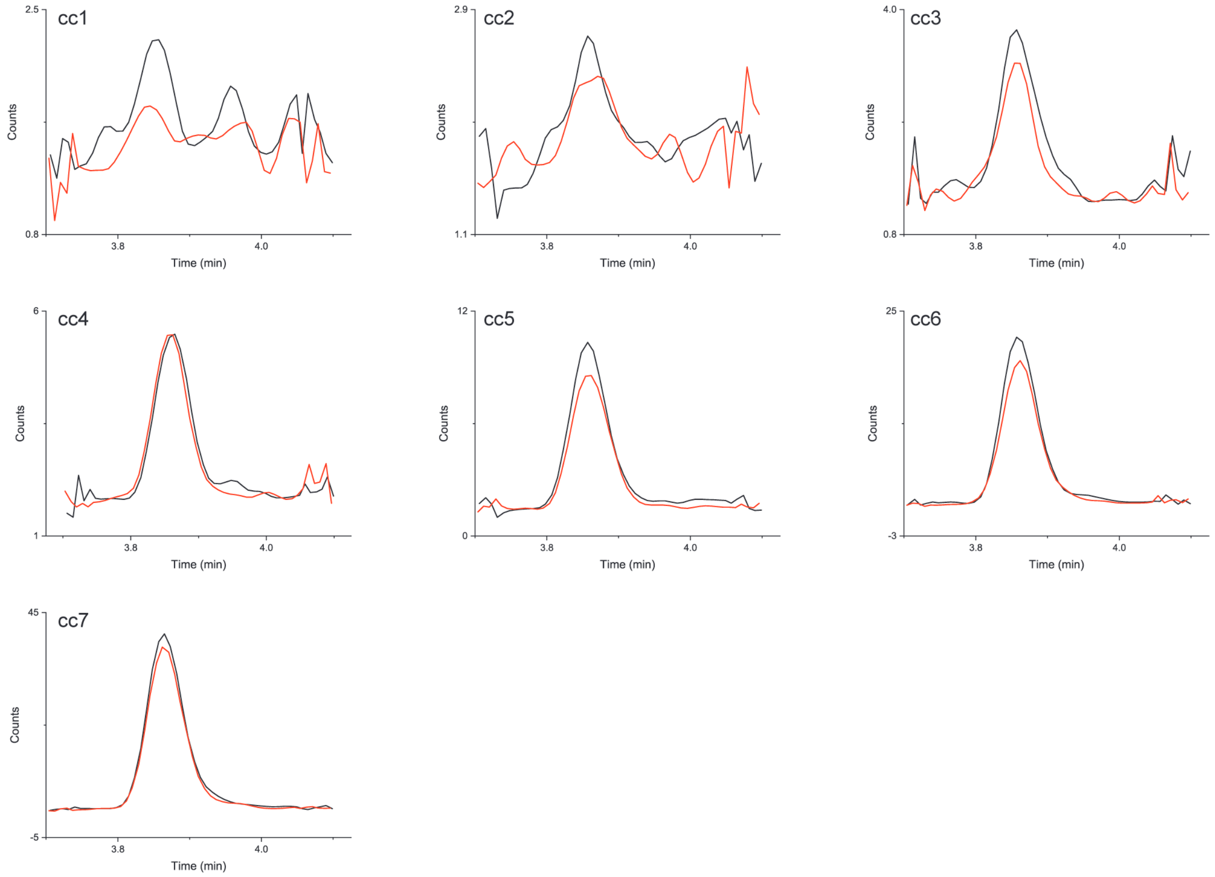

Supplementary Figure 28: **The representation of all points of calibration (cc1 to cc7) curve of prop^{5}C.** Comparison of 2 qualifiers. The qualifiers are represented by MRM 282.1 → 80.0 (black line) and 282.1 → 121.9 (red line). The figure shows the collapse of qualifier peaks between points 3 (cc3) and 2 (cc2) of calibration curve.

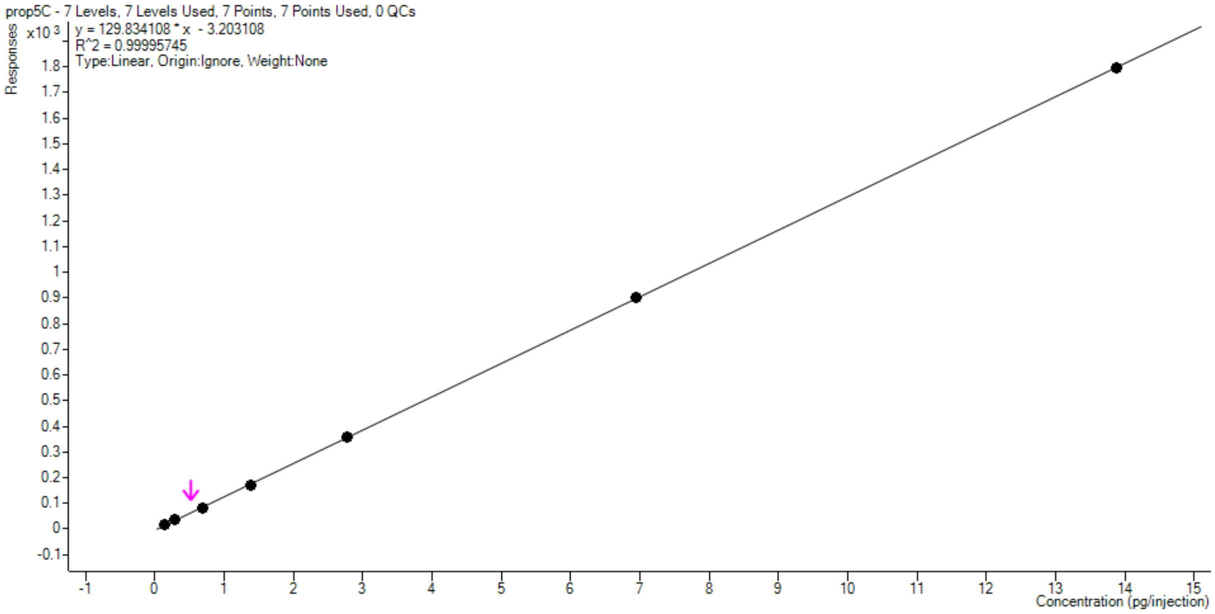

Supplementary Figure 29: **The calibration curve of prop^{5}C standard** (concentration = 4.9 nM = 1.39 pg/µL). The concentration level of prop^{5}C in the real sample is indicated by the pink arrow.

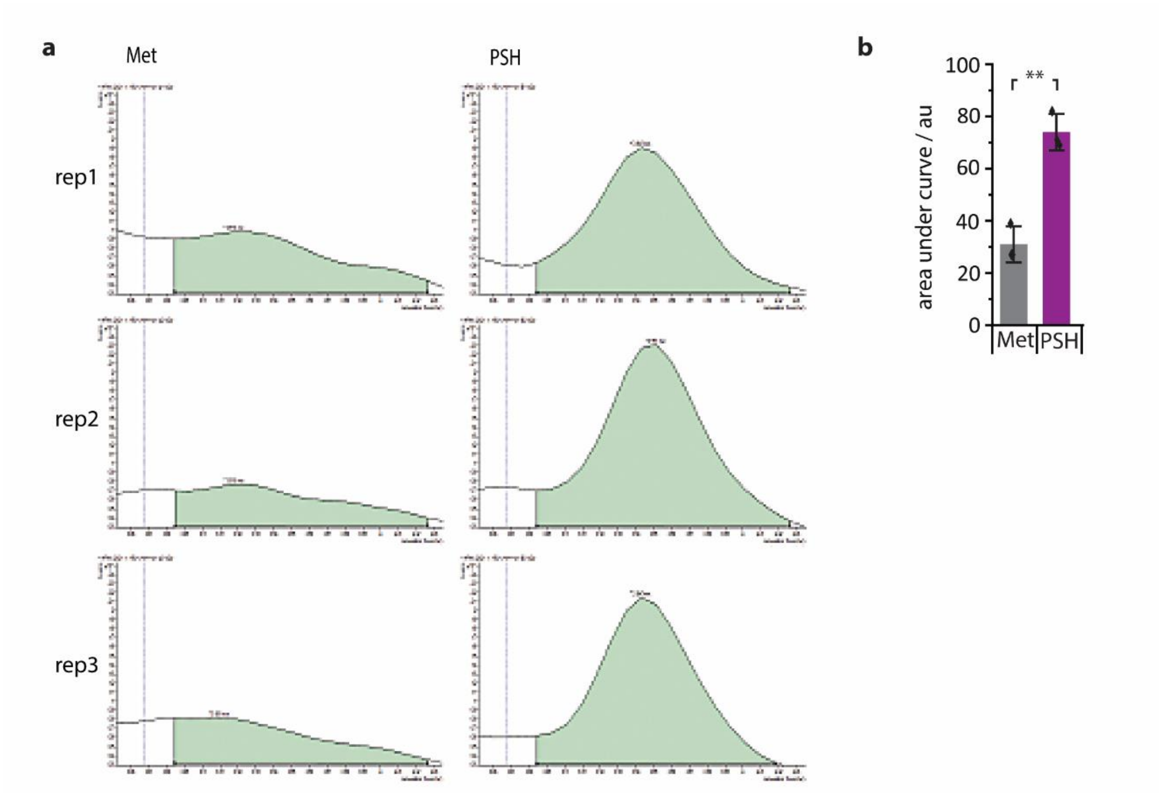

Supplementary Figure 30: **The signal intensity comparison of the MRM transition ions of prop^{5}C**. The MRM 282.1 → 150.1 (quantifier) is much more abundant than the MRM 282.1 → 121.9 and 282.1 → 80.0 (qualifiers). The signal is from highest point (cc7) of calibration curve (10 µL injection = 13.9 pg of prop^{5}C). Statistical significance determined via one-sample one-tailed t-test (n.s. P>0.05; * P≤0.05; ** P≤0.01; *** P≤0.001).

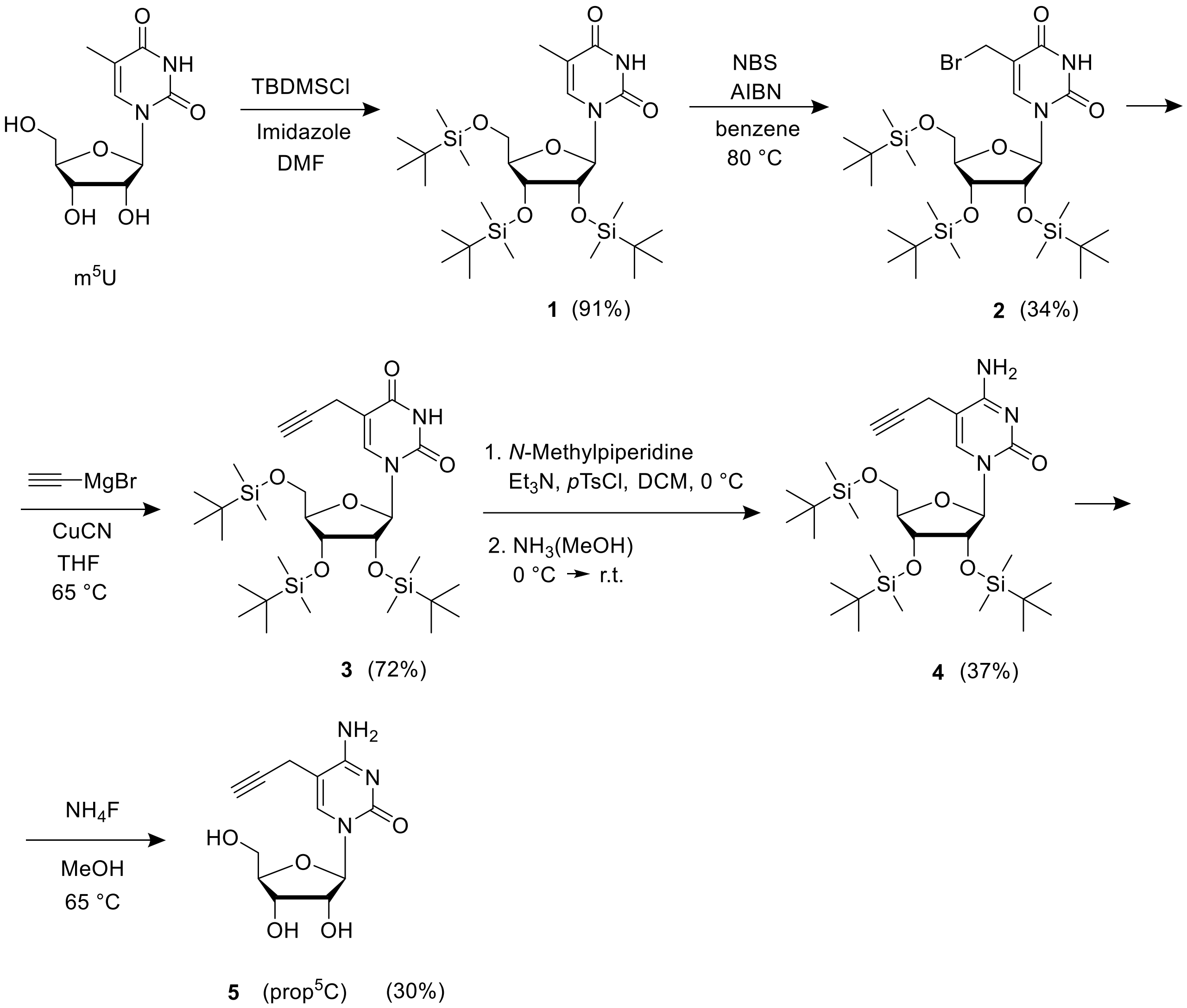

Supplementary Figure 31: **Synthesis of prop^{5}C standard.**

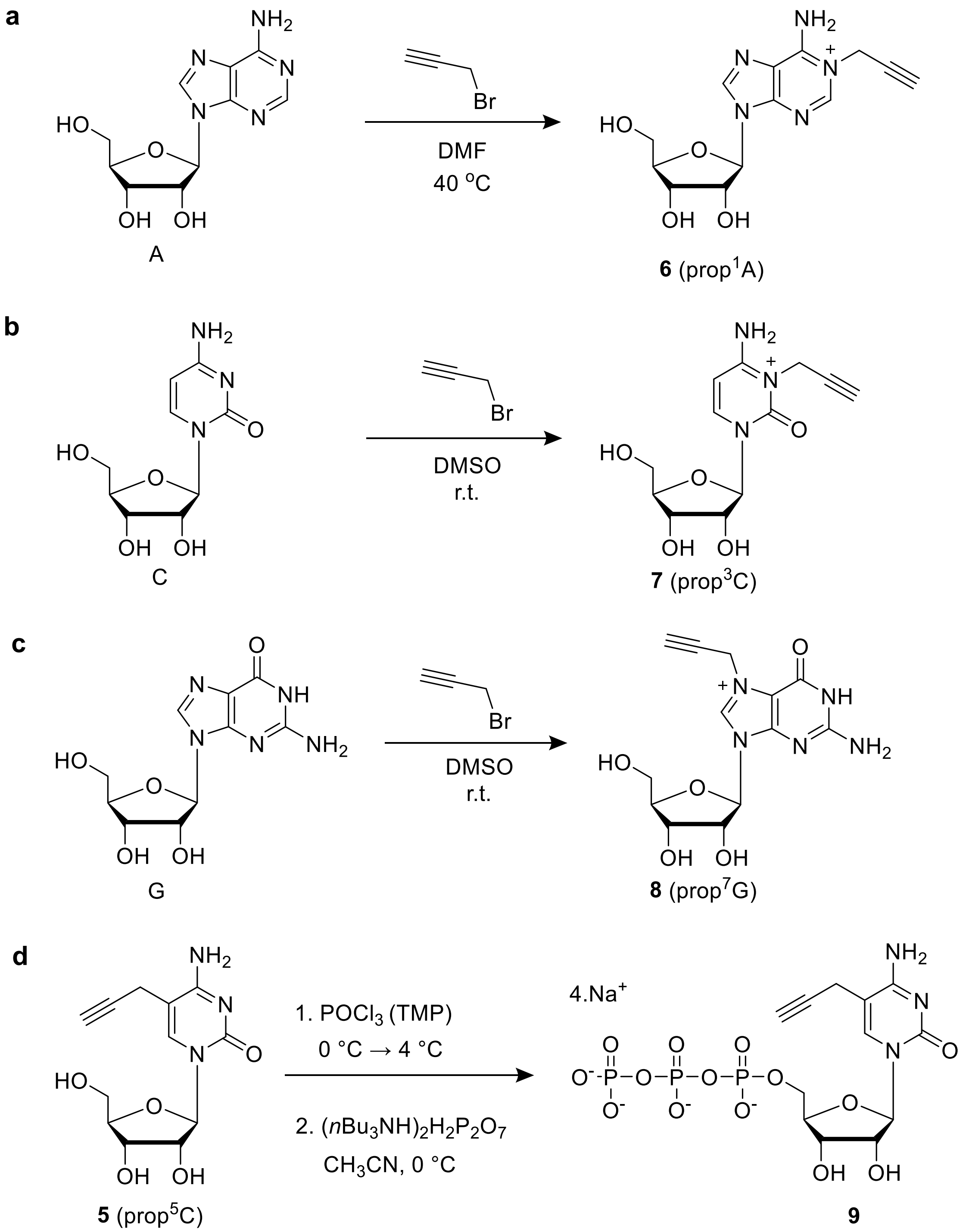

Supplementary Figure 32: **Synthesis of prop¹A (a), prop³C (b), prop⁷G (c) standards and prop⁵C triphosphate (d)**.

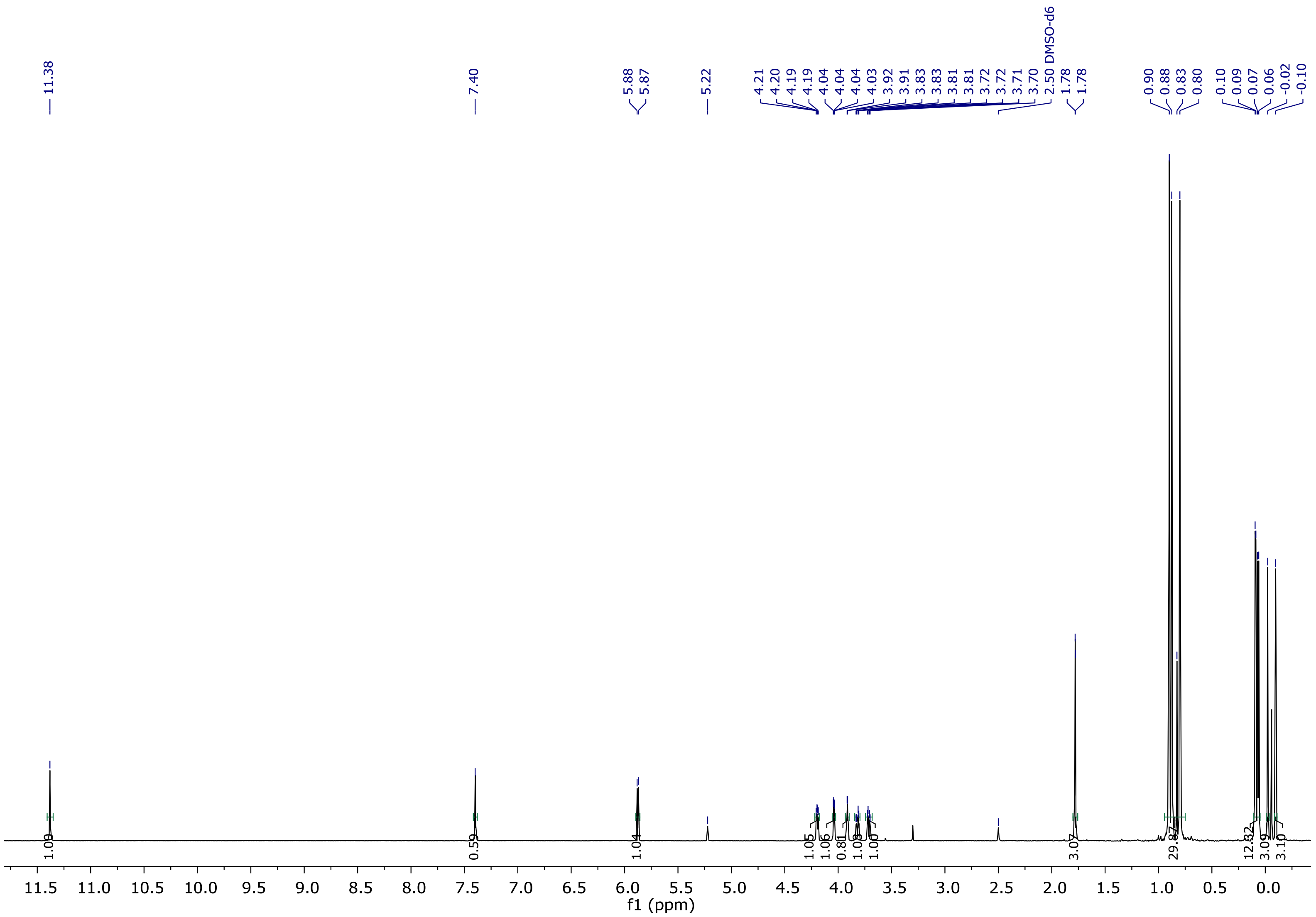

Supplementary Figure 33: ^{1}**H-NMR spectrum of compound 1**.

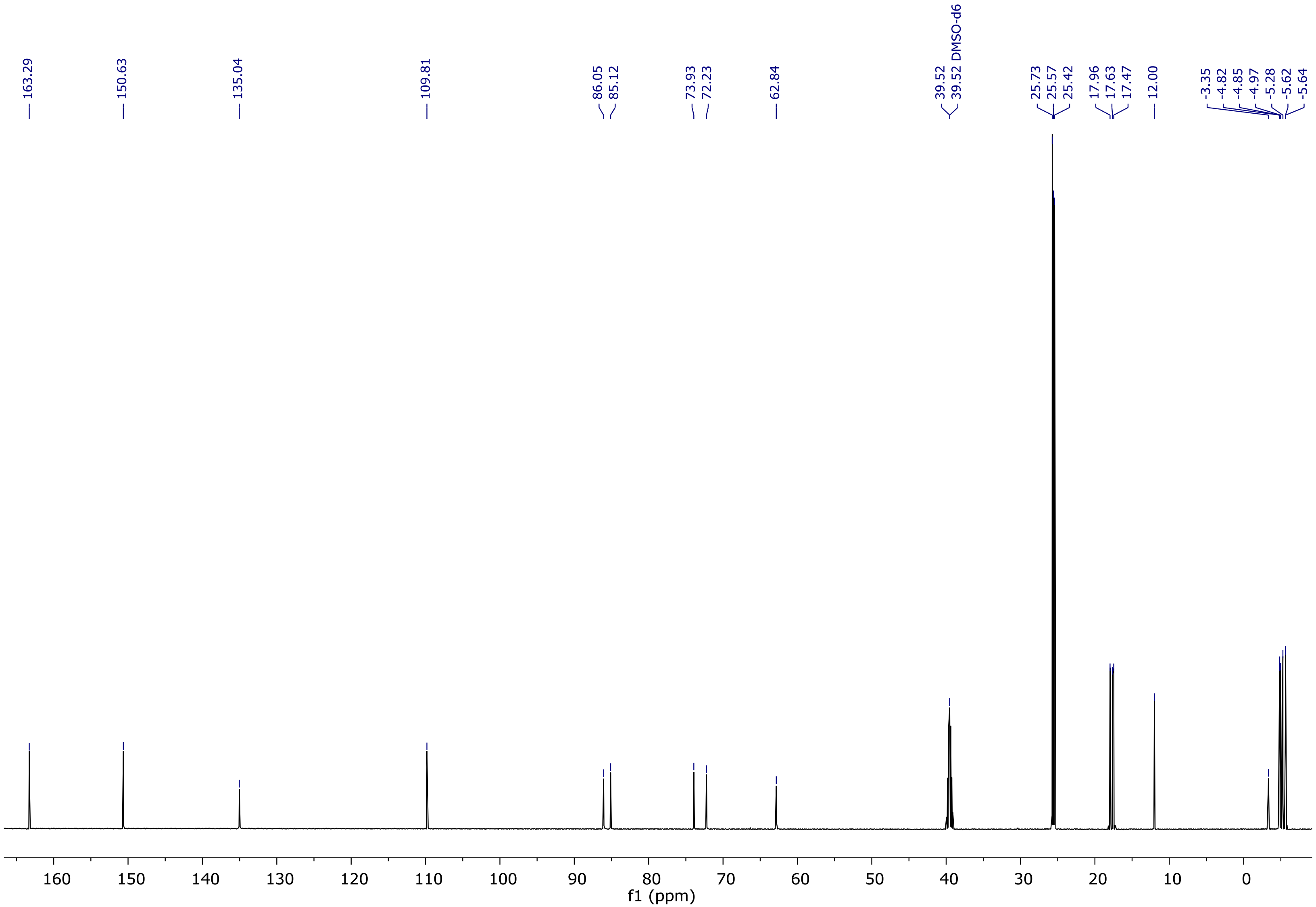

Supplementary Figure 34: **^{13}C-NMR spectrum of compound 1.**

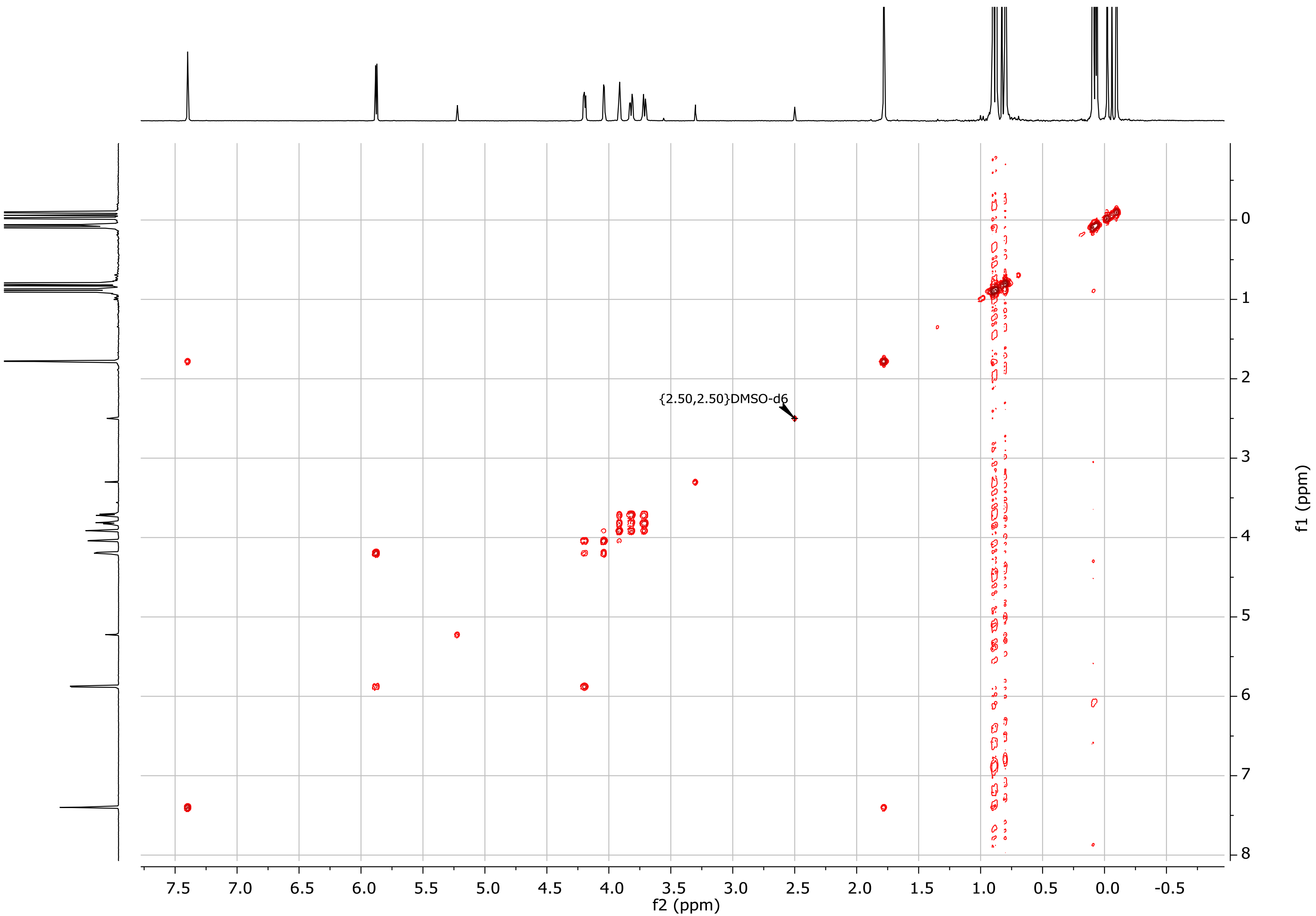

Supplementary Figure 35: **COSY spectrum of compound 1.**

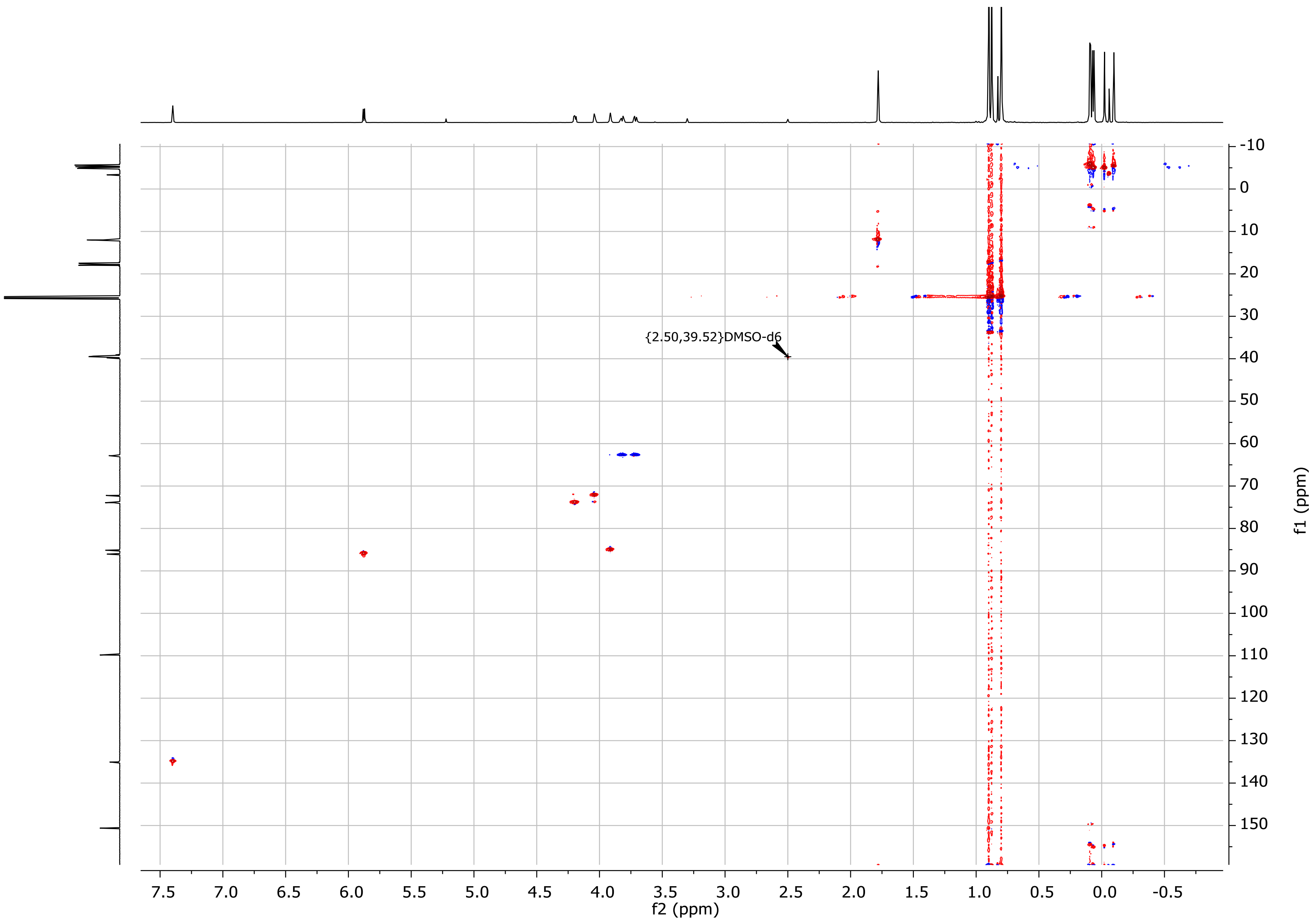

Supplementary Figure 36: **HSQC spectrum of compound 1**.

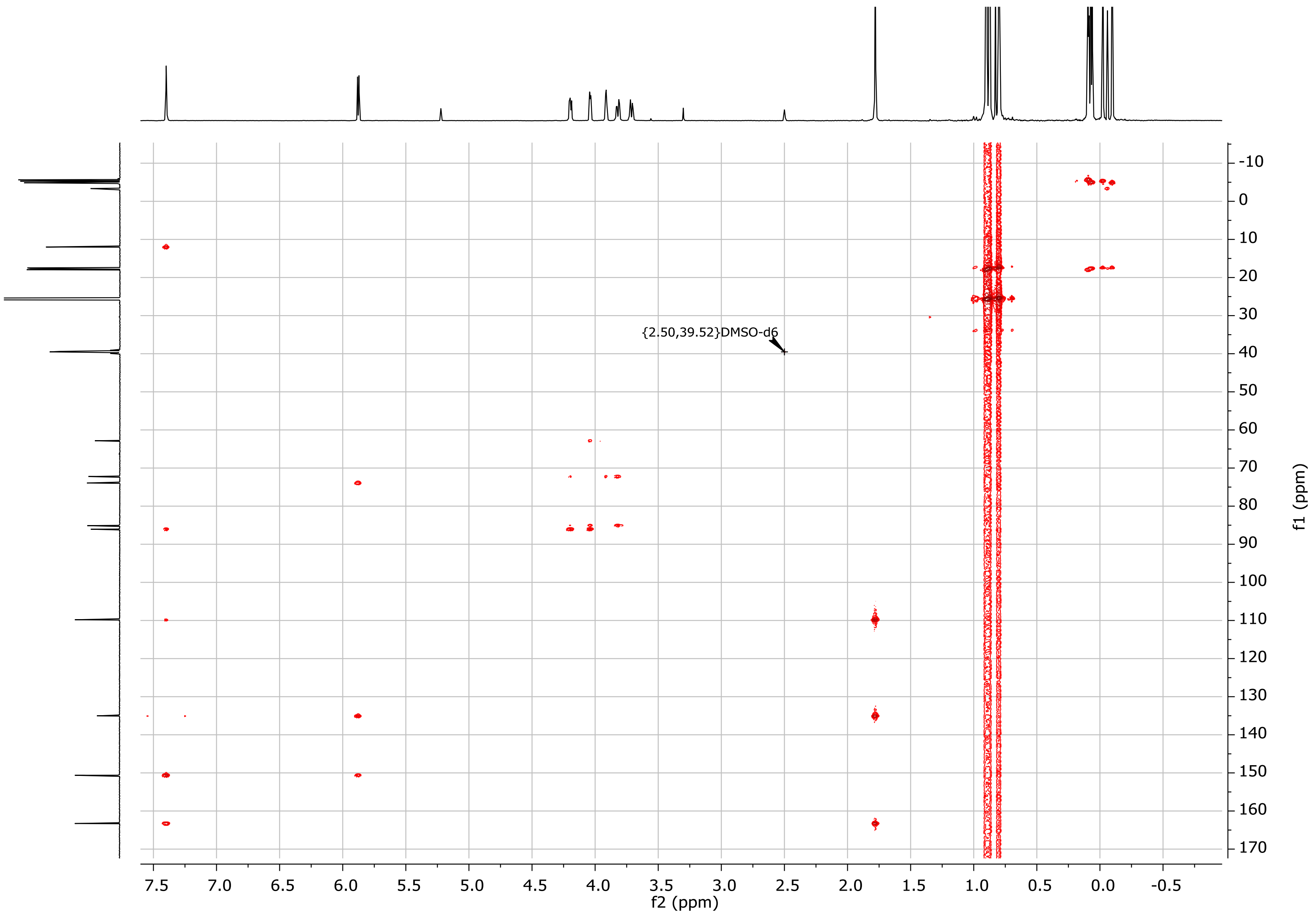

Supplementary Figure 37: **HMBC spectrum of compound 1**.

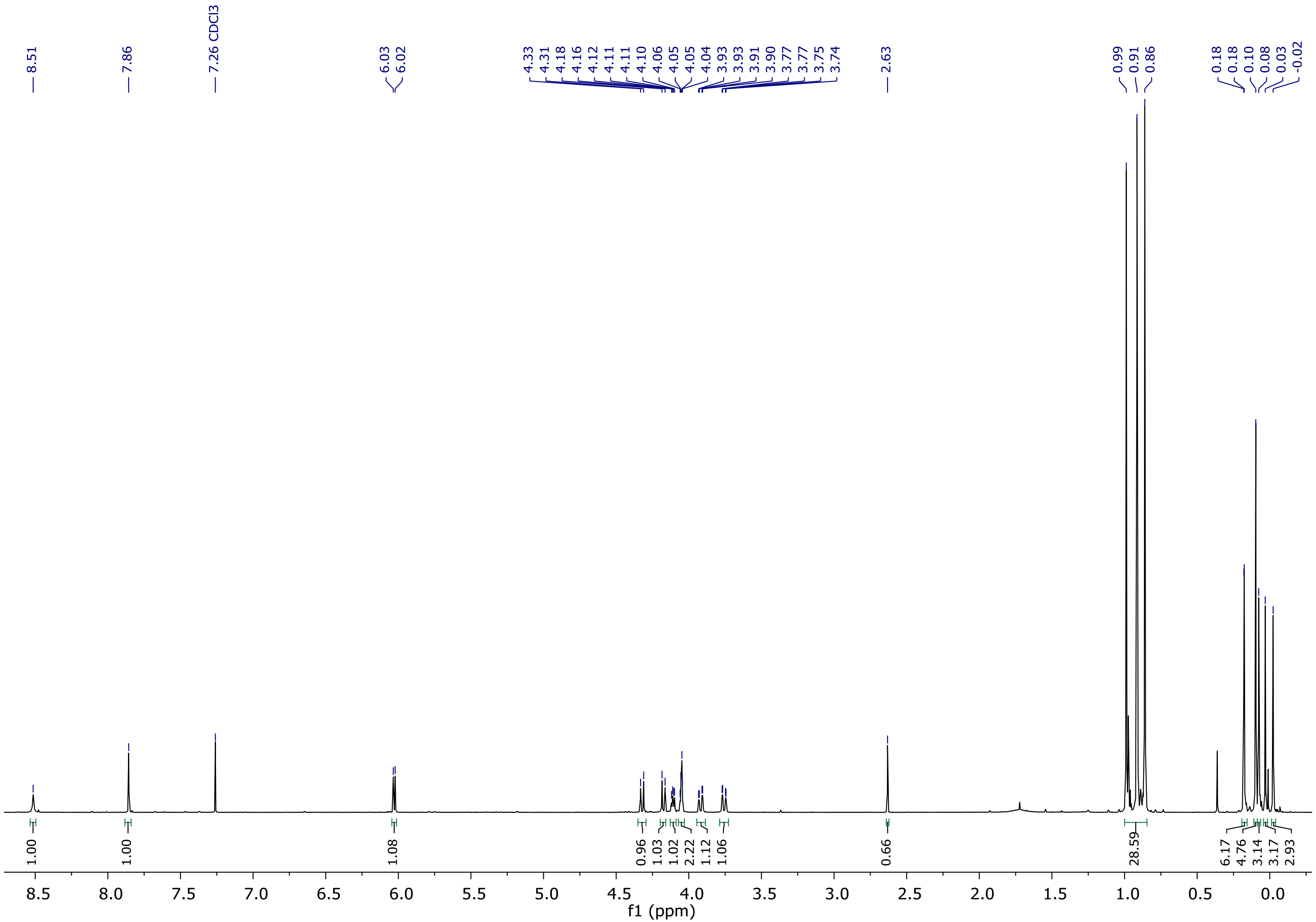

Supplementary Figure 38: ^{1}**H-NMR spectrum of compound 2**.

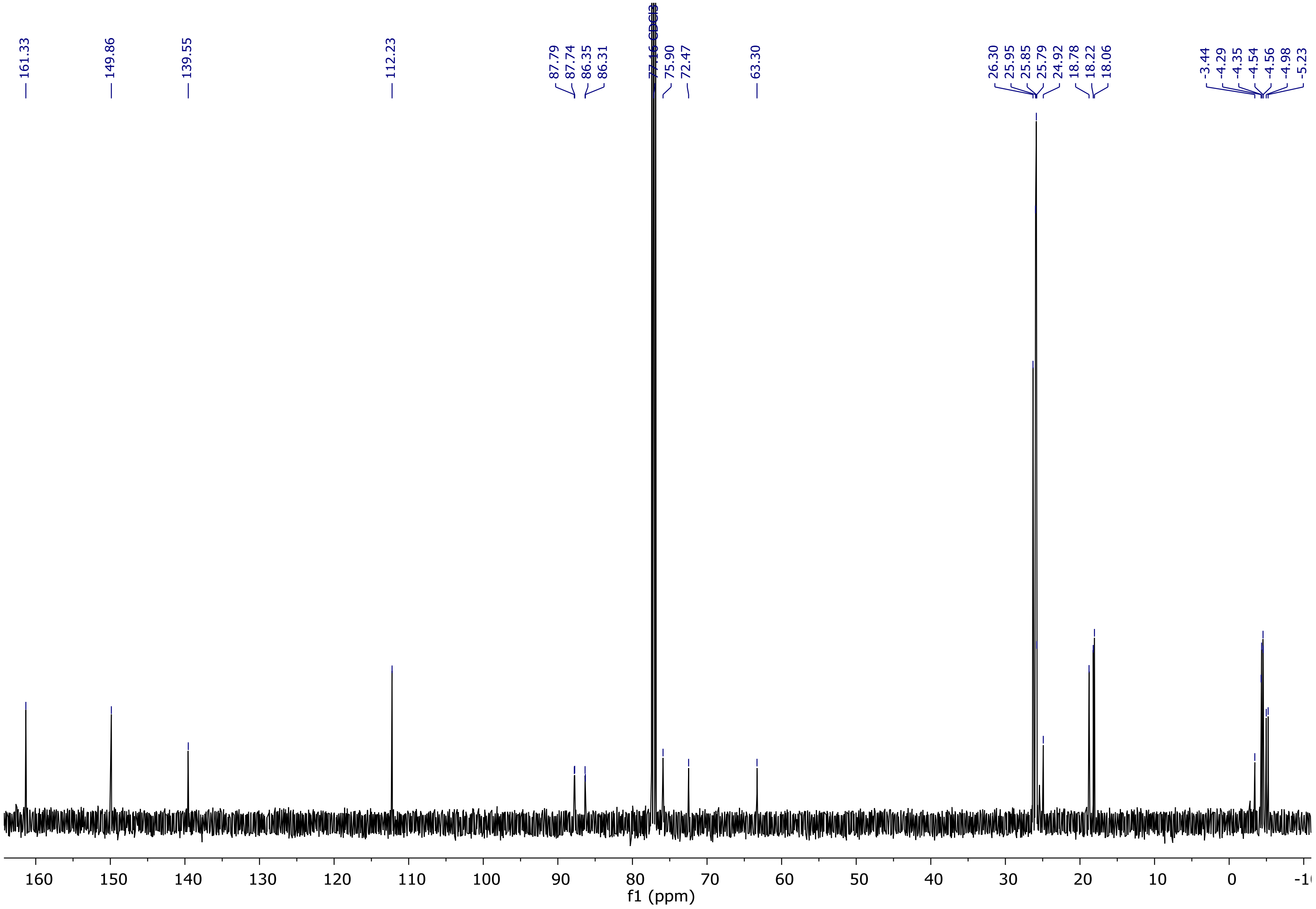

Supplementary Figure 39: ^{13}**C-NMR spectrum of compound 2**.

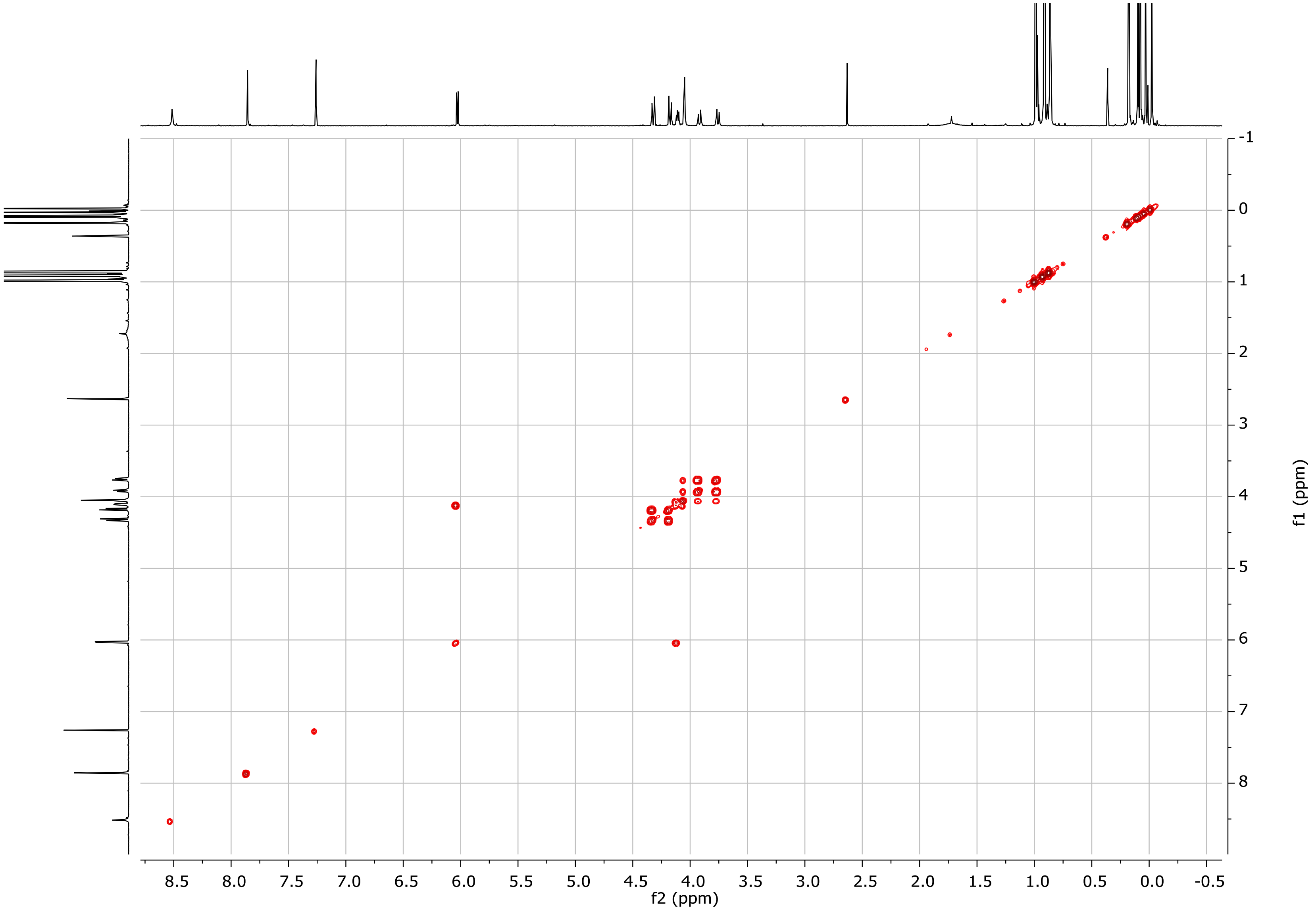

Supplementary Figure 40: **COSY spectrum of compound 2**.

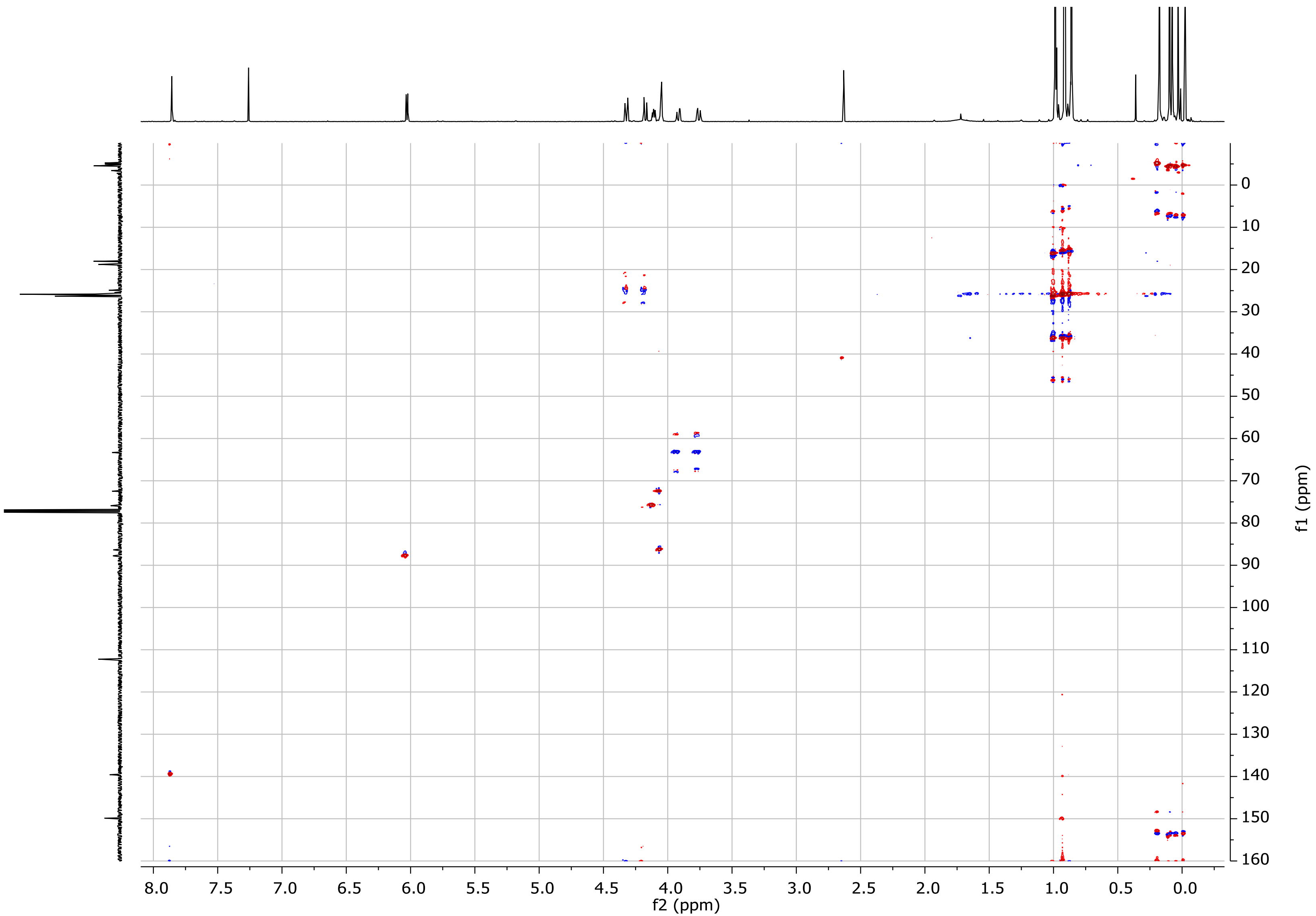

Supplementary Figure 41: **HSQC spectrum of compound 2**.

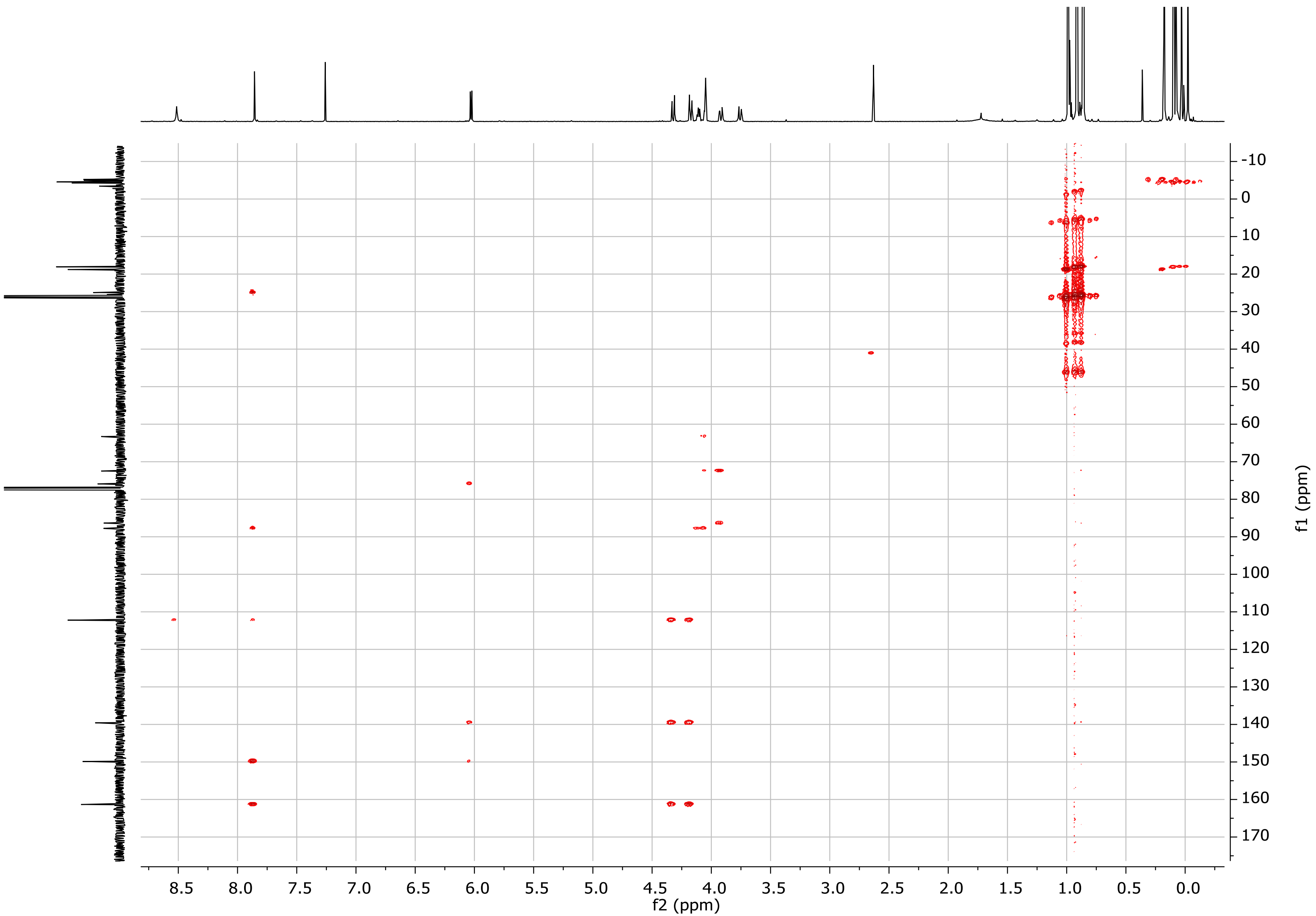

Supplementary Figure 42: **HMBC spectrum of compound 2**.

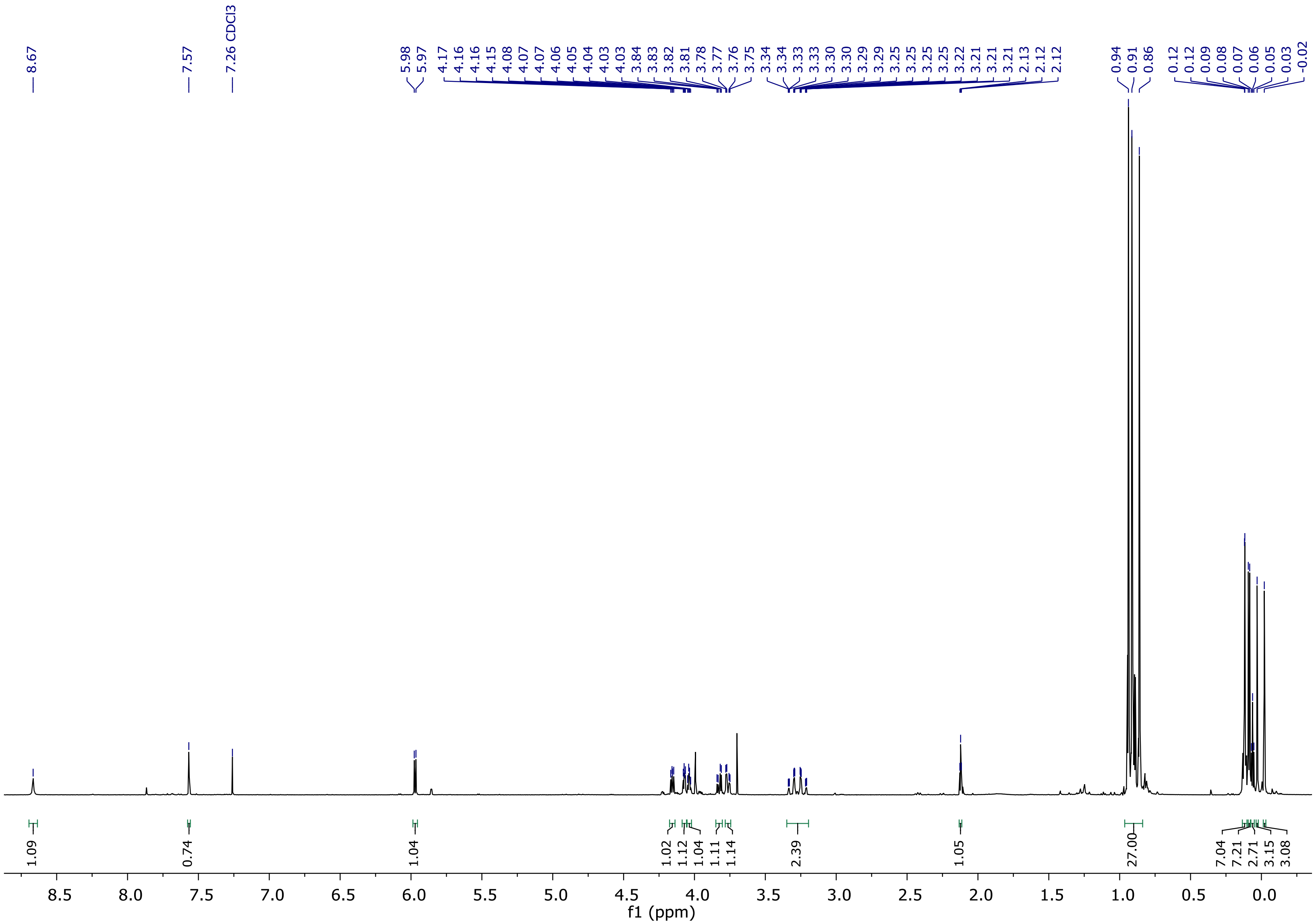

Supplementary Figure 43: **^{1}H-NMR spectrum of compound 3**.

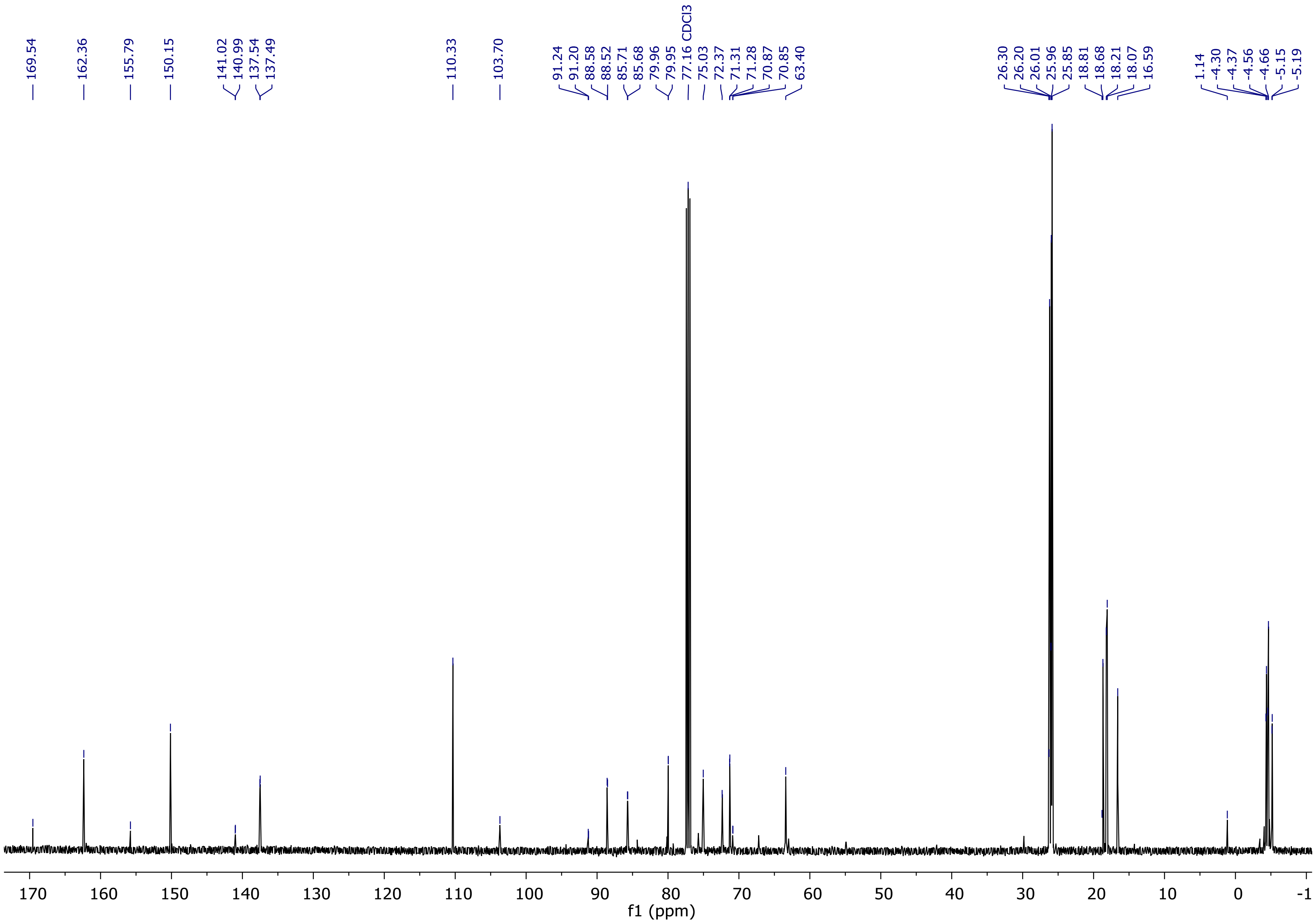

Supplementary Figure 44: **^{13}C-NMR spectrum of compound 3**.

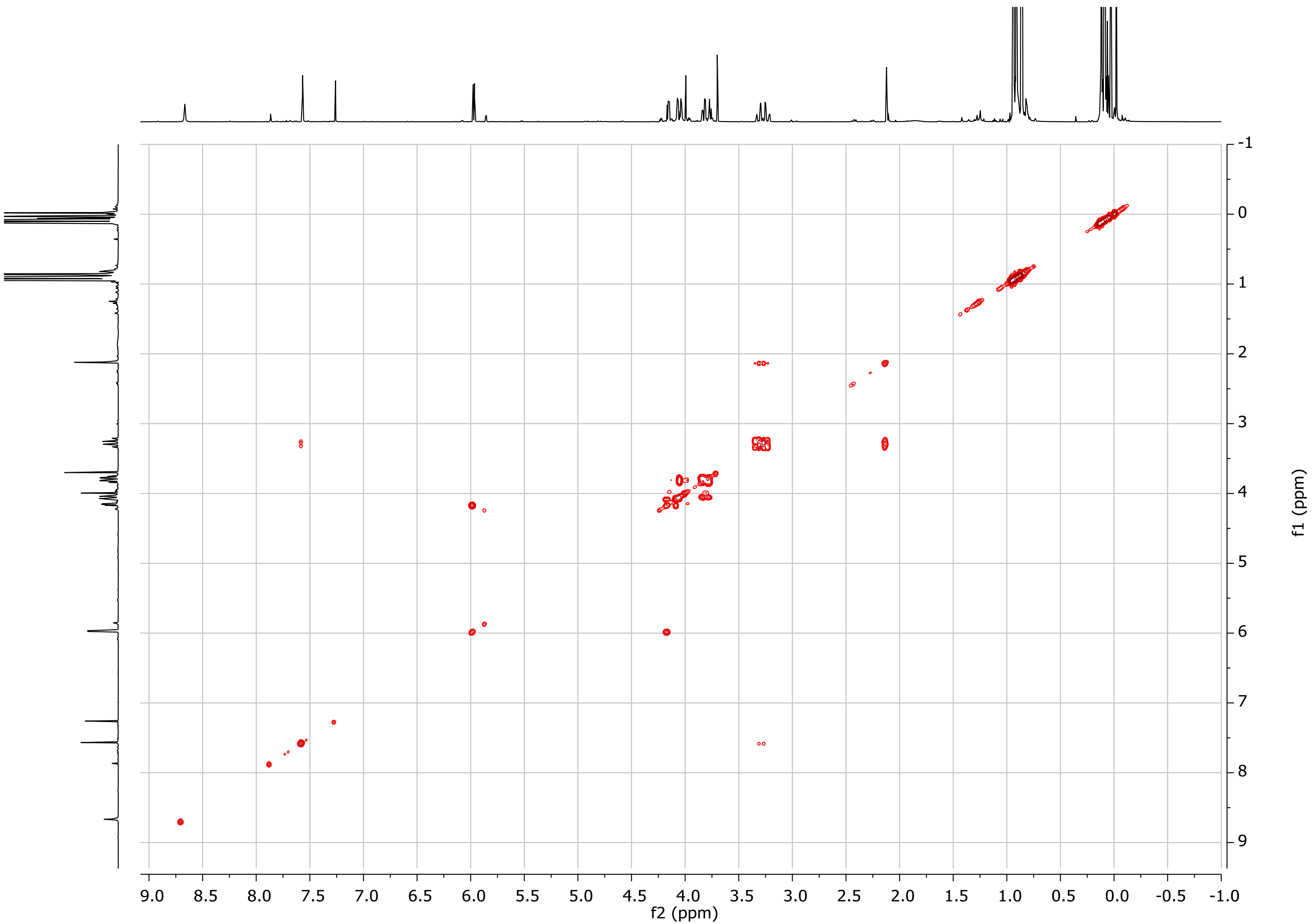

Supplementary Figure 45: **COSY spectrum of compound 3**.

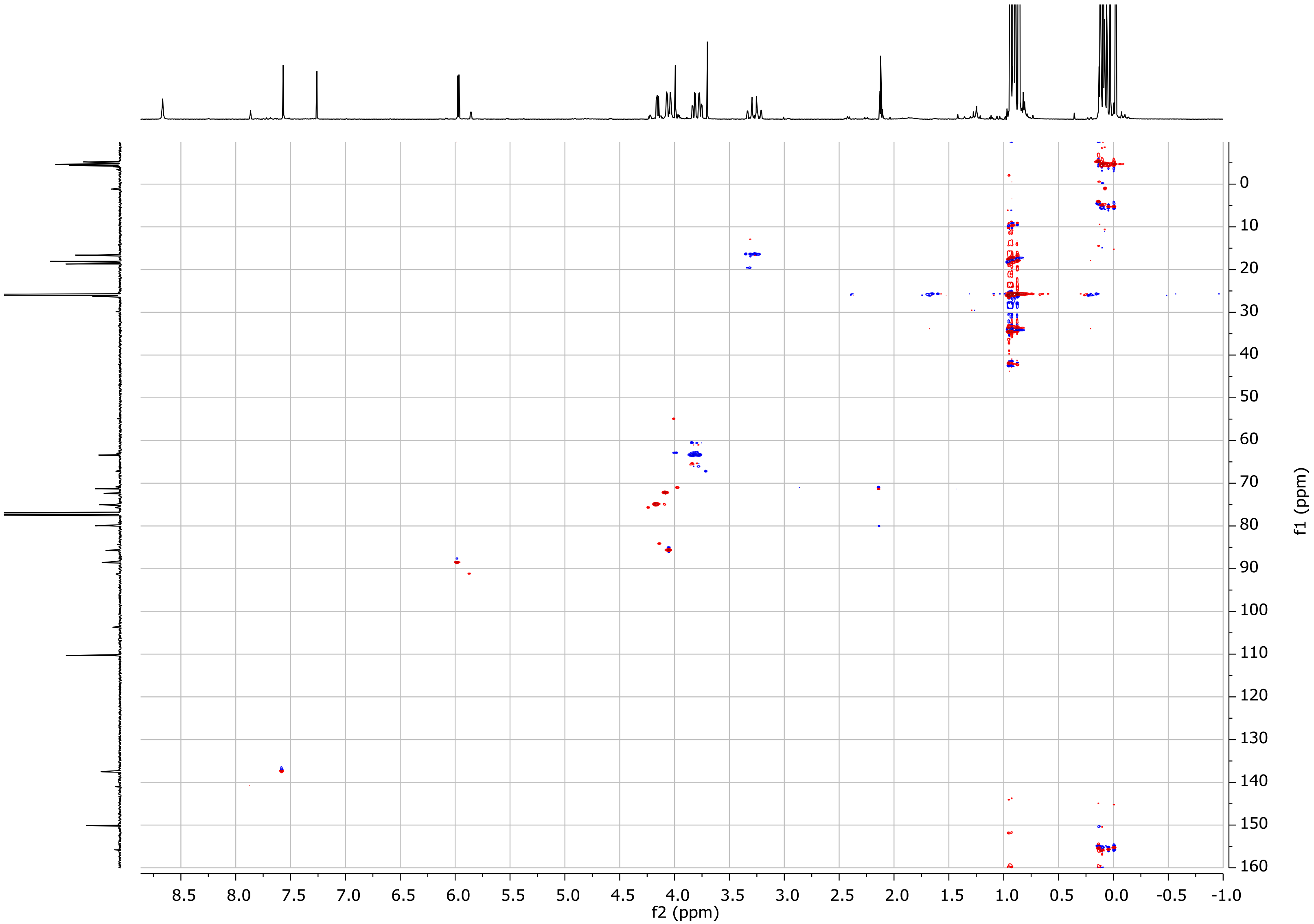

Supplementary Figure 46: **HSQC spectrum of compound 3.**

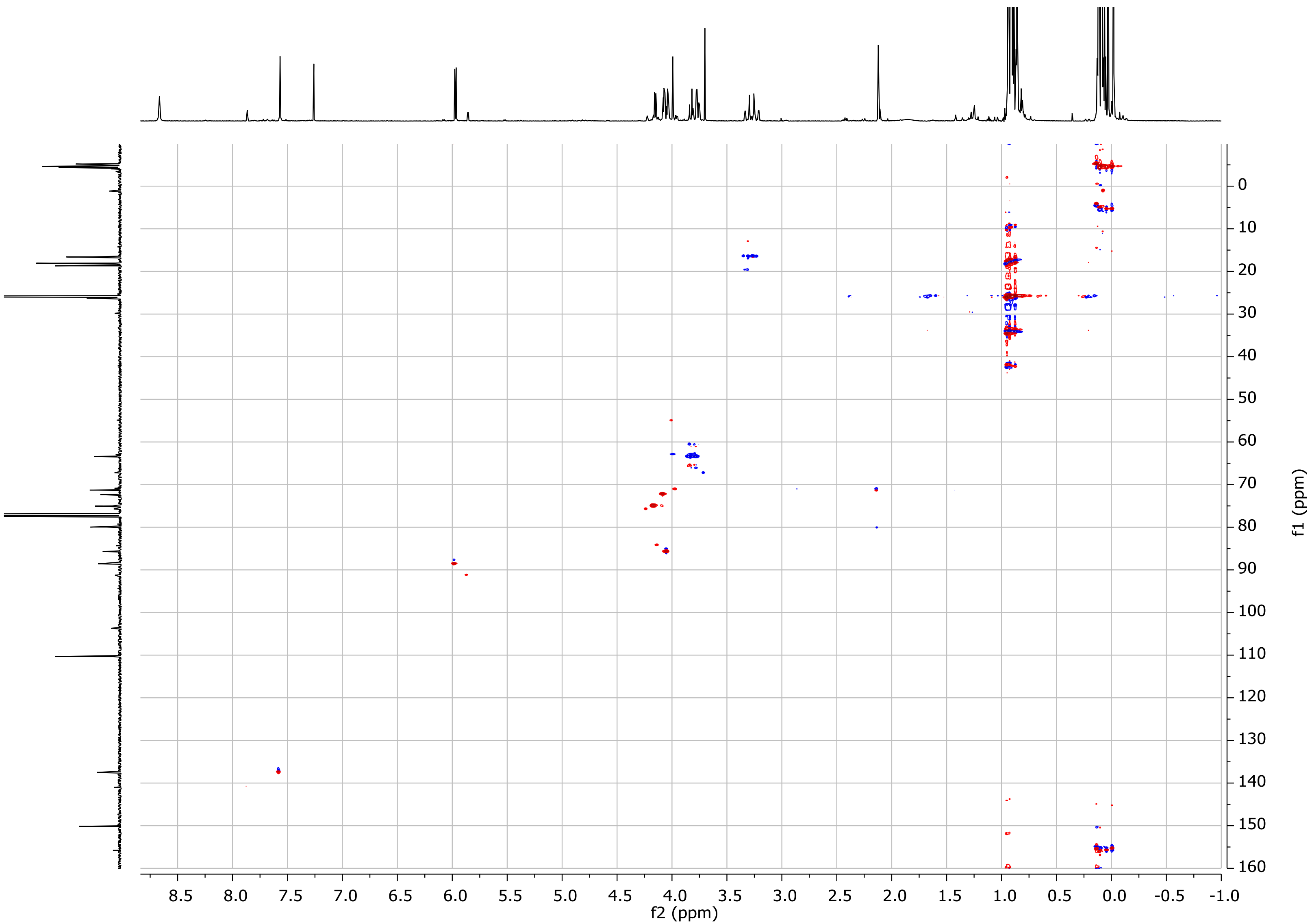

Supplementary Figure 47: **HMBC spectrum of compound 3**.

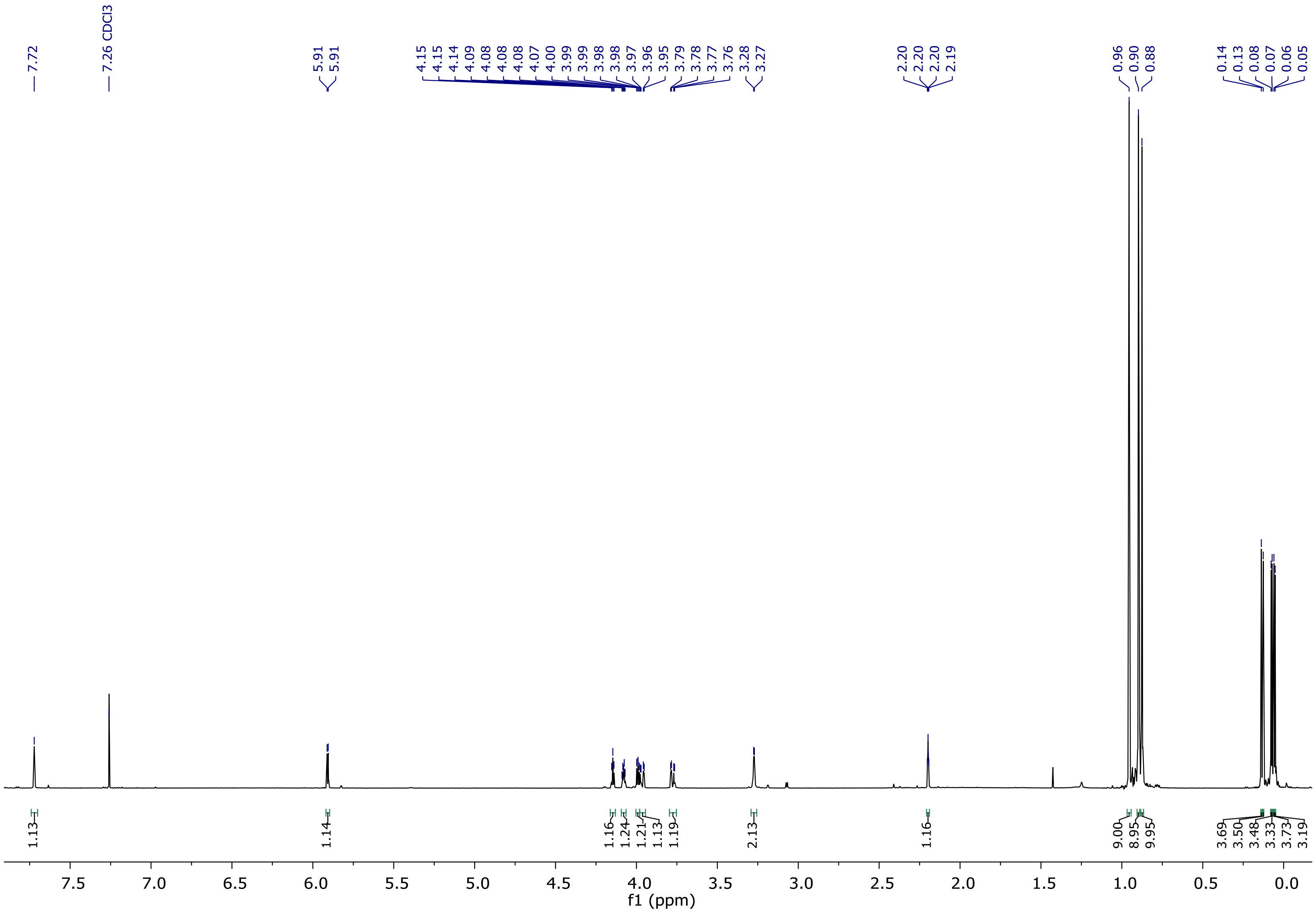

Supplementary Figure 48: **^{1}H-NMR spectrum of compound 4.**

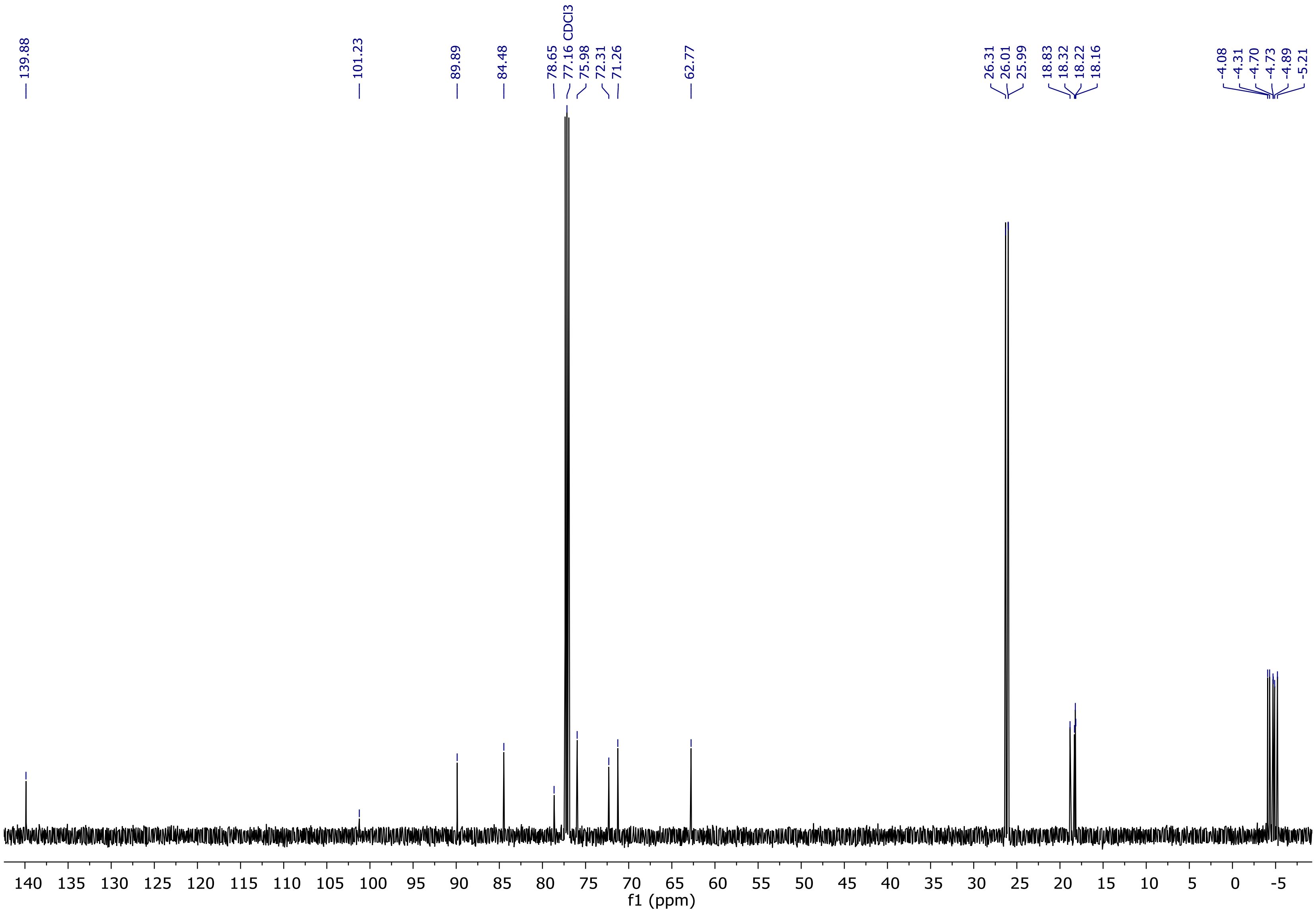

Supplementary Figure 49: **^{13}C-NMR spectrum of compound 4**.

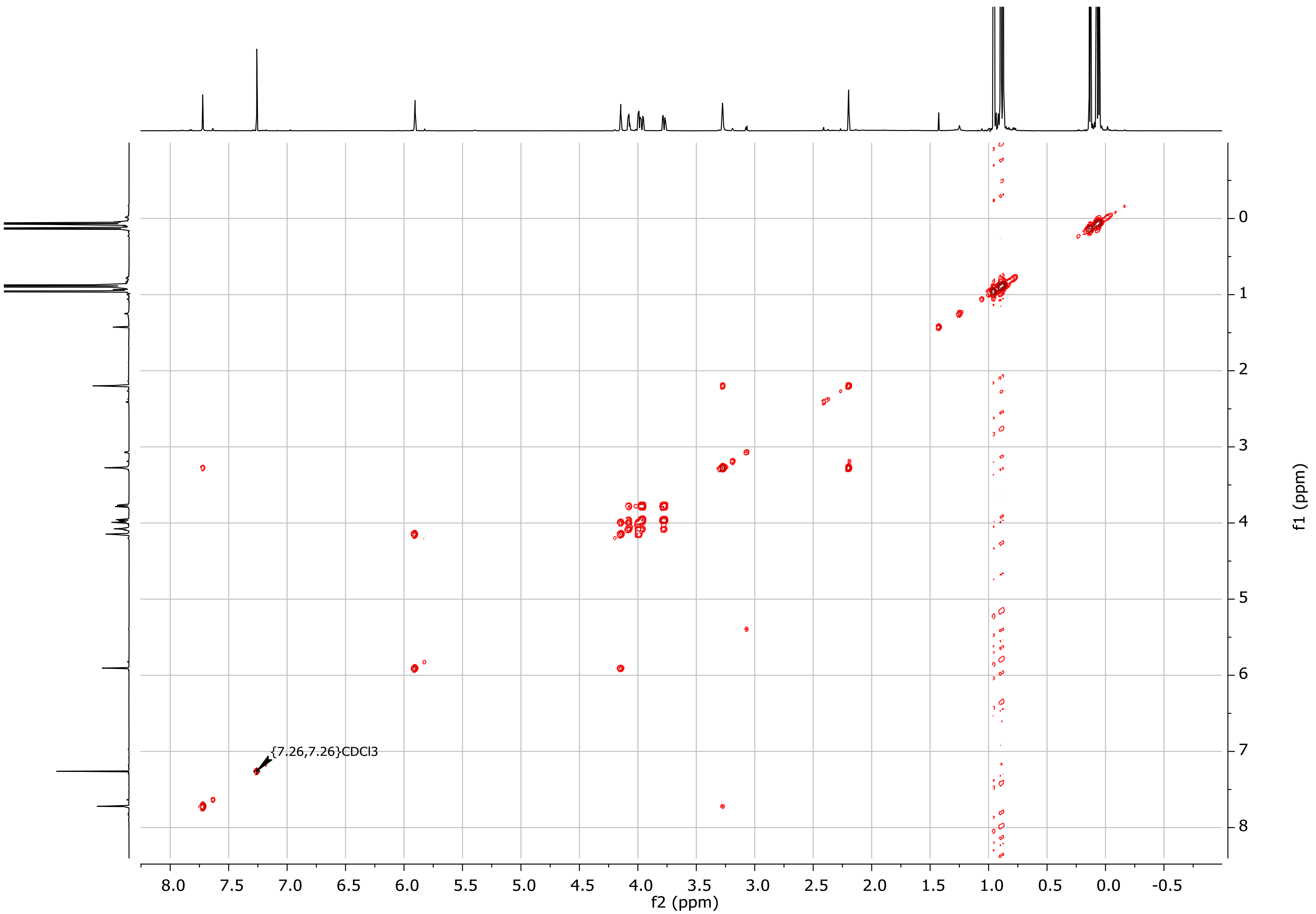

Supplementary Figure 50: **COSY spectrum of compound 4.**

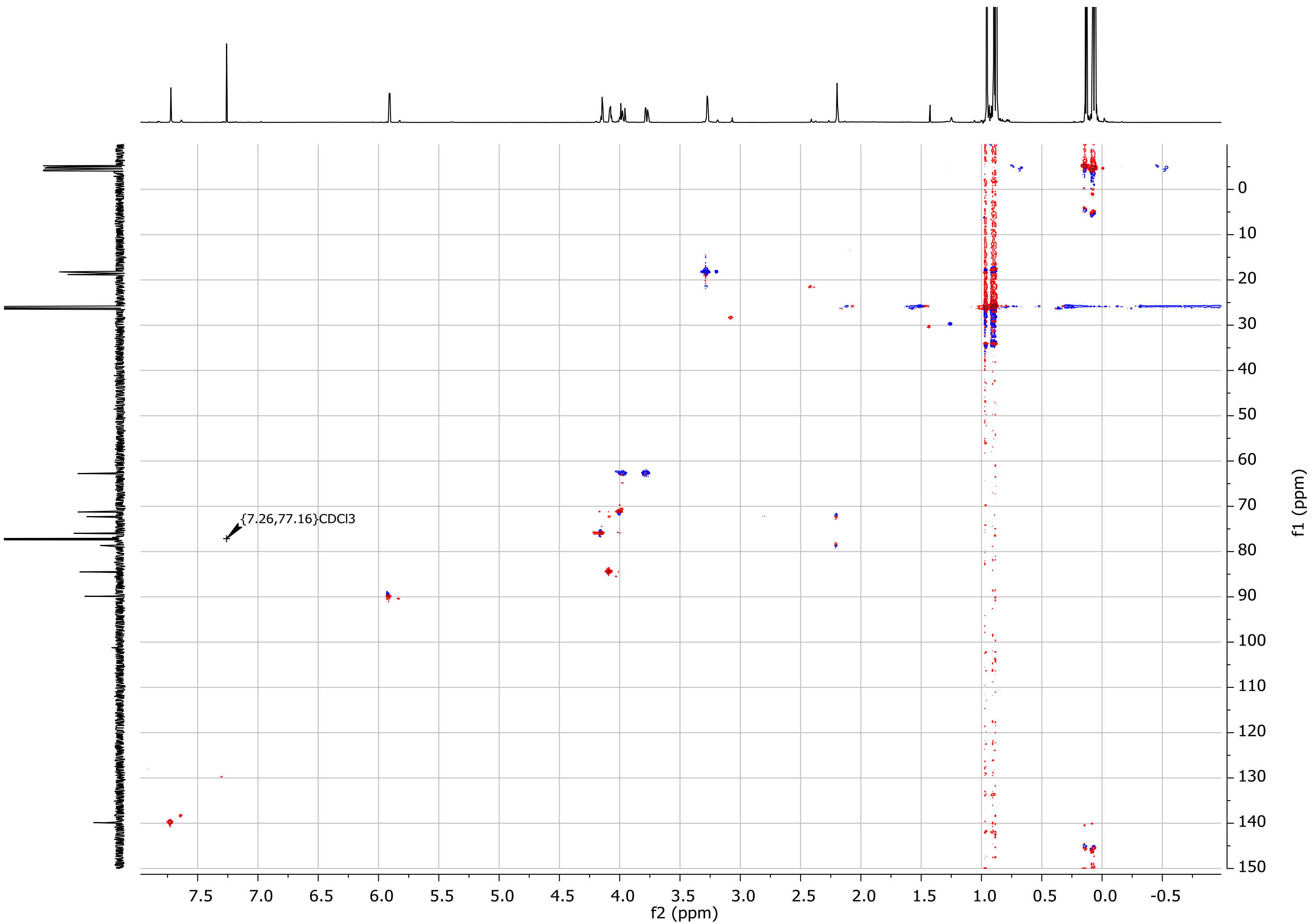

Supplementary Figure 51: **HSQC spectrum of compound 4**.

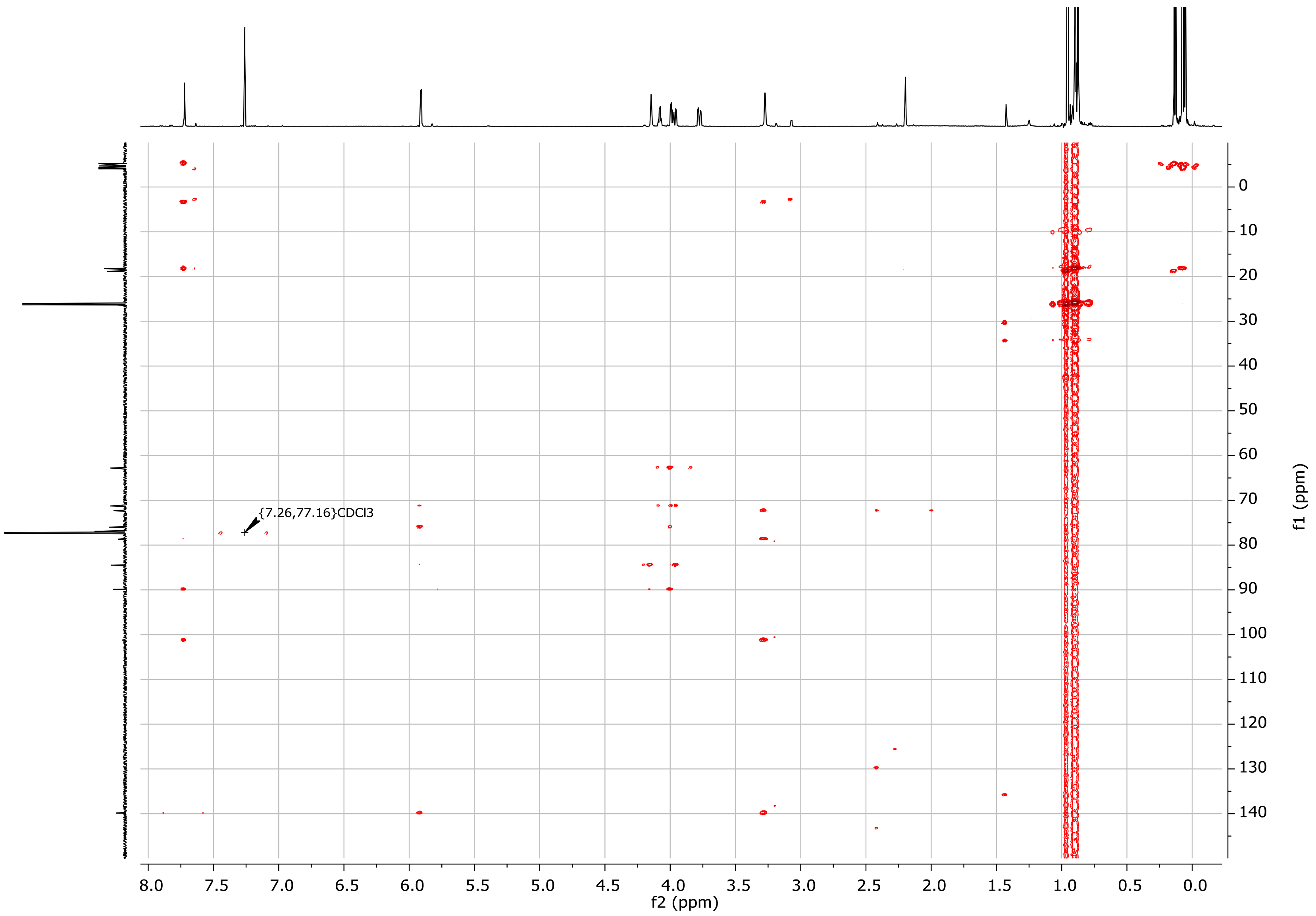

Supplementary Figure 52: **HMBC spectrum of compound 4**.

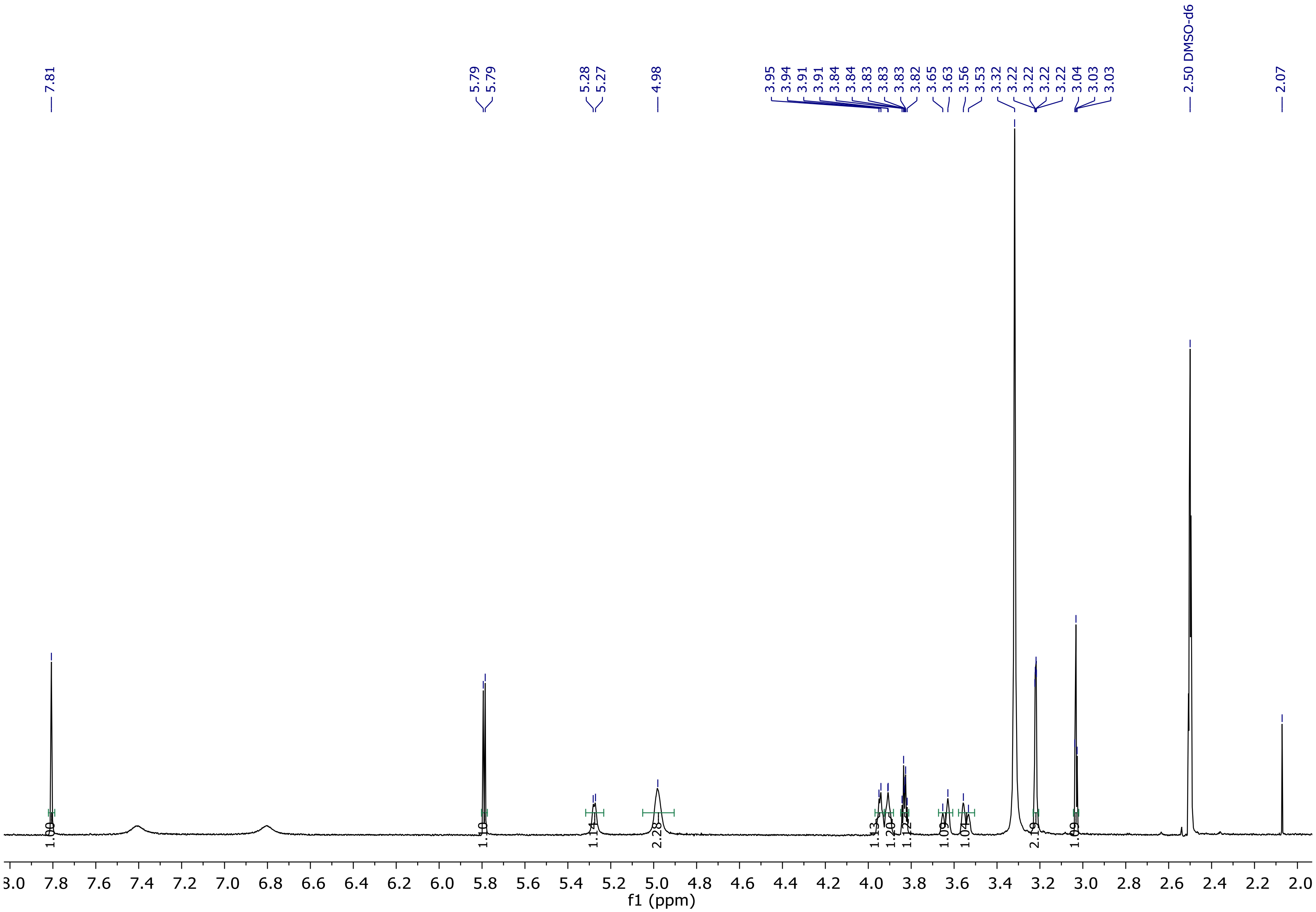

Supplementary Figure 53: ^{1}**H-NMR spectrum of compound 5**.

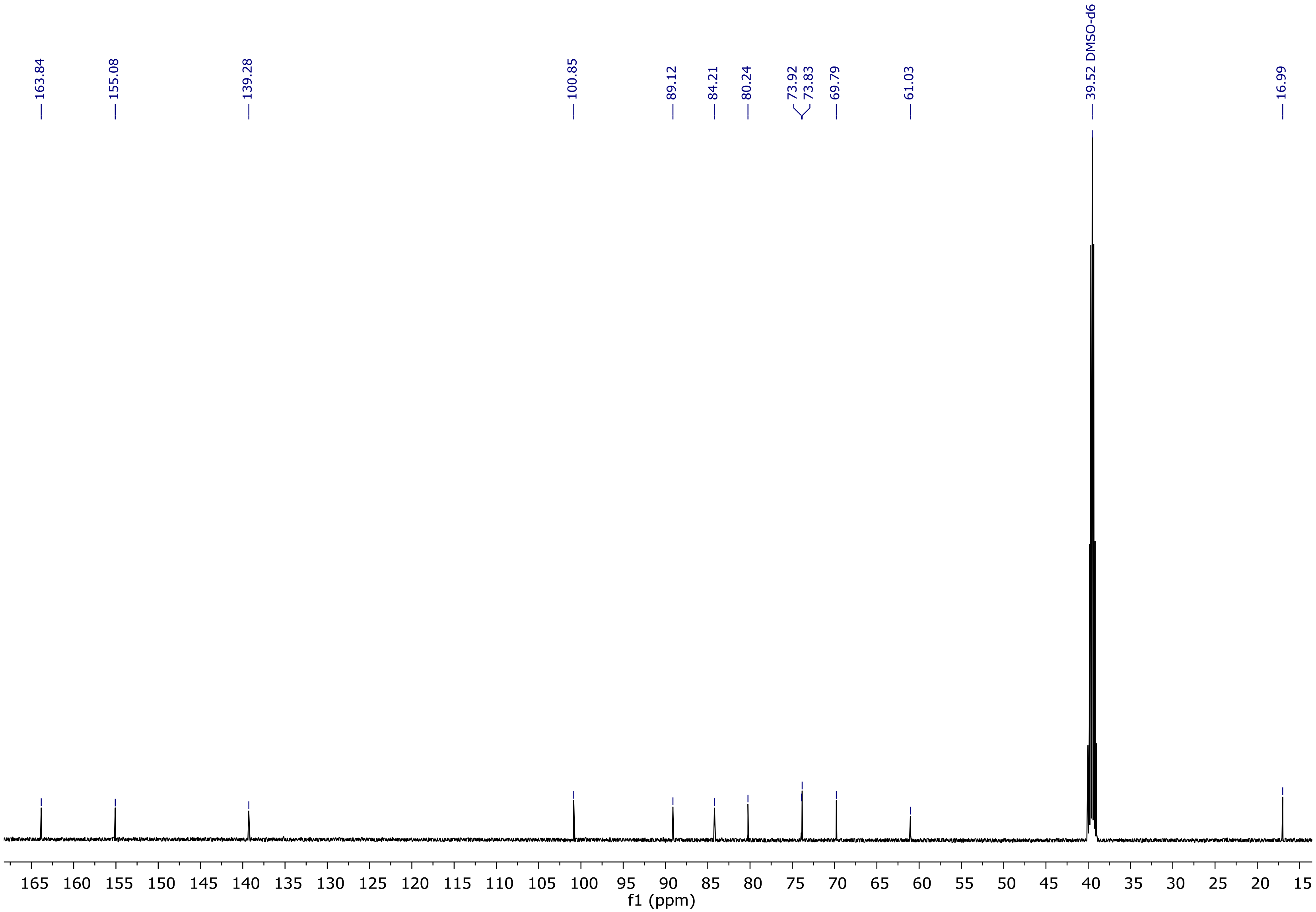

Supplementary Figure 54: **^{13}C-NMR spectrum of compound 5**.

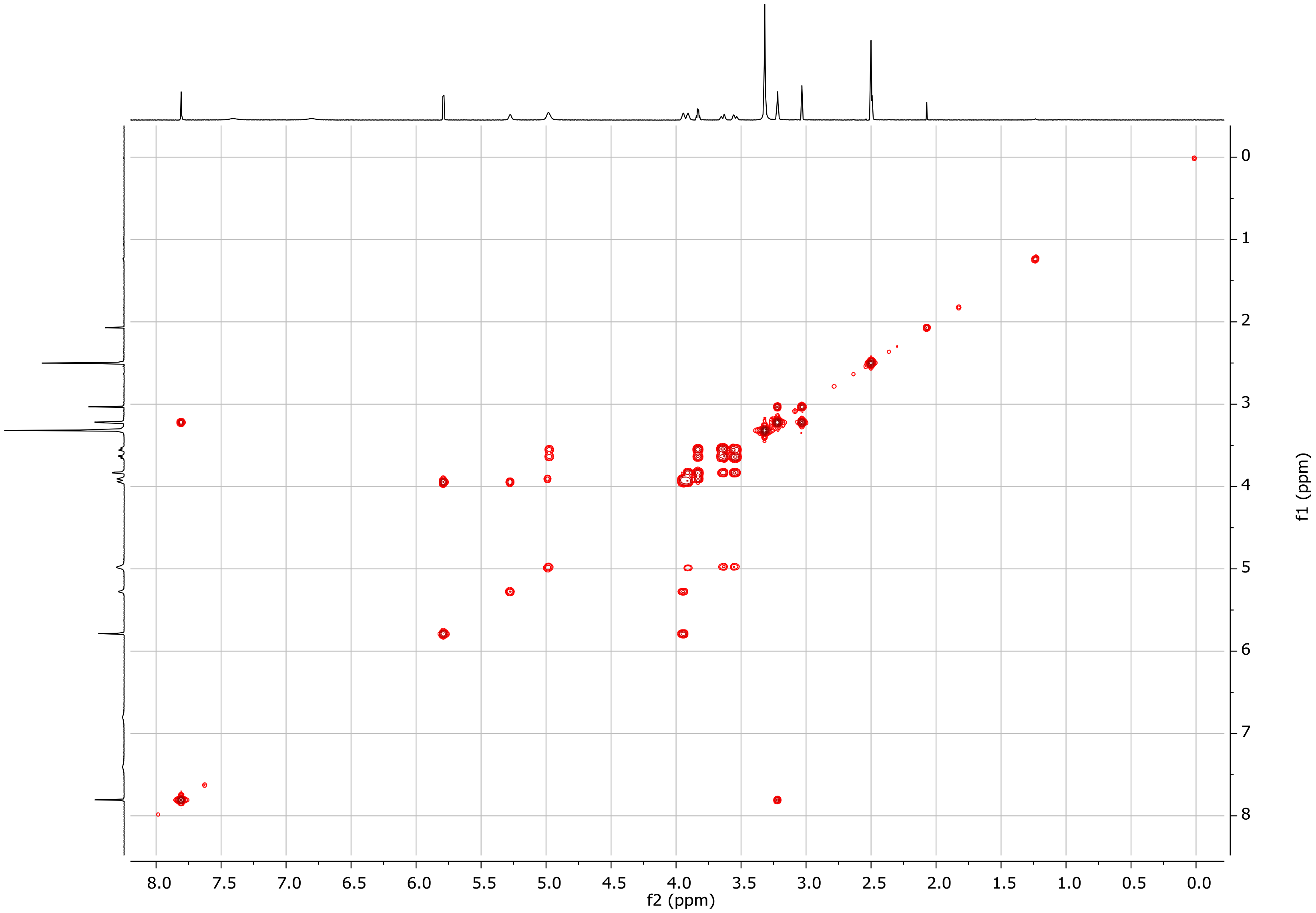

Supplementary Figure 55: **COSY spectrum of compound 5**.

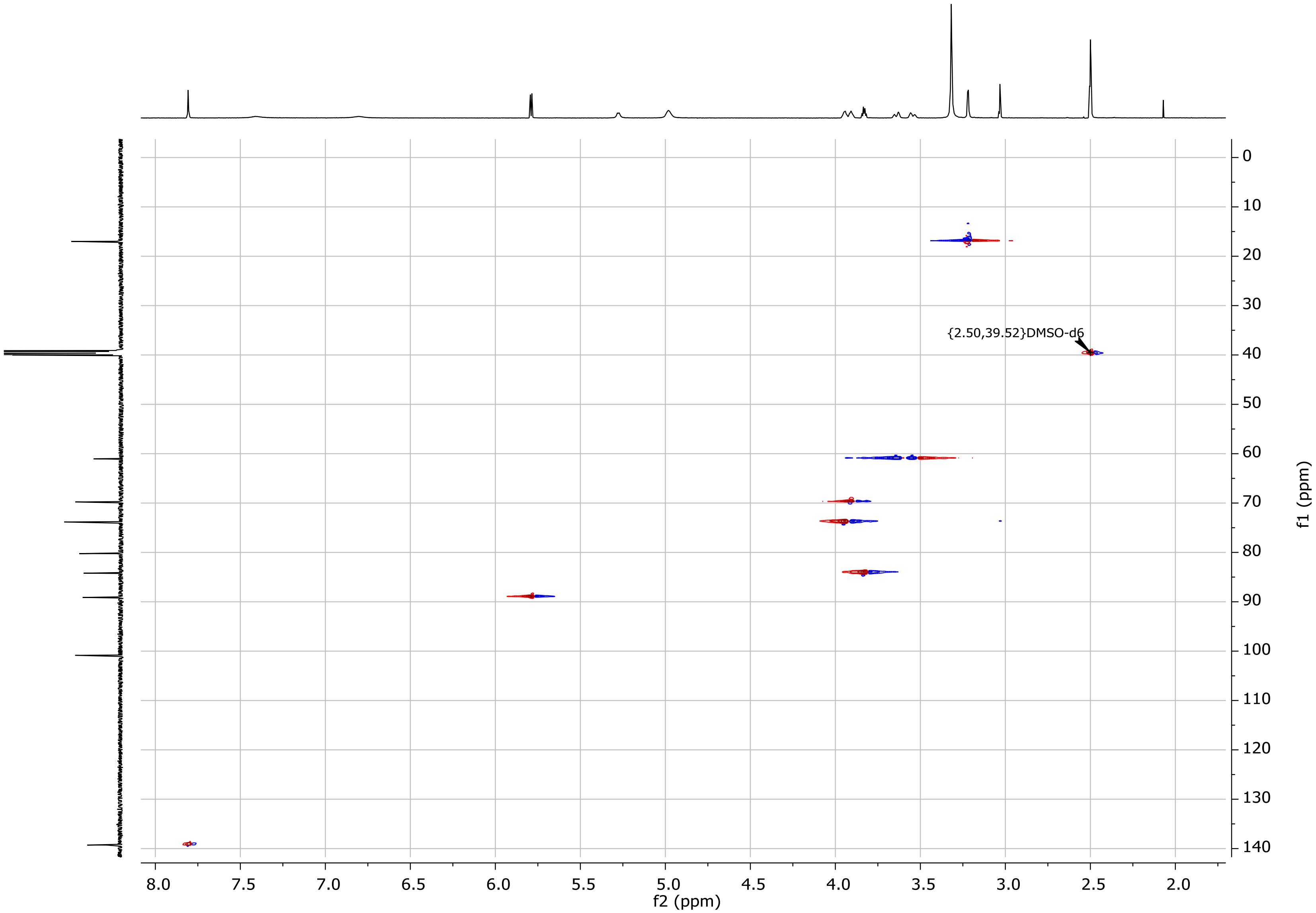

Supplementary Figure 56: **HSQC spectrum of compound 5.**

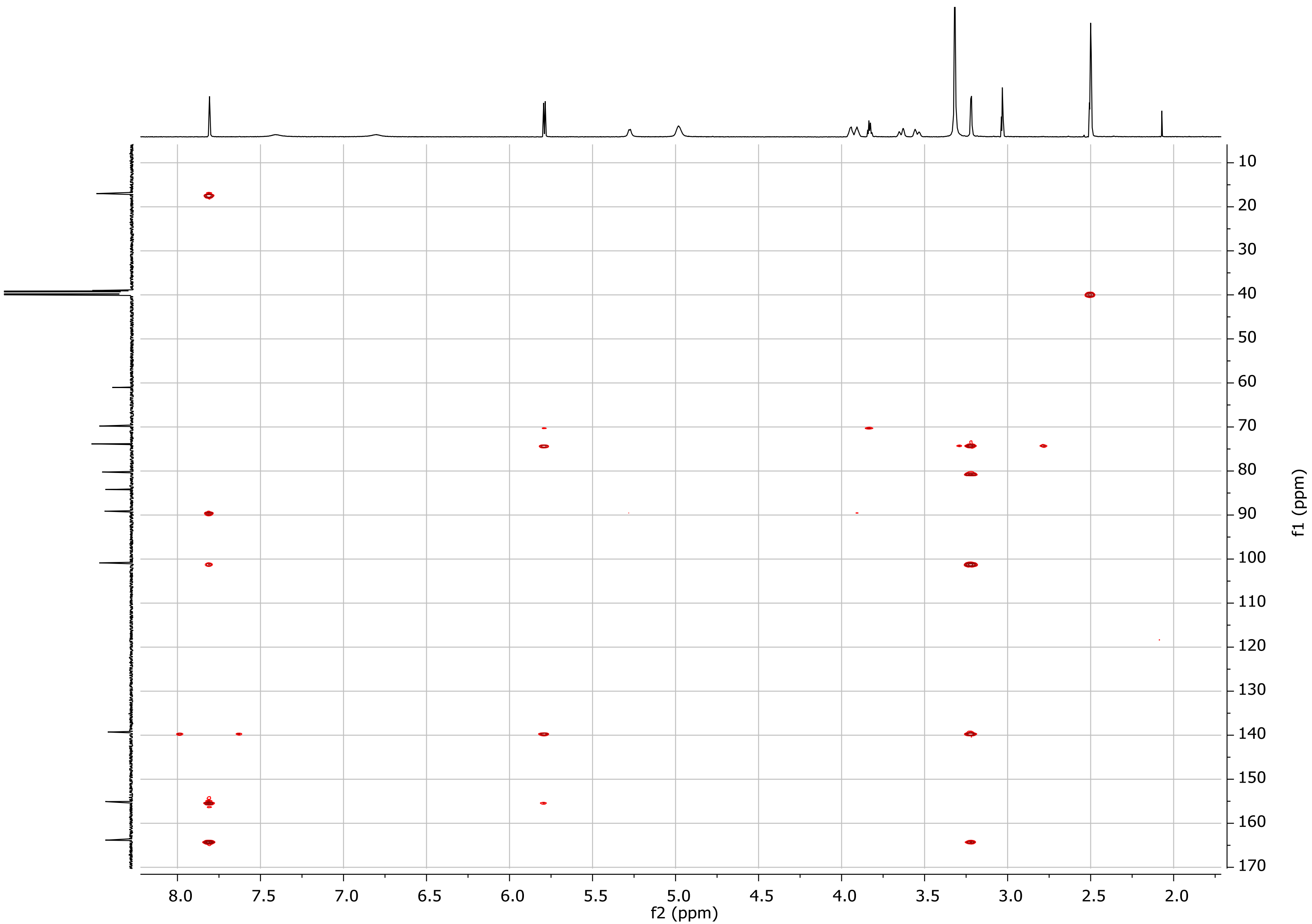

Supplementary Figure 57: **HMBC spectrum of compound 5.**

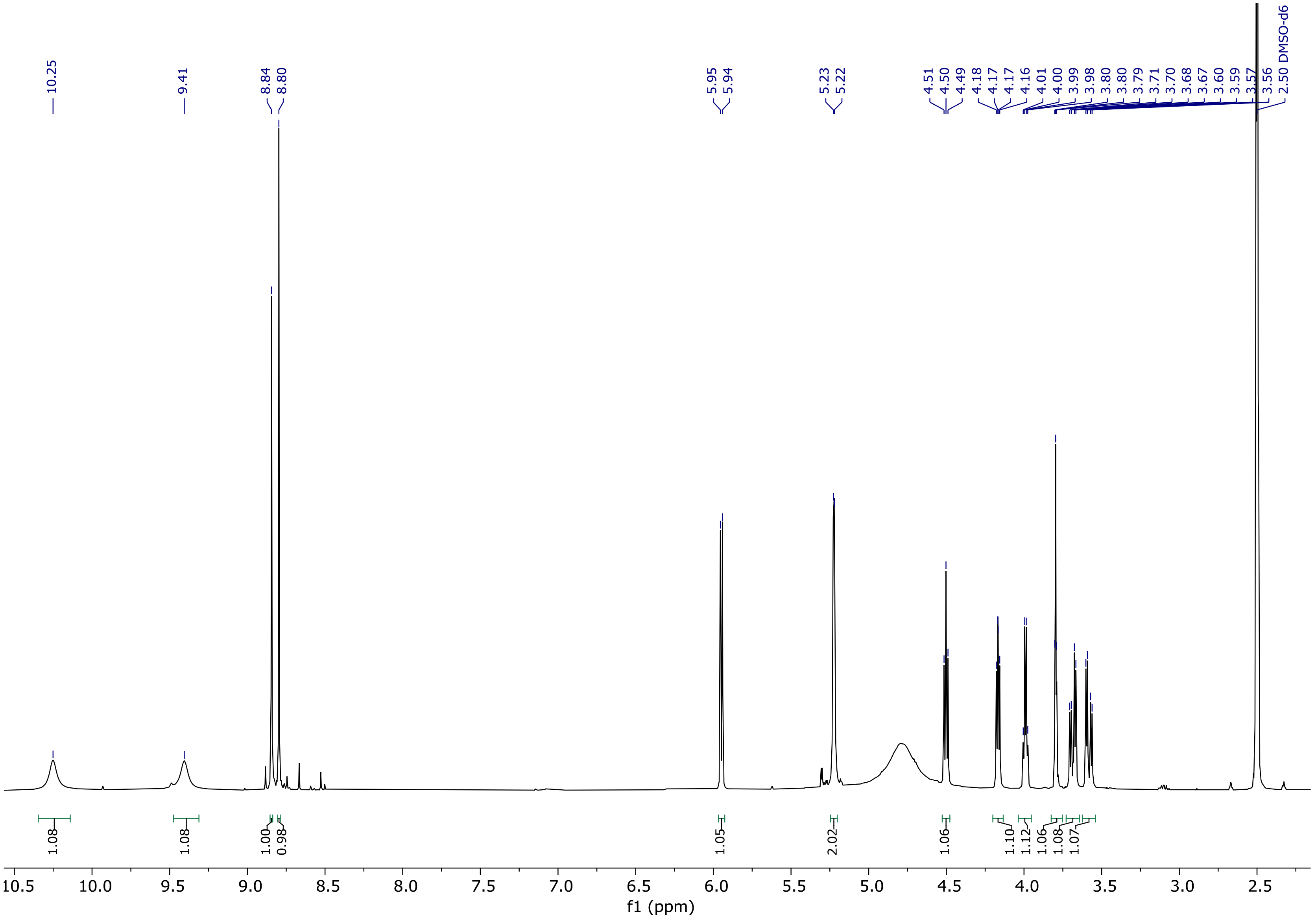

Supplementary Figure 58: ^{1}H-**NMR spectrum of compound 6** (measured on Bruker NEO 400).

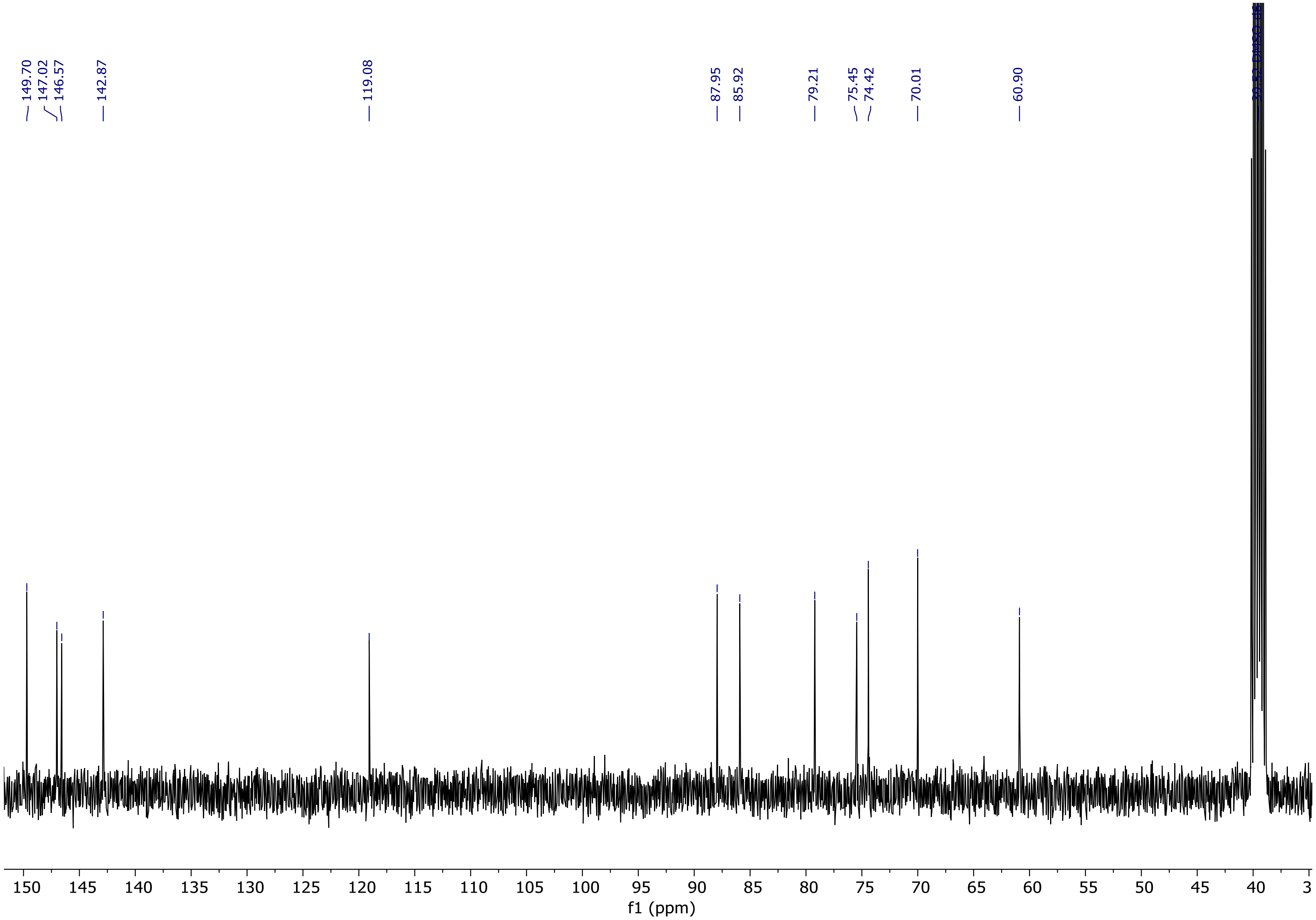

Supplementary Figure 59: **^{13}C-NMR spectrum of compound 6** (measured on Bruker NEO 400).

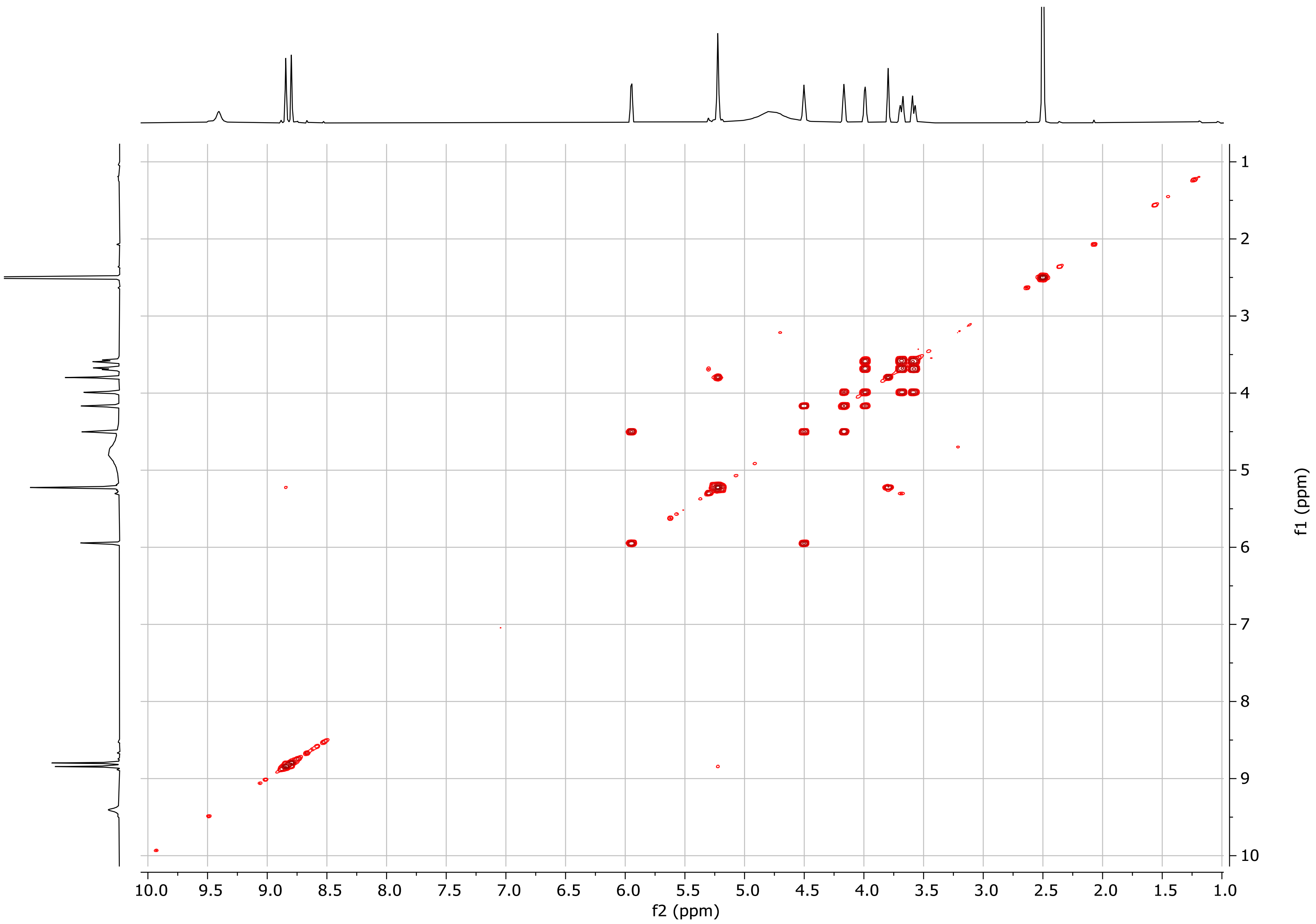

Supplementary Figure 60: **COSY spectrum of compound 6** (measured on Agilent DD2 500).

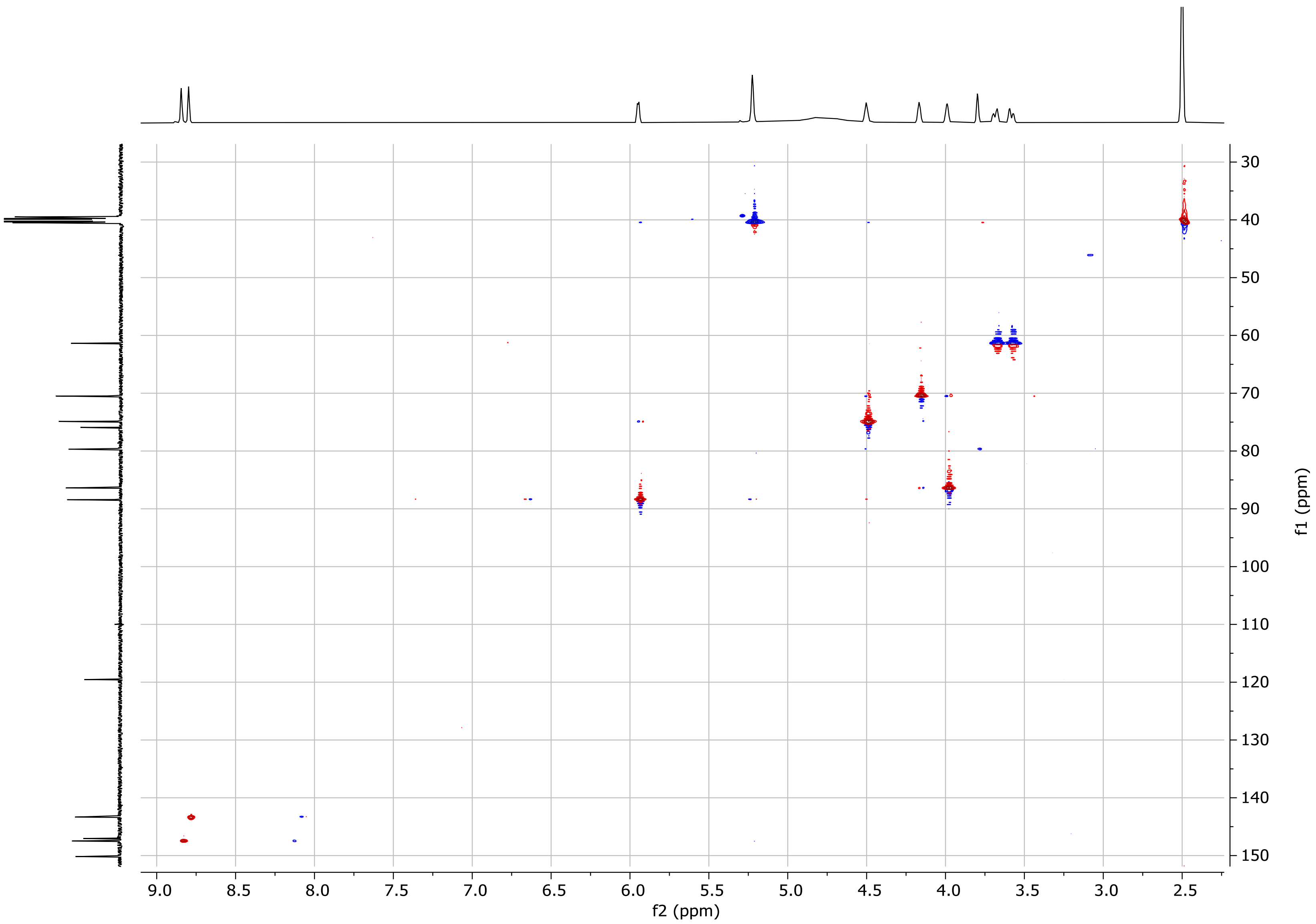

Supplementary Figure 61: **HSQC spectrum of compound 6** (measured on Agilent DD2 500).

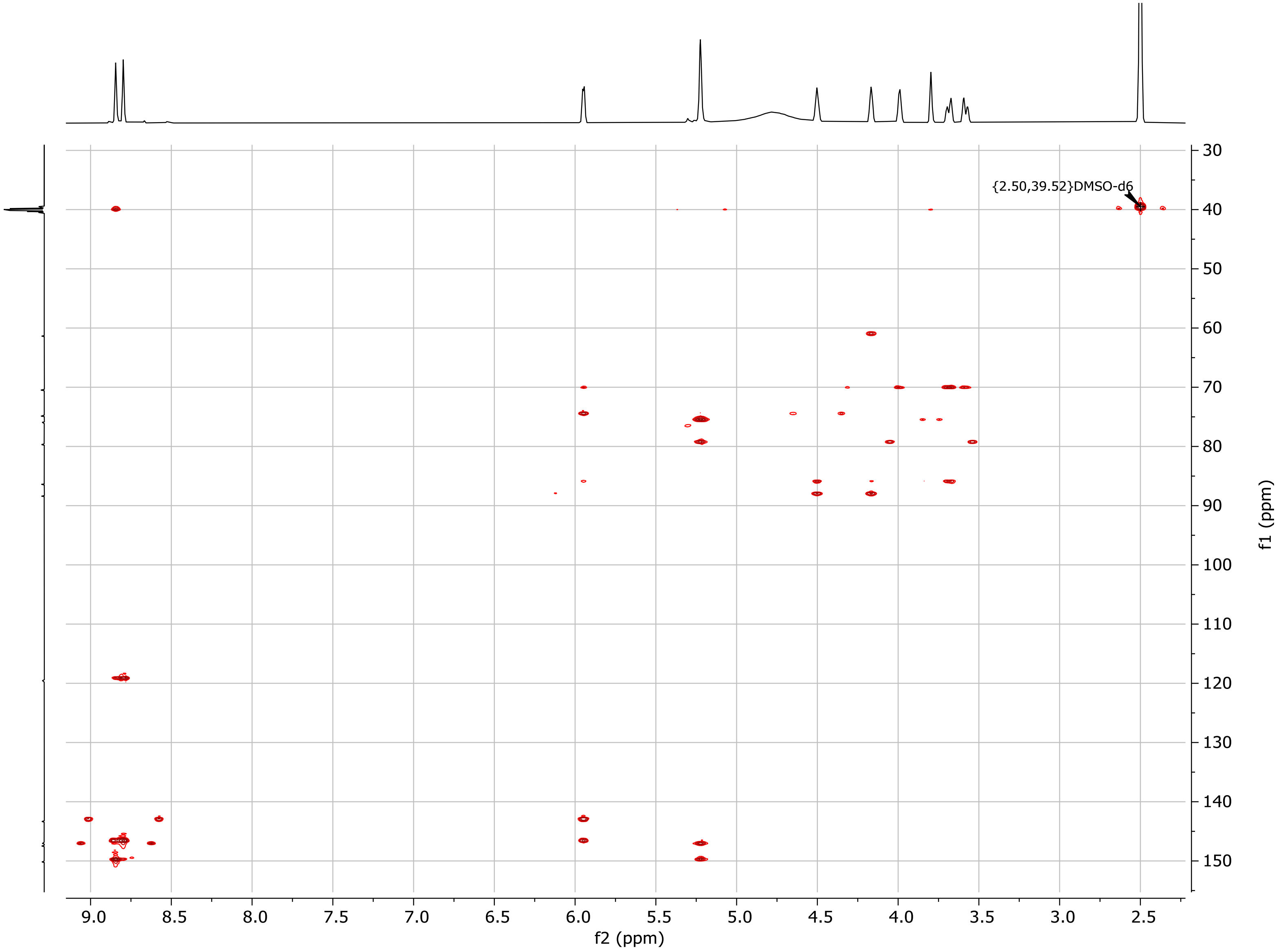

Supplementary Figure 62: **HMBC spectrum of compound 6** (measured on Agilent DD2 500).

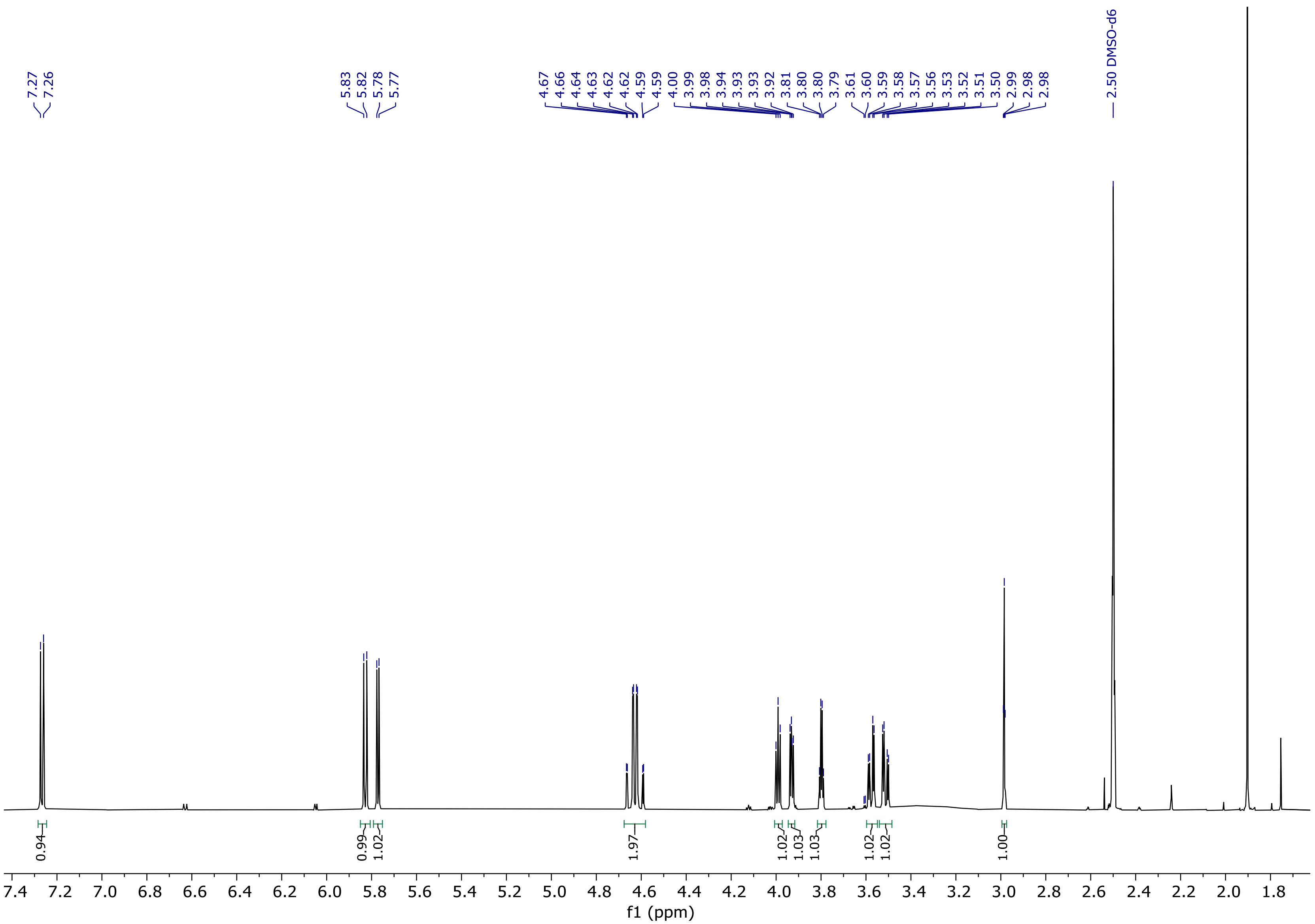

Supplementary Figure 63: **^{1}H-NMR spectrum of compound 7**.

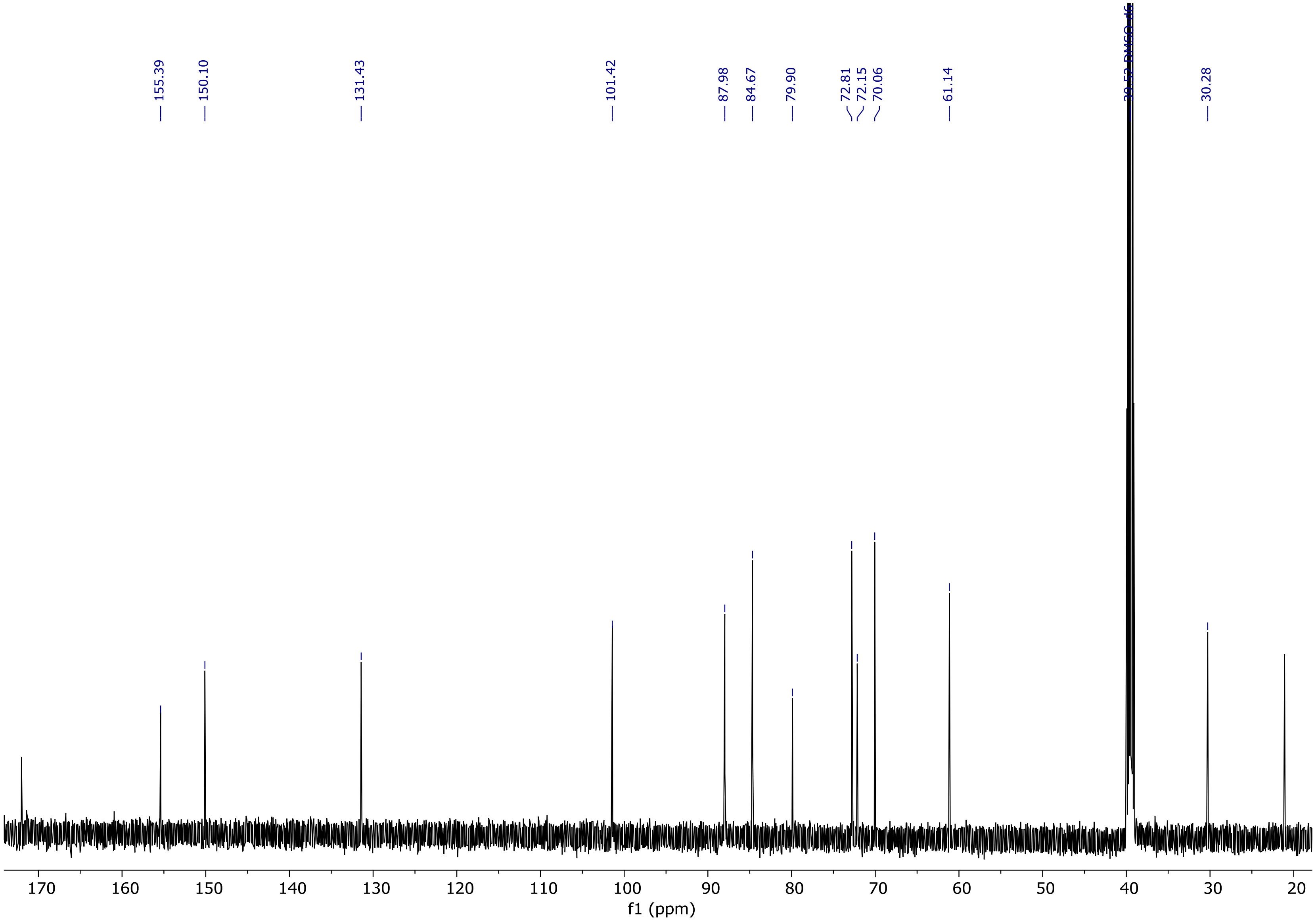

Supplementary Figure 64: **^{13}C-NMR spectrum of compound 7**.

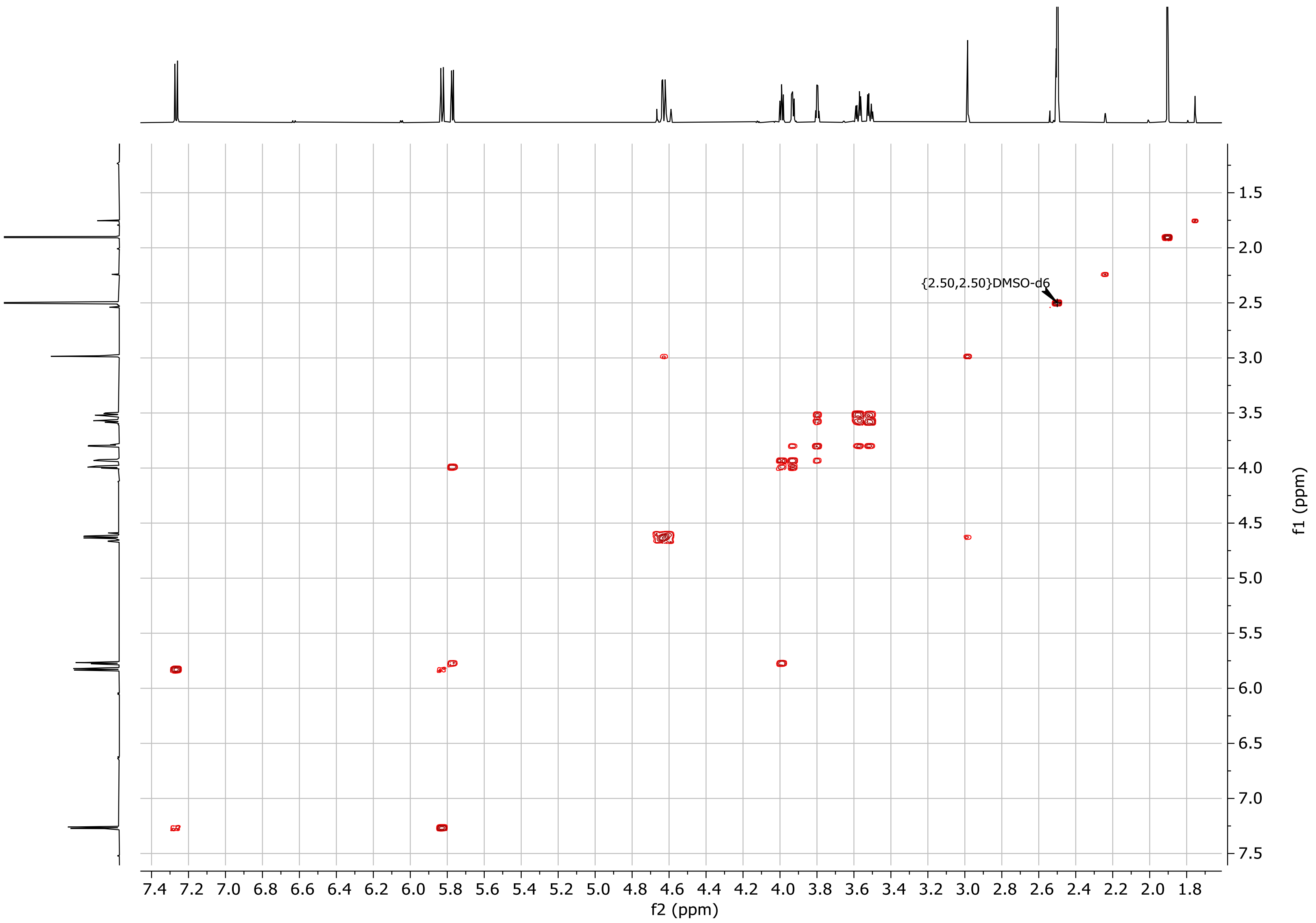

Supplementary Figure 65: **COSY spectrum of compound 7**.

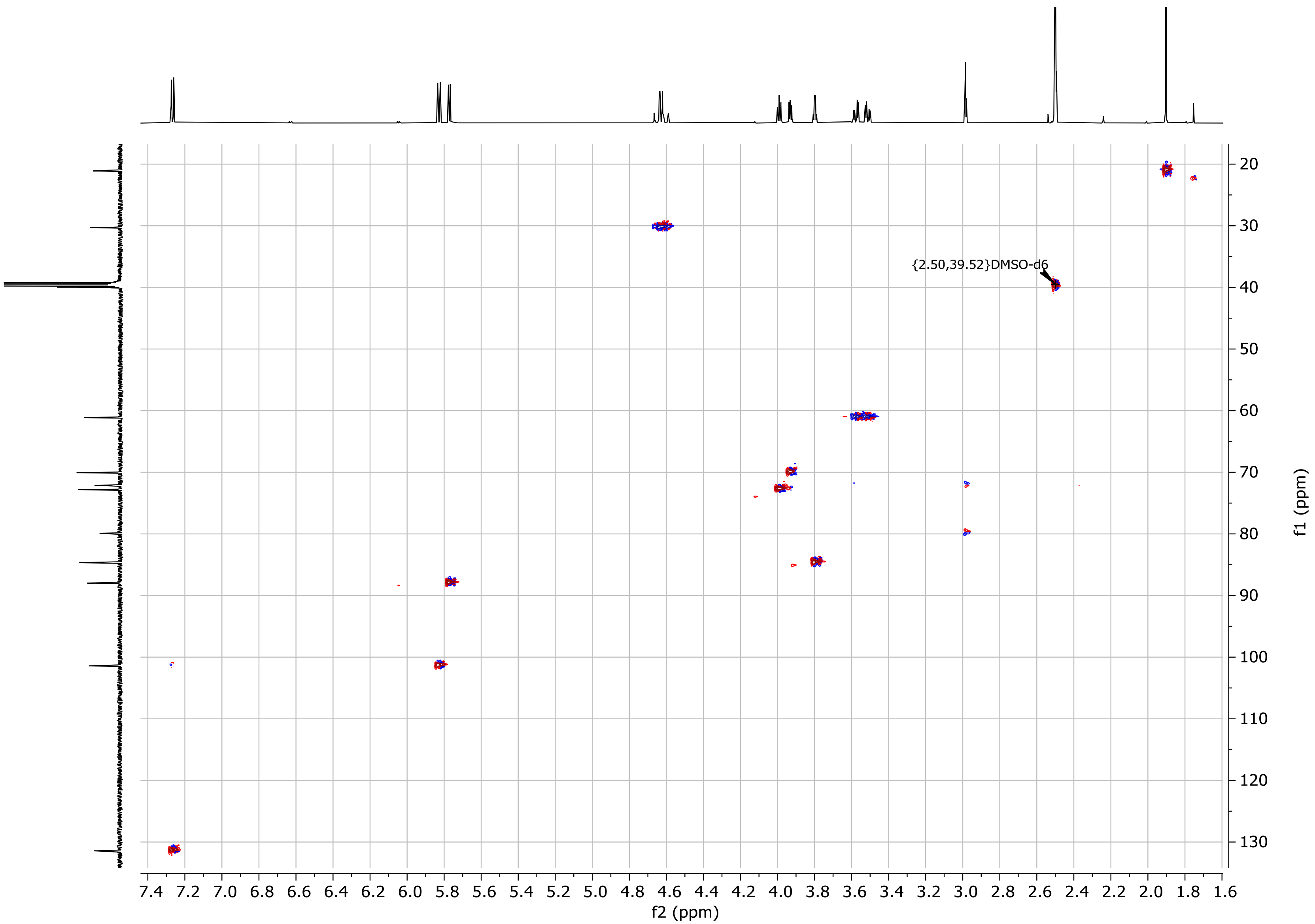

Supplementary Figure 66: **HSQC spectrum of compound 7.**

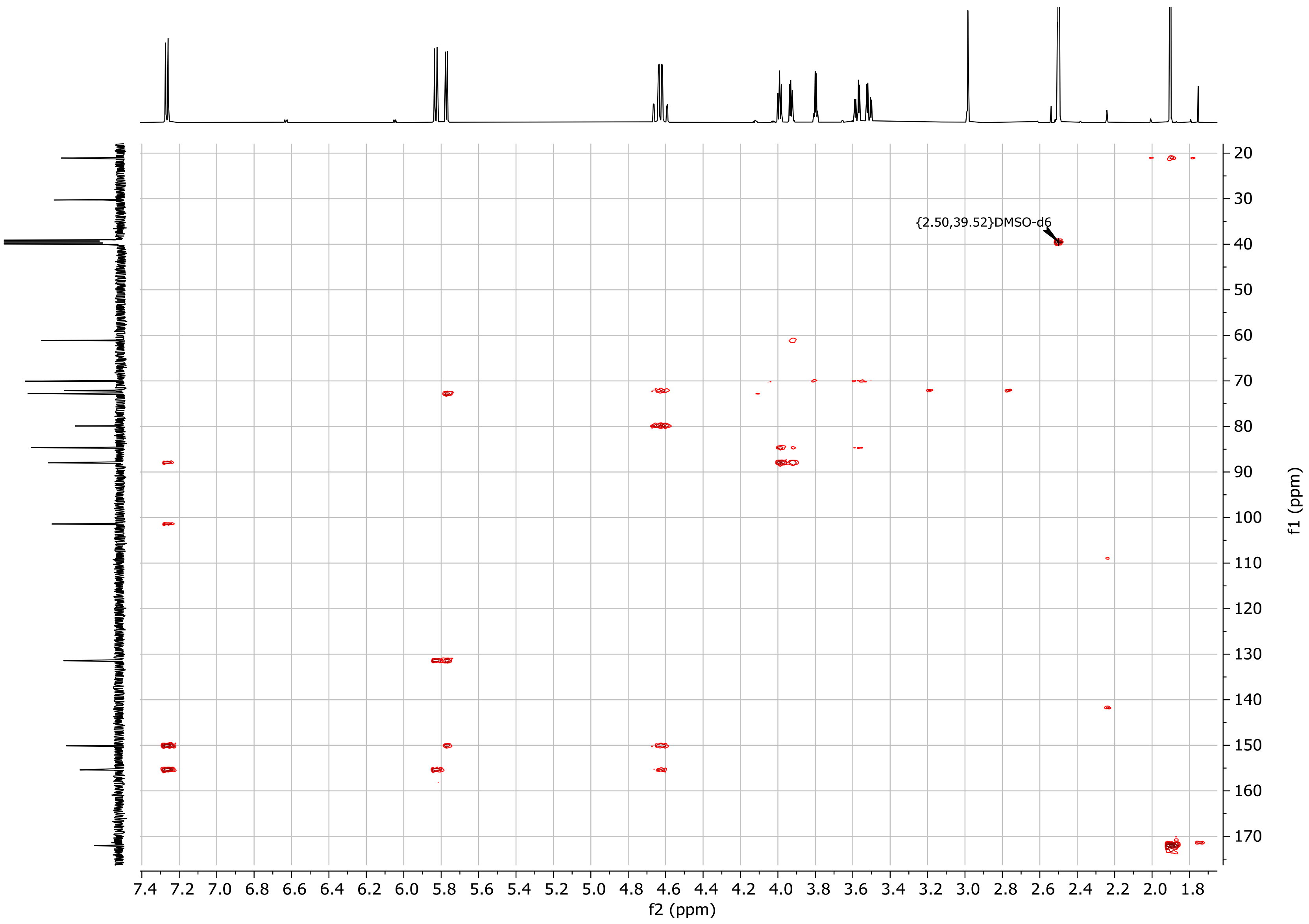

Supplementary Figure 67: **HMBC spectrum of compound 7**.

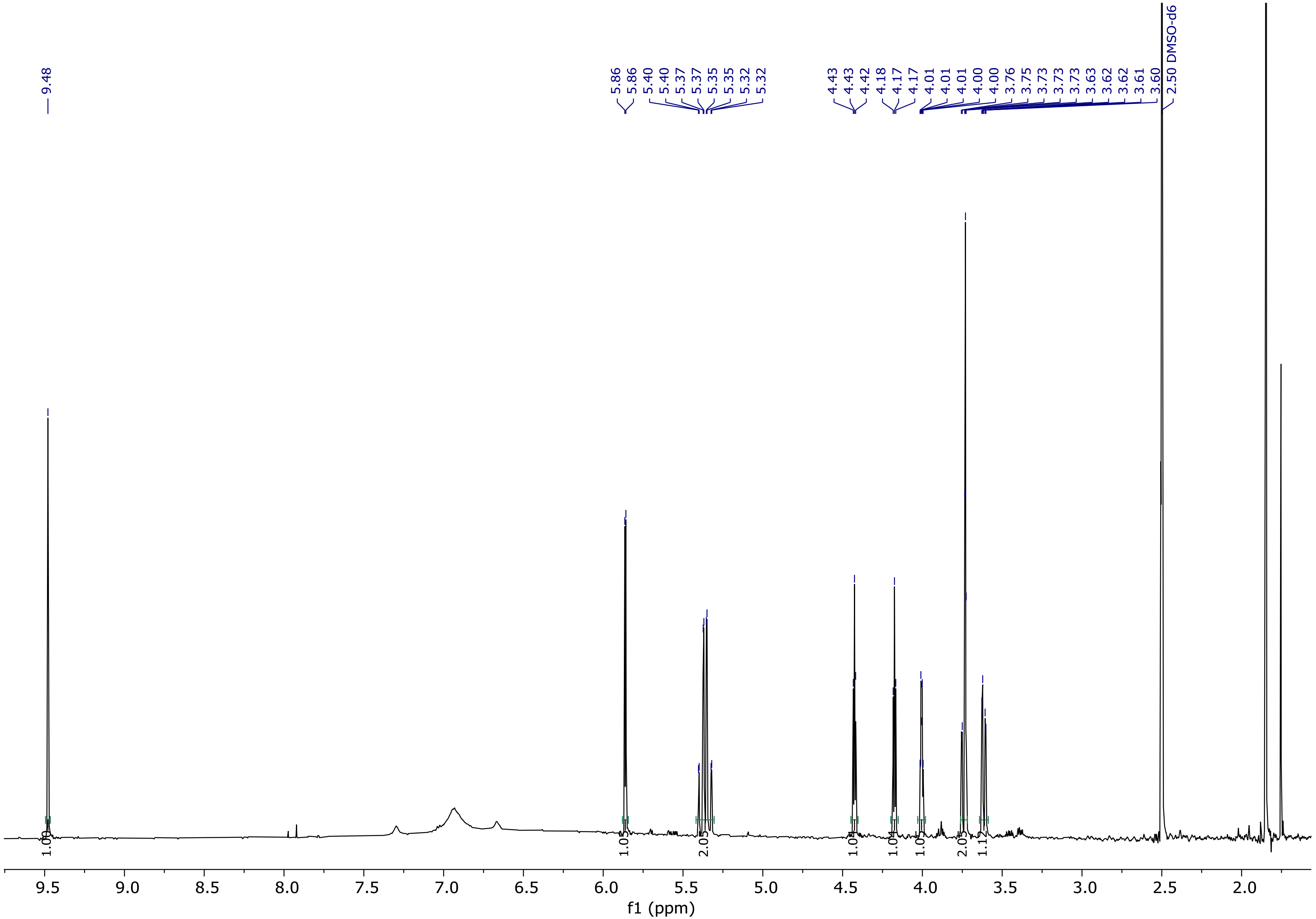

Supplementary Figure 68: **^{1}H-NMR spectrum of compound 8.**

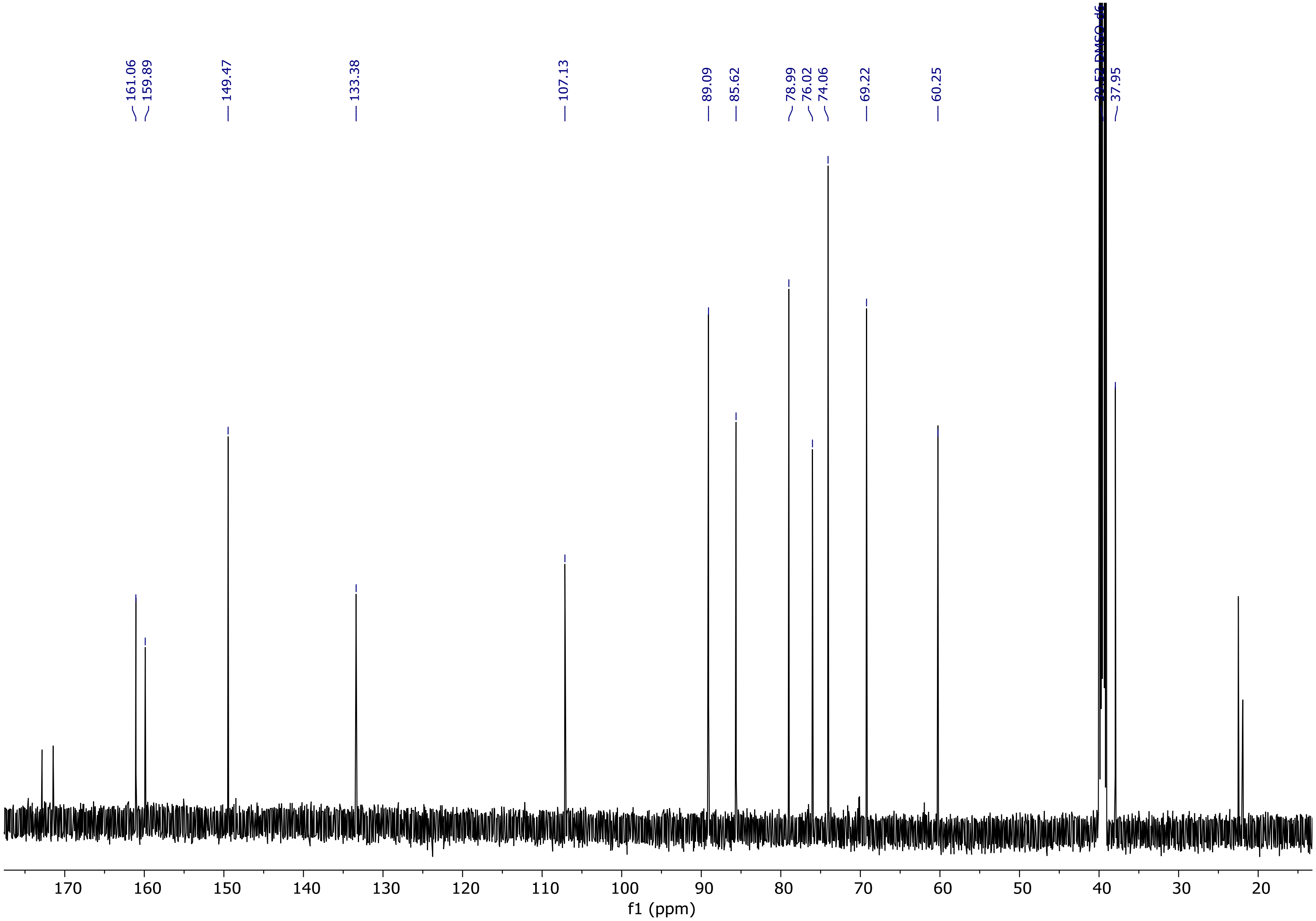

Supplementary Figure 69: **^{13}C-NMR spectrum of compound 8**.

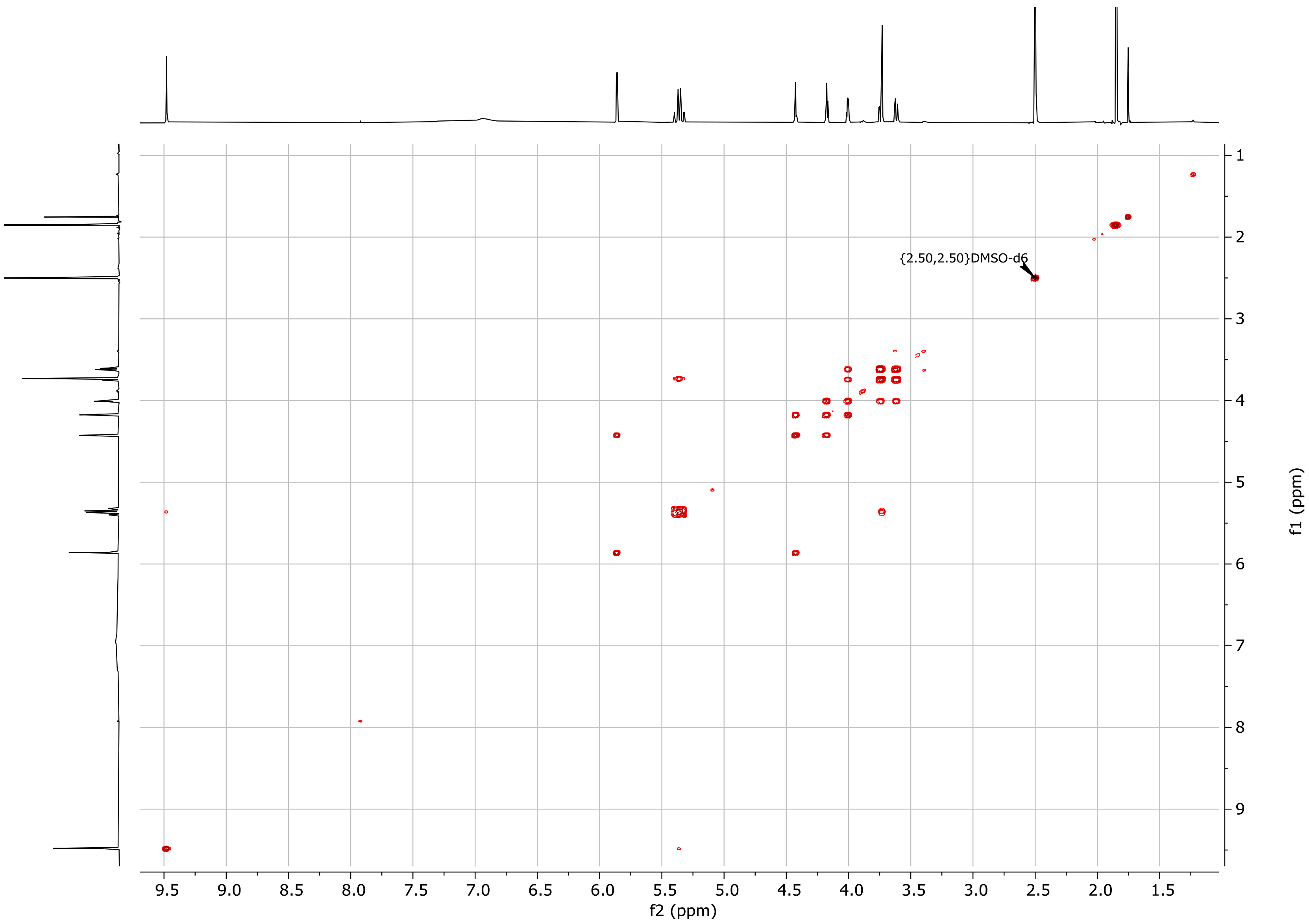

Supplementary Figure 70: **COSY spectrum of compound 8**.

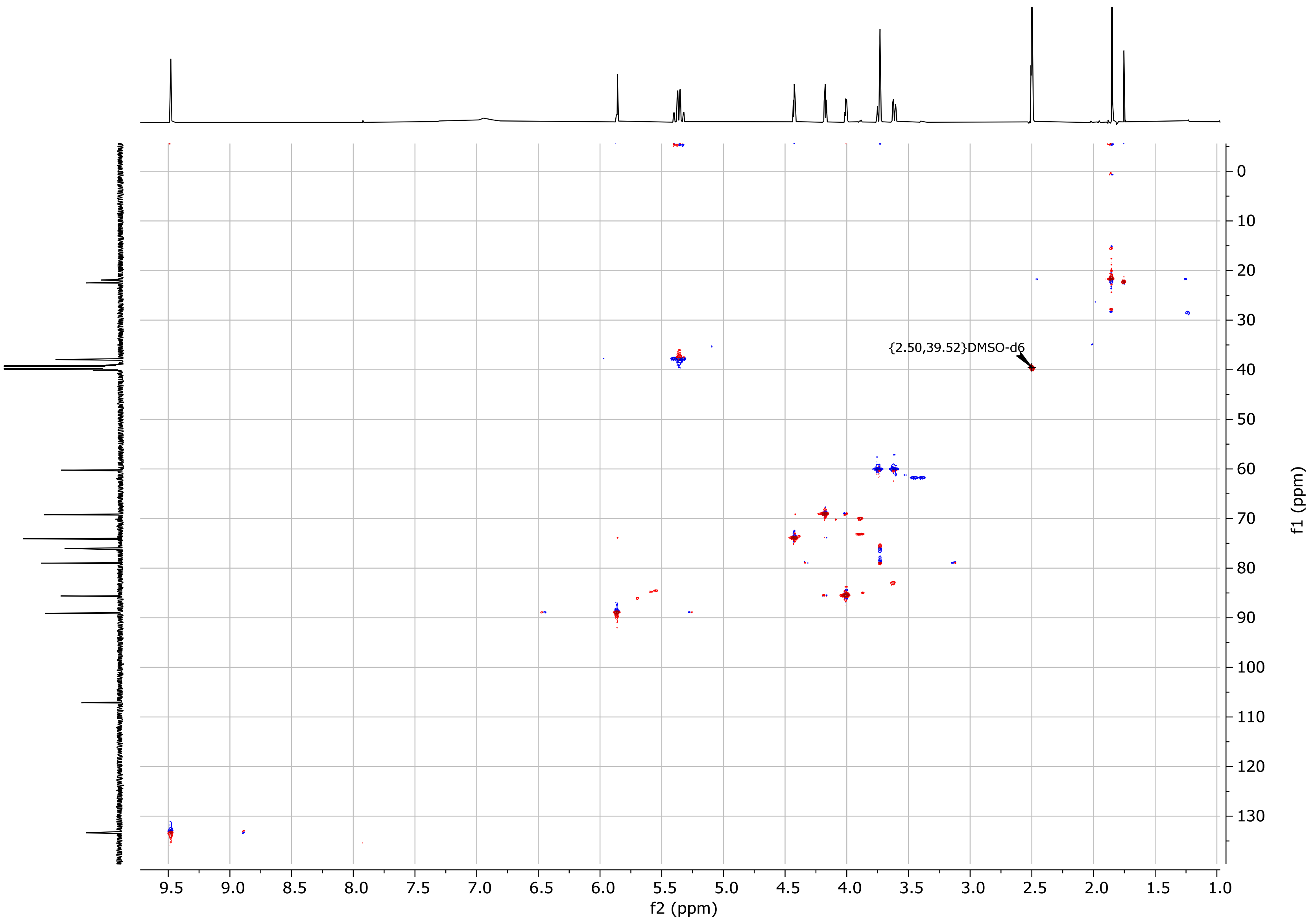

Supplementary Figure 71: **HSQC spectrum of compound 8**.

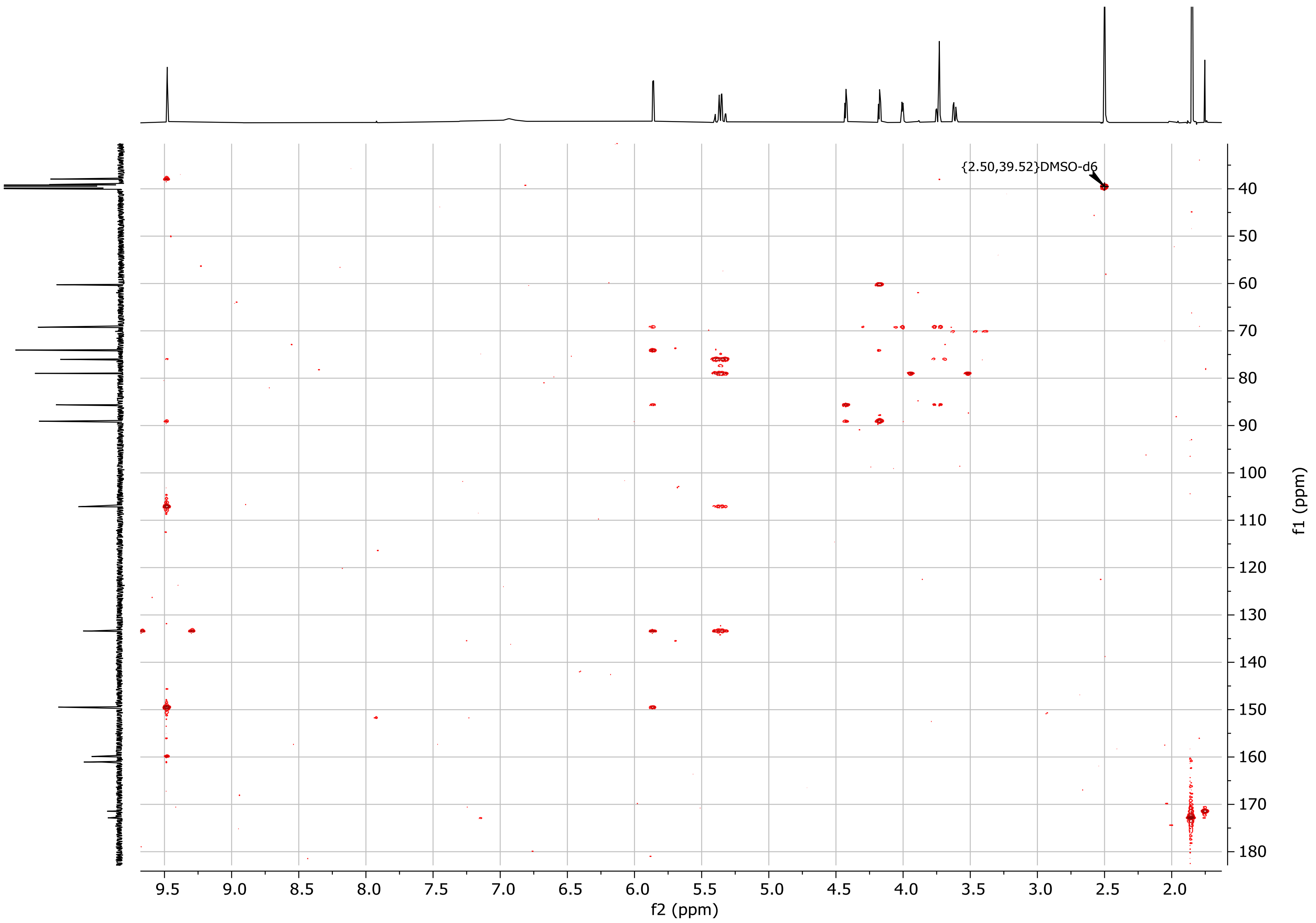

Supplementary Figure 72: **HMBC spectrum of compound 8.**

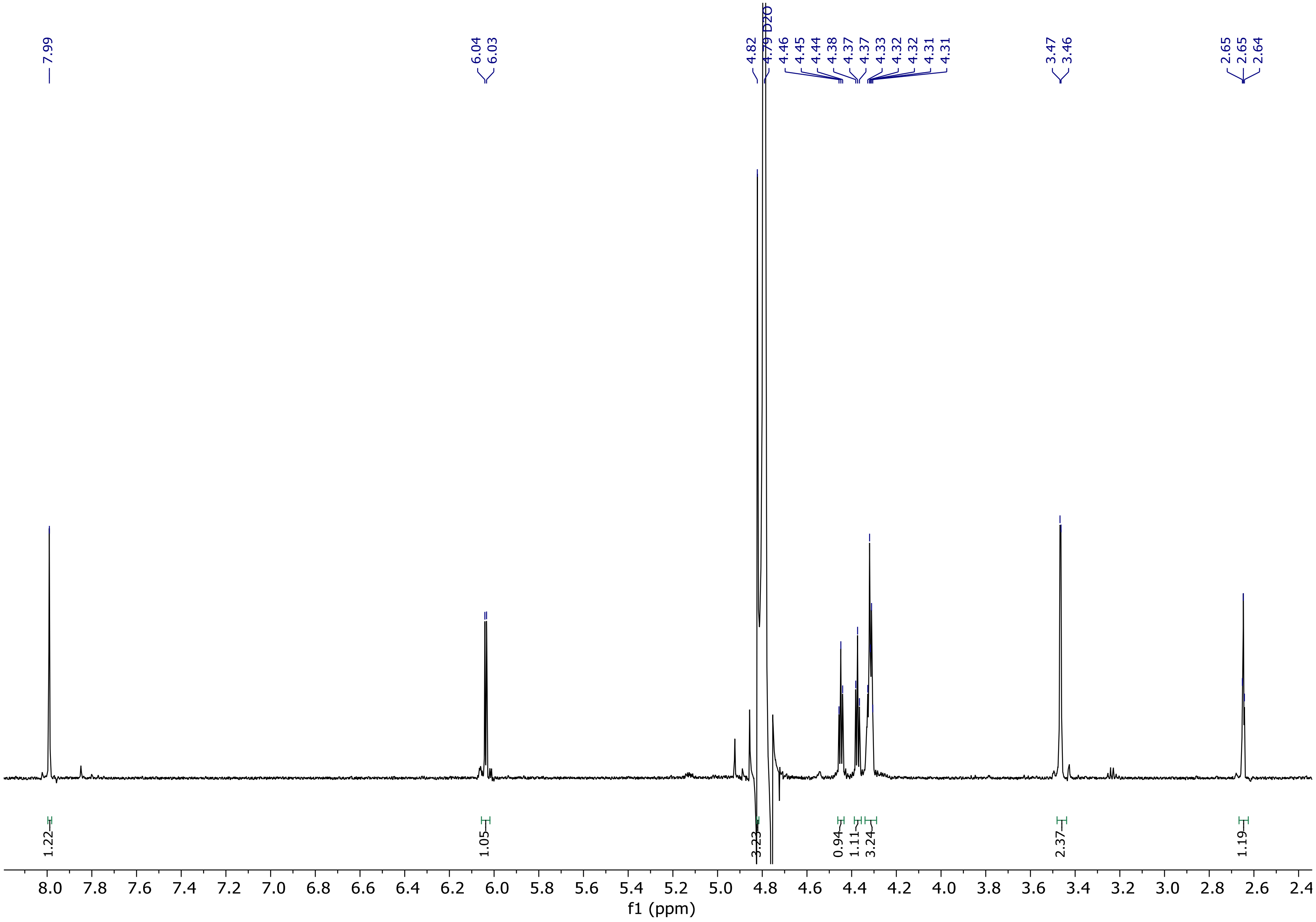

Supplementary Figure 73: **^{1}H-NMR spectrum of compound 9**.

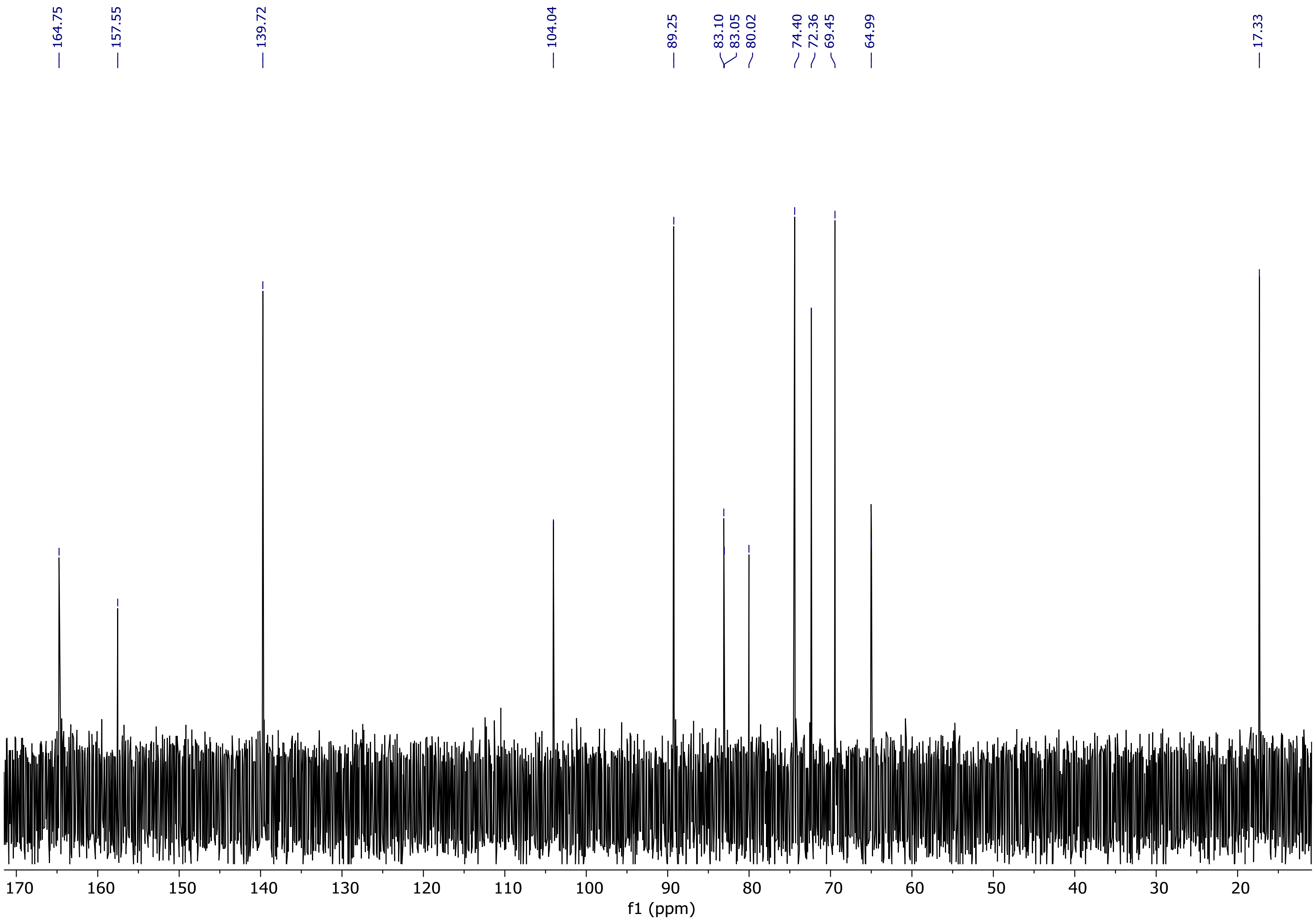

Supplementary Figure 74: **^{13}C-NMR spectrum of compound 9.**

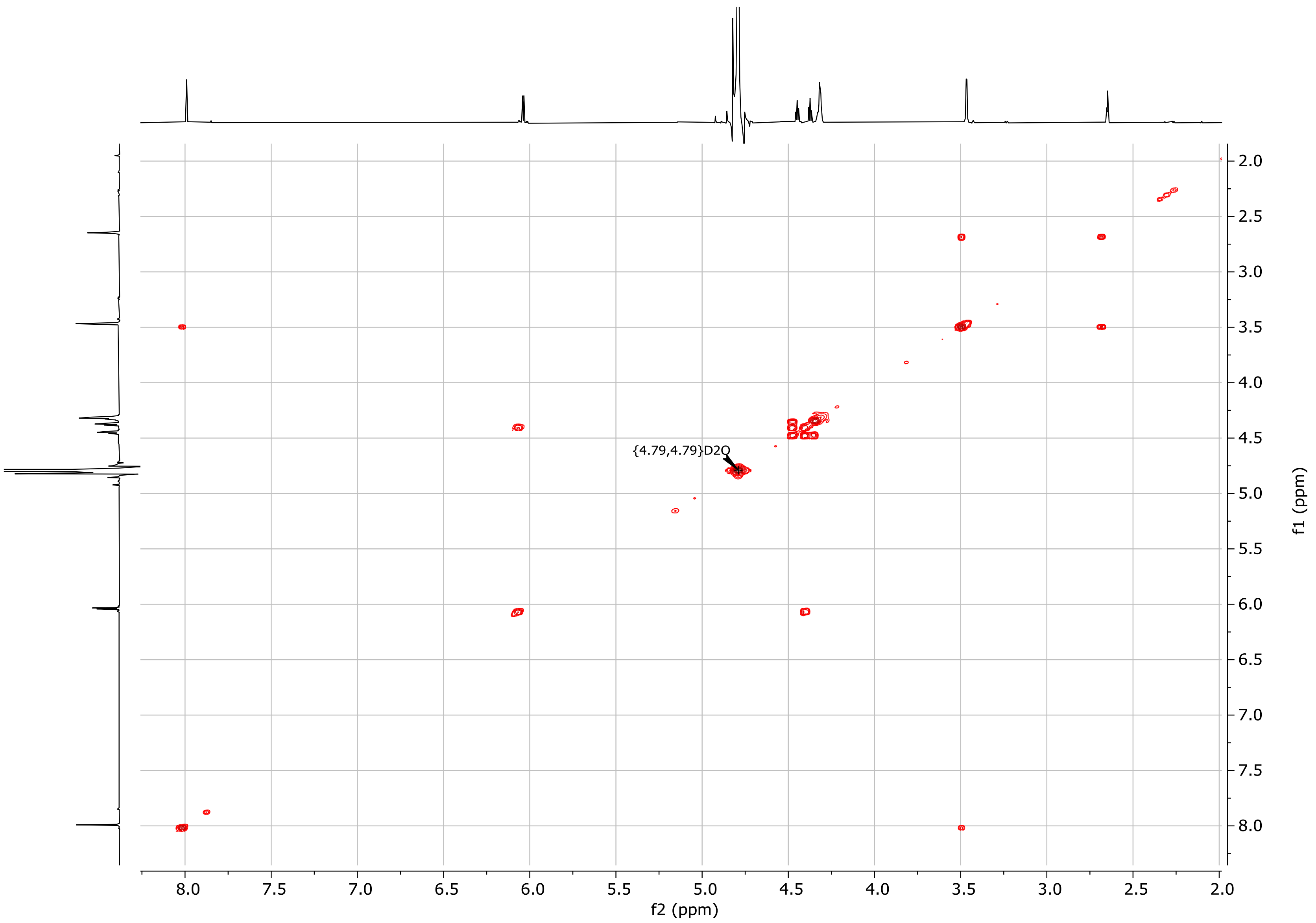

Supplementary Figure 75: **COSY spectrum of compound 9.**

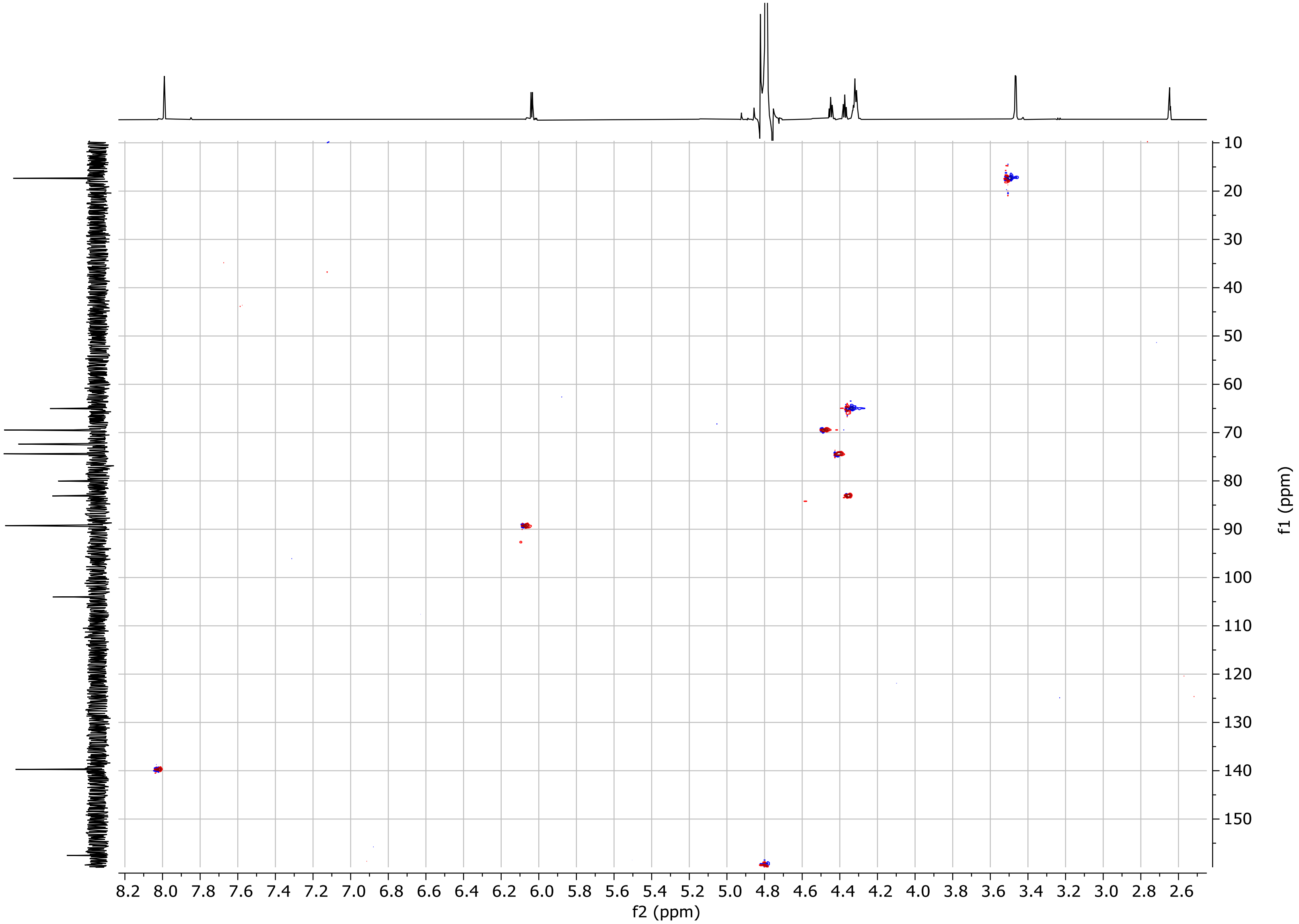

Supplementary Figure 76: **HSQC spectrum of compound 9.**

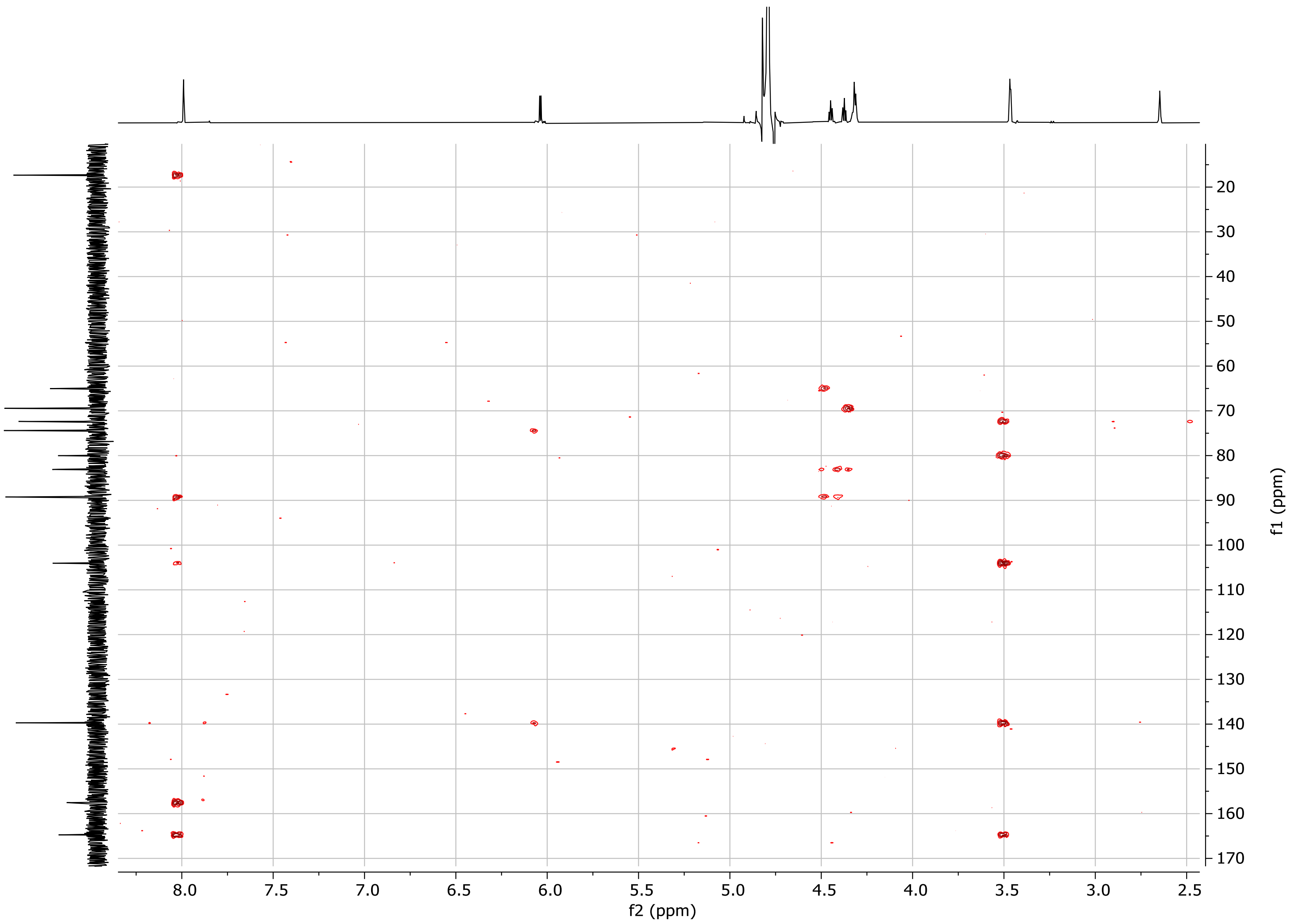

Supplementary Figure 77: **HMBC spectrum of compound 9.**

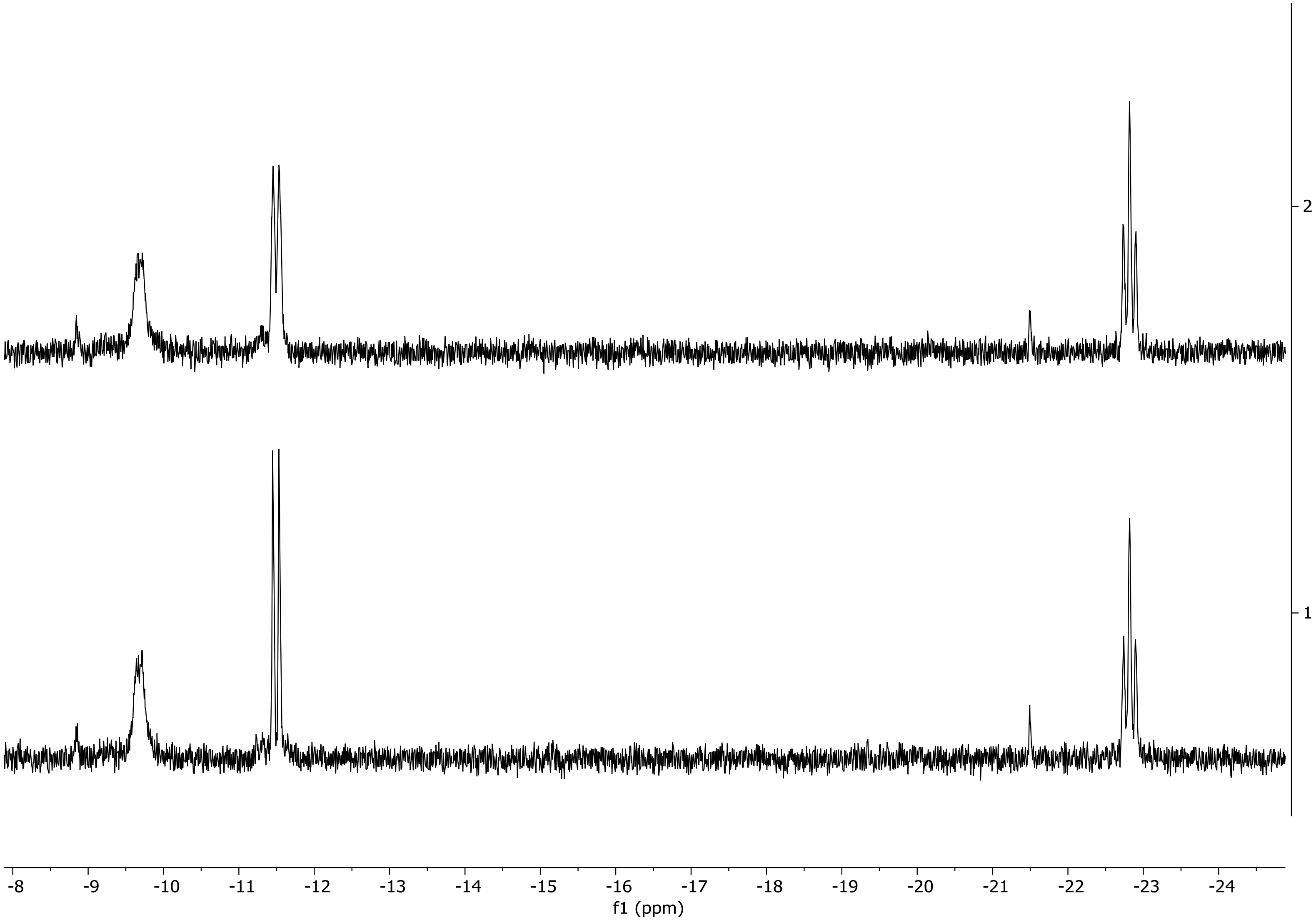

Supplementary Figure 78: **^{31}P-NMR spectrum of compound 9** (1: $^{31}P\{^{1}H\}$ decoupling; 2: no decoupling).

Supplementary Methods:

Synthesis of prop^{5}C, prop^{1}A, prop^{3}C, prop^{7}G and 5-propargylcytidine 5′-triphosphate

Materials and solvents

Unless specified, all reagents were used as received (see Supplementary Table 12) without further purification and solvents were evaporated on a rotary evaporator at 40 °C/2 kPa. Technical grade solvents were used for workup of reactions and for flash chromatography. Benzene (Carl Roth, 7173.1), 1.4-dioxane (Merck, 1.03115.1000) and chloroform (Fisher Scientific, C/4960/15) in analytical grade were used. For RP flash chromatography, the ddH$_2$O and CH$_3$CN (HPLC grade) as eluents were used. If it's mentioned dry DMF, dry DCM or dry THF, the solvents from Supplementary Table 12 were used.

General instrumentation, chromatography materials and methods for synthesis products

^{1}H and ^{13}C NMR spectra were measured on an Agilent DD2 600 spectrometer (600 MHz and 151 MHz), an Agilent DD2 500 spectrometer (500 MHz and 126 MHz) and Bruker NEO 400 (400 MHz and 101 MHz). The measurements were performed in DMSO-*d6* or CDCl$_3$ and referred to residual solvent signal. Complete assignment (if present) is based on heteronuclear correlation experiments HSQC and H, C-HMBC and COSY. Chemical shifts (δ) are in ppm and coupling constants (J) in Hz. The numbering system for the assignment of NMR signals is given for the compounds individually.

High resolution mass spectra were measured on an Orbitrap LTQ XL (Thermo Fisher Scientific) spectrometer using ESI technique and an Orbitrap Velos Pro (Thermo Fisher Scientific) spectrometer using ESI technique.

For determination of low-resolution mass spectra, HPLC - triple-quadrupole mass spectrometry system was used. The system consists of an Agilent 1260 Infinity II with dual λ absorbance detector (G7114A) and an Agilent Ultivo mass spectrometer with JetStream ion source (type of ESI). Column which was used: Agilent Poroshell 120 EC-C18 (3.0x150/2.7µm). The very lipophilic compounds (**1**, **2**, **3** and **4**) were tested by LC method (eluent 20 mM NH$_4$OAc buffer (pH = 6)/CH$_3$CN, gradient 50-100 %, column temperature = 40 °C). For general use (including the compound **5**) the universal LC method (eluent 20 mM NH$_4$OAc buffer (pH = 6)/CH$_3$CN, gradient 0-100 % or 0-60 %, column temperature = 40 °C) was used. For eluents: ammonium acetate, acetic acid, ddH$_2$O and CH$_3$CN, all in LC-MS grade were used.

For flash chromatography, Interchim puriFlash® XS520Plus with multi λ absorbance detector and various types of columns were used: a) 330g column, 50µm spherical silica gel, Interchim [PF-50SIHP-F0330]; b) 120g column, 50µm spherical silica gel, Interchim [PF-50SIHP-F0120]; c) 80g column, 25µm spherical silica gel, Interchim [PF-25SIHC-F0080]. Solid injection (dry load) mode was used for all separation with this technique. We utilized adjustable solid load cartridge system (Teledyne ISCO, 605237048) with various amounts of silica gel (5–25 g). In our experience, solid injection mode improves separation (in comparison with liquid injection) and dramatically increases the lifetime of the pre-packed chromatography columns.

For reversed-phase (RP) flash chromatography, Büchi PrepChrom C-700 multi λ absorbance detector and Teledyne ISCO column RediSepRf® HP C18 Aq GOLD 50g were used. The ddH$_2$O and CH$_3$CN (HPLC grade) were used for this technique.

Synthetic procedures

For synthetic pathway scheme look at Supplementary Figure 31 and 32.

2',3',5'-tris-*O*-(*tert*-butyldimethylsilyl)-5-methyluridine (**1**)

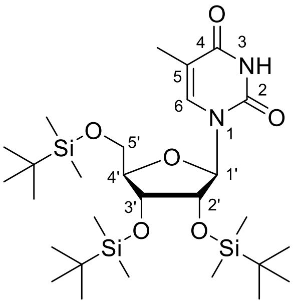

The 5-methyluridine (m^5U) (2.15 g, 8.3 mmol, 1 eq.) and 1*H*-imidazole (5.65 g, 83 mmol, 10 eq.) were suspended in dry DMF (28 mL) under argon. The suspension was cooled by an ice-water bath. *Tert*-Butyldimethylsilyl chloride (5 g, 33.2 mmol, 4 eq.) was added and reaction mixture was stirred overnight to gradually warm up to room temperature. The reaction mixture was transferred to a separation funnel with demineralized water (dH_2O) (150 mL) and extracted by EtOAc (2× 150 mL). The organic layer was extracted by dH_2O (2× 150 mL) and brine (1× 150 mL) and the resulting EtOAc solution was dried by $MgSO_4$, filtered and evaporated *in vacuo*. Raw product was purified via flash chromatography (330g SiO_2 column, eluent cyclohexane:EtOAc(2 % MeOH), gradient 90:10 to 30:70) to obtain the compound **1** as a white solid (4.55 g, 7.57 mmol, yield: 91 %).

^1H NMR (600 MHz, DMSO-d6, ppm): δ 11.38 (s, 1H, NH), 7.40 (s, 1H, H-6), 5.88 (d, *J* = 6.9 Hz, 1H, H-1'), 4.20 (dd, *J* = 6.9, 4.6 Hz, 1H, H-2'), 4.04 (dd, *J* = 4.6, 1.8 Hz, 1H, H-3'), 3.93 – 3.89 (m, 1H, H-4'), 3.82 (dd, *J* = 11.5, 3.8 Hz, 1H, H-5'[a]), 3.71 (dd, *J* = 11.5, 2.9 Hz, 1H, H-5'[b]), 1.78 (s, 3H, C-5-CH_3), 0.94 – 0.75 (m, 27H, Si-C-(CH_3)$_3$), 0.08 (dd, *J* = 14.7, 5.7 Hz, 12H, Si-CH_3), -0.02 (s, 3H, Si-CH_3), -0.10 (s, 3H, Si-CH_3).

^{13}C NMR (151 MHz, DMSO-d6, ppm): δ 163.29 (C-4), 150.63 (C-2), 135.04 (C-6), 109.81 (C-5), 86.05 (C-1'), 85.12 (C-4'), 73.93 (C-2'), 72.23 (C-3'), 62.84 (C-5'), 25.73, 25.57 and 25.42 (Si-C-(CH_3)$_3$), 17.96, 17.63 and 17.47 (Si-C-(CH_3)$_3$), 12.00 (C-5-CH_3), -4.82, -4.85, -4.97, -5.28, -5.62 and -5.64 (Si-CH_3).

HRMS (ESI+) m/z: calculated for $C_{28}H_{56}N_2O_6Si_3$: 623.33384 $[M + Na]^+$, found 623.33366.

Supplementary Fig. 33-37

2',3',5'-tris-*O*-(*tert*-butyldimethylsilyl)-5-(bromomethyl)uridine (**2**)

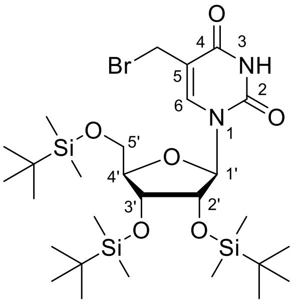

The compound **2** was synthesized according to literature procedure describing the radical bromination of 3'-5'-bis-*O*-(*tert*-butyldimethylsilyl)-thymidine reported by Xiaoxu Li et al. (2018) [1] The uridine derivative **1** (3.12 g, 5.19 mmol, 1 eq.), *N*-bromosuccinimide (1.11 g, 6.24 mmol, 1.2 eq.) and azobisisobutyronitrile (103 mg, 0.63 mmol, 0.12 eq.) were transferred to a round bottom flask. The benzene (55 mL) was added, and reaction mixture was stirred and heated to 80 °C. The reaction mixture was tested by TLC (6:4 / cyclohexane:EtOAc) and indicate the full conversion of the starting material in 1 hour. The solution was cooled to 40 °C and evaporate *in vacuo*. The crude product was purified via flash chromatography (120 g SiO_2 column, eluent DCM:acetone, gradient 0-5 %). The pure fractions were evaporated *in vacuo* and freeze-dried (from 1,4-dioxane) to obtain 1.18 g (yield: 34 %) of compound **2** as a white solid lyophilizate.

^1H NMR (500 MHz, $CDCl_3$): δ 8.51 (s, 1H, NH), 7.86 (s, 1H, H-6), 6.03 (d, *J* = 6.3 Hz, 1H, H-1'), 4.32 (d, *J* = 10.5 Hz, 1H, -CH_2^a-Br), 4.17 (d, *J* = 10.5 Hz, 1H, -CH_2^b-Br), 4.11 (dd, *J* = 6.3, 4.1 Hz, 1H, H-2'), 4.07 – 4.03 (m, 2H, H-3' and H-4'), 3.92 (dd, *J* = 11.6, 2.0 Hz, 1H, H-5'[a]), 3.76 (dd, *J* = 11.6, 1.8 Hz, 1H, H-5'[b]), 0.99, 0.91 and 0.86 (s, 27H, Si-C-(CH_3)$_3$), 0.18 (d, *J* = 1.4 Hz, 6H, Si-CH_3), 0.10, 0.08, 0.03 and -0.02 (s, 12H, Si-CH_3).

^{13}C NMR (126 MHz, $CDCl_3$): δ 161.33 (C-4), 149.86 (C-2), 139.55 (C-6), 112.23 (C-5), 87.77 (d, *J* = 6.7 Hz, C-1'), 86.33 (d, J = 4.8 Hz, C-4'), 75.90 (C-2'), 72.47 (C-3'), 63.30 (C-5'), 26.30, 25.95, 25.85 and 25.79 (Si-C-(CH_3)$_3$), 18.78, 18.22 and 18.06 (Si-C-(CH_3)$_3$), -4.29, -4.35, -4.54, -4.56, -4.98 and -5.23 (Si-CH_3).

The compound **2** was very unstable in the MS analyses. The LC-MS analysis (low resolution) processed in the aqueous buffer (20 mM NH_4OAc, pH = 6.0) showed the mass of hydroxyderivative ($ArCH_2$-OH instead of $ArCH_2$-Br). (ESI+) m/z: calculated for $C_{28}H_{56}N_2O_7Si_3$: 617.3468 $[M + H]^+$, found 617.3. The high resolution MS analysis (Orbitrap XL) processed in MeOH showed only a mass of methyl ether derivative ($ArCH_2$-OCH_3 instead of $ArCH_2$-Br). HRMS (ESI+) m/z: calculated for $C_{29}H_{58}N_2O_7Si_3$: 653.34495 $[M + Na]^+$, found 653.34507. Only the high resolution MS analysis processed in $CHCl_3/CH_3CN$ mixture provided a low abundant but correct molecular ion.
HRMS (ESI+) m/z: calculated for $C_{28}H_{55}BrN_2O_6Si_3$: 701.24435 $[M + Na]^+$, found 701.24460.
Supplementary Fig. 38-42

2',3',5'-tris-*O*-(*tert*-butyldimethylsilyl)-5-propargyluridine (**3**)

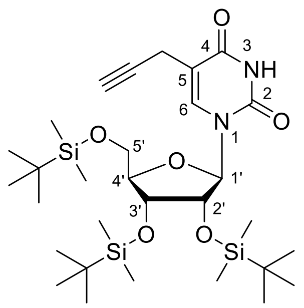

The bromo derivative **2** (900 mg, 1.32 mmol, 1 eq.) and CuCN (39 mg, 0.437 mmol, 0.33 eq.) were dissolved in dry THF (20 mL) in the Schlenk tube under argon atmosphere to form a dark green solution. The solution of ethynylmagnesium bromide in THF (5.8 mL of 0.5 M solution = 2.9 mmol, 2.2 eq.) was added dropwise at room temperature. The color of the solution changed to ochre. The reaction mixture was stirred and heated to 65 °C for 21 hours and then quenched by adding dH_2O (50 mL) and saturated water solution of NH_4Cl (50 mL) to the mixture. The resulting mixture was extracted by EtOAc (2× 100 mL). The organic layer was extracted by dH_2O (2× 200 mL) and brine (1× 200 mL). The resulting EtOAc solution was dried by $MgSO_4$, filtered and evaporated *in vacuo*. Raw product was purified via flash chromatography (120 g SiO2 column, eluent cyclohexane:EtOAc(2 % MeOH), gradient 95:5 to 60:40) to obtain the propargyl derivative **3** as a white solid (600 mg, 0.96 mmol, yield: 72 %).
^{1}H NMR (500 MHz, $CDCl_3$): δ 8.67 (s, 1H, NH), 7.58 – 7.55 (m, 1H, H-6), 5.97 (d, *J* = 6.0 Hz, 1H, H-1'), 4.16 (dd, *J* = 6.0, 4.5 Hz, 1H, H-2'), 4.07 (dd, *J* = 4.5, 2.9 Hz, 1H, H-3'), 4.04 (q, *J* = 3.7 Hz, 1H, H-4'), 3.83 (dd, *J* = 11.3, 4.0 Hz, 1H, H-5'^{a}), 3.76 (dd, *J* = 11.3, 3.5 Hz, 1H, H-5'^{b}), 3.35 – 3.20 (m, 2H, -$\underline{CH_2}$-C≡C), 2.12 (t, *J* = 2.7 Hz, 1H, -C≡C-H), 0.96 – 0.84 (m, 27H, Si-C-($\underline{CH_3}$)$_3$), 0.12 (d, *J* = 1.2 Hz, 6H, Si-$\underline{CH_3}$), 0.09, 0.08, 0.07, 0.06, 0.05, 0.03 and -0.02 (s, 12H, Si-$\underline{CH_3}$).
^{13}C NMR (126 MHz, $CDCl_3$): δ 162.36 (C-4), 150.15 (C-2), 137.52 (d, *J* = 5.4 Hz, C-6), 110.33 (C-5), 88.55 (d, *J* = 7.3 Hz, C-1'), 85.70 (d, *J* = 4.9 Hz, C-4'), 79.96 (-$\underline{C}$≡C-H), 75.03 (C-2'), 72.37 (C-3'), 71.30 (d, *J* = 3.9 Hz, -C≡$\underline{C}$-H), 63.40 (C-5'), 26.30, 26.20, 26.01, 25.96 and 25.85 (Si-C-($\underline{CH_3}$)$_3$), 18.68, 18.21 and 18.07 (Si-$\underline{C}$-(CH_3)$_3$), 16.59 (-$\underline{C}H_2$-C≡C), -4.30, -4.37, -4.56, -4.66, -5.15 and -5.19 (Si-$\underline{CH_3}$).
HRMS (ESI+) m/z: calculated for $C_{30}H_{56}N_2O_6Si_3$: 663.30778 $[M + K]^+$ and 647.33384 $[M + Na]^+$, found 663.30819 and 647.33439.
Supplementary Fig. 43-47

2',3',5'-tris-*O*-(*tert*-butyldimethylsilyl)-5-propargylcytidine (**4**)

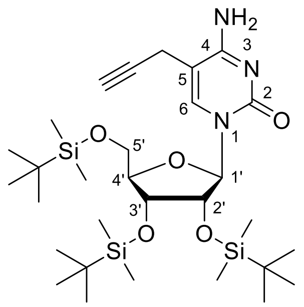

For the preparation of compound **4**, the procedure describing the conversion of a uridine derivative to a cytidine derivative reported by Qi Sun et al. [2] was considerably modified. The propargyl derivative **3** (900 mg, 1.44 mmol, 1 eq.) was dissolved in dry DCM (20 mL) under argon. *N*-Methylpiperidine (460 µL, ~ 2.6 eq.) and triethylamine (Et_3N, 460 µL, ~ 2.3 eq.) were added. Subsequently, 4-methylbenzene-1-sulfonyl chloride (800 mg, 4.2 mmol, 2.9 eq.) in dry DCM (6 mL) was added dropwise. The reaction mixture was stirred at room temperature for 3.5 hours and then cooled by an ice-water bath. The solution of NH_3 (25 mL of 7N NH_3 in MeOH) was added and formation of a temporary precipitate was observed. The solution was stirred for 2 hours and then transferred to a separation funnel with $dH_2O:CHCl_3$ (400 mL, 1:1) and extracted. The organic

layer was extracted by dH_2O (4×) and brine (1×) and evaporated *in vacuo*. The crude product was purified via flash chromatography (80 g SiO_2 column neutralized by DCM (2 % Et_3N), eluent cyclohexane:EtOAc (2 %MeOH):MeOH, gradient from 90:10:0 to 45:45:10). The pure fractions were evaporated *in vacuo* and freeze-dried (from 1,4-dioxane) to obtain 333 mg (yield: 37 %) of pure compound **4** as a white solid lyophilizate.

^{1}H NMR (600 MHz, $CDCl_3$): δ 7.72 (m, 1H, H-6), 5.91 (d, *J* = 4.0 Hz, 1H, H-1'), 4.15 (t, *J* = 4.1 Hz, 1H, H-2'), 4.08 (dt, *J* = 5.7, 3.0 Hz, 1H, H-4'), 3.99 (dd, *J* = 5.2, 4.3 Hz, 1H, H-3'), 3.96 (dd, *J* = 11.5, 2.9 Hz, 1H, H-5'^{a}), 3.78 (dd, *J* = 11.5, 3.1 Hz, 1H, H-5'^{b}), 3.27 (d, *J* = 2.7 Hz, 2H, -CH_2-C≡C), 2.20 (t, *J* = 2.7 Hz, 1H, -C≡C-H), 0.96, 0.90 and 0.88 (s, 27H, Si-C-(CH_3)$_3$), 0.14, 0.13, 0.08, 0.07, 0.06, and -0.05 (s, 18H, Si-CH_3).

^{13}C NMR (151 MHz, $CDCl_3$): δ 139.88 (C-6), 101.23 (C-5), 89.89 (C-1'), 84.48 (C-4'), 78.65 (-C≡C-H), 75.98 (C-2'), 72.31 (-C≡C-H), 71.26 (C-3'), 62.77 (C-5'), 26.31, 26.01 and 25.99 (Si-C-(CH_3)$_3$), 18.83, 18.32 and 18.16 (Si-C-(CH_3)$_3$), 18.22 (-CH_2-C≡C), -4.08, -4.31, -4.70, -4.73, -4.89 and -5.21 (Si-CH_3).

HRMS (ESI+) m/z: calculated for $C_{30}H_{57}N_3O_5Si_3$: 646.34982 $[M + Na]^+$, found 646.35015.

Supplementary Fig. 48-52

5-Propargylcytidine (**5**) (prop^{5}C)

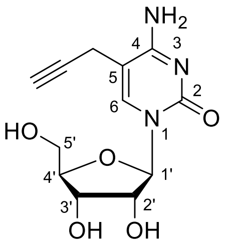

For removing the *tert*-butyldimethylsilyl protection groups, the procedure described by Venkatesham et al. [3] was adapted. The propargyl derivative **4** (22 mg, 35 µmol, 1 eq.) was dissolved in MeOH (4 mL) and NH_4F was added (18 mg, 486 µmol, 14 eq.). Reaction vessel was closed by a screw cap and the reaction mixture was stirred and heated to 65 °C (oil bath). The reaction mixture was repeatedly tested by LC-MS (50 µL of reaction mixture diluted in 1 mL MeOH, 6 µL injection) and indicated almost full conversion of the starting material in 22 hour. The reaction was stopped after 27 hours. The solvent was evaporated *in vacuo*, residue was suspended in DMSO (1 mL) and injected onto a chromatographic column and purified by RP flash chromatography (C18-aq 50 g; eluent ddH_2O/CH_3CN, gradient 0–20 %). The pure fraction was freeze-dried to obtain 3 mg (yield: 30 %) of compound **5** as a white lyophilizate. The purity was tested by LC-MS.

^{1}H NMR (500 MHz, DMSO-d_6): δ 7.81 (s, 1H, H-6); 5.79 (d, *J* = 4.6 Hz, 1H, H-1'); 5.28 (d, *J* = 5.3 Hz, 1H, -OH-2'); 4.98 (s, 2H, -OH-3' and -OH-4'); 3.97 – 3.93 (m, 1H, H-2'); 3.93 – 3.88 (m, 1H, H-3'); 3.83 (dt, *J* = 4.8, 3.6 Hz, 1H, H-4'); 3.64 (d, *J* = 11.9 Hz, 1H, H-5'^{a}); 3.54 (d, J = 11.9 Hz, 1H, H-5'^{b}); 3.22 (dd, *J* = 2.7, 1.0 Hz, 2H, -CH_2-C≡C); 3.03 (t, *J* = 2.6 Hz, 1H, -C≡C-H).

^{13}C NMR (126 MHz, DMSO-d_6): δ 163.84 (C-4); 155.08 (C-2); 139.28 (C-6); 100.85 (C-5); 89.12 (C-1'); 84.21 (C-4'); 80.24 (-C≡C-H); 73.92 (-C≡C-H); 73.83 (C-2'); 69.79 (C-3'); 61.03 (C-5'); 16.99 (-CH_2-C≡C).

HRMS (ESI+) m/z: calculated for $C_{12}H_{15}N_3O_5$: 304.09039 $[M + Na]^+$, found 304.09049.

Supplementary Fig. 53-57

*N*1-propargyladenosine (**6**) (prop^{1}A)

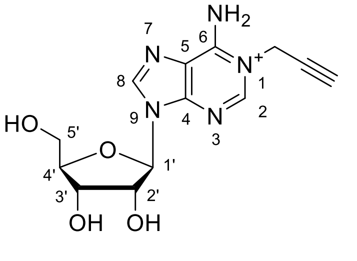

Under argon adenosine (0.5 g, 1.8 mmol, 1.0 eq) was dissolved in dry DMF (20 mL) and stirred at 40 °C until the solution became clear. Then propargyl bromide (0.7 mL, 3.2 mmol, 5.0 eq) was added dropwise over 5 min and stirred at 40 °C for 20 h. The crude mixture was then concentrated and the resulting syrup was dissolved in DMSO (5 mL) to purify it by reversed-phase (RP) flash chromatography (aq-C18 column 150 g; eluents ddH_2O/CH_3CN, gradient 0–55 % of CH_3CN, over 15 min with a flow rate of 65 mL/min). The pure fractions were combined and freeze dried to obtain 90 mg (yield: 18 %) of compound **6** as a white solid. The purity was tested by LC-MS.

^{1}H NMR (400 MHz, DMSO-d_6) δ 8.84 (s, 1H, H-2); 8.80 (s, 1H, H-8); 5.95 (d, *J* = 5.2 Hz, 1H, H-1'); 5.22 (d, 2H, *J* = 2.5 Hz, -$\underline{CH_2}$-C≡C); 4.50 (t, 1H, *J* = 5.1 Hz, H-2'); 4.17 (dd, 1H, *J* = 4.9, 3.9 Hz, H-3'); 3.99 (q, 1H, *J* = 3.9 Hz, H-4'); 3.82 – 3.75 (t, 1H, , -C≡C-H); 3.69 (dd, 1H, *J* = 12.0, 4.1 Hz, H-5'^{a}); 3.58 (dd, 1H, *J* = 12.0, 3.9 Hz, H-5'^{b}).
^{13}C NMR (101 MHz, DMSO-d_6) δ 150.17, 147.49 (C-2); 147.04; 143.34 (C-8); 119.55 (C-5); 88.42 (C-1'); 86.39 (C-4'); 79.68 (-C≡$\underline{C}$-H); 75.92 (-$\underline{C}$≡C-H); 74.89 (C-2'); 70.48 (C-3'); 61.36 (C-5'); 40.41 (-$\underline{C}H_2$-C≡C).
HRMS (ESI+) m/z: calculated for $C_{13}H_{16}N_5O_4^+$: 306.11968 [M]$^+$, found 306.11973.
Supplementary Fig. 58-62

*N*3-propargylcytidine (**7**) (prop^{3}C)

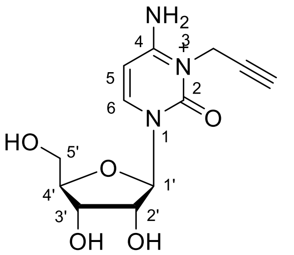

Cytidine (122 mg, 0.5 mmol, 1.0 eq) was dissolved in DMSO (2.5 mL) and propargyl bromide (64 µL, 0.6 mmol, 1.2 eq) was slowly added over 5 min. The solution was vigorously stirred at room temperature for 16 h. The reaction mixture was purified by preparative HPLC. The pure fraction was freeze dried to obtain 23.1 mg (yield: 16 %) of compound **7** as a beige solid. The purity was tested by LC-MS
^{1}H NMR (600 MHz, DMSO-*d6*): δ 7.27 (d, 1H, *J* = 8.2 Hz, H-6); 5.83 (d, 1H, *J* = 8.1 Hz, H-5); 5.77 (d, 1H, *J* = 5.9 Hz, H-1'); 4.68 – 4.58 (m, 2H, -$\underline{CH_2}$-C≡C); 3.99 (t, 1H, *J* = 5.6 Hz, H-2'); 3.93 (dd, 1H, *J* = 5.2, 3.6 Hz, H-3'); 3.80 (q, 1H, *J* = 3.5 Hz, H-4'); 3.58 (dd, 1H, *J* = 11.9, 3.5 Hz, H-5'^{a}); 3.51 (dd, 1H, *J* = 12.0, 3.6 Hz, H-5'^{b}); 2.98 (t, 1H, *J* = 2.4 Hz, -C≡C-H).
^{13}C NMR (151 MHz, DMSO-*d6*): δ 155.39 (C-4); 150.10 (C-2); 131.43 (C-6); 101.42 (C-5); 87.98 (C-1'); 84.67 (C-4'); 79.90 (-C≡$\underline{C}$-H); 72.81 (C-2'); 72.15 (-$\underline{C}$≡C-H); 70.06 (C-3'); 61.14 (C-5'); 30.28 (-$\underline{C}H_2$-C≡C).
HRMS (ESI+) m/z: calculated for $C_{12}H_{15}N_3O_5$: [M+H]$^+$ 282.10845 and 304.09039 [M+Na]$^+$, found 282.10812 and 304.09006.
Supplementary Fig. 63-67

*N*7-propargylguanosine (**8**) (prop^{7}G)

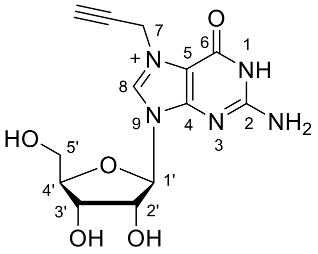

Guanosine (142 mg, 0.5 mmol, 1.0 eq) was dissolved in DMSO (2.5 mL) and propargyl bromide (64 µL, 0.6 mmol, 1.2 eq) was slowly added over 5 min. The solution was vigorously stirred at room temperature for 16 h. The reaction mixture was purified by preparative HPLC. The pure fraction was freeze dried to obtain 33.5 mg (yield: 18 %) of compound **8** as a colorless solid. The purity was tested by LC-MS
^{1}H NMR (600 MHz, DMSO-*d6*): δ 9.48 (s, 1H, H-8); 5.86 (d, 1H, *J* = 4.0 Hz, H-1'); 5.42 – 5.30 (m, 2H, -$\underline{CH_2}$-C≡C); 4.43 (t, 1H, *J* = 4.4 Hz, H-2'); 4.17 (t, 1H, *J* = 4.9 Hz, H-3'); 4.01 (dt, 1H, *J* = 5.8, 3.1 Hz, H-4'); 3.74 (dd, 1H, *J* = 12.3, 3.1 Hz, H-5'^{a}); 3.73 (t, 1H, *J* = 2.6 Hz, -C≡C-H); 3.61 (dd, 1H, *J* = 12.3, 3.1 Hz, H-5'^{b}).
^{13}C NMR (151 MHz, DMSO-*d6*): δ 161.06 (C-2); 159.89 (C-6); 149.47 (C-4); 133.38 (C-8); 107.13 (C-5); 89.09 (C-1'); 85.62 (C-4'); 78.99 (-CH_2-C≡$\underline{C}$); 76.02 (-CH_2-$\underline{C}$≡C); 74.06 (C-2'); 69.22 (C-3'); 60.25 (C-5'); 37.95 (-$\underline{C}H_2$-C≡C). HRMS (ESI+) m/z: calculated for $C_{13}H_{16}N_5O_5^+$: 322.11460 [M]$^+$, found 322.11458.
Supplementary Fig. 68-72

5-Propargylcytidine 5'-triphosphate (**9**)

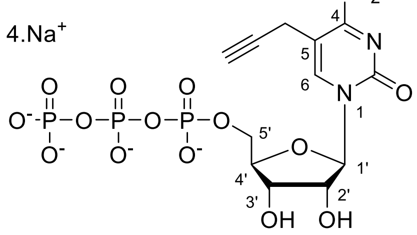

5-Propargylcytidine (**5**) (55 mg, 196 µmol, 1 eq.) was dried overnight in the pressure Schlenk flask (10 mL) under high vacuum. Trimethyl phosphate (TMP) (1.5 mL) was added under argon and the resulting mixture was stirred and cooled (ice-water bath) to form suspension. Then, phosphoryl chloride ($POCl_3$) (55 µL, 592 µmol, 3 eq.) was added directly to the suspension. The neck and argon connection of the Schlenk flask were sealed by thread lids with teflon sealing and the reaction mixture was stirred on ice-water bath until (1 hour) the suspension was changed to clear homogeneous solution. The Schlenk flask was transferred to the fridge (+ 4 °C) and left unstirred overnight.

The next day, the bis-(tri-*n*-butylammonium) pyrophosphate (430 mg, 784 µmol, 4 eq.) was dissolved in dry CH_3CN (3.5 mL) in the another Schlenk flask (10 mL) under argon and cooled by ice-water bath. The reaction mixture from day before was added dropwise to the solution of bis-(tri-*n*-butylammonium) pyrophosphate. The resulting mixture was stirred for 4 hours and then quenched by dropwise adding to the pre-cooled (ice-water bath) 2M TEAB solution (2 mL). The quenched mixture was stirred for 1 hour and then evaporated *in vacuo* (40 °C). Following step was repeated co-distillation (40 °C, gradually 100 → 20 mbar) with ddH_2O (5x 10 mL) to the solid-syrup form. The crude product was re-dissolved by ddH_2O to the final volume of 6 mL and injected onto a chromatographic column and purified by RP flash chromatography (RP-aq 130g/30µm; eluent 0.1M TEAB/CH_3CN, gradient 0-55%). The fractions containing the product (confirmed by LC-MS) were collected and evaporated *in vacuo* (40 °C). The residue was re-dissolved in ddH_2O/DMSO (8/1.5 mL) and split it to the 2 portions. Each portion was purified by ion-exchange chromatography (29 mL column POROS 50 HQ, eluent ddH_2O/1M TEAB, gradient 0-100%). The fractions containing the pure product were collected, analyzed by HRMS and freeze-dried to obtain white solid. The lyophilizate was dissolved in ddH_2O (3mL) and passed through the small gravity column (5 mL) containing the ion-exchange resin (DOWEX 50W8 in Na^+ cycle, 2 mL). The UV active fraction was freeze-dried to obtain 2.3 mg (yield: 2%) of compound **9** as tetrasodium salt as a white lyophilizate.

^1H NMR (600 MHz, D_2O): δ 7.99 [s, 1H, H-6]; 6.04 [d, 1H, *J* = 4.7 Hz, H-1']; 4.45 [t, 1H, *J* = 4.9 Hz, H-3']; 4.37 [t, 1H, *J* = 4.9 Hz, H-2']; 4.32 [m, 3H, H-4', H-5'[a], H-5'[b]]; 3.47 [d, 2H, *J* = 2.7 Hz, -$\underline{CH_2}$-C≡C]; 2.65 [t, 1H, *J* = 2.7 Hz, -C≡C-H].

^{13}C NMR (151 MHz, D_2O): δ 164.75 [C-4]; 157.55 [C-2]; 139.72 [C-6]; 104.04 [C-5]; 89.25 [C-1']; 83.10 and 83.05 [C-4']; 80.02 [-$\underline{C}$≡C-H]; 74.40 [C-2']; 72.36 [-C≡$\underline{C}$-H]; 69.45 [C-3']; 64.99 [C-5']; 17.33 [-$\underline{C}H_2$-C≡C].

^{31}P NMR (243 MHz, D_2O): δ -9.59, -11.40, -22.72.

HRMS (ESI-) m/z: calculated for $C_{12}H_{18}N_3O_{14}P_3$: 519.99288 $[M - H]^-$, found 519.99269.

Supplementary Fig. 73-78

Notes for monitoring the reaction (Compound **9**): The reaction monitoring was realized by LC-MS: Before transferring to the fridge, directly before and after (5 min, 1 hour and 2.5 hours) adding to the bis-(tri-*n*-butylammonium) pyrophosphate solution, after quench.

Analytical sample preparation: 50 µL of reaction mixture was quenched by 200 µL of 2M TEAB and then diluted to 1,5 mL by ddH_2O. The mixture was vigorously stirred (vortex) and filtrated by syringe filter (PVDF, 0.22 µm). The sample was analyzed by LC method (eluent 20 mM NH_4OAc buffer (pH = 6.0)/CH_3CN, gradient 0-30%, column temperature = 20 °C) with MS method (scan 100-1000 m/z, ESI positive/negative, 200ms/200ms, Fragmentor = 100V), (ion-source: main gas temperature = 250 °C, sheat gas temperature = 375 °C, capillary voltage: 2500V [pos] / 4000V [neg], nozzle voltage: 0V [pos] / 1500V [neg]).

Supplementary References

1. Li, X. et al. Photochemical conversion of a cytidine derivative to a thymidine analog via [2+2]-cycloaddition. *Photochem. Photobiol. Sci.* **17**, 1049-1055 (2018).
2. Sun, Q. et al. Efficient synthesis of 5-hydroxymethyl-, 5-formyl-, and 5-carboxyl-2'-deoxycytidine and their triphosphates. *RSC Adv.* **4**, 36036-36039 (2014).
3. Venkatesham, A. et al. Propargylated Purine Deoxynucleosides: New Tools for Fluorescence Imaging Strategies. *Molecules* **24**, 468 (2019).
5. Buchbender, A. et al. Improved library preparation with the new iCLIP2 protocol. *Methods* **178**, 33-48 (2020).
