# Supplementary material for: MePMe-seq: antibody-free simultaneous m6A and m5C mapping in mRNA by metabolic propargyl labeling and sequencing
Source: Nat Commun. 2023 Nov 7;14:7154. doi: 10.1038/s41467-023-42832-z (PMC10630376; doi:10.1038/s41467-023-42832-z)
Supplement: Supplementary file 2 — Description of Additional Supplementary Files [file 41467_2023_42832_MOESM2_ESM.pdf]

Description of Additional Supplementary Information Files

Supplementary Data 1: JACUSA2 filtered MePMe-seq data for both replicates including all identified modified nucleotides.

Supplementary Data 2: JACUSA2 filtered MePMe-seq data for both replicates including all identified m6A sites in mRNA.

Supplementary Data 3: Comparison of via MePMe-seq identified m6A sites in mRNA with other methods providing single nucleotide resolution.

Supplementary Data 4: JACUSA2 filtered data from in vitro METTL16 labeling for both replicates including all identified modified nucleotides.

Supplementary Data 5: JACUSA2 filtered data from in vitro METTL16 labeling for both replicates including all identified m6A sites in mRNA.

Supplementary Data 6: JACUSA2 filtered MePMe-seq data for both replicates including all identified m5C sites in mRNA.

Supplementary Data 7: Comparison of via MePMe-seq identified m5C sites in mRNA with other methods.
