# Supplementary material for: MePMe-seq: antibody-free simultaneous m6A and m5C mapping in mRNA by metabolic propargyl labeling and sequencing
Source: Nat Commun. 2023 Nov 7;14:7154. doi: 10.1038/s41467-023-42832-z (PMC10630376; doi:10.1038/s41467-023-42832-z)
Supplement: Supplementary file 10 — Reporting Summary [file 41467_2023_42832_MOESM10_ESM.pdf]

nature portfolio

Corresponding author(s): Andrea Rentmeister

Last updated by author(s): Oct 20, 2023

Reporting Summary

Nature Portfolio wishes to improve the reproducibility of the work that we publish. This form provides structure for consistency and transparency in reporting. For further information on Nature Portfolio policies, see our Editorial Policies and the Editorial Policy Checklist.

Statistics

For all statistical analyses, confirm that the following items are present in the figure legend, table legend, main text, or Methods section.

n/a	Confirmed	
☐	☒	The exact sample size (*n*) for each experimental group/condition, given as a discrete number and unit of measurement
☐	☒	A statement on whether measurements were taken from distinct samples or whether the same sample was measured repeatedly
☐	☒	The statistical test(s) used AND whether they are one- or two-sided<br>*Only common tests should be described solely by name; describe more complex techniques in the Methods section.*
☒	☐	A description of all covariates tested
☐	☒	A description of any assumptions or corrections, such as tests of normality and adjustment for multiple comparisons
☐	☒	A full description of the statistical parameters including central tendency (e.g. means) or other basic estimates (e.g. regression coefficient) AND variation (e.g. standard deviation) or associated estimates of uncertainty (e.g. confidence intervals)
☐	☒	For null hypothesis testing, the test statistic (e.g. *F*, *t*, *r*) with confidence intervals, effect sizes, degrees of freedom and *P* value noted<br>*Give P values as exact values whenever suitable.*
☒	☐	For Bayesian analysis, information on the choice of priors and Markov chain Monte Carlo settings
☒	☐	For hierarchical and complex designs, identification of the appropriate level for tests and full reporting of outcomes
☒	☐	Estimates of effect sizes (e.g. Cohen's *d*, Pearson's *r*), indicating how they were calculated

Our web collection on statistics for biologists contains articles on many of the points above.

Software and code

Policy information about availability of computer code

Data collection	Bio-Rad CFX Manager Software V 3.1, Qualitative Navigator B.08.00, Agilent Openlab CDS ChemStationdition Rev. C.01.10, 2100 Expert Software, LC/MS data Acquisition for ULTIVO LC/TQ Version C.01.00, Optimizer for Ultivo LC/TQ Version C.01.00, Offline Method Editor for ULTIVO LC/TQ Version C.01.00, Typhoon FLA 9500 , Bioanalyzer 2100 Expert Version B.02.10.SI764
Data analysis	Bio-Rad CFX Maestro 1.0 (4.0.2325.0418), OriginPro 2019b (32-bit) 9.6.5.169, ImageJ (Version 20160205), Adobe Illustrator CS6, MestReNova 14, Excel 2016, Word 2016, Adobe Acrobat Pro 2017, PerkinElmer ChemDraw 20.1, Jupyter Notebook 1.9.12, Clone Manager 9.51, ChemBioDraw Ultra 14.0, Agilent Mass Hunter Qualitative Analysis Navigator, Agilent Mass Hunter Quantitative Analysis for QqO, Intas GDC, samtools 1.8, hisat2 2.1.0, FASTQC 0.11.7, fastp 0.20.1, UMI-tools 1.0.1, bedtools V.2.29.2-25-g3dd63c4, R 4.0.2, ggseqlogo 0.1, rstudio 1.3.959, R ver. 4.0.2, ensembldb ver. 2.14.l,ggplot2 library ver. 3.3.2 IGV 2.4.14, Bioanalyzer 2100 Expert Version B.02.10.SI764

For manuscripts utilizing custom algorithms or software that are central to the research but not yet described in published literature, software must be made available to editors and reviewers. We strongly encourage code deposition in a community repository (e.g. GitHub). See the Nature Portfolio guidelines for submitting code & software for further information.

Data

Policy information about availability of data

All manuscripts must include a data availability statement. This statement should provide the following information, where applicable:

- Accession codes, unique identifiers, or web links for publicly available datasets
- A description of any restrictions on data availability
- For clinical datasets or third party data, please ensure that the statement adheres to our policy

The mapped data generated in this study for both replicates of MePMe-seq and in vitro METTL16 labeling in HeLa cells have been deposited in the NCBI Sequence Read Archive (SRA) under the accession code https://www.ncbi.nlm.nih.gov/sra/PRJNA811414. The processed data shown in this study, including JACUSA2 sites from both replicates of MePMe-seq (Supplementary data 1), identified m6A sites from MePMe-seq (Supplementary data 2), identified m5C sites from MePMe-seq (Supplementary data 6), JACUSA2 sites from both replicates of in vitro METTL16 labeling (Supplementary data 4) and identified m6A sites from in vitro METTL16 labeling (Supplementary data 5) are provided in the Supplementary data. The public datasets of PA-m6A-CLIP, DART-seq, miCLIP, m6A-REF-seq were downloaded from ATLAS database, public datasets of eTAM-seq, m6A-SAC-seq, GLORI were downloaded from GEO database (GSE211303, GSE210563, GSE198246). The genome hg38 was downloaded from the following link: http://apr2019.archive.ensembl.org/index.html. Source data are provided with this paper.

Research involving human participants, their data, or biological material

Policy information about studies with human participants or human data. See also policy information about sex, gender (identity/presentation), and sexual orientation and race, ethnicity and racism.

Reporting on sex and gender	/
Reporting on race, ethnicity, or other socially relevant groupings	/
Population characteristics	/
Recruitment	/
Ethics oversight	/

Note that full information on the approval of the study protocol must also be provided in the manuscript.

Field-specific reporting

Please select the one below that is the best fit for your research. If you are not sure, read the appropriate sections before making your selection.

☒ Life sciences ☐ Behavioural & social sciences ☐ Ecological, evolutionary & environmental sciences

For a reference copy of the document with all sections, see nature.com/documents/nr-reporting-summary-flat.pdf

Life sciences study design

All studies must disclose on these points even when the disclosure is negative.

Sample size	This study did not involve experiments with living animals. Thus, it was not necessary to define sample sizes in advance to ensure adequate statistical power. Sample size, number of replicates and statistical tests were chosen based on accepted practices in the field. In general, 3 or more biological replicates were performed for most experiments with the exception of NGS sequencing experiments. Accurate number of replicates are stated with each experiment.
Data exclusions	For SELECT, datasets for other m6A-sites with a normalized ΔCq significantly smaller than 1 for +FTO were excluded, as it would mean that after demethylation via FTO As were N6-methylated.
Replication	All experiments were indepenently repeated 2-5 times as indicated in the figure legends.
Randomization	This is not a clinical trial. No animals or patients that would require randomization were involved. When individual cells were analyzed in cell populations, they were arbitrarily selected from these populations.
Blinding	This is not a clinical trial. No animals or patients were involved. For gel analysis and NGS analysis blinding is not possible because the samples need to be arranged and analyzed with knowledge of the sample identity.

Reporting for specific materials, systems and methods

We require information from authors about some types of materials, experimental systems and methods used in many studies. Here, indicate whether each material, system or method listed is relevant to your study. If you are not sure if a list item applies to your research, read the appropriate section before selecting a response.

Materials & experimental systems

n/a	Involved in the study
☒	☐ Antibodies
☐	☒ Eukaryotic cell lines
☒	☐ Palaeontology and archaeology
☒	☐ Animals and other organisms
☒	☐ Clinical data
☒	☐ Dual use research of concern
☒	☐ Plants

Methods

n/a	Involved in the study
☒	☐ ChIP-seq
☒	☐ Flow cytometry
☒	☐ MRI-based neuroimaging

Eukaryotic cell lines

Policy information about cell lines and Sex and Gender in Research

Cell line source(s)	HeLa cells (Merck, 93021013), Sf21 insect cells (Thermo Fisher, 11497013)
Authentication	The cell lines were obtained from commercial suppiers (Merkck/Thermo Fisher). No additional authentication was performed.
Mycoplasma contamination	Cells were tested negative for Myoplasma contamination.
Commonly misidentified lines (See ICLAC register)	No commonly misidentified cell lines were used in this study.

Plants

Seed stocks	*Report on the source of all seed stocks or other plant material used. If applicable, state the seed stock centre and catalogue number. If plant specimens were collected from the field, describe the collection location, date and sampling procedures.*
Novel plant genotypes	*Describe the methods by which all novel plant genotypes were produced. This includes those generated by transgenic approaches, gene editing, chemical/radiation-based mutagenesis and hybridization. For transgenic lines, describe the transformation method, the number of independent lines analyzed and the generation upon which experiments were performed. For gene-edited lines, describe the editor used, the endogenous sequence targeted for editing, the targeting guide RNA sequence (if applicable) and how the editor was applied.*
Authentication	*Describe any authentication procedures for each seed stock used or novel genotype generated. Describe any experiments used to assess the effect of a mutation and, where applicable, how potential secondary effects (e.g. second site T-DNA insertions, mosiacism, off-target gene editing) were examined.*
